# A comparative gas chromatography-mass spectrometry (GC-MS) profiling of Egyptian and Indian ashwagandha (*Withania somnifera*) root extracts

**DOI:** 10.1038/s41598-025-25896-3

**Published:** 2025-11-21

**Authors:** Mohamed M. Elghazaly, Yasmeen M. Gawaan, Shereen Abdelkader, Eman Hashem Radwan, Hadeer M. El-Sayed

**Affiliations:** Zoology Department, Faculty of Science, Damanhour University, Damanhour, Egypt

**Keywords:** Egyptian ashwagandha, Indian ashwagandha, Phytoconstituents, Gas Chromatography-Mass spectrometry, Biochemistry, Chemical biology, Chemistry, Drug discovery, Plant sciences

## Abstract

Ashwagandha (*Withania somnifera*) is a woody shrub that grows up to 2 feet in height. Ashwagandha roots extract has been used as a traditional herbal medicine for centuries because it has phytochemicals. This study aimed to analyse the chemical composition of both the Egyptian and Indian ashwagandha roots’ extracts by Gas Chromatography-Mass Spectrometry (GC-MS). Both extracts analysis by GC-MS found that they contain essential compounds that have important health benefits. Both Egyptian and Indian ashwagandha extracts contain phytosterols and fatty acids with different area percentages. From the comparison between the ashwagandha extracts of the present study, it is revealed that the Egyptian ashwagandha extract contains higher area percentages of campesterol (28.70%), stigmasterol (16.11%), n-hexadecanoic acid (17.43%), octadecanoic acid (2.59%), 1-heptatriacotanol (1.64%) and atropine (1.49%) than those present in the Indian extract. On the other side, the Indian ashwagandha extract contains higher area percentages of ҫ-sitosterol (20.34%), oleic acid (9.14%), 9,12-octadecadienoic acid (Z, Z) (8.62%) and hexadecanoic acid, methyl ester (0.96%) than those present in the Egyptian extract. Depending on the analysis of the Egyptian and Indian ashwagandha extracts, it is concluded that both extracts contain essential bioactive compounds with vital effects on human health. The Egyptian ashwagandha extract is available, low cost and contains higher area percentages in the most bioactive compounds than were found in the Indian ashwagandha extract.

**Supplementary Information:**

The online version contains supplementary material available at 10.1038/s41598-025-25896-3.

## Introduction

 Ashwagandha (*Withania somnifera*) is a small, typically upright, woody shrub that belongs to the Solanaceae family. This plant can reach a height of up to 2 feet. Its roots are fleshy, whitish-brown and covered in bristles. The roots are the primary part of the plant and are used for therapeutic purposes. The leaves are simple, ovate, smooth, petiolate and smaller, arranged oppositely. The flowers are greenish or yellow and grow in axillary, umbellate clusters; the small berries are globular and turn orange-red when they ripen, encapsulated in a persistent calyx; the seeds are yellow and reniform. It grows throughout the drier regions and subtropical areas of India, as well as in other countries such as Pakistan, Egypt, Jordan, Morocco and Eastern and South Africa^1^.

Ashwagandha is an Ayurvedic herb that is also known as Indian winter cherry and Indian ginseng. It has been used for centuries in India for its various beneficial health activities in stress management, energy elevation and improving cognitive health^2^. Various metabolites derived from Ashwagandha leaves and root extracts have therapeutic efficacy^3^.

It was reported that Ashwagandha roots extract consumption for 8 weeks was safe in both males and females volunteers^4^. Ashwagandha contains a wide range of phytochemicals. Many studies have used Ashwagandha roots extract, either alone or in combination with other natural plants, for a variety of biomedical purposes. These include its anti-microbial, anti-inflammatory, anti-stress, anti-tumour, cardio-protective and neuroprotective qualities. It also enhances endothelial function, reduces reactive oxygen species, regulates apoptosis and enhances mitochondrial function as well as the treatment of age-related symptoms, anxiety, neurodegenerative diseases, diabetes, stress, arthritis, fatigue and cognitive/memory impairment^5^. In last years, ashwagandha has attracted a lot of attention as an adaptogen that helps sleep, reduces stress and has variety of health related benefits^6^.

## Material & methods

### Plant collection

**Fig. 1:**
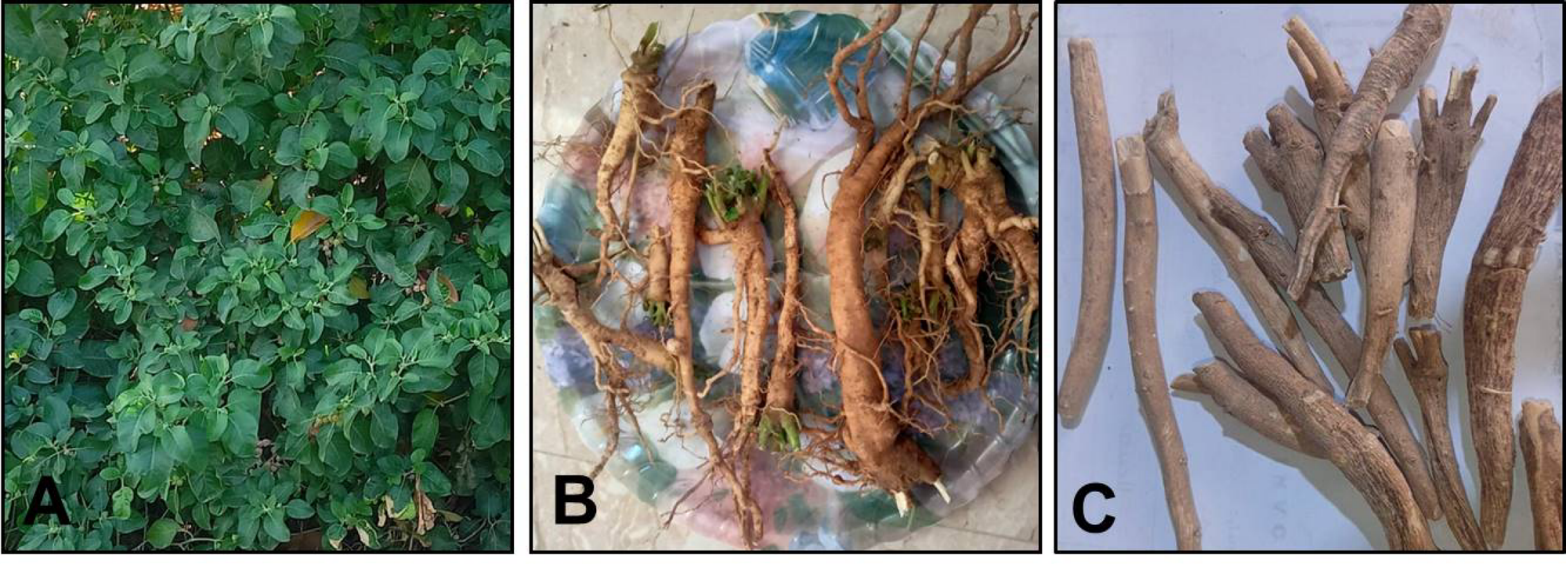
**(A)** Egyptian Ashwagandha plant, (**B**) Egyptian ashwagandha roots & (**C**) Indian Ashwagandha roots.

Ashwagandha plant grows in many places in Egypt (Fig. 1A) and its roots (Fig. 1B) were collected from Faculty of Agriculture nursery, Alexandria University, Egypt and the plant was identified as *Withania somnifera* according to the plant atlas of Faculty of Science, Alexandria University, Egypt. The collected roots were left at room temperature to dry. While dried Indian Ashwagandha roots (Fig. 1C) were purchased from Haraz shop for natural products, Cairo, Egypt.

### Preparation of Ashwagandha roots’ extracts

The dried roots of both Egyptian and Indian Ashwagandha were ground into fine powders. Powdered roots of both Egyptian and Indian Ashwagandha were macerated in a stoppered container with 50 ml of 85% methanol and left at room temperature for 48 h. The extracts were filtered and concentrated using a rotary evaporator under a vacuum at 40° C to evaporate the solvent and obtain crude extracts^7^. The extraction processes were done in Faculty of Agriculture, Alexandria University, Alexandria, Egypt.

### Gas chromatography–mass spectrometry (GC-MS) analysis

The chemical composition analysis was performed using GC-TSQ mass spectrometer (Thermo Scientific, Austin, TX, USA) with a direct capillary column TG-5MS (30 m x 0.25 mm x 0.25 μm film thickness). The column oven temperature was initially held at 60 °C and then increased by 5 °C/min to 250 °C withhold 2 min then increased to 300 with 30 C/min. The injector temperature was kept at 270 °C. Helium was used as a carrier gas at a constant flow rate of 1 ml/min. The solvent delay was 4 min and diluted samples of 1 µl were injected automatically using Autosampler AS3000 coupled with GC in the split mode. EI mass spectra were collected at 70 eV ionization voltages over the range of m/z 50–650 in full scan mode. The ion source and transfer line were set at 200 °C and 280 °C, respectively. The components were identified by comparing their mass spectra to those found in the Wiley Registry and NIST14 mass spectral database^8^.

## Results

**Fig. 2:**
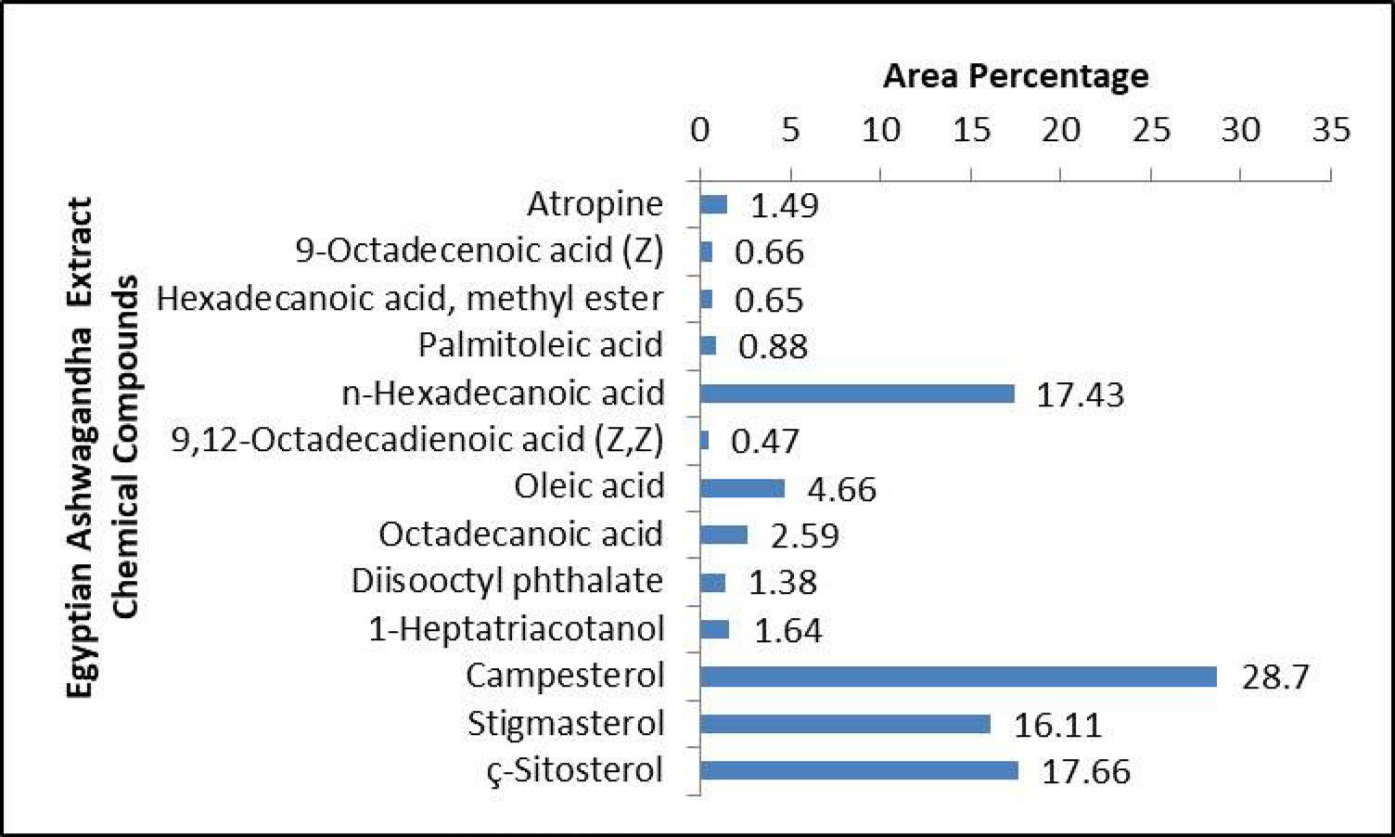
Histogram of the Egyptian Ashwagandha extract chemical compounds and their area percentage.

**Fig. 3:**
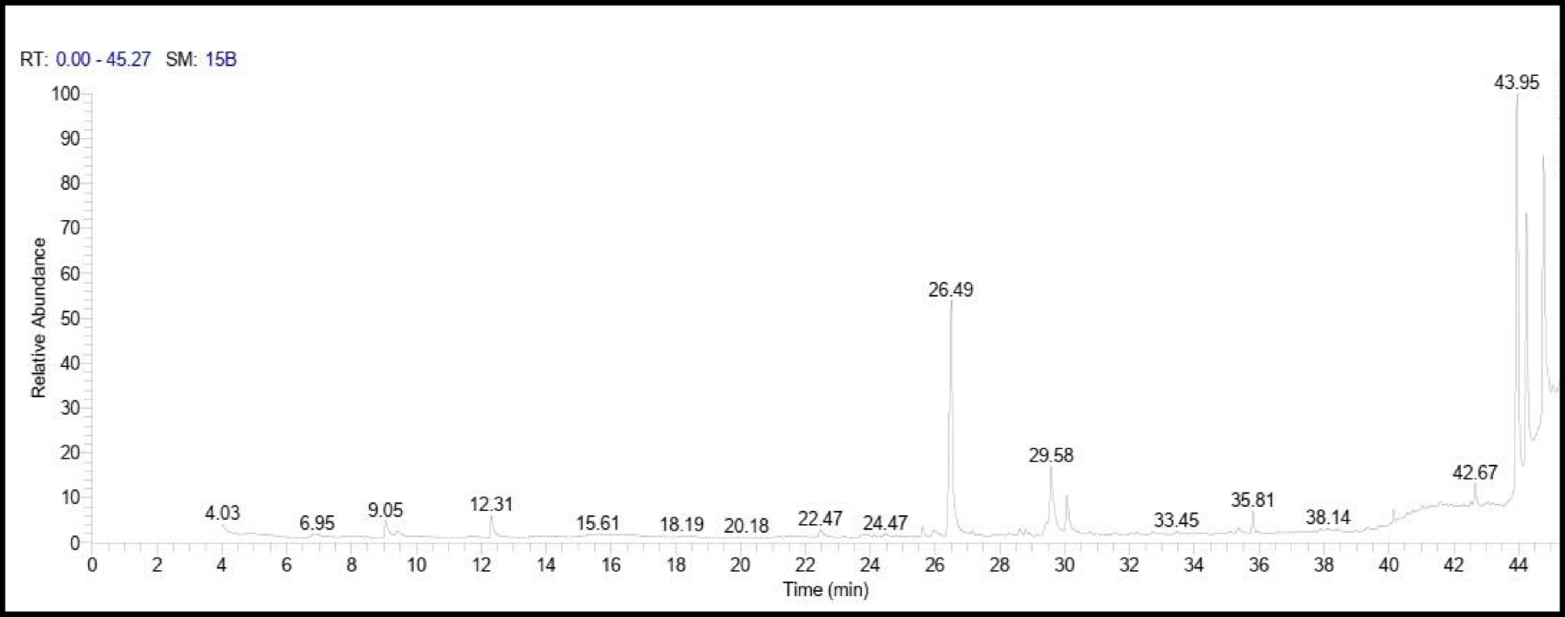
Hit spectrum of the Egyptian Ashwagandha extract.

**Fig. 4:**
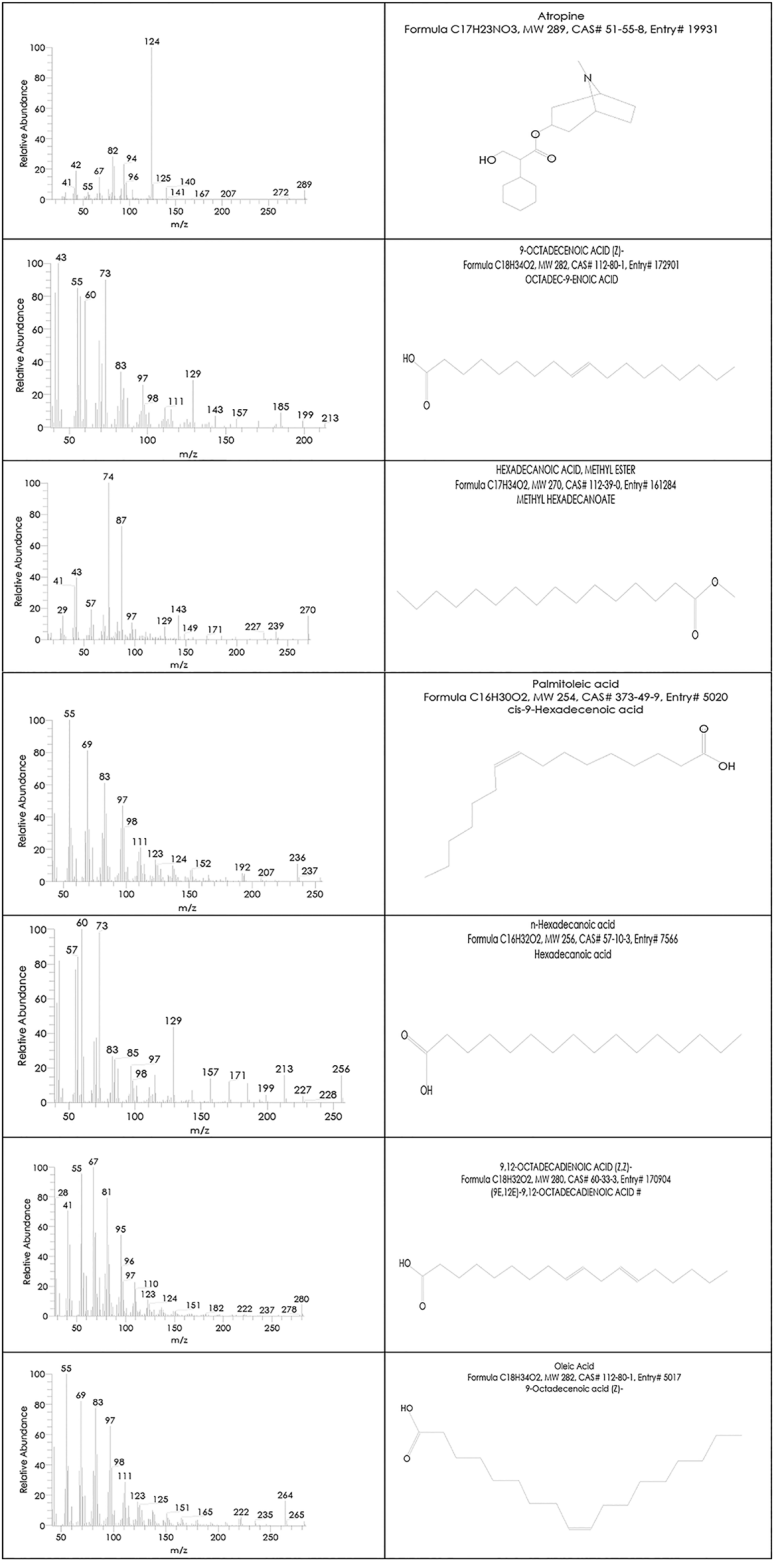

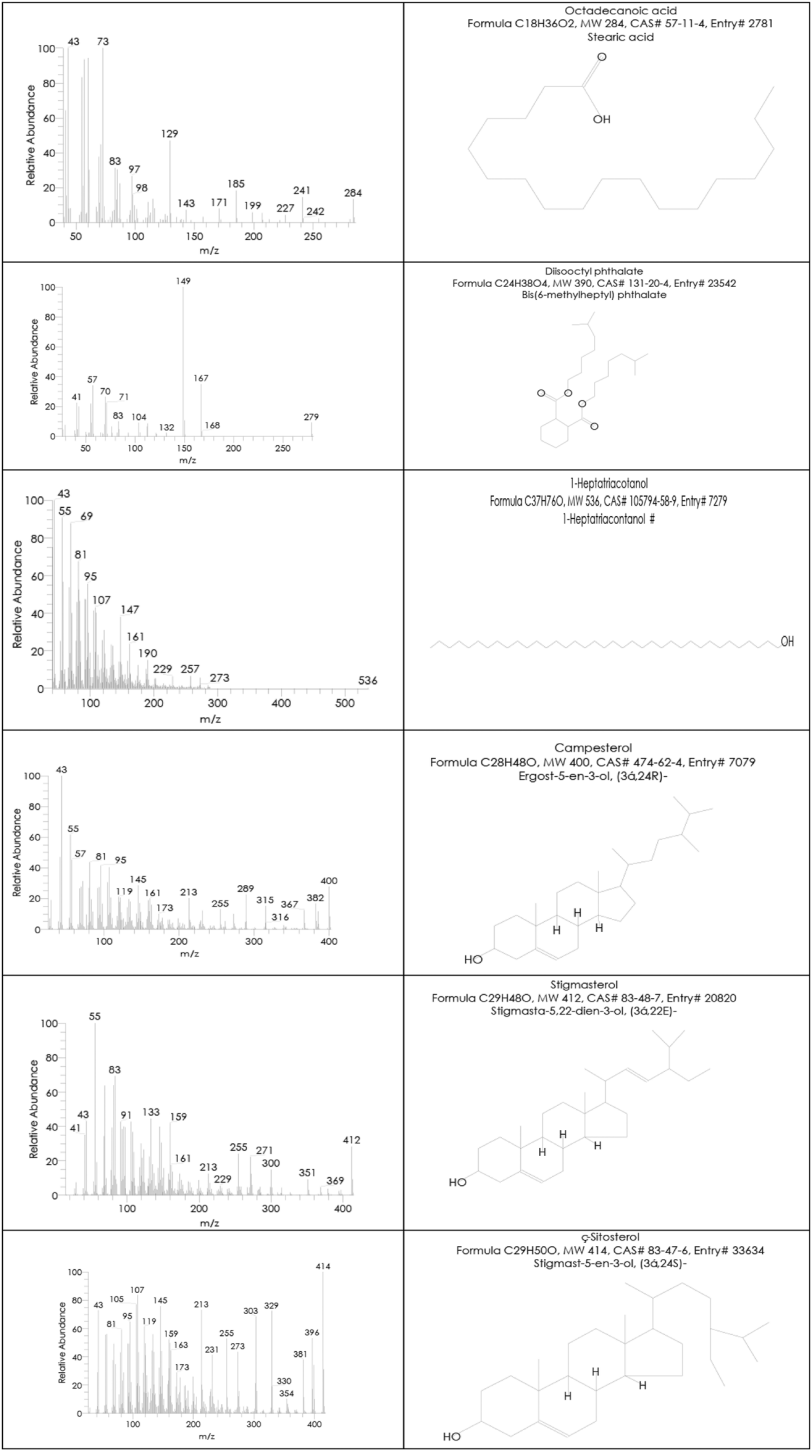
List of Hit spectrum and chemical structure of the Egyptian Ashwagandha bioactive compounds.

Egyptian Ashwagandha roots extract components analysis by GC-MS showed that it contains numerous essential bioactive compounds with several biological activities. The most important compounds found in the Egyptian Ashwagandha roots extract are listed in Table 1; Fig. 2 and their peaks and chemical structures are shown in Figs. 3 and 4. From the GC-MS obtained data, the Egyptian Ashwagandha roots extract contains high area percentages of phytosterols; campesterol (28.70%), stigmasterol (16.11%) and ҫ-sitosterol (17.66%) in addition to numerous fatty acids; n-hexadecanoic acid (17.43%), oleic acid (4.66%), octadecanoic acid (2.59%),1-heptatriacotanol (1.64%), palmitoleic acid (0.88%), 9-octadecenoic acid (Z) (0.66%), hexadecanoic acid, methyl ester (0.65%) and 9,12-octadecadienoic acid (Z, Z) (0.47%), besides other phytochemicals as atropine (1.49%) and diisooctyl phthalate (1.38%).

**Fig. 5:**
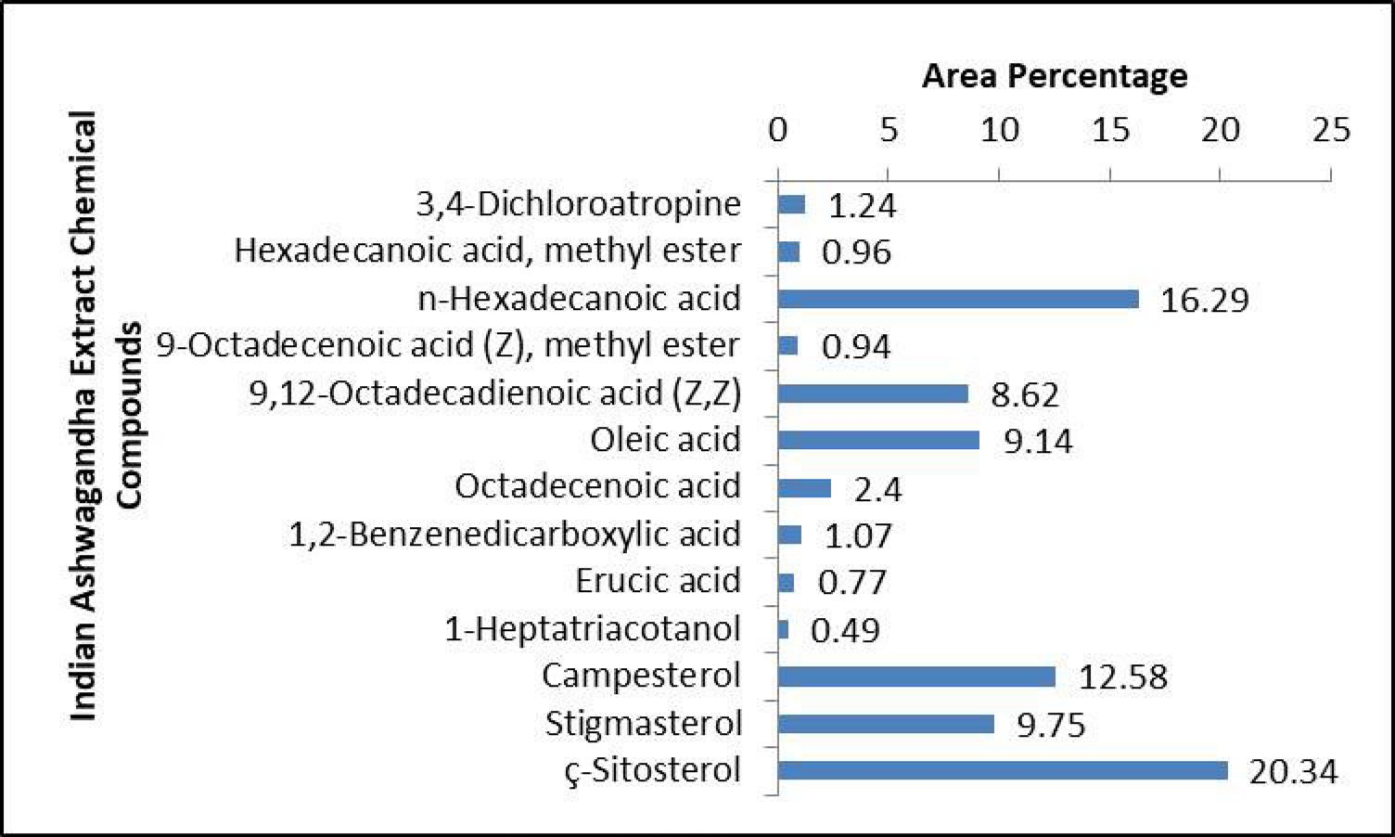
Histogram of the Indian Ashwagandha extract chemical compounds and their area percentage.

**Fig. 6:**
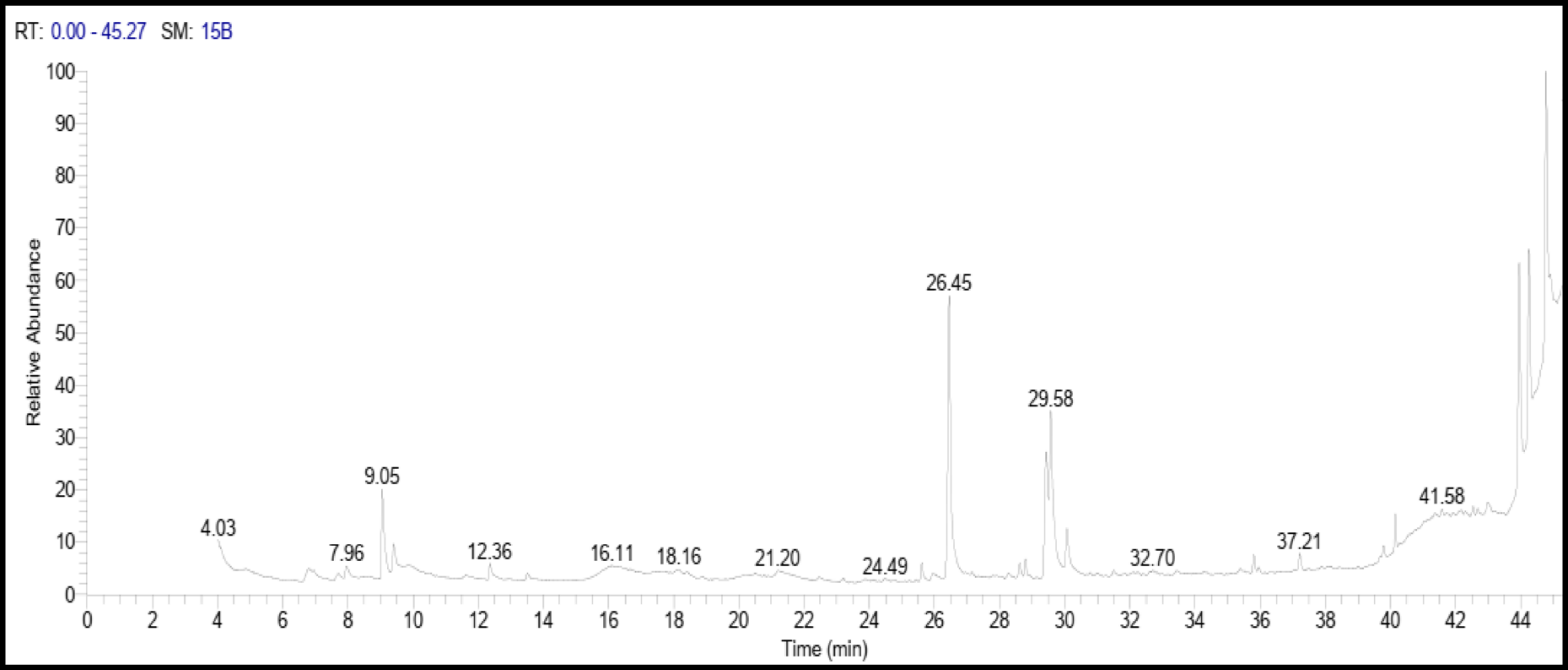
Hit spectrum of Indian Ashwagandha extract.

**Fig. 7:**
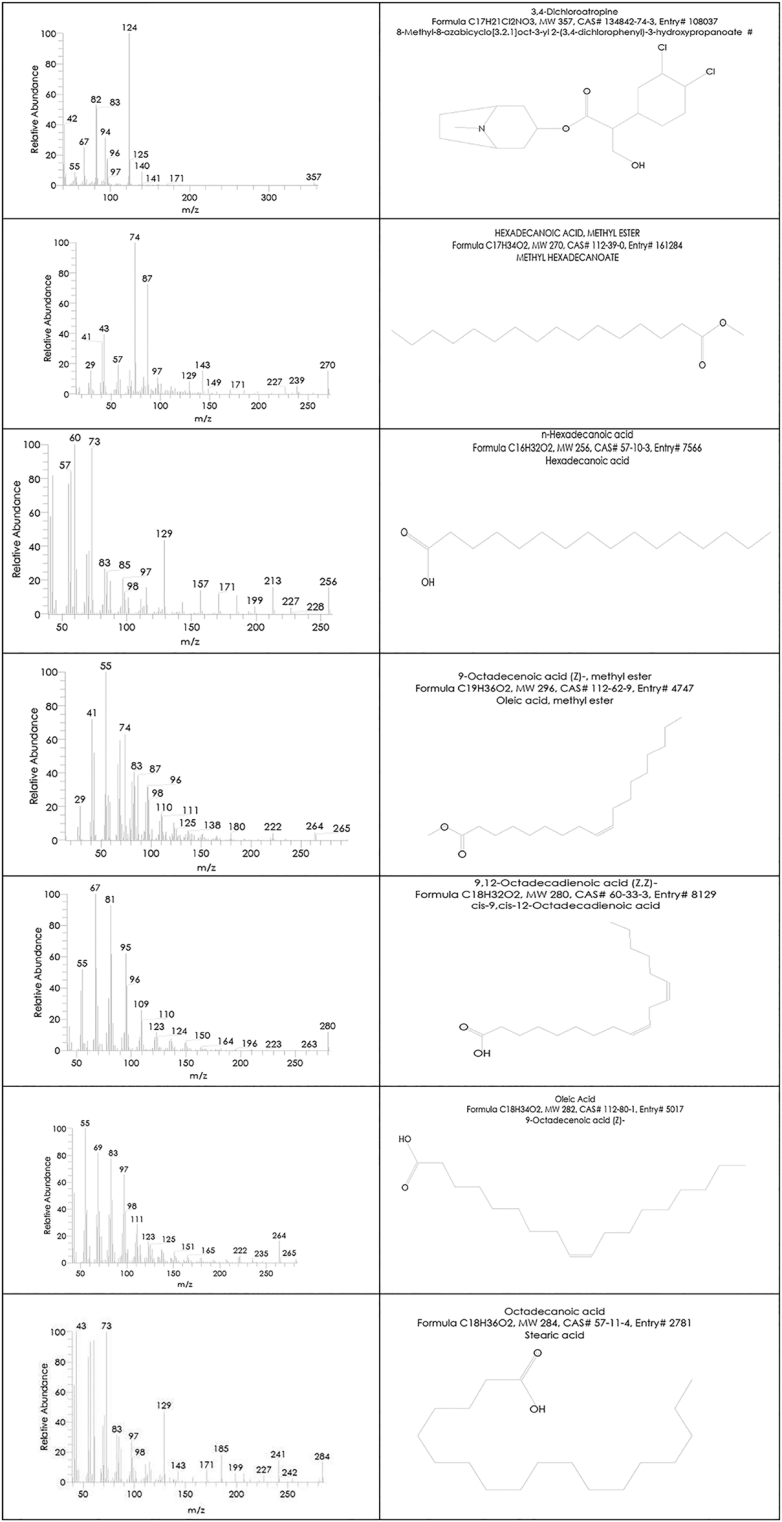

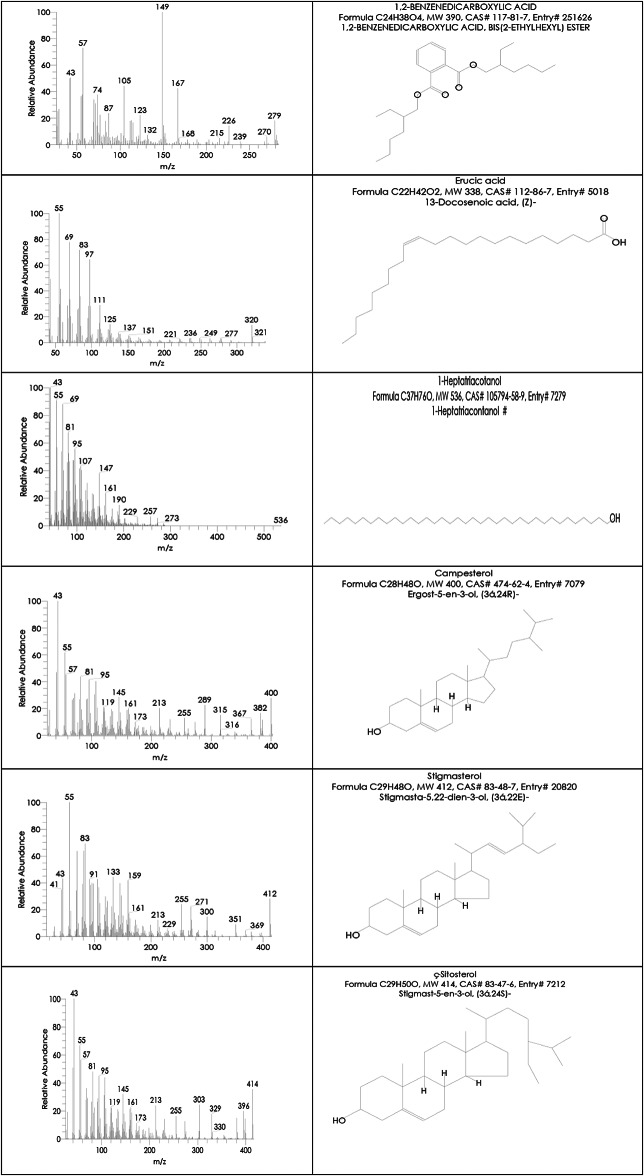
List of Hit spectrum and chemical structures of the Indian Ashwagandha bioactive compounds.

On the other side, the Indian Ashwagandha roots extract components analysis by GC-MS revealed that it also contains numerous essential bioactive compounds relatively similar to those in the Egyptian Ashwagandha roots extract with different area percentages. These effective compounds are listed in Table 2; Fig. 5 and their peaks and chemical structures are shown in Figs. 6 and 7. The Indian extract contains high area percentages of phytosterols; ҫ-sitosterol (20.34%), campesterol (12.58%) and stigmasterol (9.75%) in addition to numerous fatty acids; n-hexadecanoic acid (16.29%), oleic acid (9.14%), octadecanoic acid (2.40%), 9,12-octadecadienoic acid (Z, Z) (8.62%), 1,2-benzenedicarboxylic acid (1.07%), hexadecanoic acid, methyl ester (0.96%), 9-octadecenoic acid (Z), methyl ester (0.94%), erucic acid (0.77%) and 1-heptatriacotanol (0.49%), besides the presence of 3,4-dichloroatropine (1.24%).

From the comparison between the Ashwagandha extracts of the present study, it is revealed that the Egyptian Ashwagandha extract contains higher area percentages of campesterol (28.70%), stigmasterol (16.11%), n-hexadecanoic acid (17.43%), octadecanoic acid (2.59%), 1-heptatriacotanol (1.64%) and atropine (1.49%) than those present in the Indian extract. On the other side, the Indian ashwagandha extract contains a higher area percentage of ҫ-sitosterol (20.34%), oleic acid (9.14%), 9,12-octadecadienoic acid (Z, Z) (8.62%) and hexadecanoic acid, methyl ester (0.96%) than those present in the Egyptian extract.

**Table 1:** Detected compounds by GC-MS in the Egyptian Ashwagandha extract.

Retention time	Compound name	Area percentage	Matching factor	Molecular formula
12.31	Atropine	1.49	816	C_17_H_23_NO_3_
22.46	9-Octadecenoic acid (Z)	0.66	875	C_18_H_34_O_2_
25.61	Hexadecanoic acid, methyl ester	0.65	860	C_17_H_34_O_2_
25.97	Palmitoleic acid	0.88	856	C_16_H_30_O_2_
26.50	n-Hexadecanoic acid	17.43	924	C_16_H_32_O_2_
29.43	9,12-Octadecadienoic acid (Z, Z)	0.47	825	C_18_H_32_O_2_
29.58	Oleic acid	4.66	908	C_18_H_34_O_2_
30.07	Octadecanoic acid	2.59	932	C_18_H_36_O_2_
35.81	Diisooctyl phthalate	1.38	940	C_24_H_38_O_4_
42.67	1-Heptatriacotanol	1.64	744	C_37_H_76_O
43.95	Campesterol	28.70	868	C_28_H_48_O
44.25	Stigmasterol	16.11	867	C_29_H_48_O
44.77	ҫ-Sitosterol	17.66	824	C_29_H_50_O

**Table 2:** Detected compounds by GC-MS in the Indian Ashwagandha extract.

Retention time	Compound name	Area percentage	Matching factor	Molecular formula
12.35	3,4-Dichloroatropine	1.24	765	C_17_H_21_Cl_2_NO_3_
25.61	Hexadecanoic acid, methyl ester	0.96	843	C_17_H_34_O_2_
26.46	n-Hexadecanoic acid	16.29	932	C_16_H_32_O_2_
28.79	9-Octadecenoic acid (Z), methyl ester	0.94	883	C_19_H_36_O_2_
29.43	9,12-Octadecadienoic acid (Z, Z)	8.62	912	C_18_H_32_O_2_
29.58	Oleic acid	9.14	893	C_18_H_34_O_2_
30.06	Octadecenoic acid	2.40	897	C_18_H_36_O_2_
35.81	1,2-Benzenedicarboxylic acid	1.07	773	C_24_H_38_O_4_
39.79	Erucic acid	0.77	677	C_22_H_42_O_2_
42.53	1-Heptatriacotanol	0.49	723	C_37_H_76_O
43.95	Campesterol	12.58	796	C_28_H_48_O
44.24	Stigmasterol	9.75	812	C_29_H_48_O
44.77	ҫ-Sitosterol	20.34	735	C_29_H_50_O

##  Discussion

The obtained data from the present study revealed that both Egyptian and Indian Ashwagandha extracts contain phytosterols, fatty acids and other essential phytochemicals indicating their possible therapeutic potential.

Phytosterols are plant sterols that are vital secondary plant metabolites^9^. Campesterol, stigmasterol and sitosterol are the main and significant phytosterols in plants^10^. As these compounds are vital biomolecules and have beneficial effects on human health, they must be taken from foods^11^. It is well known that phytosterols have anti-cancer, anti-inflammatory, cholesterol-lowering and immune-protective activities^12^. The biological activities of stigmasterol as phytosterol have been studied through in vitro and in vivo pathways and its potent pharmacological capabilities such as anti-cancer, anti-oxidant, anti-inflammatory, anti-osteoarthritis, anti-diabetic, immunomodulatory, anti-parasitic, anti-fungal, anti-bacterial and neuroprotective properties have been documented^13^. Campesterol is a well-known phytosterol and it is documented that it has anti-cancer, anti-oxidant and hypocholesterolemic effects^14^ in addition to anti-inflammatory potential^15^. β-sitosterol performs various activities such as hepatoprotective and cholesterol-lowering potentials^16^, insulin-like biological activity^17^, anti-inflammatory and anti-oxidant effects^18^.

Fatty acids are fat-soluble portions of plants or animals, which are the basic components of lipids. Fatty acids can be saturated or unsaturated, depending on the presence of double bonds. Unsaturated fatty acids are also classified into mono and polyunsaturated fatty acids based on the number of double bonds^19^. n-Hexadecanoic acid is known as palmitic acid. It is the most prevalent saturated fatty acid in animals, plants and microorganisms and performs numerous basic biological functions at cellular and tissue levels^20^. It was documented that n-hexadecanoic acid has anti-inflammatory properties^21^, anti-oxidant and hypocholesterolemic potentials^22^. Hexadecanoic acid, methyl ester is documented that it has anti-inflammatory, cancer-preventive, hepatoprotective, anti-arthritic and anti-coronary potentials^23^.

Oleic acid is a monounsaturated fatty acid generally known for its low-density lipoprotein lowering effect, a slight anti-inflammatory effect and insulin-regulating activity^24^ and it also has anti-microbial and anti-oxidant activities^22^.

Octadecanoic acid is a fatty acid that has anti-microbial efficacy^25^, anti-inflammatory and anti-cancer effects^26^. 9,12-Octadecadienoic acid (Z, Z) is a fatty acid that has anti-inflammatory, hypocholesterolemic, cancer preventive, hepatoprotective, anti-histaminic, anti-eczemic, anti-acne, 5-α reductase inhibitor, anti-androgenic, anti-arthritic, anti-coronary and anti-microbial activities^27^. 9-Octadecenoic acid (Z), a fatty acid, can suppress rheumatoid arthritis and different types of cancer^28^. 9-Octadecenoic acid (Z), methyl ester, a fatty acid methyl ester, has anti-inflammatory and anti-cancer activities^29^.

Palmitoleic acid, a monounsaturated omega-7 fatty acid, has various beneficial effects; it suppresses lipogenesis in the liver and muscles (toxic storage sites) and on the contrary, it stimulates lipogenesis in adipose tissues (safe storage sites). It is related to ameliorated insulin sensitivity and lipid profile in addition to a decreased risk of type 2 diabetes and cardiovascular diseases including myocardial infarction^30^. 1-Heptatriacotanol, a fatty acid, has anti-oxidant, anti-cancer and anti-inflammatory properties, besides coronary heart disease treatment^31^.

Erucic acid, a monounsaturated fatty acid, has been reported to have side effects on health, particularly myocardial lipidosis and hepatic steatosis^32^. But, erucic acid can be converted into nervonic acid, a main component of myelin. So, it has remyelinating potential in demyelinating conditions treatment and it also has neurodegenerative disorders treatment potential through anti-oxidant and anti-inflammatory effects^33^.

Atropine is a tropane alkaloid that belongs to secondary metabolites and can be isolated from many plant species of the Solanaceae family^34^. It is documented that atropine has numerous medicinal applications including anti-cancer activity by suppressing epithelial-mesenchymal transition in breast cancerous cells^35^ and anti-cholinergic activity^36^ in addition to anti-bacterial activity^37^ and anti-viral activity^38^. 1,2-Benzenedicarboxylic acid has anti-oxidant and anti-microbial potentials^29^. Diisooctyl phthalate has anti-microbial and anti-fouling effects^39^.

## Conclusion

Depending on GC-MS analysis of the Egyptian and Indian Ashwagandha extracts, it is concluded that both extracts contain essential bioactive compounds with vital effects on human health. As the Egyptian Ashwagandha extract is available and contains higher area percentages of most bioactive compounds than found in the Indian Ashwagandha extract, we will test its efficacy in treating some health problems.

## Supplementary Information

Below is the link to the electronic supplementary material.

Supplementary Material 1

Supplementary Material 2

Supplementary Material 3

Supplementary Material 4

## Data Availability

All data generated or analysed during this study are included in this published article and presented as supplementary information files.
