# Supplementary figures and images for: A comparative gas chromatography-mass spectrometry (GC-MS) profiling of Egyptian and Indian ashwagandha (Withania somnifera) root extracts
Source: Sci Rep. 2025 Nov 21;15:41156. doi: 10.1038/s41598-025-25896-3 (PMC12639082; doi:10.1038/s41598-025-25896-3)

**Egyptian ashwagandha compounds**

| 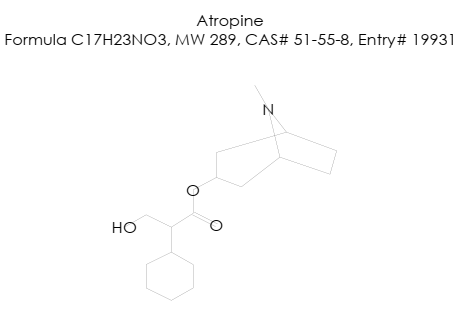 | 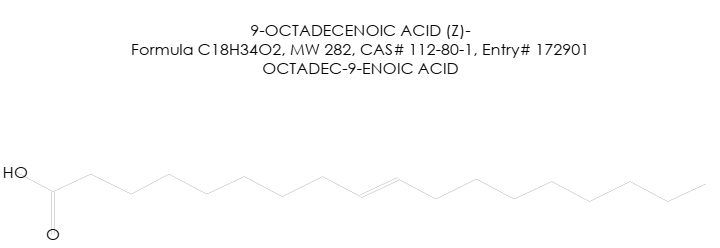 |
| --- | --- |
| 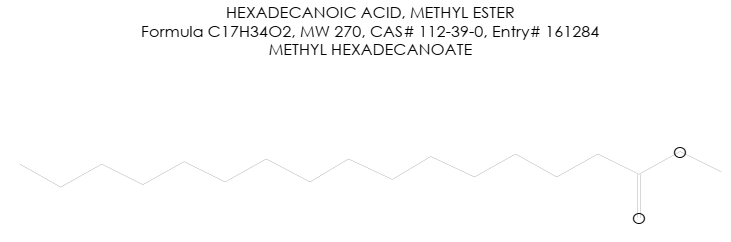 | 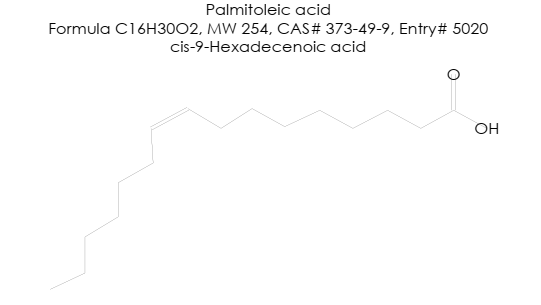 |
| 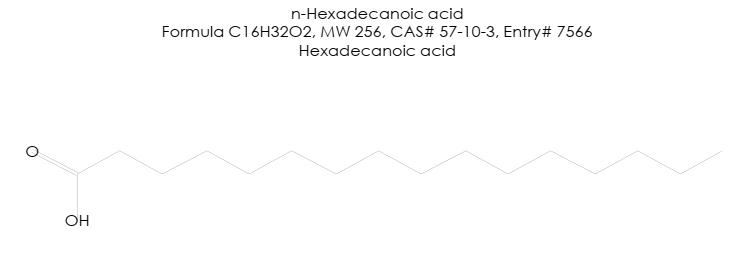 | 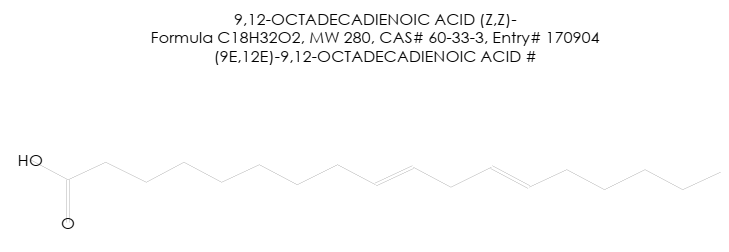 |
| 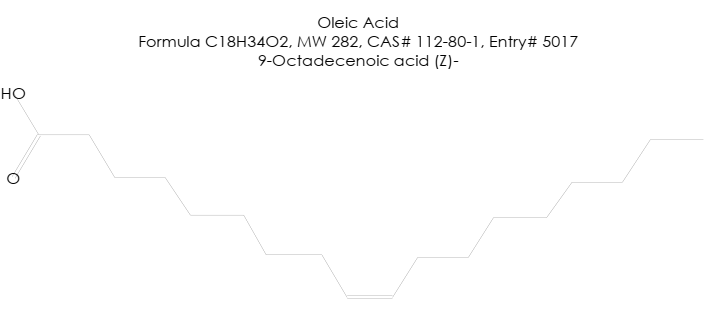 | 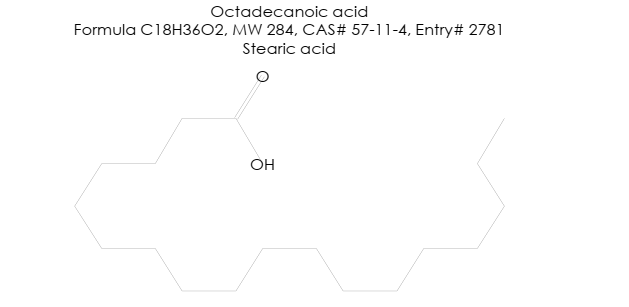 |
| 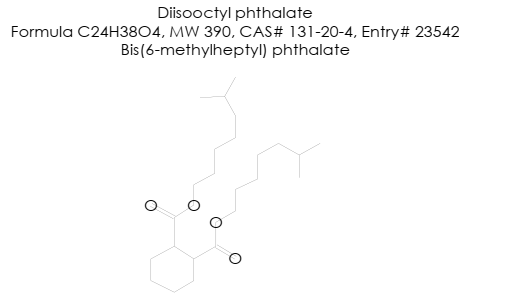 | 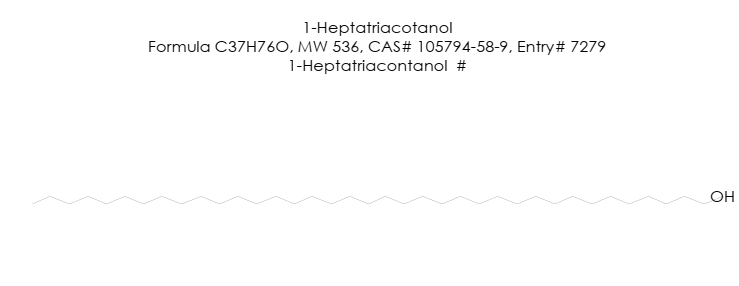 |
| 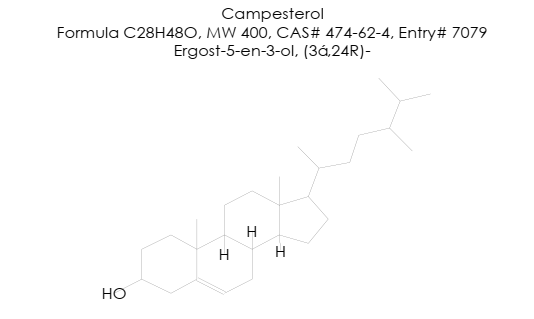 | 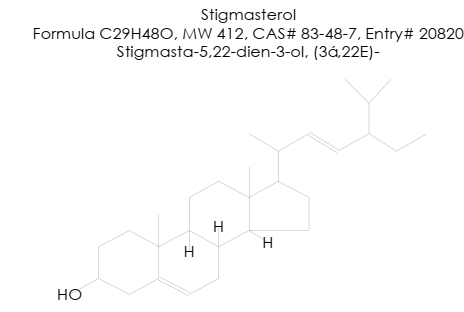 |
| 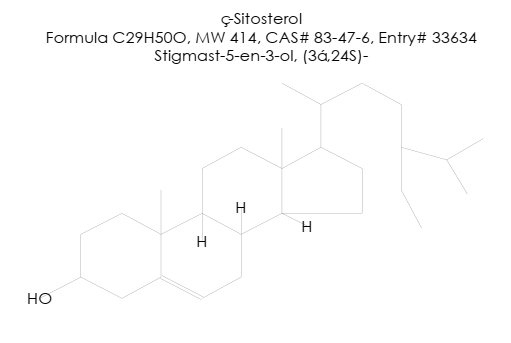 |  |

**Indian ashwagandha compounds**

| 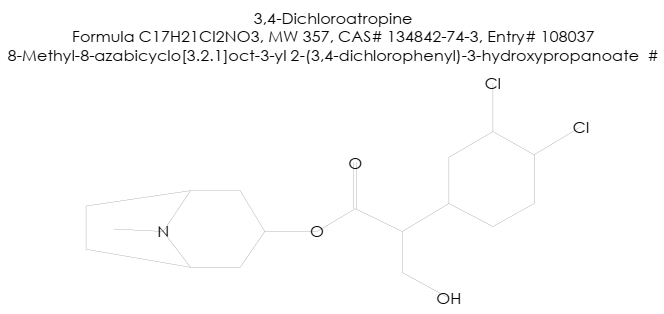 | 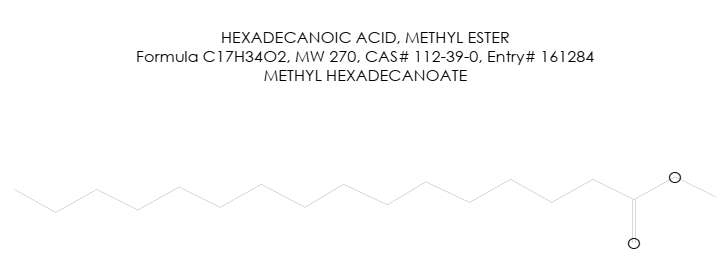 |
| --- | --- |
| 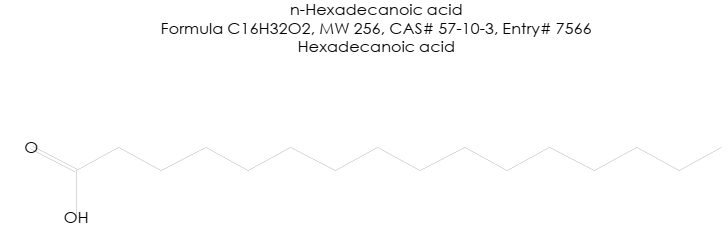 | 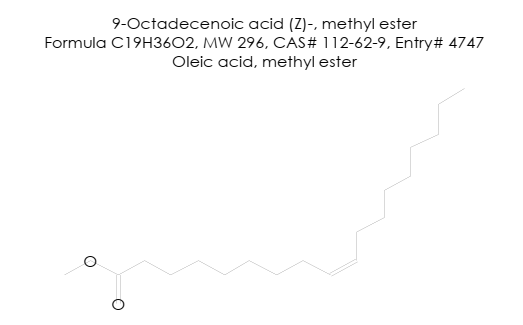 |
| 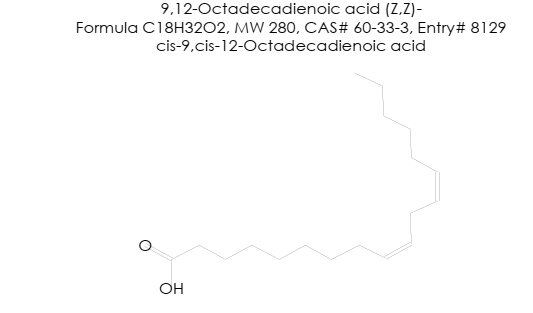 | 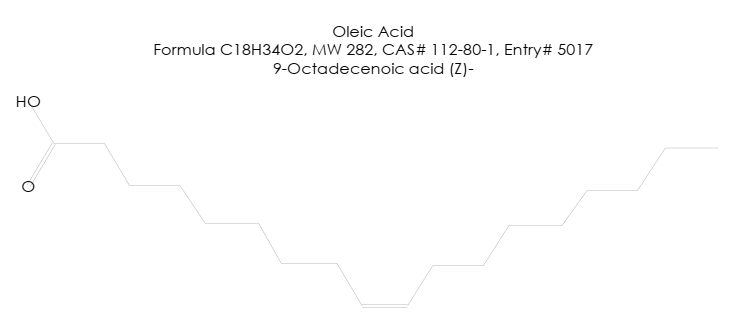 |
| 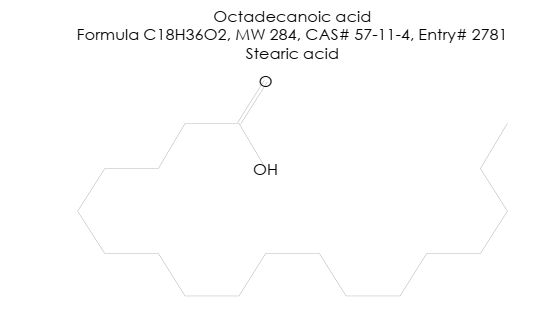 | 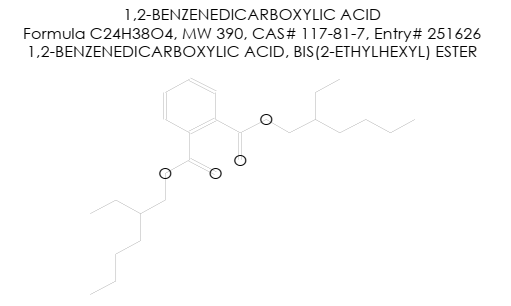 |
| 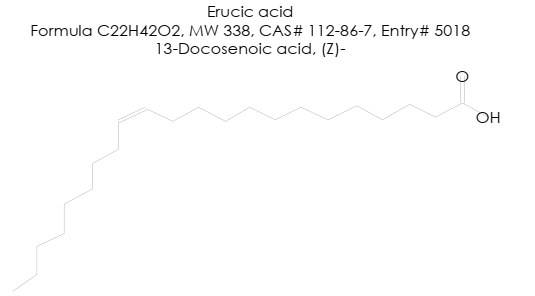 | 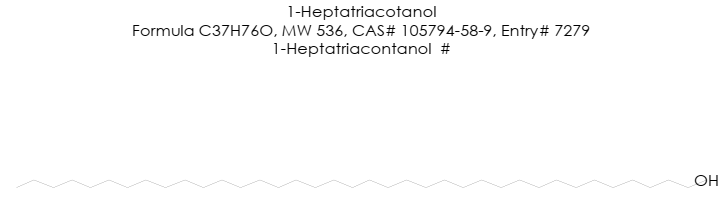 |
| 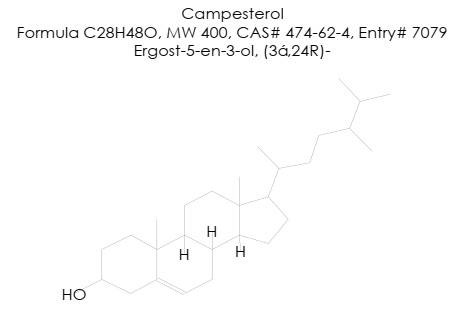 | 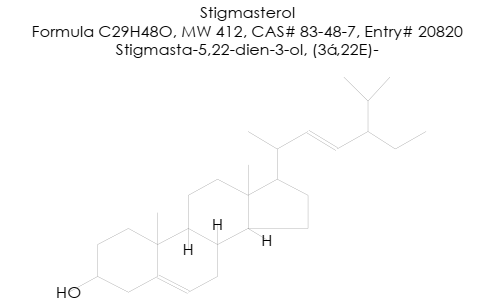 |
| 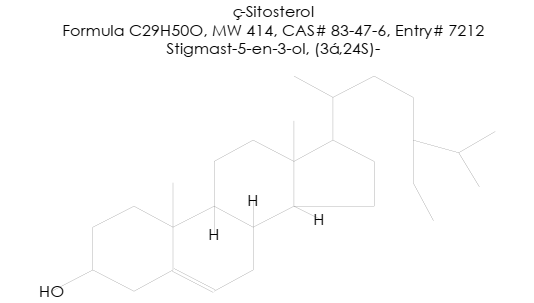 |  |

Supplement: Supplementary file 1 — Supplementary Material 1 [file 41598_2025_25896_MOESM1_ESM.doc]
