# Supplementary material for: A comparative gas chromatography-mass spectrometry (GC-MS) profiling of Egyptian and Indian ashwagandha (Withania somnifera) root extracts
Source: Sci Rep. 2025 Nov 21;15:41156. doi: 10.1038/s41598-025-25896-3 (PMC12639082; doi:10.1038/s41598-025-25896-3)
Supplement: Supplementary file 2 — Supplementary Material 2 [file 41598_2025_25896_MOESM2_ESM.docx]

**Hit spectrum and chemical structure of the Egyptian ashwagandha bioactive compounds**

| 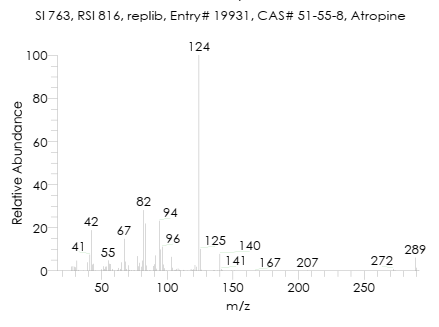 | 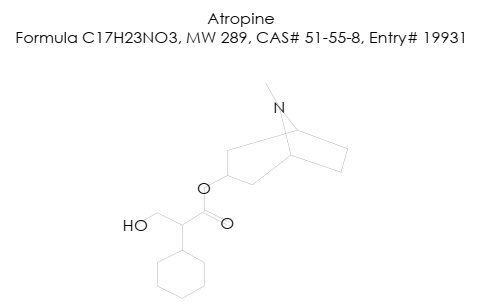 |
| --- | --- |
| 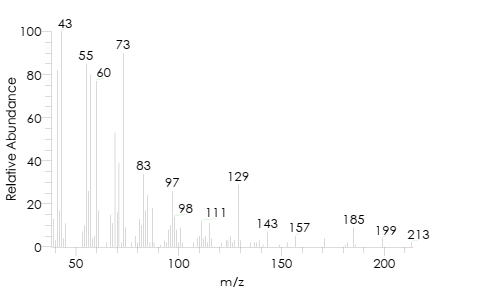 | 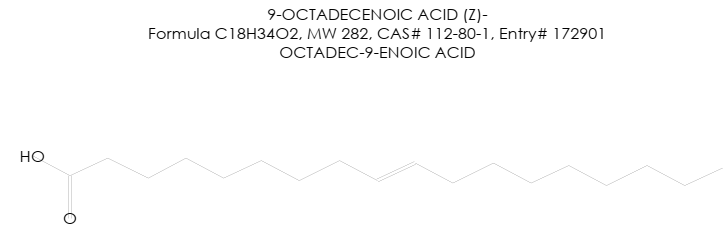 |
| 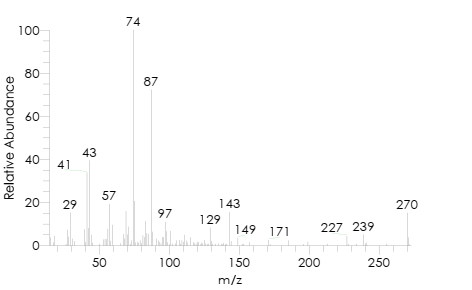 | 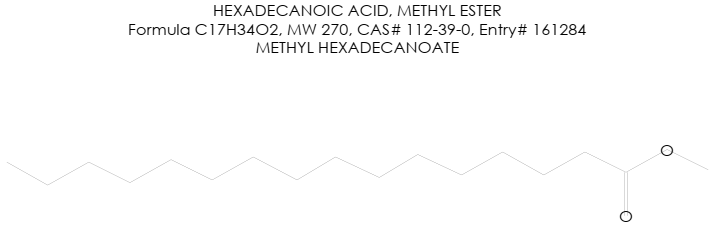 |
| 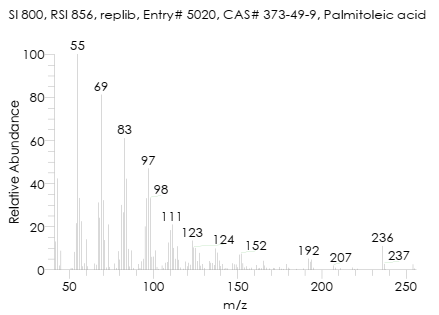 | 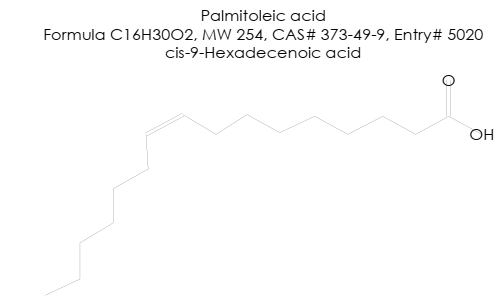 |
| 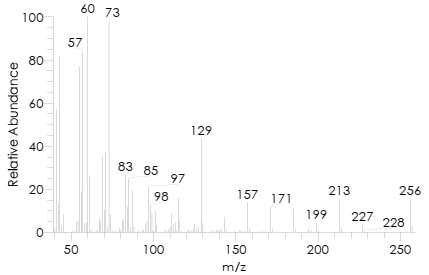 | 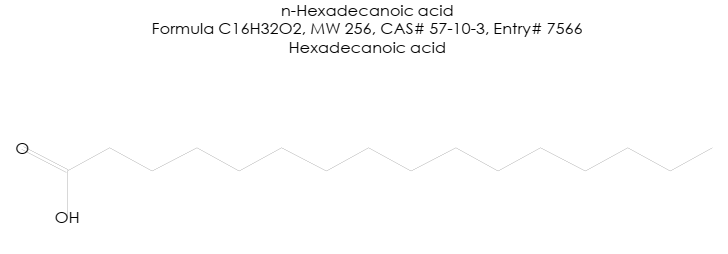 |
| 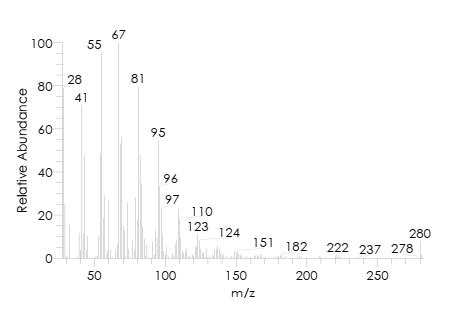 | 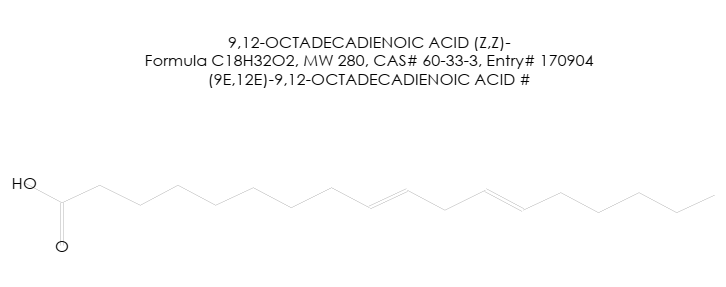 |
| 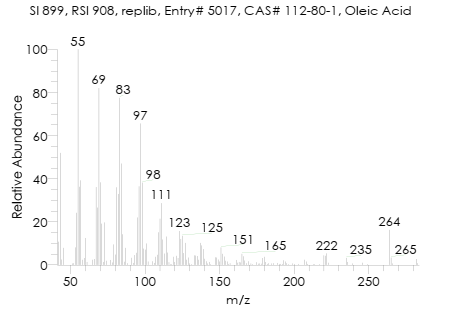 | 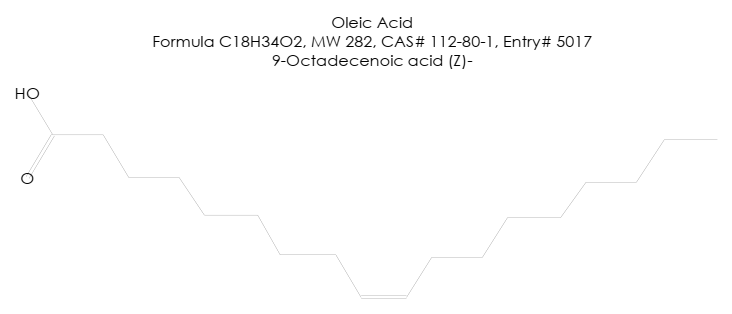 |
| 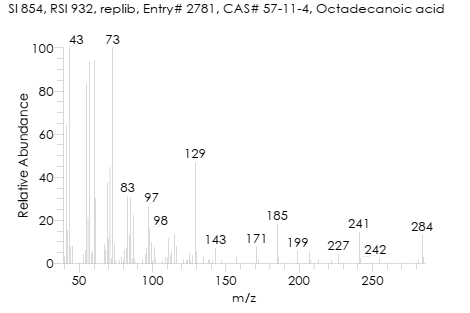 | 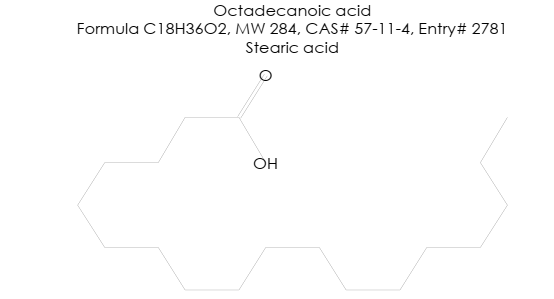 |
| 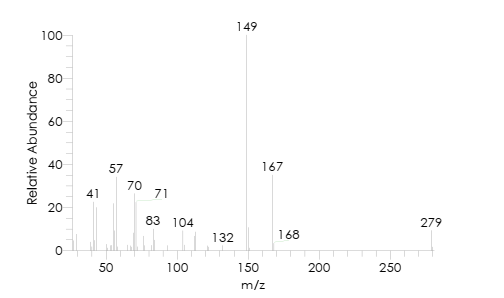 | 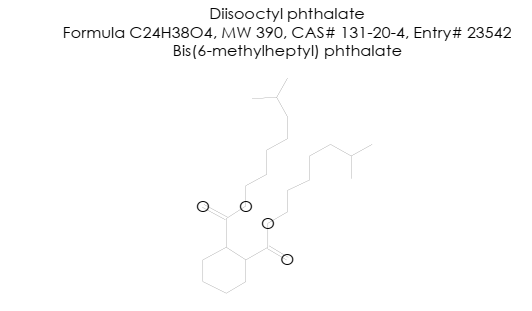 |
| 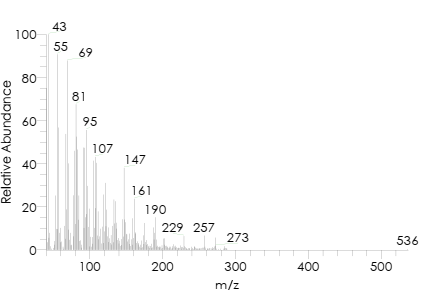 | 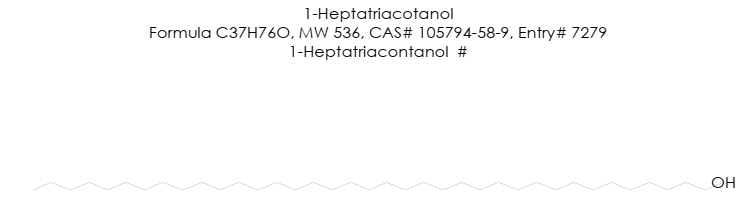 |
| 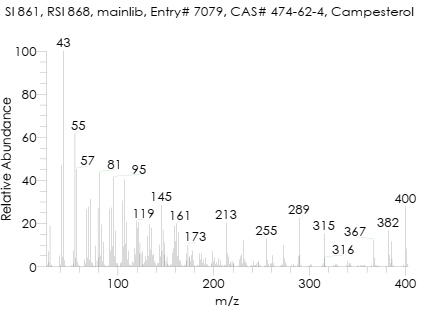 | 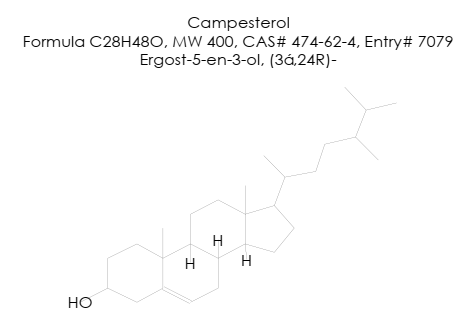 |
| 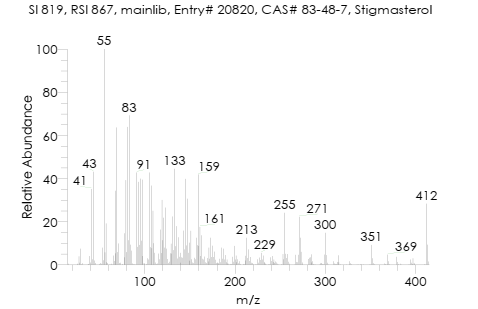 | 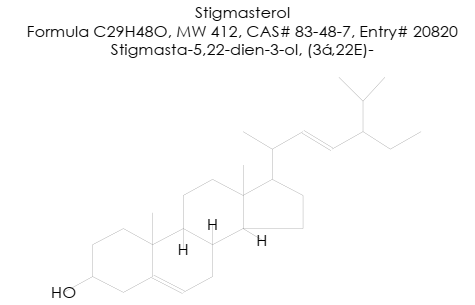 |
| 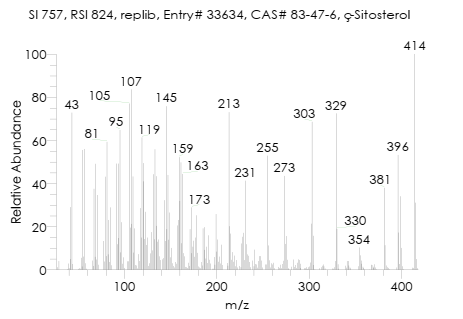 | 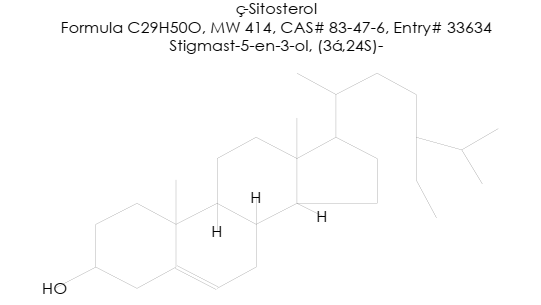 |

**Hit spectrum and chemical structures of the Indian ashwagandha bioactive compounds**

| 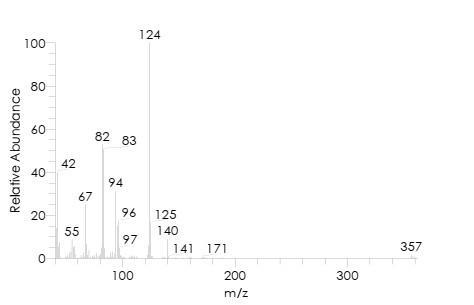 | 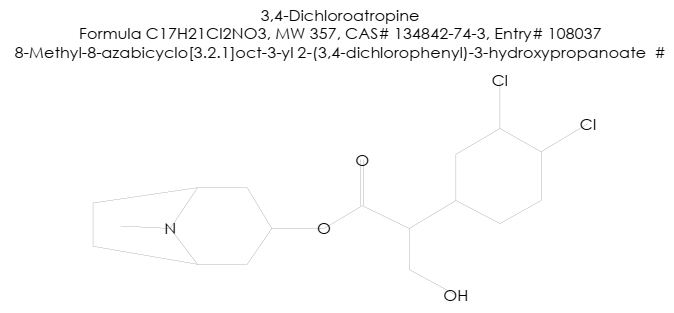 |
| --- | --- |
| 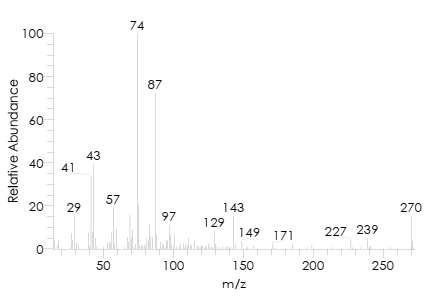 | 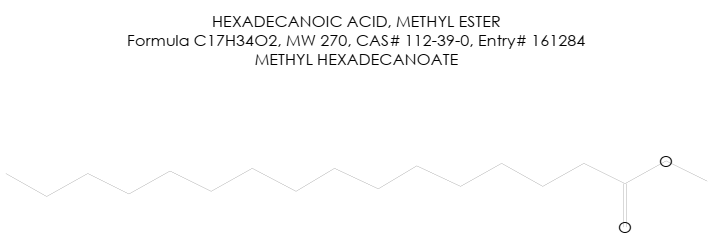 |
| 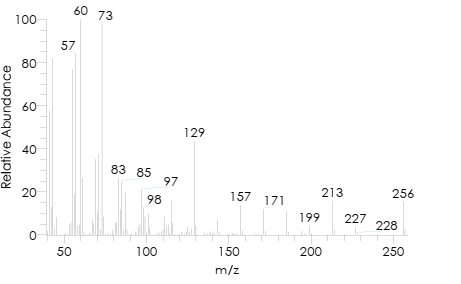 | 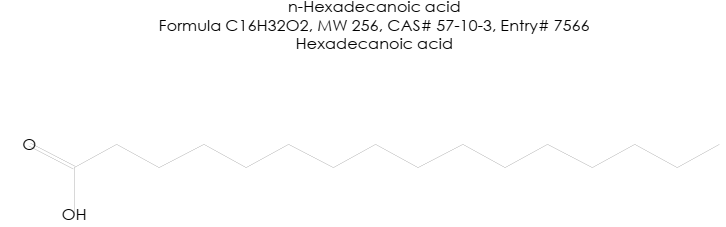 |
| 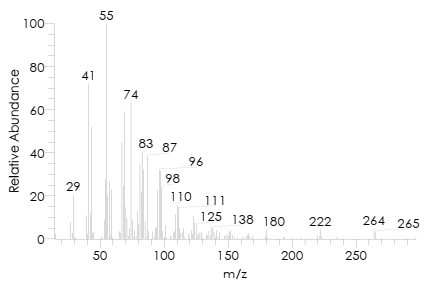 | 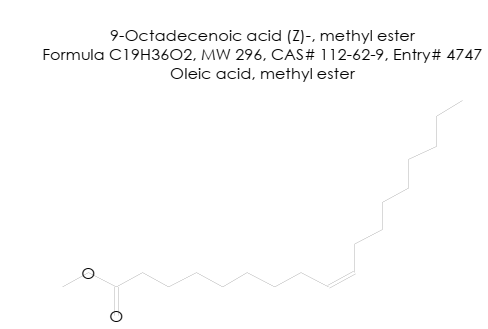 |
| 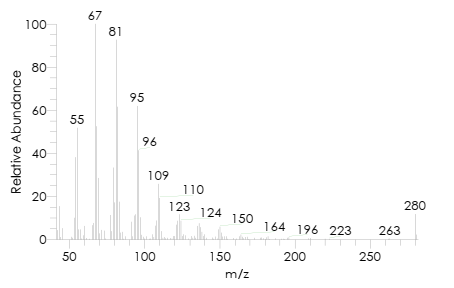 | 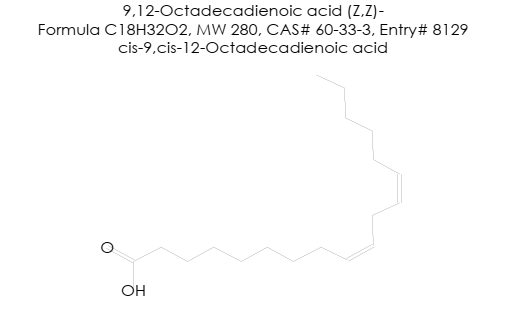 |
| 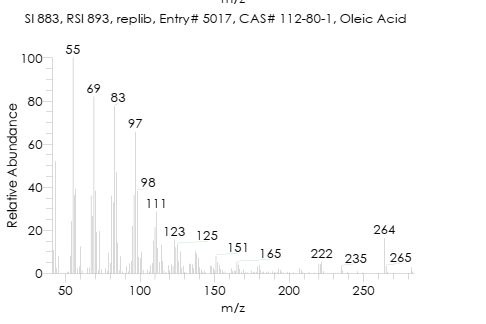 | 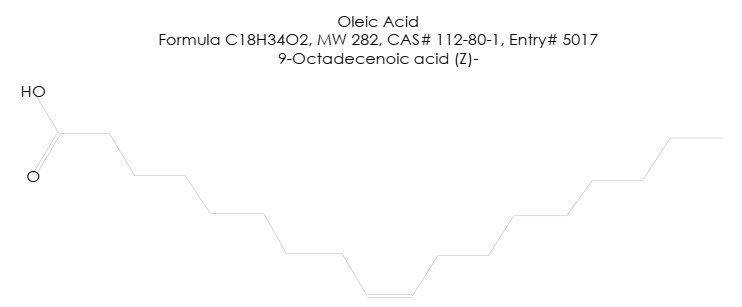 |
| 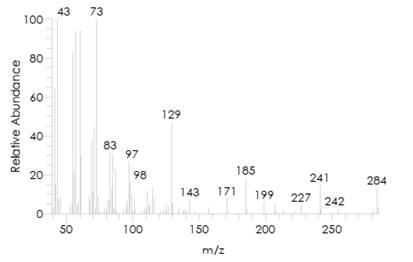 | 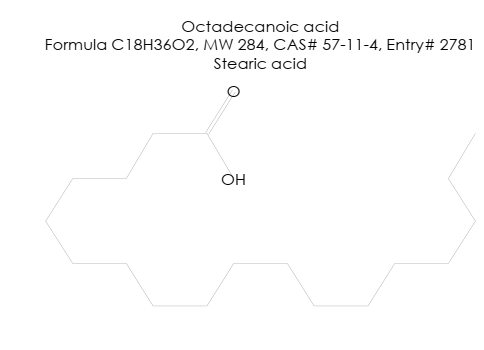 |
| 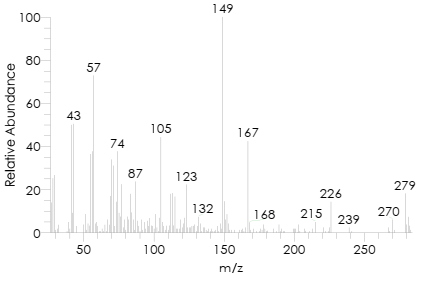 | 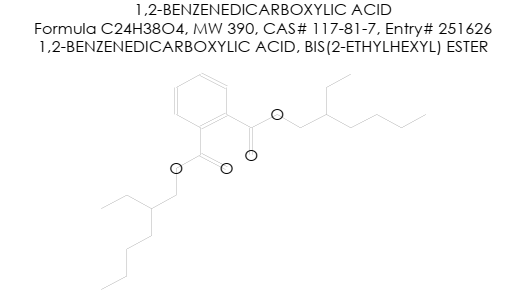 |
| 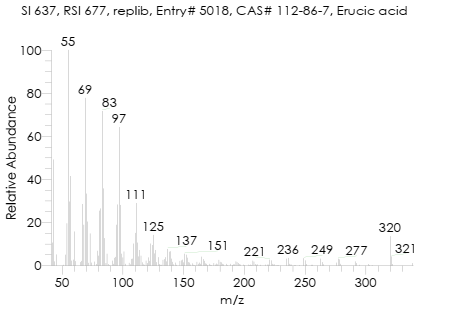 | 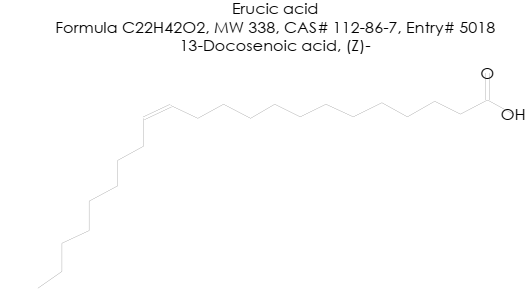 |
| 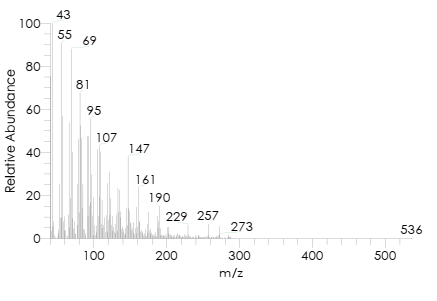 | 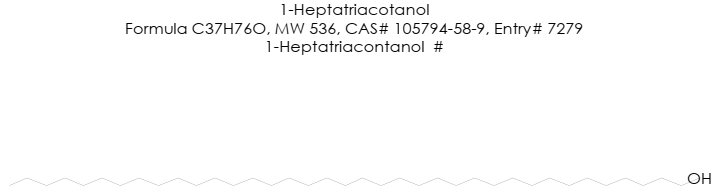 |
| 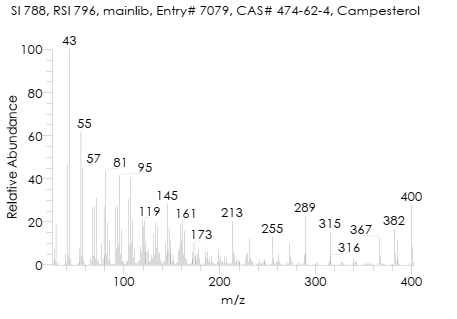 | 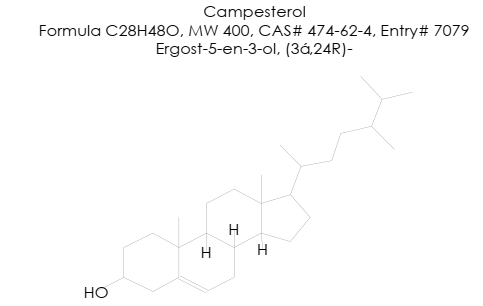 |
| 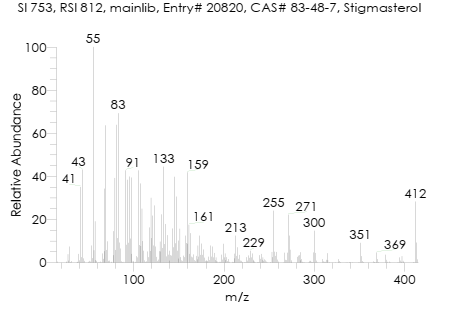 | 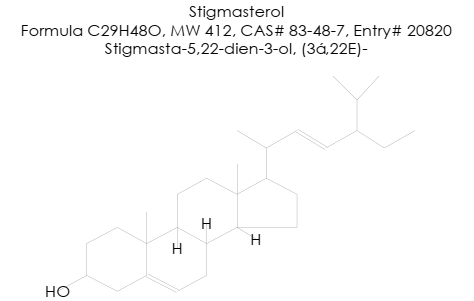 |
| 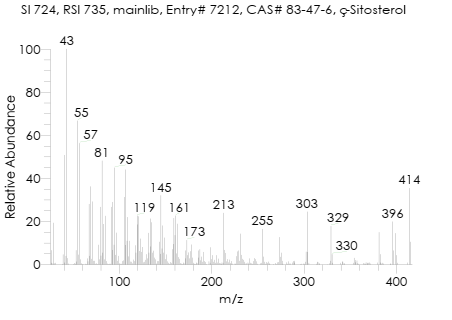 | 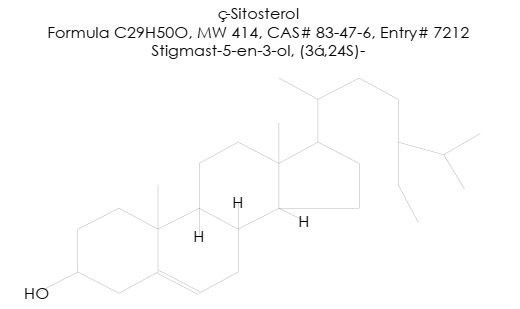 |
