# Supplementary material for: A comparative gas chromatography-mass spectrometry (GC-MS) profiling of Egyptian and Indian ashwagandha (Withania somnifera) root extracts
Source: Sci Rep. 2025 Nov 21;15:41156. doi: 10.1038/s41598-025-25896-3 (PMC12639082; doi:10.1038/s41598-025-25896-3)

My GC-MS Report

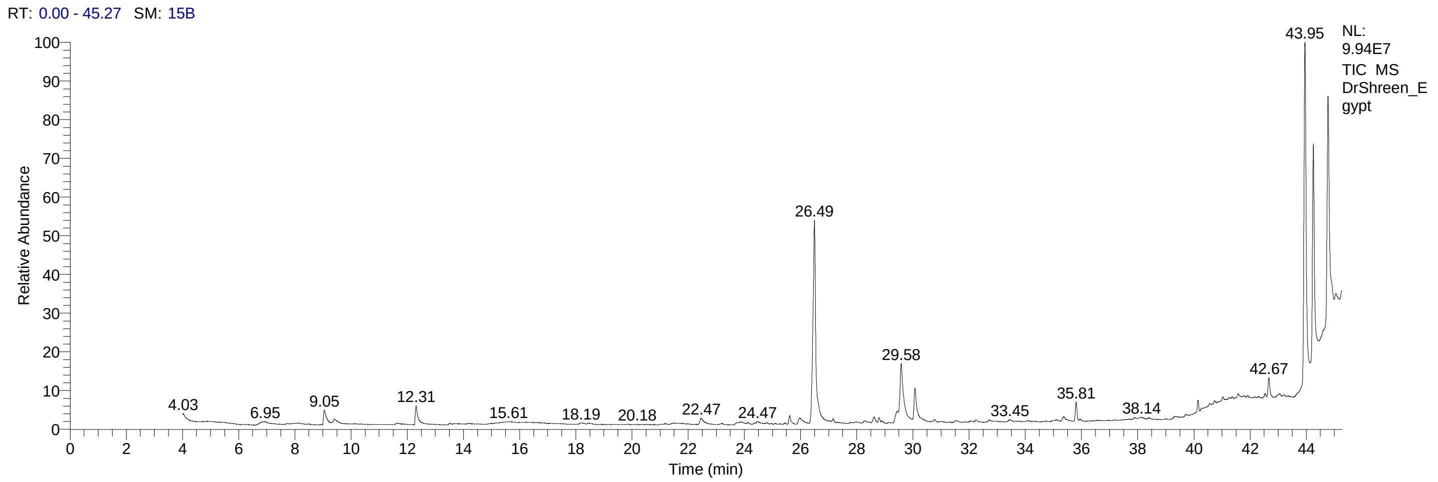

RT	Area %	Peak Area	Peak Height
9.04	1.21	16666143.77	3528399.86
9.41	0.38	5266524.74	893207.45
12.31	1.49	20563121.63	4859536.72
22.46	0.66	9088491.17	1416481.39
25.61	0.65	8944701.84	2414779.74
25.97	0.88	12123489.86	1392113.24
26.50	17.43	239866436.57	48737917.98
27.16	0.24	3261925.56	966801.53
28.62	0.57	7810453.01	1565907.74
28.80	0.37	5154504.83	1478512.78
29.43	0.47	6443781.01	1177765.17
29.58	4.66	64127190.14	12259700.57
30.07	2.59	35698557.72	7933328.61
35.36	0.52	7217637.29	1105373.66
35.81	1.38	19004278.42	5381658.46
40.15	0.74	10219235.71	3763638.91
41.03	0.31	4241897.83	1210513.72
41.58	0.37	5109404.68	1082040.55
42.53	0.31	4206290.26	1189976.85
42.67	1.64	22549885.82	5571138.15
43.95	28.70	395074267.47	88640878.24
44.25	16.11	221775763.78	58145197.75
44.77	17.66	243048698.63	57022274.01
45.05	0.66	9057361.07	1783013.62

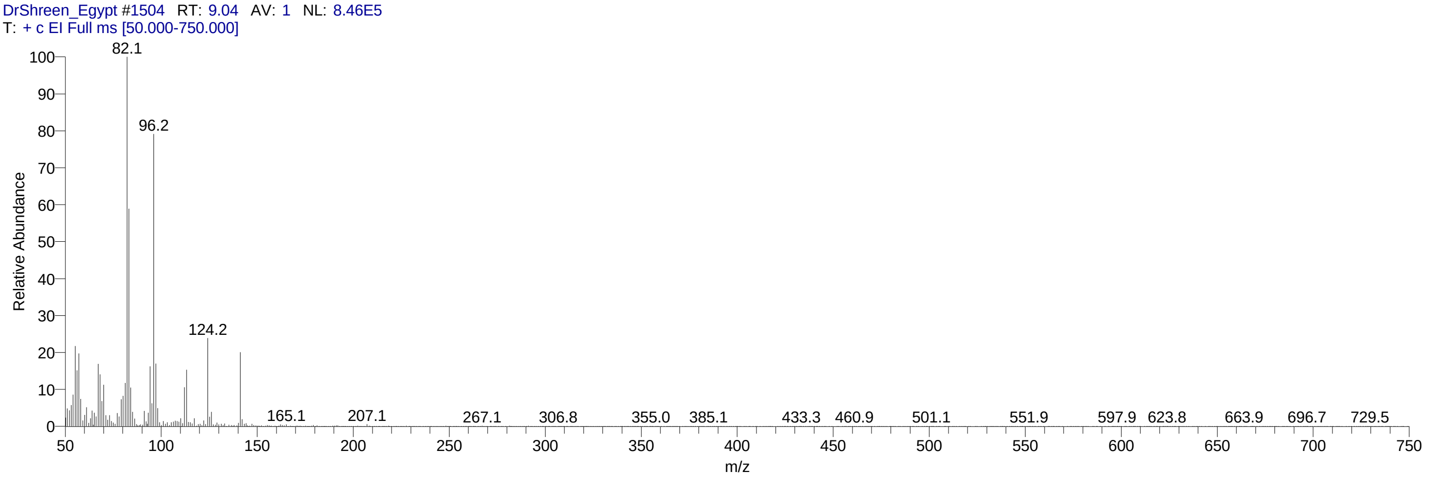

My GC-MS Report

RT	Compound Name	Area %	MF	Molecular Formula	Molecular Weight	Cas #	Library
9.04	8-Azabicyclo[3.2.1]octan-3-ol, 8-methyl-, endo-	1.21	909	C8H15NO	141	120-29-6	replib
9.04	8-AZABICYCLO[3.2.1]OCTAN-3-OL, 8-METHYL-, ENDO-	1.21	909	C8H15NO	141	120-29-6	WileyRegistry8e
9.04	8-Azabicyclo[3.2.1]octan-3-ol, 8-methyl-, endo-	1.21	910	C8H15NO	141	120-29-6	mainlib
9.04	8-METHYL-8-AZABICYCLO[3.2.1]OCTAN-3-OL	1.21	910	C8H15NO	141	120-29-6	WileyRegistry8e
9.04	8-METHYL-8-AZABICYCLO[3.2.1]OCTAN-3-OL	1.21	910	C8H15NO	141	NA	WileyRegistry8e

Compound Structure | Hit Spectrum

8-Azabicyclo[3.2.1]octan-3-ol, 8-methyl-, endo-
Formula C8H15NO, MW 141, CAS# 120-29-6, Entry# 11803
Tropine

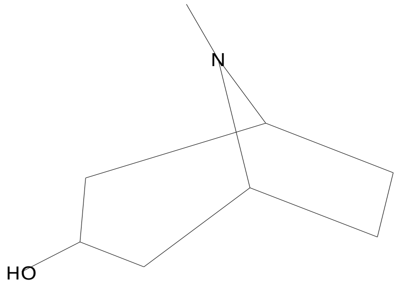

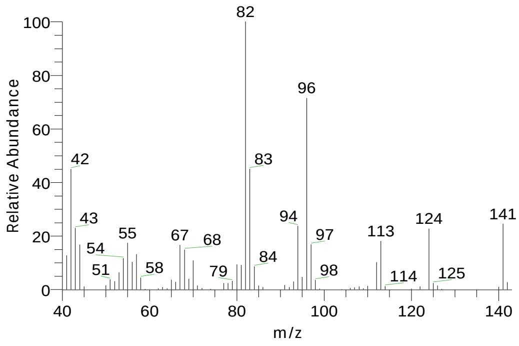

8-AZABICYCLO[3.2.1]OCTAN-3-OL, 8-METHYL-, ENDO-
Formula C8H15NO, MW 141, CAS# 120-29-6, Entry# 394844
8-METHYL-8-AZABICYCLO[3.2.1]OCTAN-3-OL

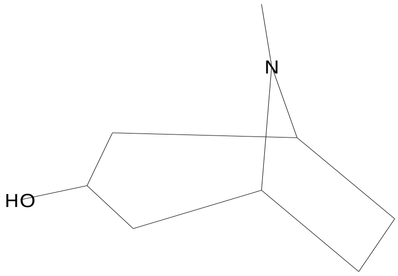

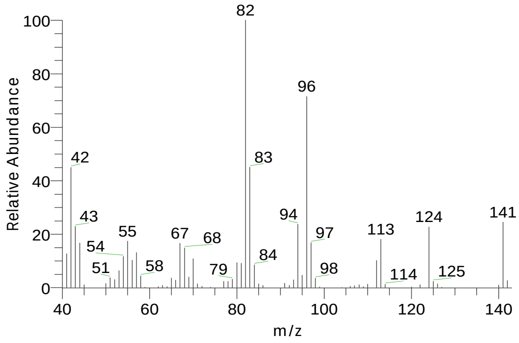

8-Azabicyclo[3.2.1]octan-3-ol, 8-methyl-, endo-
Formula C8H15NO, MW 141, CAS# 120-29-6, Entry# 51507
Tropine

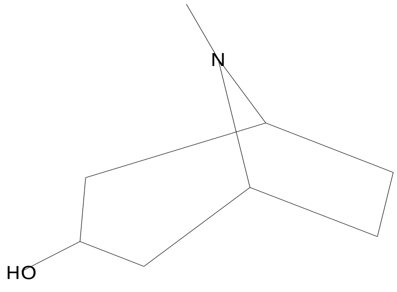

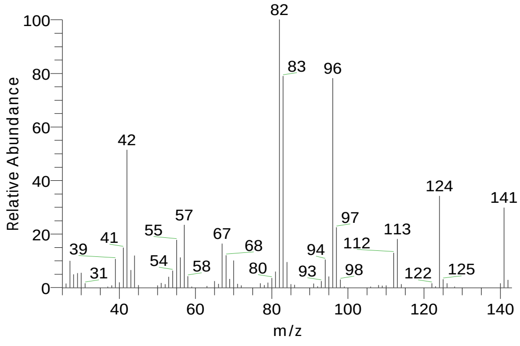

My GC-MS Report

Compound Structure | Hit Spectrum

8-METHYL-8-AZABICYCLO[3.2.1]OCTAN-3-OL
Formula C8H15NO, MW 141, CAS# 120-29-6, Entry# 359696
8-METHYL-8-AZA-BICYCLO[3.2.1]OCTAN-3-OL

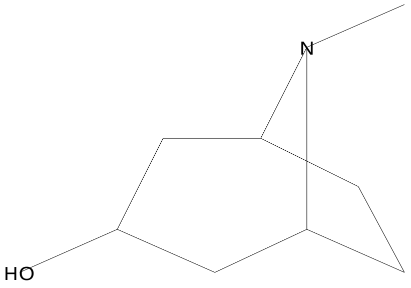

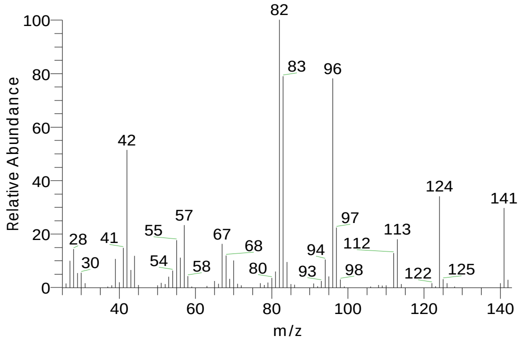

8-METHYL-8-AZABICYCLO[3.2.1]OCTAN-3-OL
Formula C8H15NO, MW 141, CAS# NA, Entry# 397029
8-METHYL-8-AZA-BICYCLO[3.2.1]OCTAN-3-OL

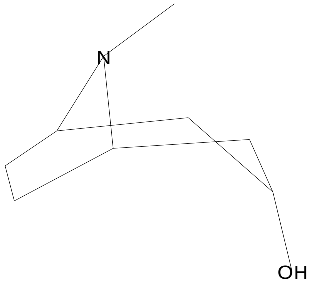

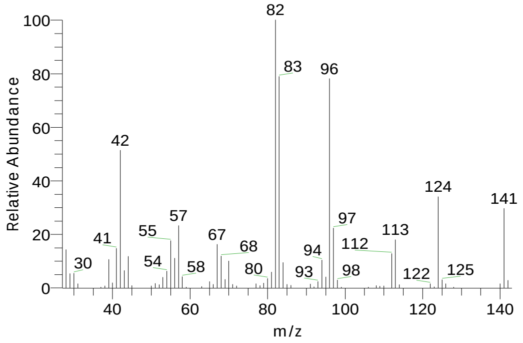

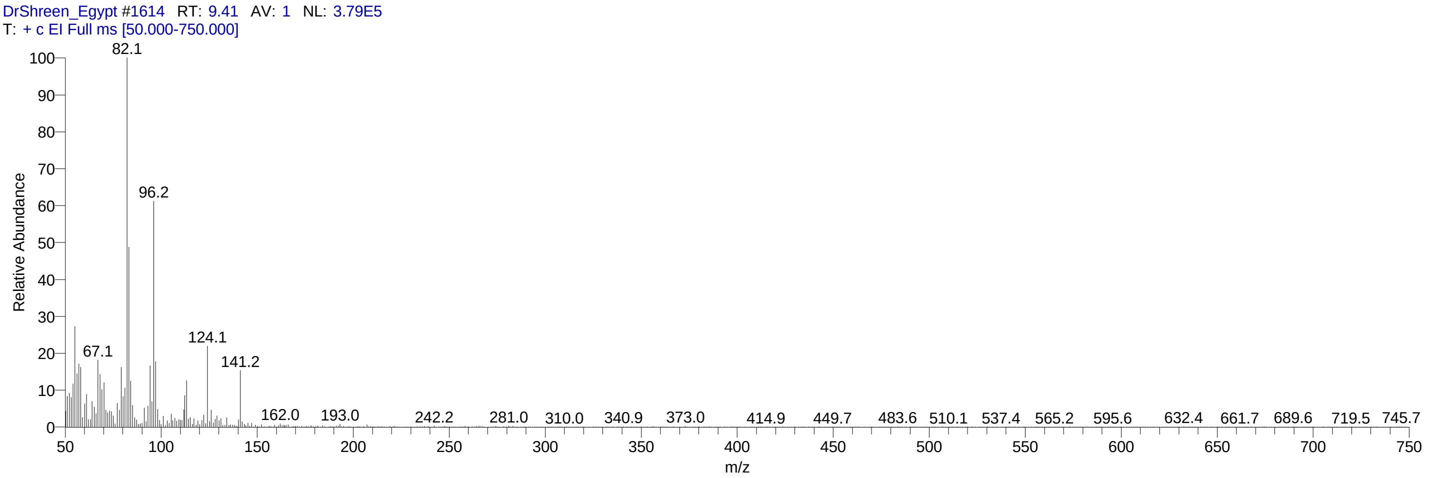

RT	Compound Name	Area %	MF	Molecular Formula	Molecular Weight	Cas #	Library
9.41	8-Azabicyclo[3.2.1]octan-3-ol, 8-methyl-, endo-	0.38	844	C8H15NO	141	120-29-6	replib
9.41	8-AZABICYCLO[3.2.1]OCTAN-3-OL, 8-METHYL-, ENDO-	0.38	844	C8H15NO	141	120-29-6	WileyRegistry8e
9.41	8-METHYL-8-AZABICYCLO[3.2.1]OCTAN-3-OL	0.38	846	C8H15NO	141	NA	WileyRegistry8e
9.41	8-METHYL-8-AZABICYCLO[3.2.1]OCTAN-3-OL	0.38	845	C8H15NO	141	120-29-6	WileyRegistry8e
9.41	8-Azabicyclo[3.2.1]octan-3-ol, 8-methyl-, endo-	0.38	844	C8H15NO	141	120-29-6	mainlib

My GC-MS Report

Compound Structure | Hit Spectrum

8-Azabicyclo[3.2.1]octan-3-ol, 8-methyl-, endo-
Formula C8H15NO, MW 141, CAS# 120-29-6, Entry# 11803
Tropine

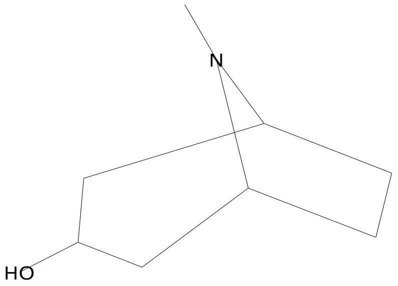

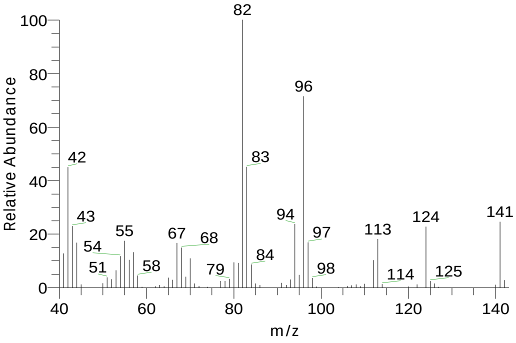

8-AZABICYCLO[3.2.1]OCTAN-3-OL, 8-METHYL-, ENDO-
Formula C8H15NO, MW 141, CAS# 120-29-6, Entry# 394844
8-METHYL-8-AZABICYCLO[3.2.1]OCTAN-3-OL

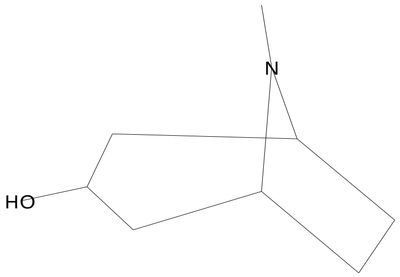

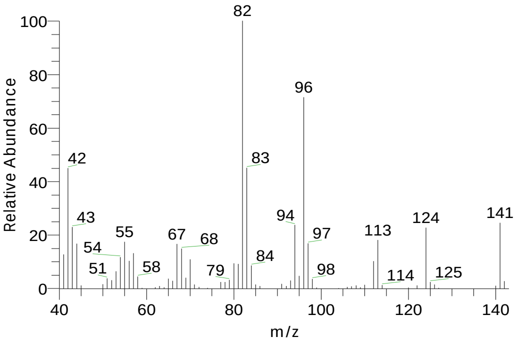

8-METHYL-8-AZABICYCLO[3.2.1]OCTAN-3-OL
Formula C8H15NO, MW 141, CAS# NA, Entry# 397029
8-METHYL-8-AZA-BICYCLO[3.2.1]OCTAN-3-OL

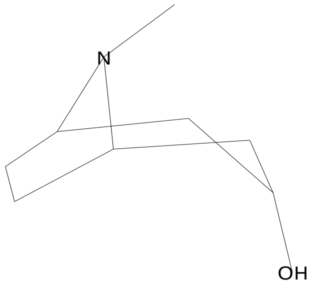

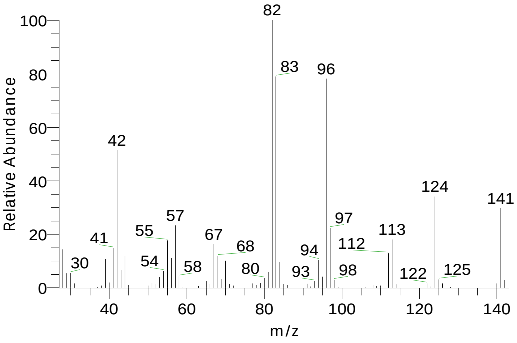

8-METHYL-8-AZABICYCLO[3.2.1]OCTAN-3-OL
Formula C8H15NO, MW 141, CAS# 120-29-6, Entry# 359696
8-METHYL-8-AZA-BICYCLO[3.2.1]OCTAN-3-OL

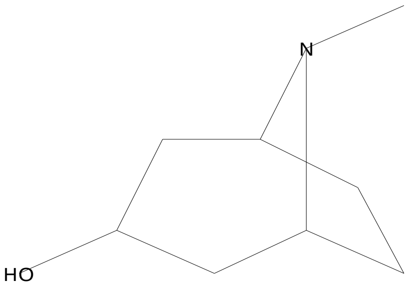

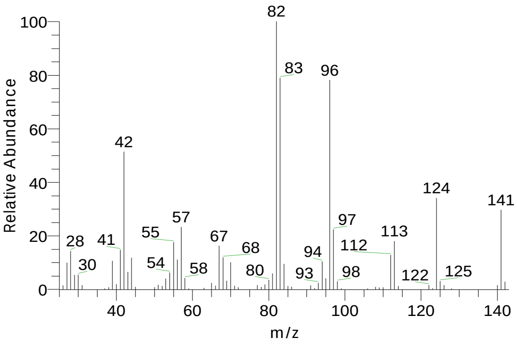

My GC-MS Report

Compound Structure | Hit Spectrum

8-Azabicyclo[3.2.1]octan-3-ol, 8-methyl-, endo-
Formula C8H15NO, MW 141, CAS# 120-29-6, Entry# 51507
Tropine

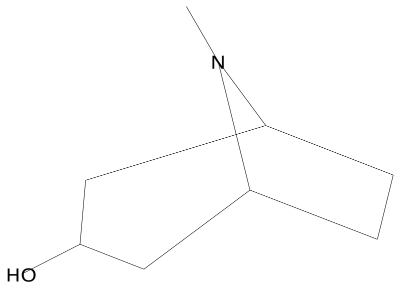

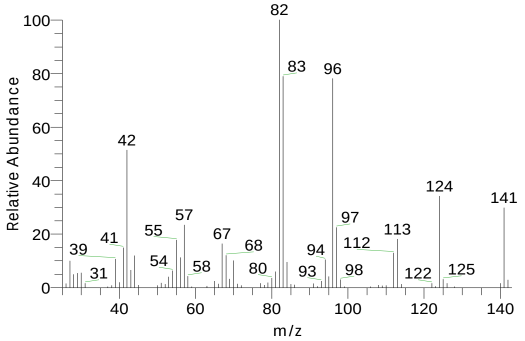

DrShreen_Egypt #2478 RT: 12.31 AV: 1 NL: 1.35E6
T: + c EI Full ms [50.000-750.000]

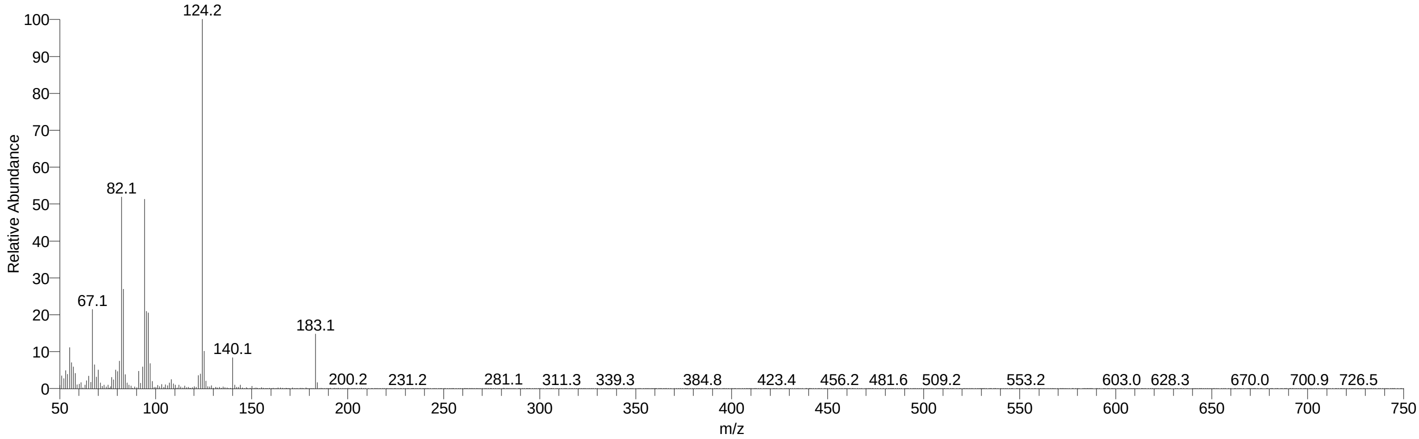

RT	Compound Name	Area %	MF	Molecular Formula	Molecular Weight	Cas #	Library
12.31	8-Azabicyclo[3.2.1]octan-3-ol,8-methyl-acetate(ester),exo-	1.49	946	C10H17NO2	183	3423-26-5	mainlib
12.31	8-Azabicyclo[3.2.1]octan-3-ol, 8-methyl-, acetate (ester), endo-	1.49	908	C10H17NO2	183	3423-27-6	mainlib
12.31	3,4-Dichloroatropine	1.49	815	C17H21Cl2NO3	357	134842-74-3	mainlib
12.31	O-Bromoatropine	1.49	783	C17H22BrNO3	367	51491-66-8	mainlib
12.31	Atropine	1.49	816	C17H23NO3	289	51-55-8	replib

Compound Structure | Hit Spectrum

8-Azabicyclo[3.2.1]octan-3-ol,8-methyl-acetate(ester),exo-
Formula C10H17NO2, MW 183, CAS# 3423-26-5, Entry# 108048
$:28MDIDMOWWLBGYPG-UHFFFAOYSA-N

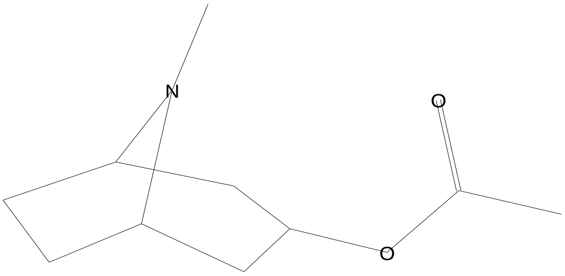

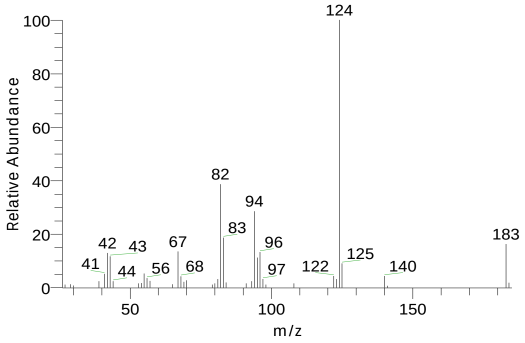

My GC-MS Report

Compound Structure | Hit Spectrum

8-Azabicyclo[3.2.1]octan-3-ol, 8-methyl-, acetate (ester), endo-
Formula C10H17NO2, MW 183, CAS# 3423-27-6, Entry# 108049
$:28MDIDMOWWLBGYPG-UHFFFAOYSA-N

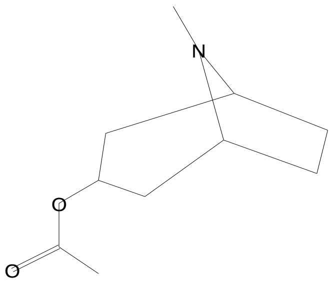

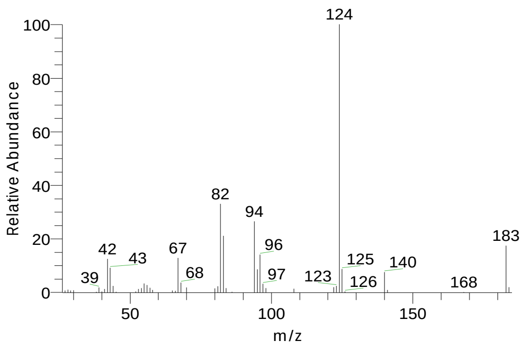

3,4-Dichloroatropine
Formula C17H21Cl2NO3, MW 357, CAS# 134842-74-3, Entry# 108037
8-Methyl-8-azabicyclo[3.2.1]oct-3-yl 2-(3,4-dichlorophenyl)-3-hydroxypropanoate #

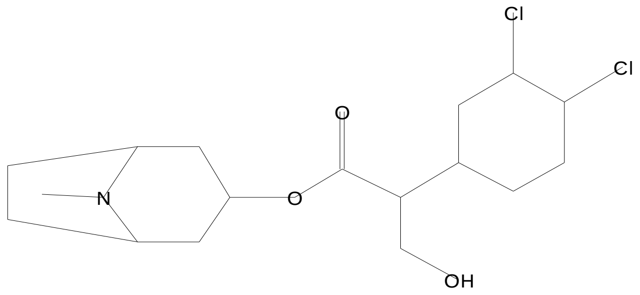

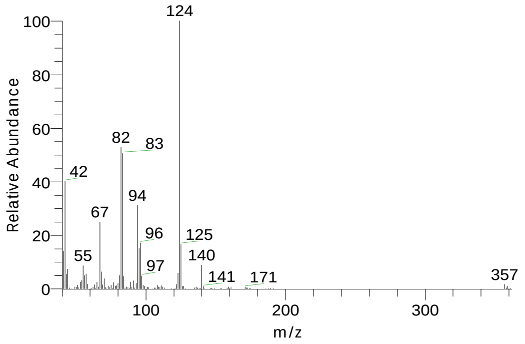

O-Bromoatropine
Formula C17H22BrNO3, MW 367, CAS# 51491-66-8, Entry# 108035
8-Methyl-8-azabicyclo[3.2.1]oct-3-yl 2-(2-bromophenyl)-3-hydroxypropanoate #

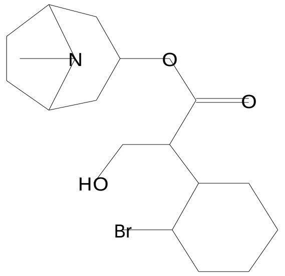

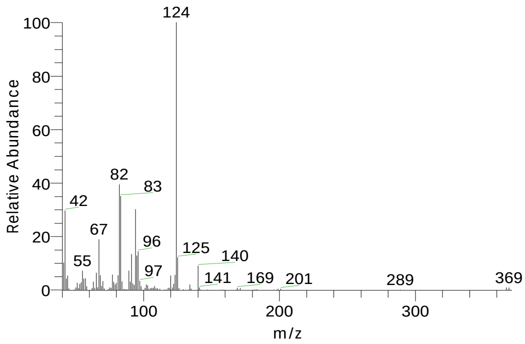

Atropine
Formula C17H23NO3, MW 289, CAS# 51-55-8, Entry# 19931

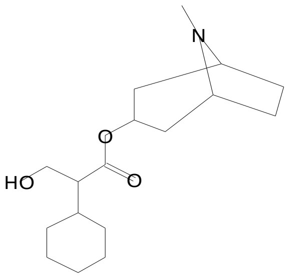

SI 763, RSI 816, replib, Entry# 19931, CAS# 51-55-8, Atropine

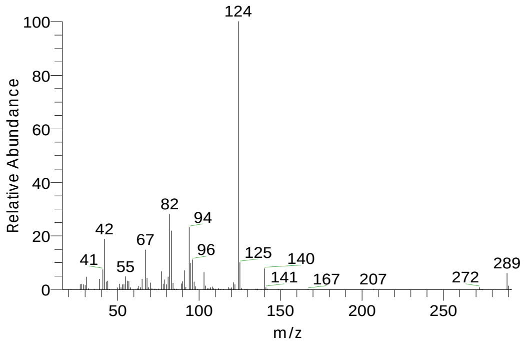

My GC-MS Report

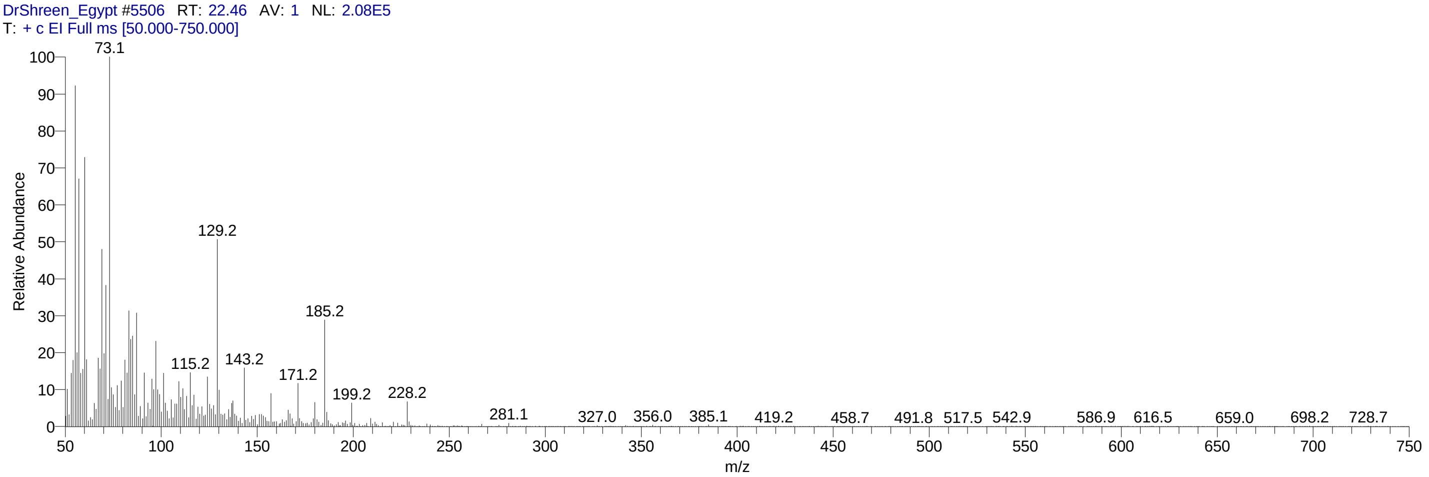

RT	Compound Name	Area %	MF	Molecular Formula	Molecular Weight	Cas #	Library
22.46	TETRADECANOIC ACID	0.66	809	C14H28O2	228	544-63-8	WileyRegistry8e
22.46	TETRADECANOIC ACID	0.66	773	C14H28O2	228	544-63-8	WileyRegistry8e
22.46	PENTADECANOIC ACID	0.66	769	C15H30O2	242	1002-84-2	WileyRegistry8e
22.46	9-OCTADECENOIC ACID (Z)-	0.66	875	C18H34O2	282	112-80-1	WileyRegistry8e
22.46	2-AMINOETHANETHIOL HYDROGEN SULFATE (ESTER)	0.66	834	C2H7NO3S2	157	2937-53-3	WileyRegistry8e

Compound Structure	Hit Spectrum

TETRADECANOIC ACID
Formula C14H28O2, MW 228, CAS# 544-63-8, Entry# 116584
METHYL TRIDECANOATE

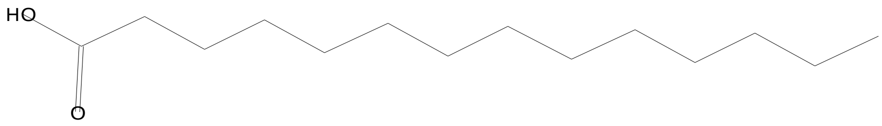

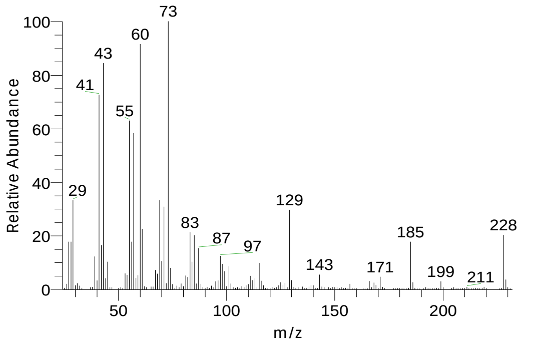

TETRADECANOIC ACID
Formula C14H28O2, MW 228, CAS# 544-63-8, Entry# 116588
METHYL TRIDECANOATE

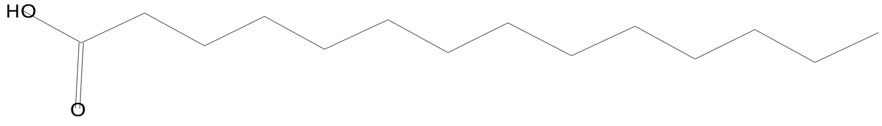

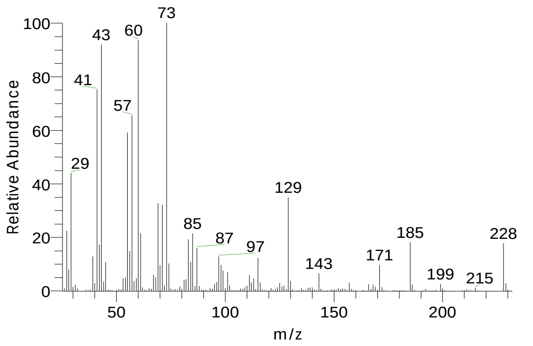

My GC-MS Report

Compound Structure | Hit Spectrum

PENTADECANOIC ACID
Formula C15H30O2, MW 242, CAS# 1002-84-2, Entry# 131990
14FA

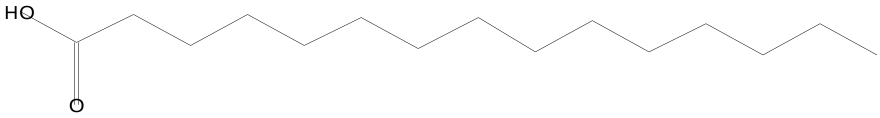

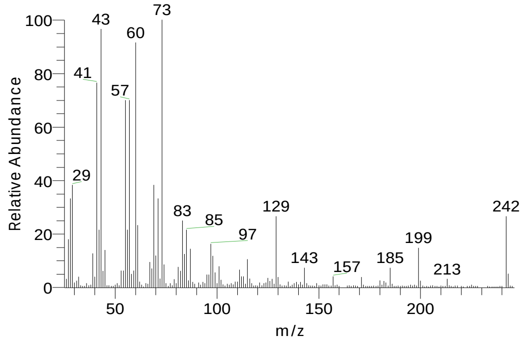

9-OCTADECENOIC ACID (Z)-
Formula C18H34O2, MW 282, CAS# 112-80-1, Entry# 172901
OCTADEC-9-ENOIC ACID

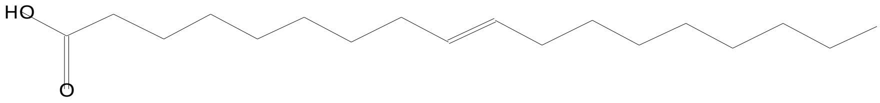

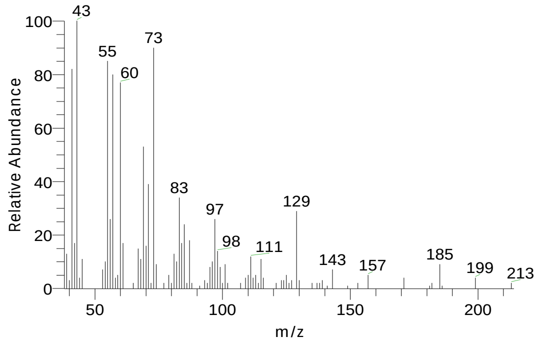

2-AMINOETHANETHIOL HYDROGEN SULFATE (ESTER)
Formula C2H7NO3S2, MW 157, CAS# 2937-53-3, Entry# 41029
2-AMINOETHANETHIOLSULFURIC ACID

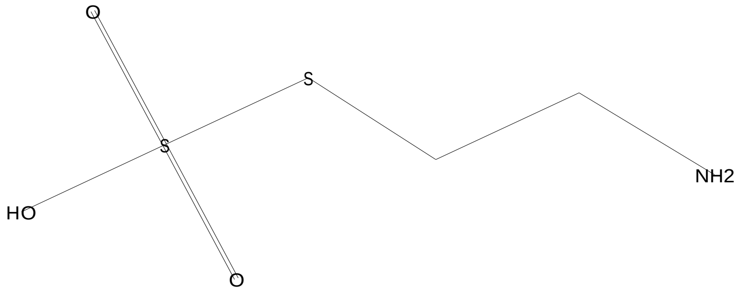

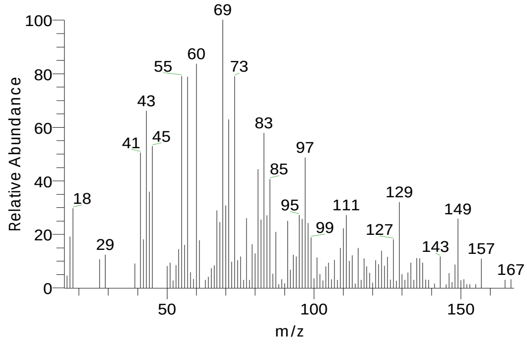

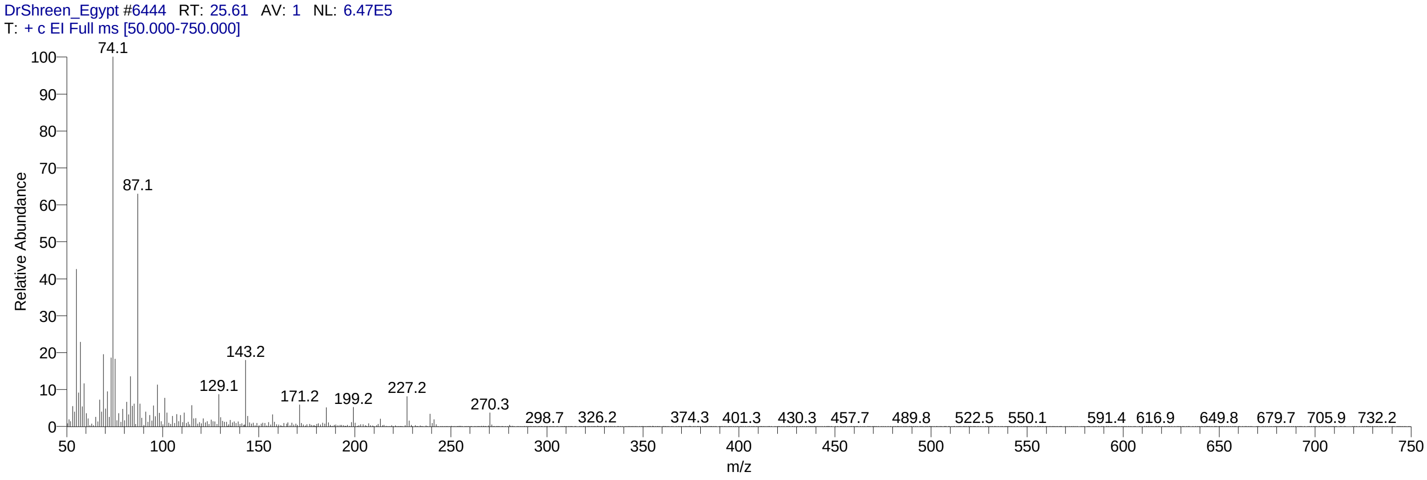

RT	Compound Name	Area %	MF	Molecular Formula	Molecular Weight	Cas #	Library
25.61	PENTADECANOIC ACID, 14-METHYL-, METHYL ESTER	0.65	819	C17H34O2	270	5129-60-2	WileyRegistry8e
25.61	HEXADECANOIC ACID, METHYL ESTER	0.65	831	C17H34O2	270	112-39-0	WileyRegistry8e

My GC-MS Report

RT	Compound Name	Area %	MF	Molecular Formula	Molecular Weight	Cas #	Library
25.61	PENTADECANOIC ACID, 14-METHYL-, METHYL ESTER	0.65	840	C17H34O2	270	5129-60-2	WileyRegistry8e
25.61	Palmitic Acid methyl ester	0.65	807	C17H34O2	270	112-39-0	CaymanSpectralLibrary-NIST.HP
25.61	HEXADECANOIC ACID, METHYL ESTER	0.65	860	C17H34O2	270	112-39-0	WileyRegistry8e

Compound Structure | Hit Spectrum

PENTADECANOIC ACID, 14-METHYL-, METHYL ESTER
Formula C17H34O2, MW 270, CAS# 5129-60-2, Entry# 161312
METHYL 14-METHYLPENTADECANOATE

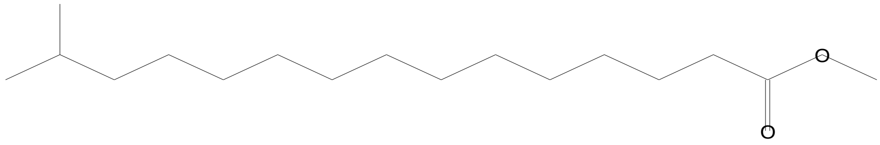

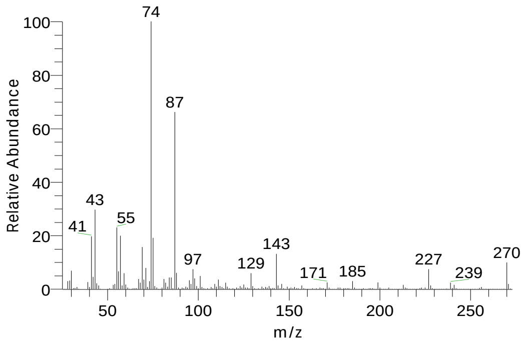

HEXADECANOIC ACID, METHYL ESTER
Formula C17H34O2, MW 270, CAS# 112-39-0, Entry# 161288
METHYL HEXADECANOATE

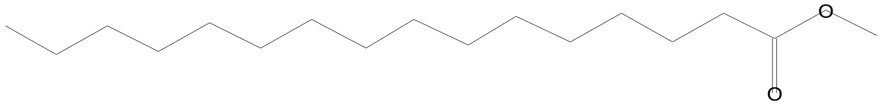

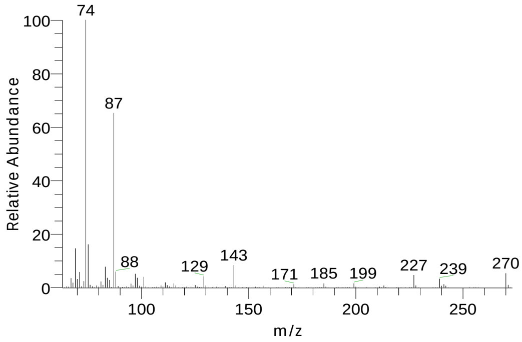

PENTADECANOIC ACID, 14-METHYL-, METHYL ESTER
Formula C17H34O2, MW 270, CAS# 5129-60-2, Entry# 161313
METHYL 14-METHYLPENTADECANOATE

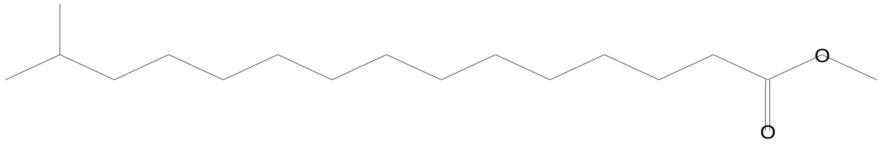

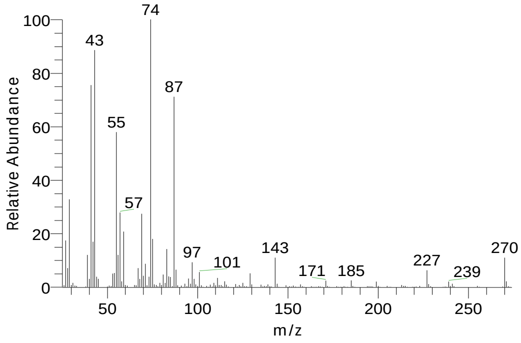

Palmitic Acid methyl ester
Formula C17H34O2, MW 270, CAS# 112-39-0, Entry# 1088
Palmitic acid, methyl ester

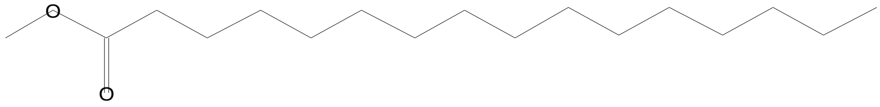

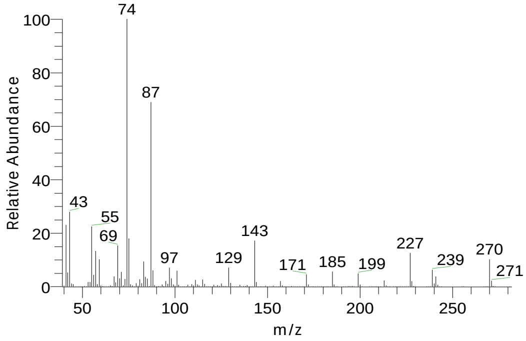

My GC-MS Report

Compound Structure | Hit Spectrum

HEXADECANOIC ACID, METHYL ESTER
Formula C17H34O2, MW 270, CAS# 112-39-0, Entry# 161284
METHYL HEXADECANOATE

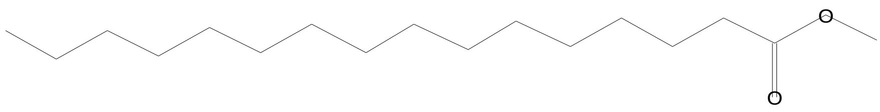

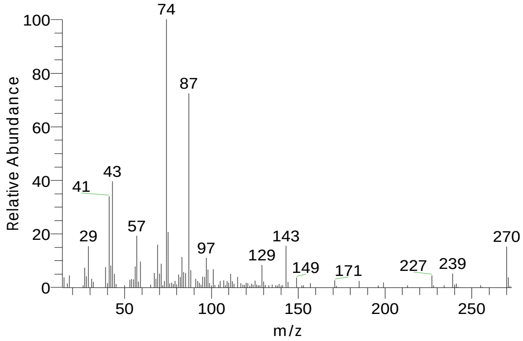

DrShreen_Egypt #6551 RT: 25.97 AV: 1 NL: 2.19E5
T: + c EI Full ms [50.000-750.000]

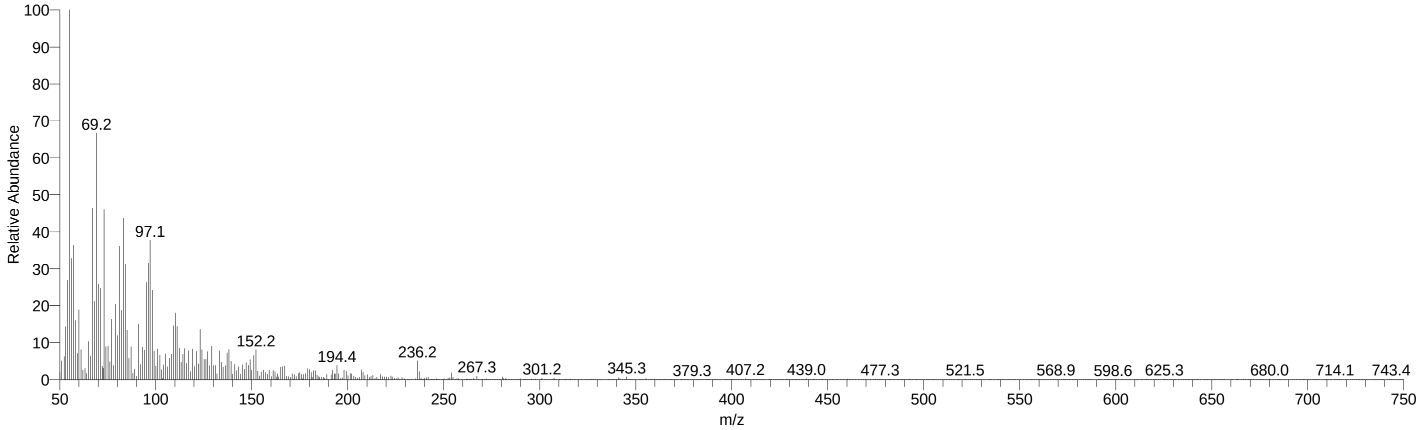

RT	Compound Name	Area %	MF	Molecular Formula	Molecular Weight	Cas #	Library
25.97	9-Hexadecenoic acid	0.88	851	C16H30O2	254	2091-29-4	mainlib
25.97	9-HEXADECENOIC ACID	0.88	851	C16H30O2	254	2091-29-4	WileyRegistry8e
25.97	9-OCTADECENOIC ACID (Z)-	0.88	815	C18H34O2	282	112-80-1	WileyRegistry8e
25.97	Palmitoleic acid	0.88	833	C16H30O2	254	373-49-9	mainlib
25.97	Palmitoleic acid	0.88	856	C16H30O2	254	373-49-9	replib

Compound Structure | Hit Spectrum

9-Hexadecenoic acid
Formula C16H30O2, MW 254, CAS# 2091-29-4, Entry# 19244
(9E)-9-Hexadecenoic acid #

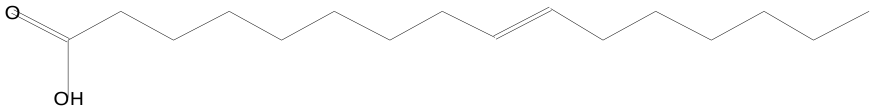

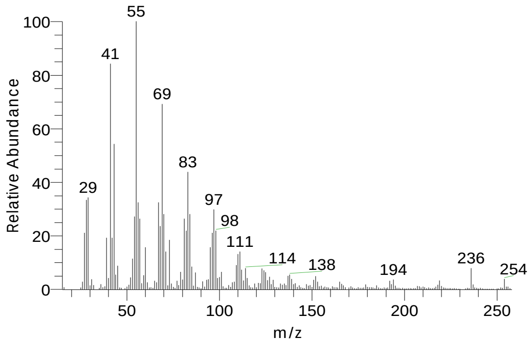

My GC-MS Report

Compound Structure | Hit Spectrum

9-HEXADECENOIC ACID
Formula C16H30O2, MW 254, CAS# 2091-29-4, Entry# 144722
(9E)-9-HEXADECENOIC ACID #

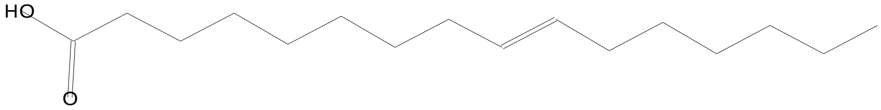

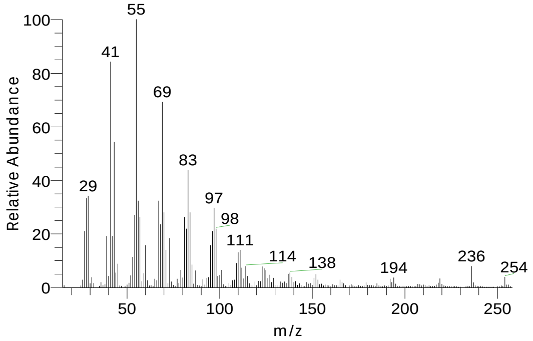

9-OCTADECENOIC ACID (Z)-
Formula C18H34O2, MW 282, CAS# 112-80-1, Entry# 172910
OCTADEC-9-ENOIC ACID

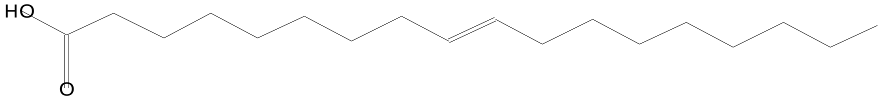

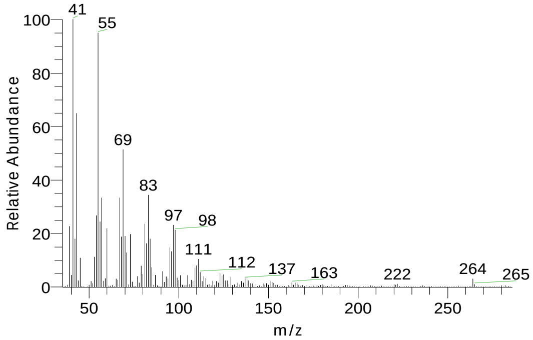

Palmitoleic acid
Formula C16H30O2, MW 254, CAS# 373-49-9, Entry# 20127
cis-9-Hexadecenoic acid

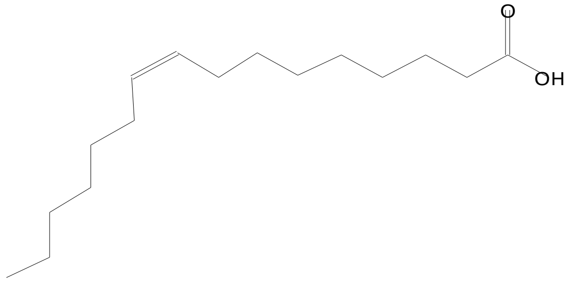

SI 811, RSI 833, mainlib, Entry# 20127, CAS# 373-49-9, Palmitoleic acid

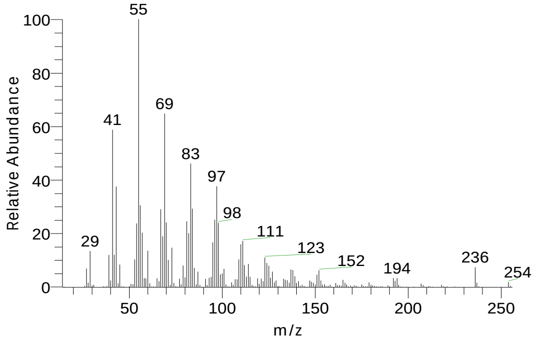

Palmitoleic acid
Formula C16H30O2, MW 254, CAS# 373-49-9, Entry# 5020
cis-9-Hexadecenoic acid

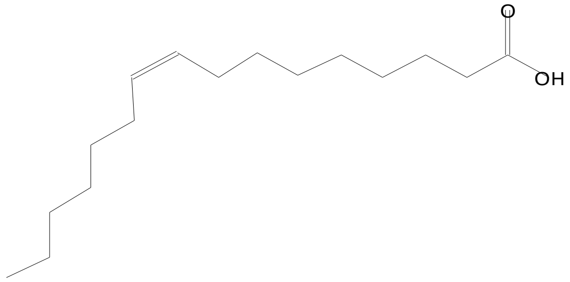

SI 800, RSI 856, replib, Entry# 5020, CAS# 373-49-9, Palmitoleic acid

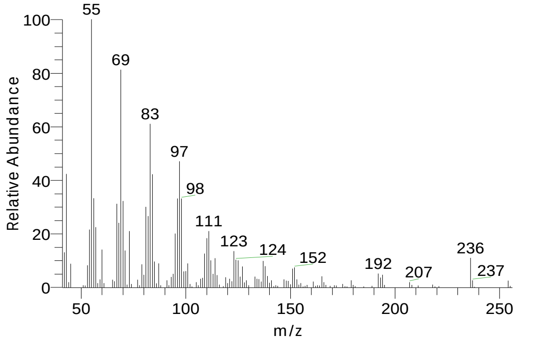

My GC-MS Report

DrShreen_Egypt #6710 RT: 26.50 AV: 1 NL: 5.66E6
T: + c EI Full ms [50.000-750.000]

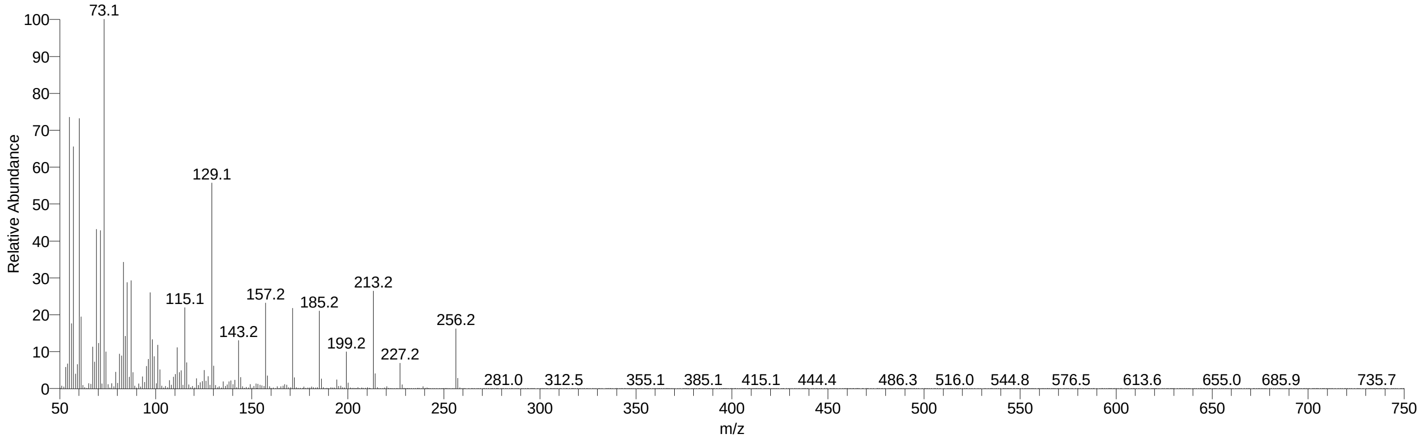

RT	Compound Name	Area %	MF	Molecular Formula	Molecular Weight	Cas #	Library
26.50	n-Hexadecanoic acid	17.43	924	C16H32O2	256	57-10-3	replib
26.50	n-Hexadecanoic acid	17.43	915	C16H32O2	256	57-10-3	replib
26.50	HEXADECANOIC ACID	17.43	903	C16H32O2	256	57-10-3	WileyRegistry8e
26.50	HEXADECANOIC ACID	17.43	902	C16H32O2	256	57-10-3	WileyRegistry8e
26.50	n-Hexadecanoic acid	17.43	878	C16H32O2	256	57-10-3	replib

Compound Structure	Hit Spectrum

n-Hexadecanoic acid
Formula C16H32O2, MW 256, CAS# 57-10-3, Entry# 7566
Hexadecanoic acid

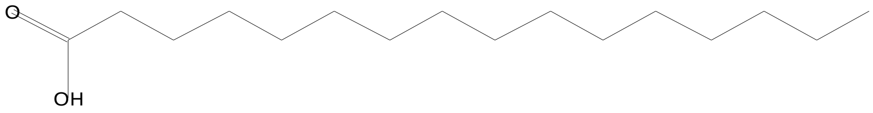

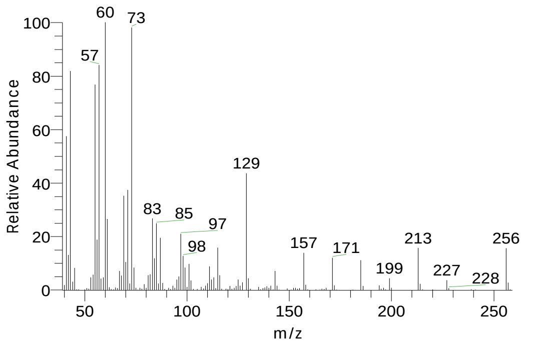

n-Hexadecanoic acid
Formula C16H32O2, MW 256, CAS# 57-10-3, Entry# 9622
Hexadecanoic acid

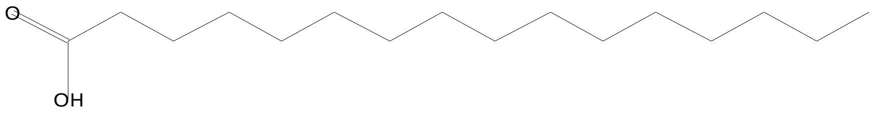

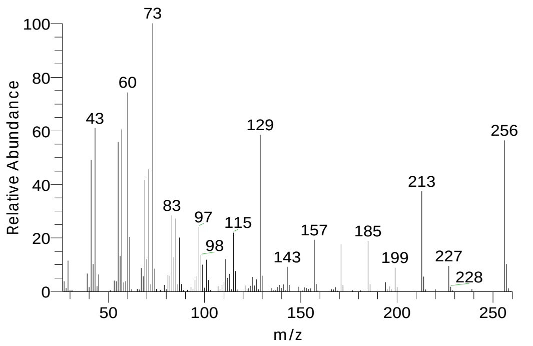

My GC-MS Report

Compound Structure | Hit Spectrum

HEXADECANOIC ACID
Formula C16H32O2, MW 256, CAS# 57-10-3, Entry# 397116
HEXADECANOATE

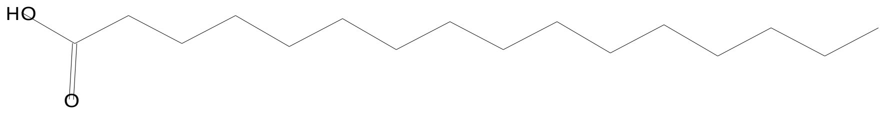

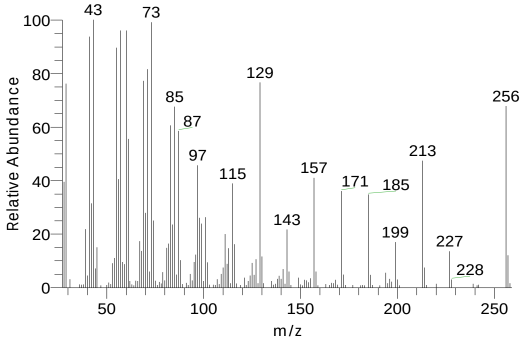

HEXADECANOIC ACID
Formula C16H32O2, MW 256, CAS# 57-10-3, Entry# 146744
HEXADECANOATE

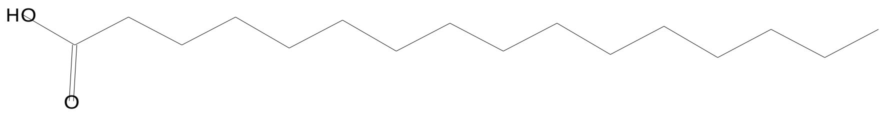

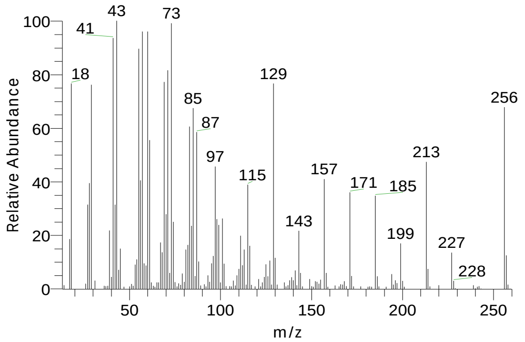

n-Hexadecanoic acid
Formula C16H32O2, MW 256, CAS# 57-10-3, Entry# 2779
Hexadecanoic acid

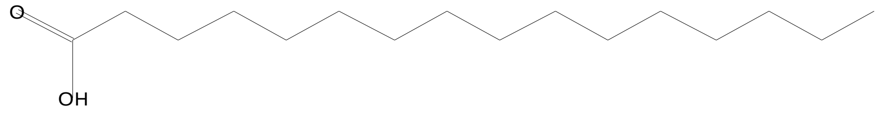

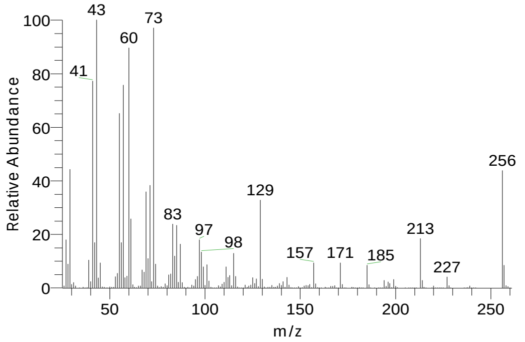

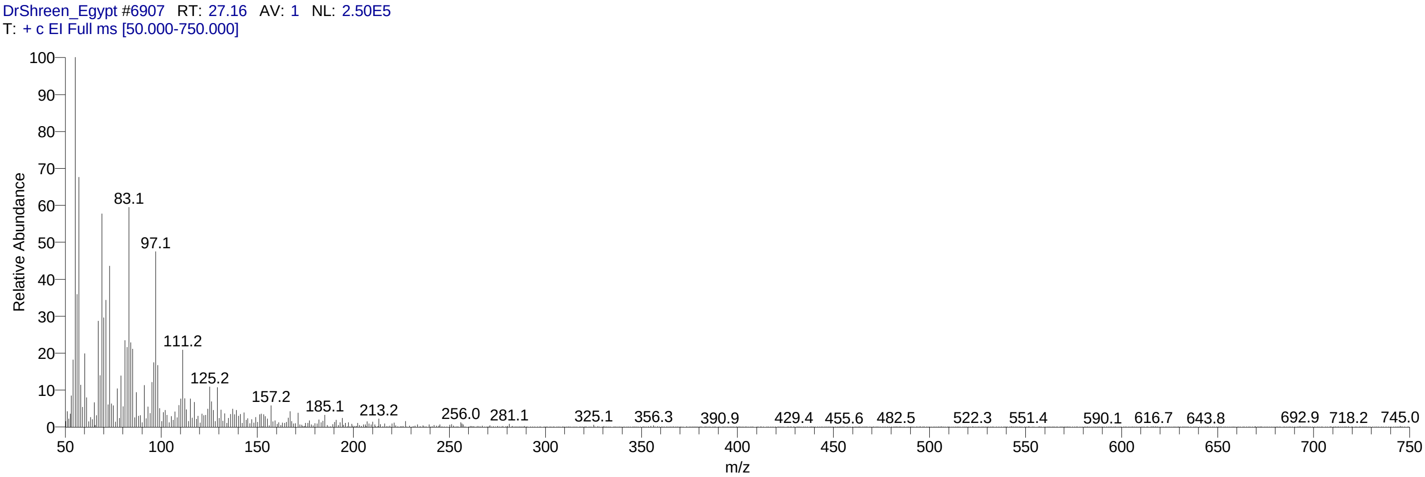

RT	Compound Name	Area %	MF	Molecular Formula	Molecular Weight	Cas #	Library
27.16	Oleic Acid	0.24	836	C18H34O2	282	112-80-1	replib
27.16	9-OCTADECENOIC ACID	0.24	836	C18H34O2	282	NA	WileyRegistry8e

My GC-MS Report

RT	Compound Name	Area %	MF	Molecular Formula	Molecular Weight	Cas #	Library
27.16	9-OCTADECENOIC ACID (Z)-	0.24	804	C18H34O2	282	112-80-1	WileyRegistry8e
27.16	Oleic Acid	0.24	777	C18H34O2	282	112-80-1	replib
27.16	Z-8-Methyl-9-tetradecenoic acid	0.24	789	C15H28O2	240	NA	mainlib

Compound Structure	Hit Spectrum

Oleic Acid
Formula C18H34O2, MW 282, CAS# 112-80-1, Entry# 4727
9-Octadecenoic acid (Z)-

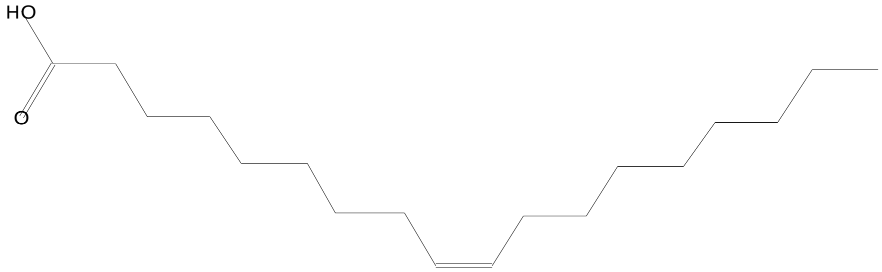

SI 776, RSI 836, replib, Entry# 4727, CAS# 112-80-1, Oleic Acid

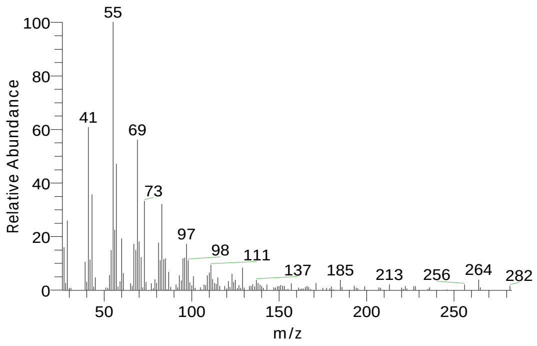

9-OCTADECENOIC ACID
Formula C18H34O2, MW 282, CAS# NA, Entry# 384641
9-OCTADECENSAEURE, (Z)- (OELSAEURE)

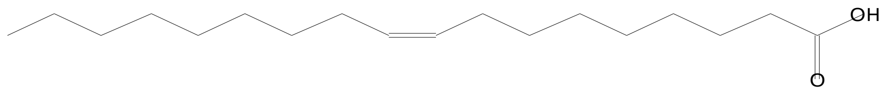

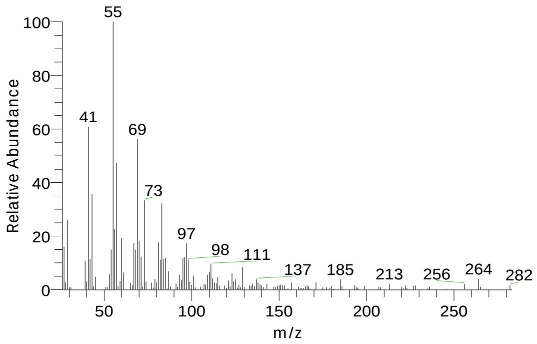

9-OCTADECENOIC ACID (Z)-
Formula C18H34O2, MW 282, CAS# 112-80-1, Entry# 172902
OCTADEC-9-ENOIC ACID

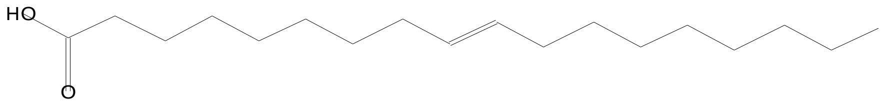

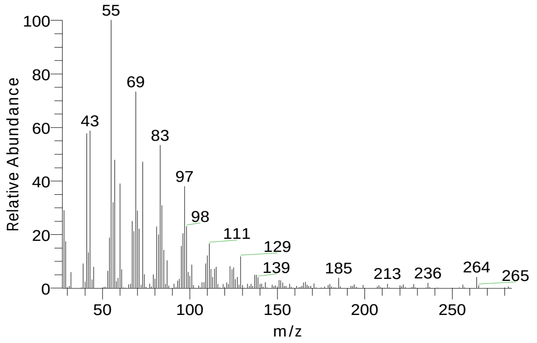

Oleic Acid
Formula C18H34O2, MW 282, CAS# 112-80-1, Entry# 4977
9-Octadecenoic acid (Z)-

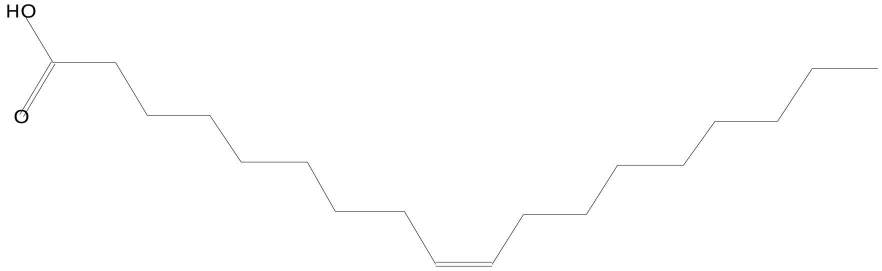

SI 773, RSI 777, replib, Entry# 4977, CAS# 112-80-1, Oleic Acid

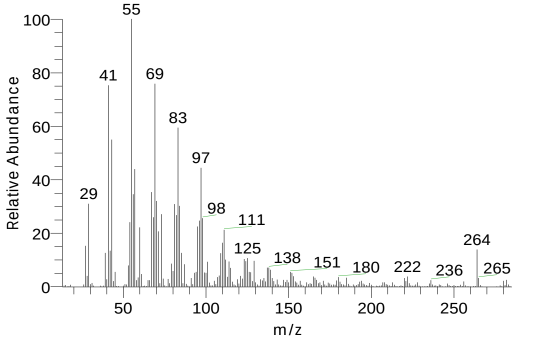

My GC-MS Report

Compound Structure | Hit Spectrum

Z-8-Methyl-9-tetradecenoic acid
Formula C15H28O2, MW 240, CAS# NA, Entry# 19015
(9Z)-8-Methyl-9-tetradecenoic acid #

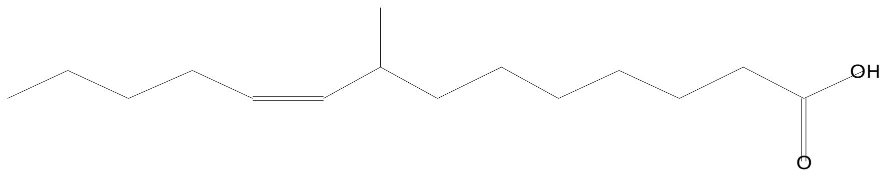

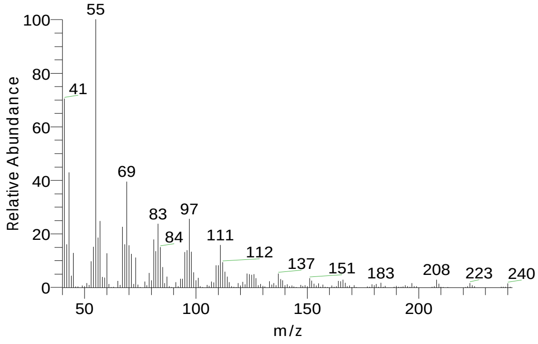

DrShreen_Egypt #7341 RT: 28.62 AV: 1 NL: 2.15E5
T: + c EI Full ms [50.000-750.000]

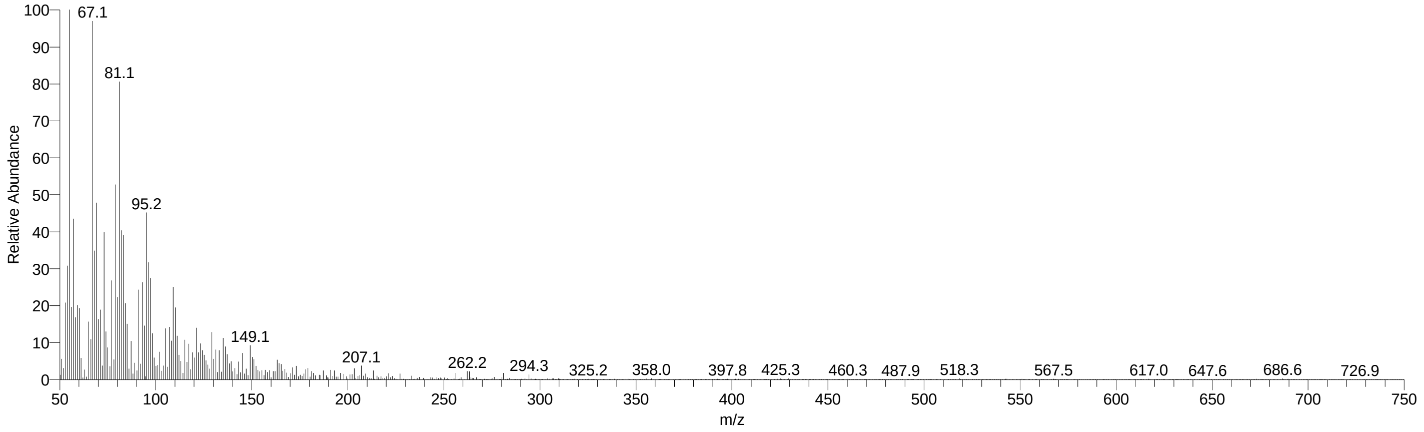

RT	Compound Name	Area %	MF	Molecular Formula	Molecular Weight	Cas #	Library
28.62	HEXADECADIENOIC ACID, METHYL ESTER	0.57	807	C17H30O2	266	29961-54-4	WileyRegistry8e
28.62	9,12-Octadecadienoyl chloride, (Z,Z)-	0.57	822	C18H31ClO	298	7459-33-8	replib
28.62	(9E,12E)-9,12-OCTADECADIENOYL CHLORIDE #	0.57	821	C18H31ClO	298	7459-33-8	WileyRegistry8e
28.62	17-Octadecynoic acid	0.57	800	C18H32O2	280	34450-18-5	mainlib
28.62	ETHYL (9Z,12Z)-9,12-OCTADECADIENOATE #	0.57	837	C20H36O2	308	544-35-4	WileyRegistry8e

Compound Structure | Hit Spectrum

HEXADECADIENOIC ACID, METHYL ESTER
Formula C17H30O2, MW 266, CAS# 29961-54-4, Entry# 157129
METHYL HEXADECADIENOATE

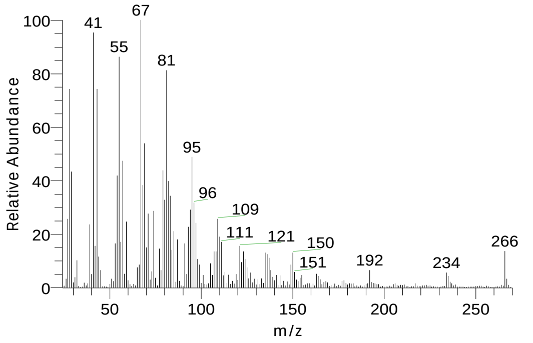

My GC-MS Report

Compound Structure | Hit Spectrum

9,12-Octadecadienoyl chloride, (Z,Z)-
Formula C18H31ClO, MW 298, CAS# 7459-33-8, Entry# 4940
Linoleoyl chloride

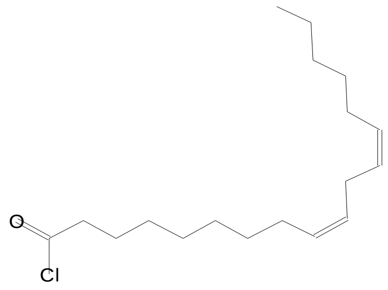

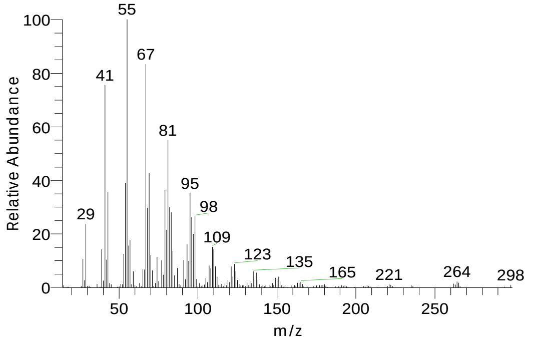

(9E,12E)-9,12-OCTADECADIENOYL CHLORIDE #
Formula C18H31ClO, MW 298, CAS# 7459-33-8, Entry# 187801
(9E,12E)-9,12-OCTADECADIENOYL CHLORIDE

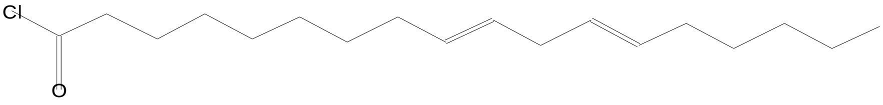

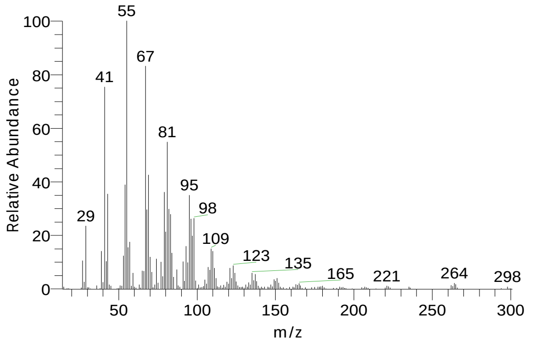

17-Octadecynoic acid
Formula C18H32O2, MW 280, CAS# 34450-18-5, Entry# 20510
$:28DZIILFGADWDKMF-UHFFFAOYSA-N

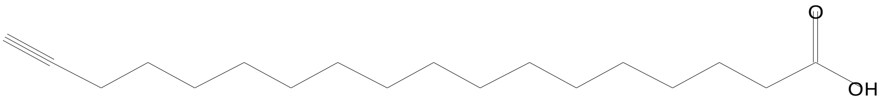

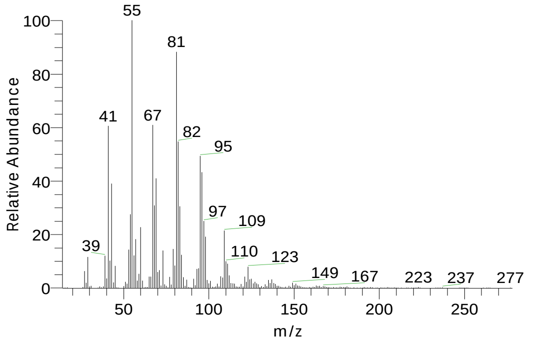

ETHYL (9Z,12Z)-9,12-OCTADECADIENOATE #
Formula C20H36O2, MW 308, CAS# 544-35-4, Entry# 196852
9,12-OCTADECADIENOIC ACID (9Z,12Z)-, ETHYL ESTER

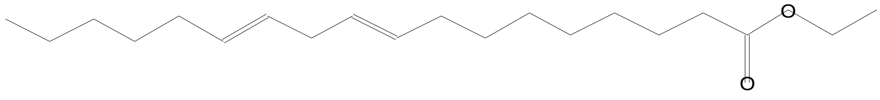

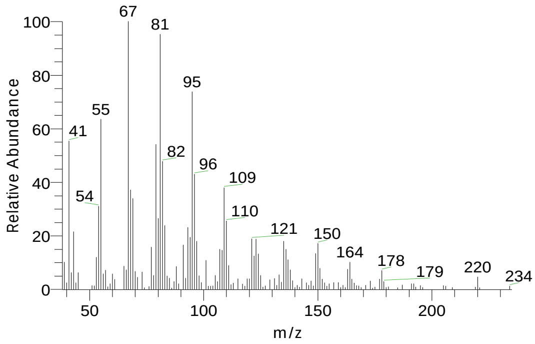

My GC-MS Report

DrShreen_Egypt #7395 RT: 28.80 AV: 1 NL: 2.41E5
T: + c EI Full ms [50.000-750.000]

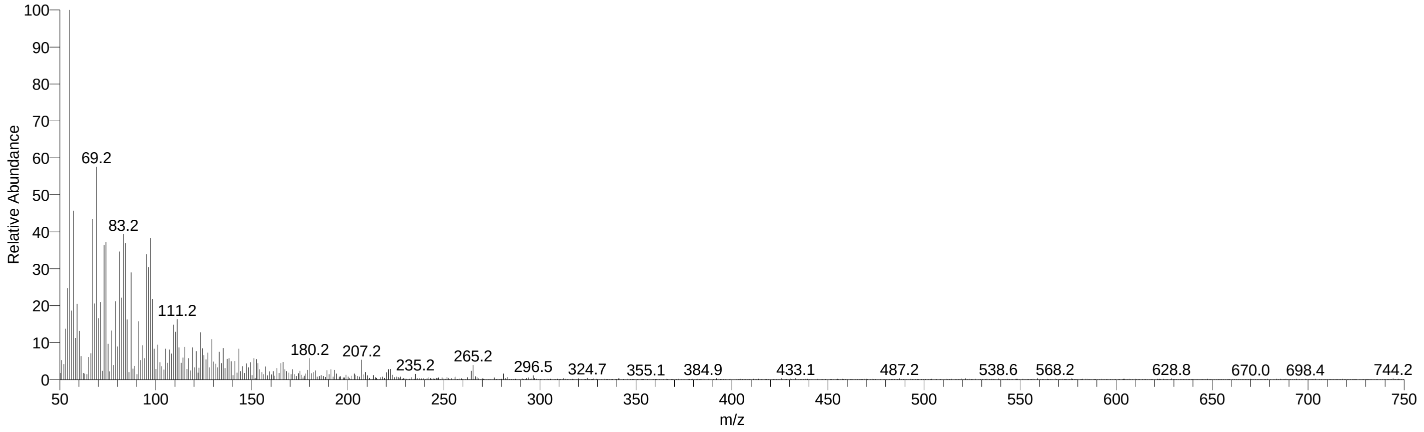

RT	Compound Name	Area %	MF	Molecular Formula	Molecular Weight	Cas #	Library
28.80	9-OCTADECENOIC ACID (Z)-	0.37	831	C18H34O2	282	112-80-1	WileyRegistry8e
28.80	11-Octadecenoic acid, methyl ester	0.37	820	C19H36O2	296	52380-33-3	mainlib
28.80	11-OCTADECENOIC ACID, METHYL ESTER	0.37	820	C19H36O2	296	52380-33-3	WileyRegistry8e
28.80	10-OCTADECENOIC ACID, METHYL ESTER	0.37	804	C19H36O2	296	13481-95-3	WileyRegistry8e
28.80	10-Octadecenoic acid, methyl ester	0.37	803	C19H36O2	296	13481-95-3	mainlib

Compound Structure | Hit Spectrum

9-OCTADECENOIC ACID (Z)-
Formula C18H34O2, MW 282, CAS# 112-80-1, Entry# 172910
OCTADEC-9-ENOIC ACID

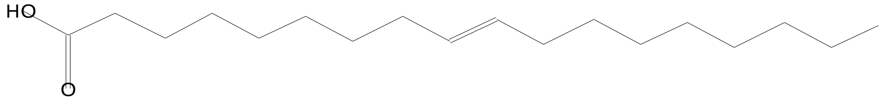

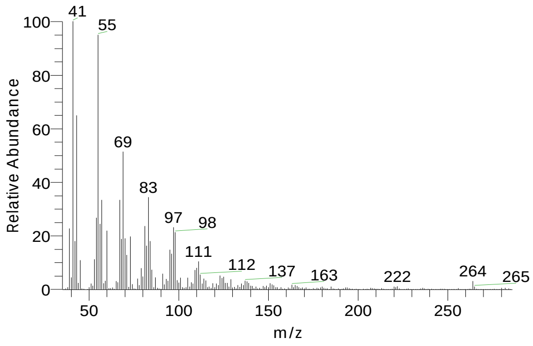

11-Octadecenoic acid, methyl ester
Formula C19H36O2, MW 296, CAS# 52380-33-3, Entry# 19082
Methyl 11-octadecenoate

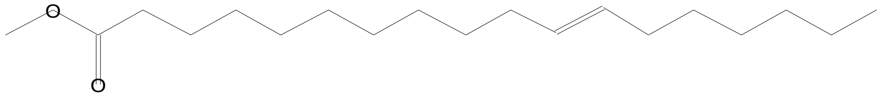

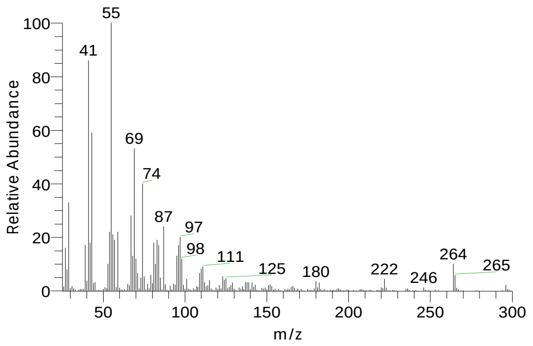

My GC-MS Report

Compound Structure	Hit Spectrum

11-OCTADECENOIC ACID, METHYL ESTER
Formula C19H36O2, MW 296, CAS# 52380-33-3, Entry# 186176
OCTADEC-11-ENOIC ACID METHYL ESTER

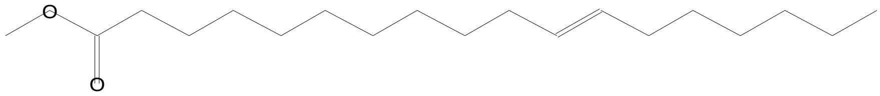

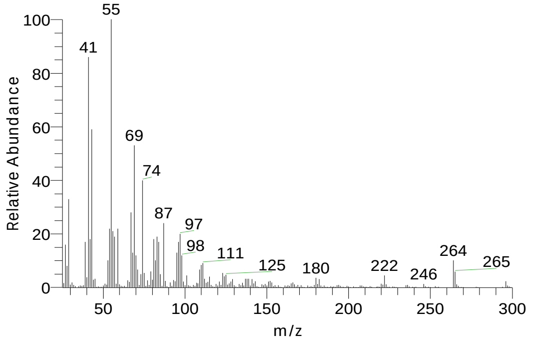

10-OCTADECENOIC ACID, METHYL ESTER
Formula C19H36O2, MW 296, CAS# 13481-95-3, Entry# 186173
METHYL OCTADEC-10-ENOATE

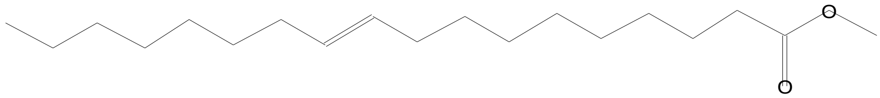

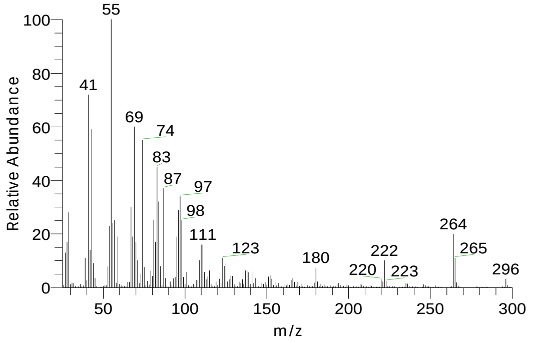

10-Octadecenoic acid, methyl ester
Formula C19H36O2, MW 296, CAS# 13481-95-3, Entry# 19319
Methyl 10-octadecenoate

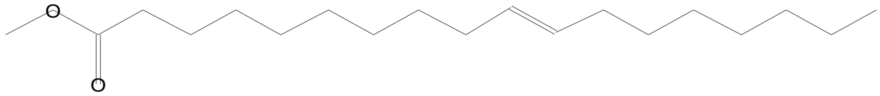

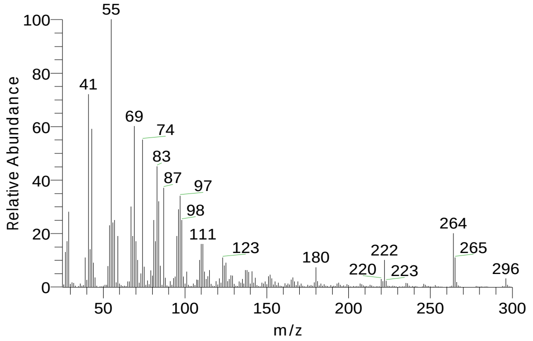

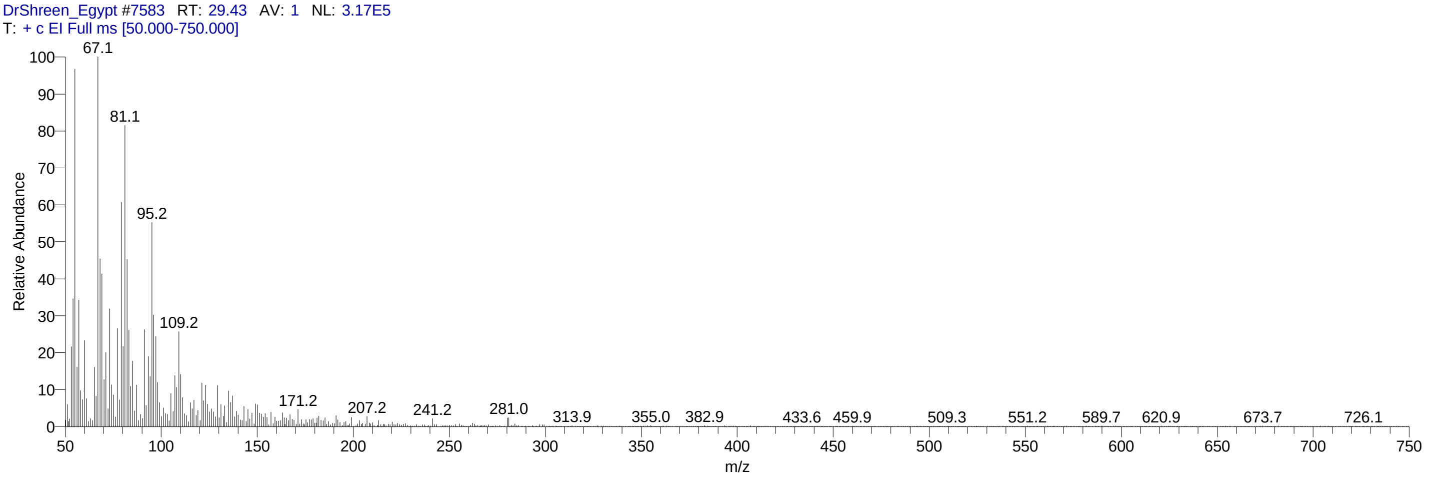

RT	Compound Name	Area %	MF	Molecular Formula	Molecular Weight	Cas #	Library
29.43	HEXADECADIENOIC ACID, METHYL ESTER	0.47	810	C17H30O2	266	29961-54-4	WileyRegistry8e
29.43	9,12-OCTADECADIENOIC ACID (Z,Z)-	0.47	825	C18H32O2	280	60-33-3	WileyRegistry8e
29.43	9,12-Octadecadienoic acid (Z,Z)-	0.47	819	C18H32O2	280	60-33-3	replib

My GC-MS Report

RT	Compound Name	Area %	MF	Molecular Formula	Molecular Weight	Cas #	Library
29.43	9,12-Octadecadienoic acid (Z,Z)-	0.47	824	C18H32O2	280	60-33-3	replib
29.43	Cyclopropaneoctanoic acid, 2-[[2-[(2-ethylcyclopropyl)methyl]cyclopropyl]methyl]-, methyl ester	0.47	833	C22H38O2	334	10152-71-3	mainlib

Compound Structure | Hit Spectrum

HEXADECADIENOIC ACID, METHYL ESTER
Formula C17H30O2, MW 266, CAS# 29961-54-4, Entry# 157129
METHYL HEXADECADIENOATE

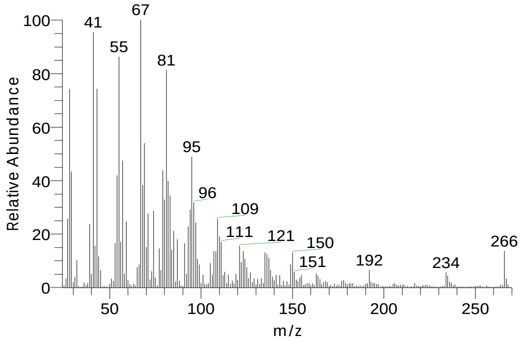

9,12-OCTADECADIENOIC ACID (Z,Z)-
Formula C18H32O2, MW 280, CAS# 60-33-3, Entry# 170904
(9E,12E)-9,12-OCTADECADIENOIC ACID #

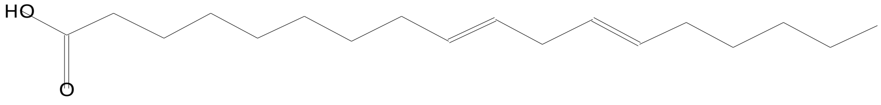

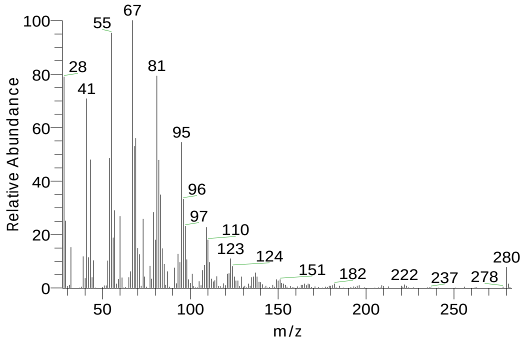

9,12-Octadecadienoic acid (Z,Z)-
Formula C18H32O2, MW 280, CAS# 60-33-3, Entry# 8112
cis-9,cis-12-Octadecadienoic acid

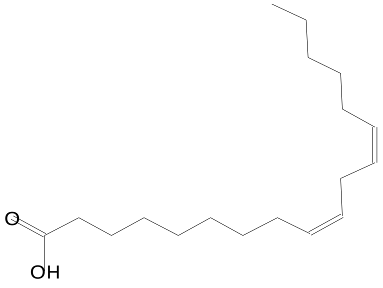

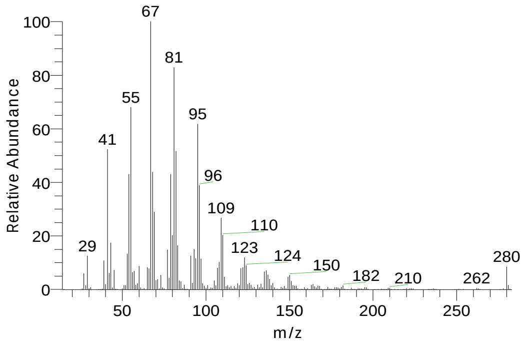

9,12-Octadecadienoic acid (Z,Z)-
Formula C18H32O2, MW 280, CAS# 60-33-3, Entry# 8057
cis-9,cis-12-Octadecadienoic acid

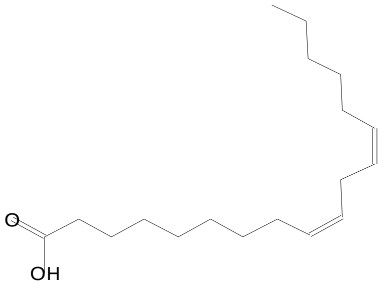

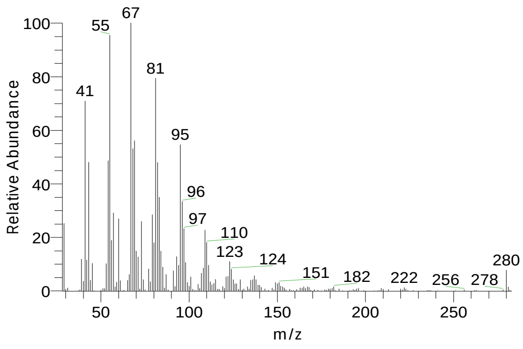

My GC-MS Report

Compound Structure | Hit Spectrum

Formula C22H38O2, MW 334, CAS# 10152-71-3, Entry# 2765
Methyl 8-[2-((2-[(2-ethylcyclopropyl)methyl]cyclopropyl)methyl)cyclopropyl]octanoate #

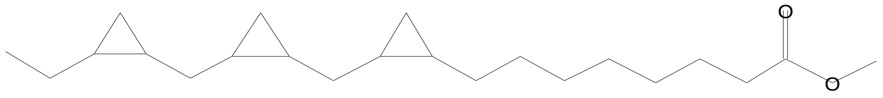

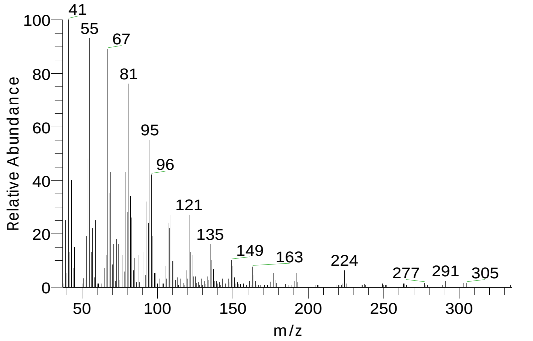

DrShreen_Egypt #7628 RT: 29.58 AV: 1 NL: 1.79E6
T: + c EI Full ms [50.000-750.000]

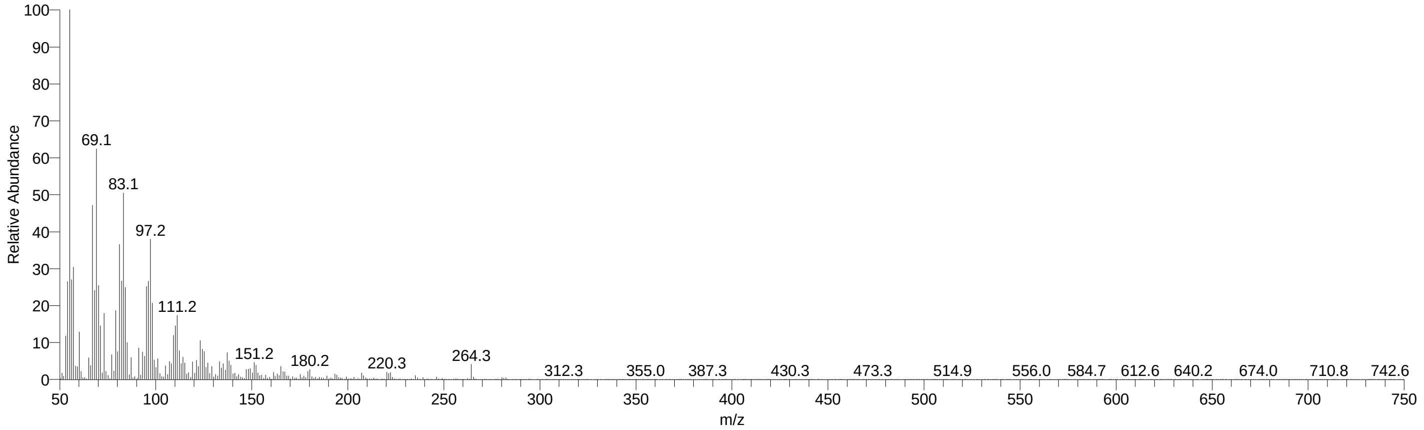

RT	Compound Name	Area %	MF	Molecular Formula	Molecular Weight	Cas #	Library
29.58	cis-Vaccenic acid	4.66	906	C18H34O2	282	506-17-2	mainlib
29.58	trans-13-Octadecenoic acid	4.66	904	C18H34O2	282	693-71-0	mainlib
29.58	Oleic Acid	4.66	908	C18H34O2	282	112-80-1	replib
29.58	cis-13-Octadecenoic acid	4.66	900	C18H34O2	282	13126-39-1	mainlib
29.58	9-Octadecenoic acid, (E)-	4.66	908	C18H34O2	282	112-79-8	replib

Compound Structure | Hit Spectrum

cis-Vaccenic acid
Formula C18H34O2, MW 282, CAS# 506-17-2, Entry# 20090
11-Octadecenoic acid, (Z)-

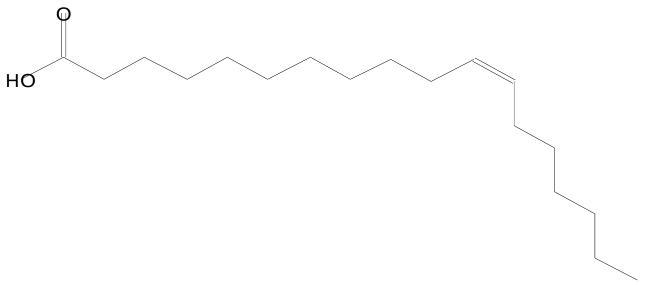

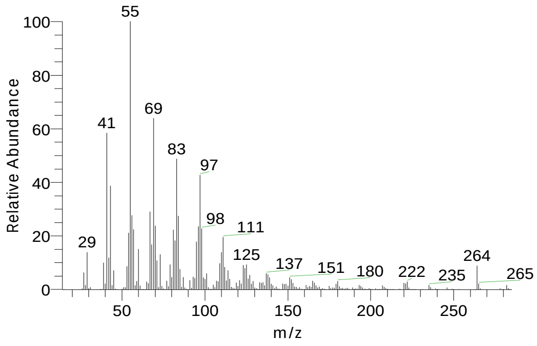

My GC-MS Report

Compound Structure | Hit Spectrum

trans-13-Octadecenoic acid
Formula C18H34O2, MW 282, CAS# 693-71-0, Entry# 19306
$:28BDLLSHRIFPDGQB-AATRIKPKSA-N

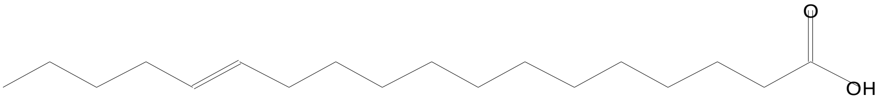

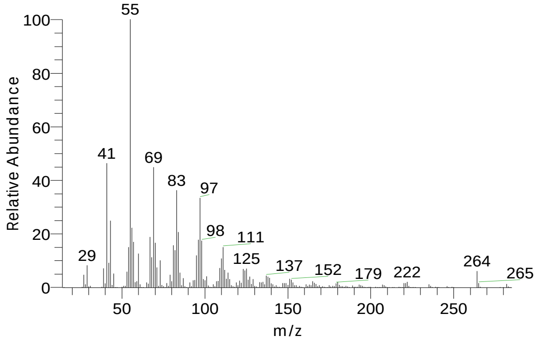

Oleic Acid
Formula C18H34O2, MW 282, CAS# 112-80-1, Entry# 5017
9-Octadecenoic acid (Z)-

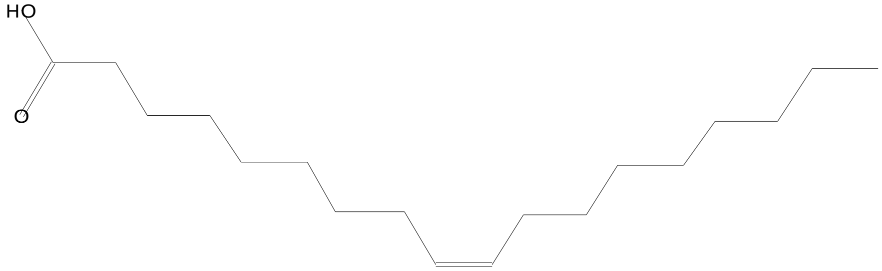

SI 899, RSI 908, replib, Entry# 5017, CAS# 112-80-1, Oleic Acid

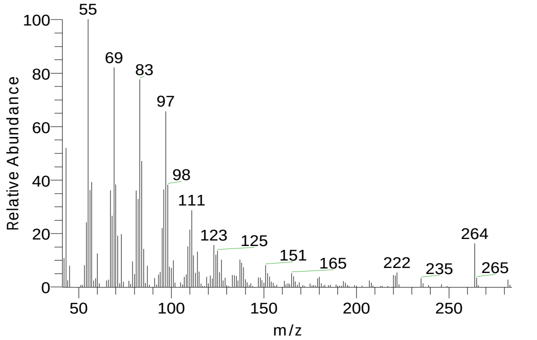

cis-13-Octadecenoic acid
Formula C18H34O2, MW 282, CAS# 13126-39-1, Entry# 20126
$:28BDLLSHRIFPDGQB-WAYWQWQTSA-N

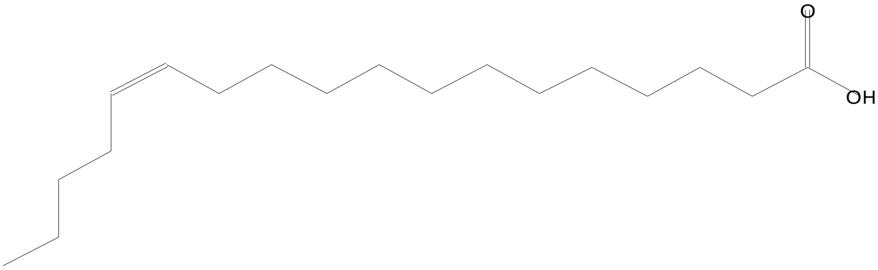

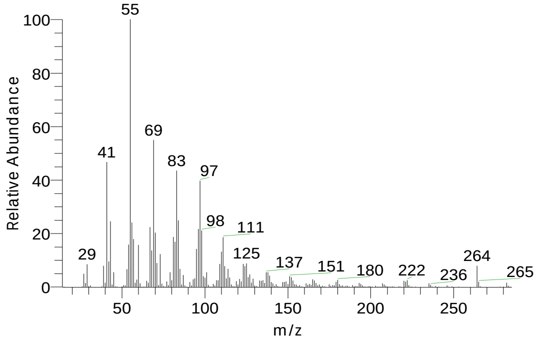

9-Octadecenoic acid, (E)-
Formula C18H34O2, MW 282, CAS# 112-79-8, Entry# 5015
trans-ë(sup 9)-Octadecenoic acid

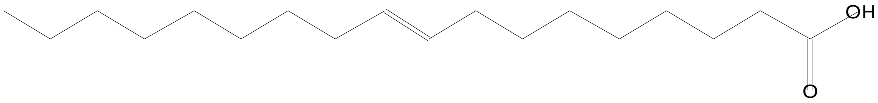

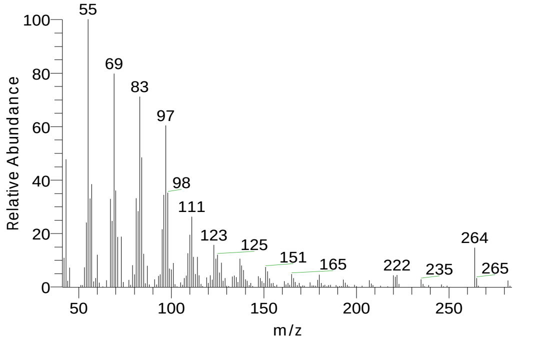

My GC-MS Report

DrShreen_Egypt #7775 RT: 30.07 AV: 1 NL: 1.03E6
T: + c EI Full ms [50.000-750.000]

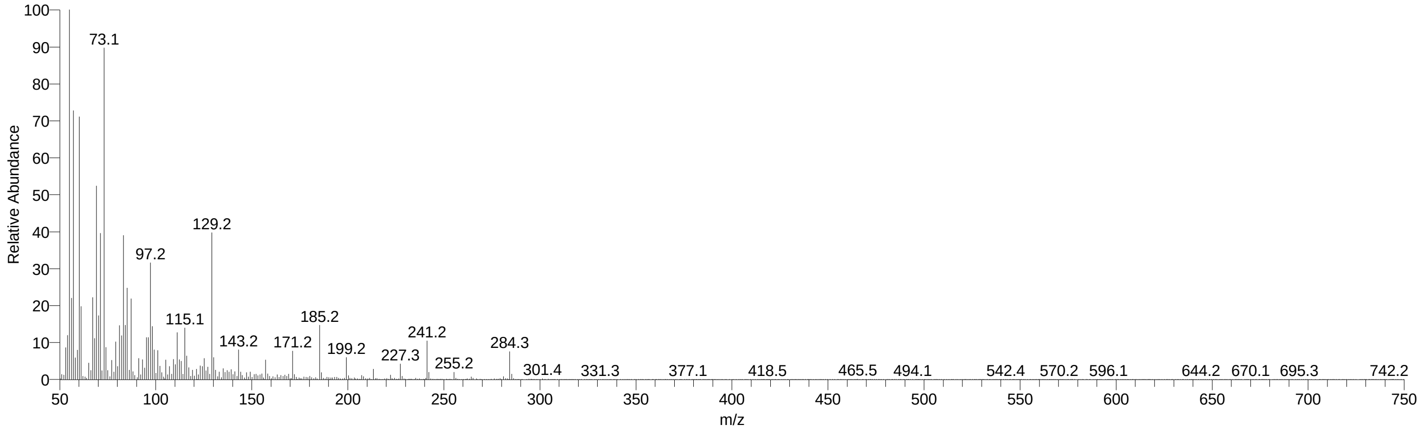

RT	Compound Name	Area %	MF	Molecular Formula	Molecular Weight	Cas #	Library
30.07	Octadecanoic acid	2.59	932	C18H36O2	284	57-11-4	replib
30.07	OCTADECANOIC ACID	2.59	861	C18H36O2	284	57-11-4	WileyRegistry8e
30.07	Octadecanoic acid	2.59	849	C18H36O2	284	57-11-4	mainlib
30.07	OCTADECANOIC ACID	2.59	867	C18H36O2	284	57-11-4	WileyRegistry8e
30.07	Octadecanoic acid	2.59	865	C18H36O2	284	57-11-4	replib

Compound Structure | Hit Spectrum

Octadecanoic acid
Formula C18H36O2, MW 284, CAS# 57-11-4, Entry# 2781
Stearic acid

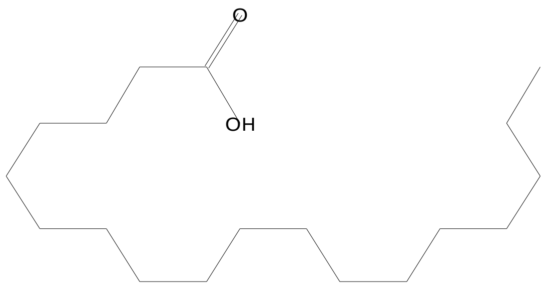

SI 854, RSI 932, replib, Entry# 2781, CAS# 57-11-4, Octadecanoic acid

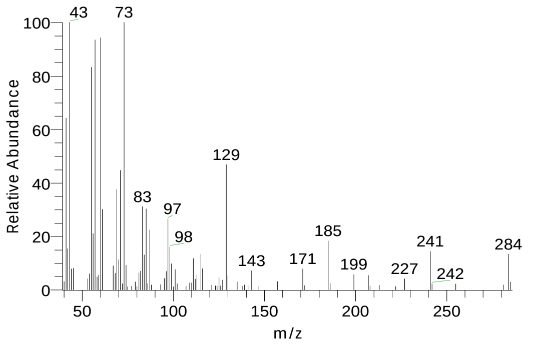

OCTADECANOIC ACID
Formula C18H36O2, MW 284, CAS# 57-11-4, Entry# 174897
STEARATE

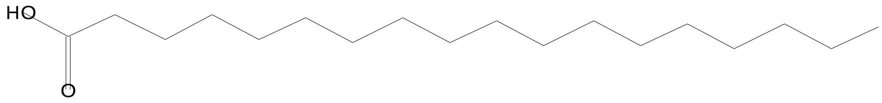

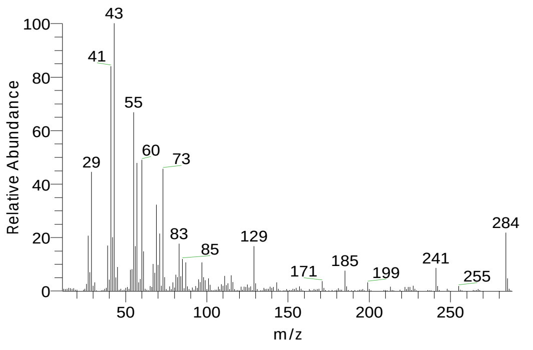

My GC-MS Report

Compound Structure | Hit Spectrum

Octadecanoic acid
Formula C18H36O2, MW 284, CAS# 57-11-4, Entry# 9210
Stearic acid

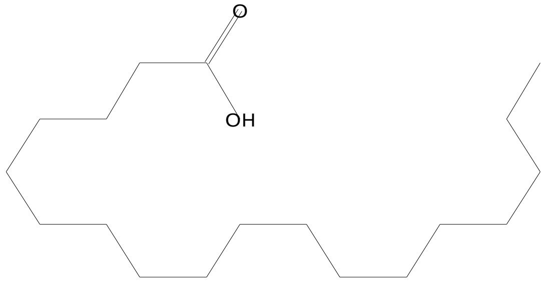

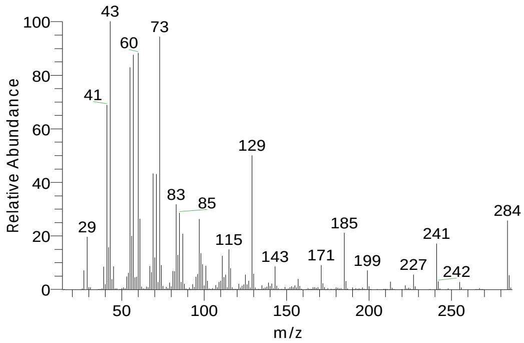

OCTADECANOIC ACID
Formula C18H36O2, MW 284, CAS# 57-11-4, Entry# 174902
STEARATE

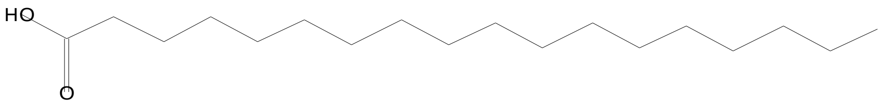

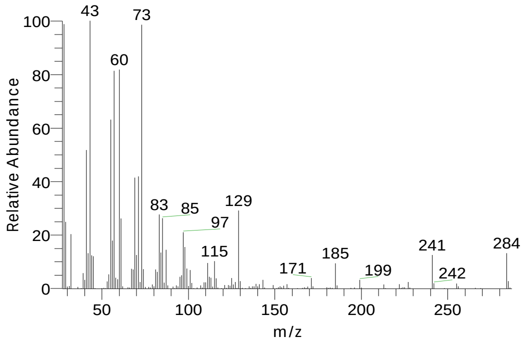

Octadecanoic acid
Formula C18H36O2, MW 284, CAS# 57-11-4, Entry# 1866
Stearic acid

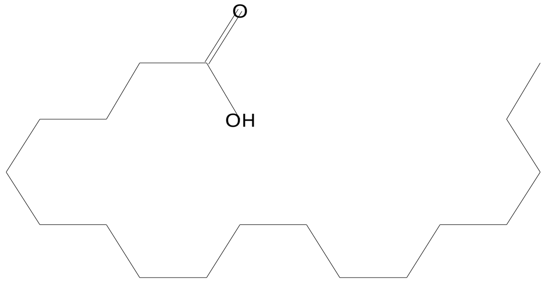

SI 839, RSI 865, replib, Entry# 1866, CAS# 57-11-4, Octadecanoic acid

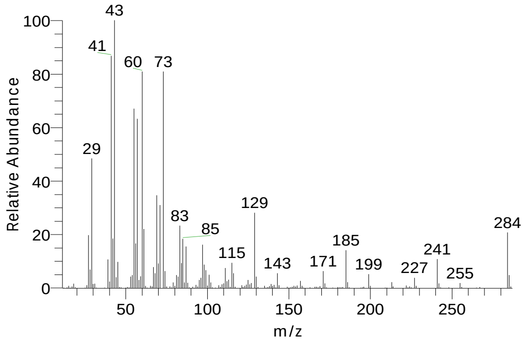

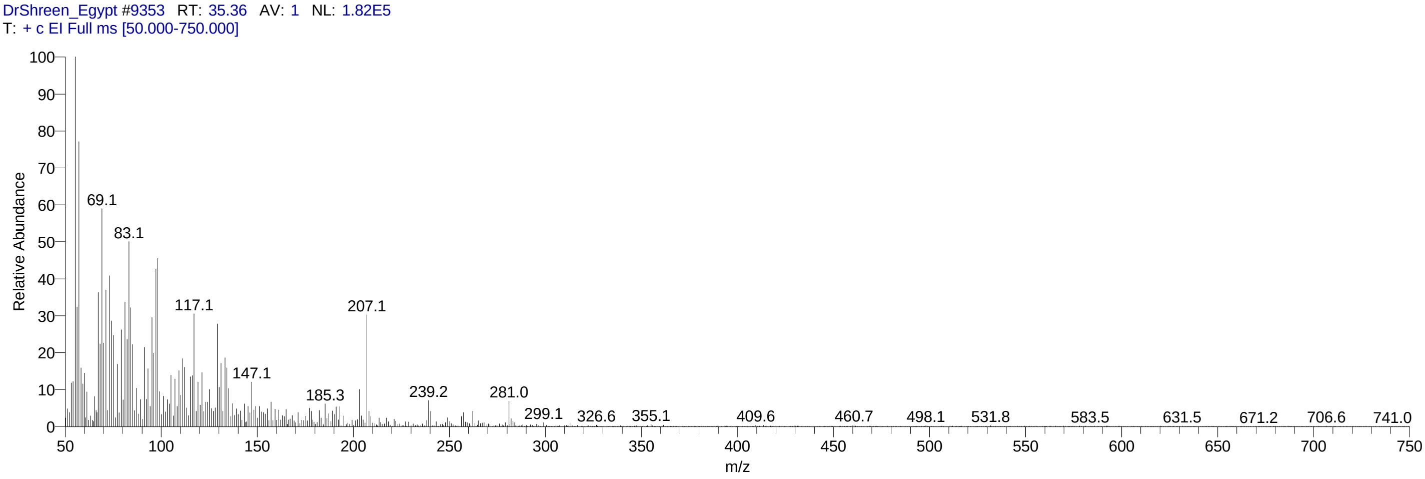

RT	Compound Name	Area %	MF	Molecular Formula	Molecular Weight	Cas #	Library
35.36	9,12,15-OCTADECATRIENOIC ACID, 2-[(TRIMETHYLSILYL)OXY]-1-[[(TRIMETHYLSILYL)OXY]METHYL]ETHYL ESTER, (Z,Z,Z)-	0.52	795	C27H52O4Si2	496	55521-23-8	WileyRegistry8e

My GC-MS Report

RT	Compound Name	Area %	MF	Molecular Formula	Molecular Weight	Cas #	Library
35.36	HEXADECANOIC ACID, 2,3-DIHYDROXYPROPYL ESTER	0.52	743	C19H38O4	330	542-44-9	WileyRegistry8e
35.36	2-HYDROXY-3-[(9E)-9-OCTADECENOYLOXY]PROPYL (9E)-9-OCTADECENOATE #	0.52	722	C39H72O5	620	2465-32-9	WileyRegistry8e
35.36	Oleic Acid	0.52	722	C18H34O2	282	112-80-1	replib
35.36	Hexadecanoic acid, 1-(hydroxymethyl)-1,2-ethanediyl ester	0.52	711	C35H68O5	568	761-35-3	mainlib

Compound Structure | Hit Spectrum

Formula C27H52O4Si2, MW 496, CAS# 55521-23-8, Entry# 284835

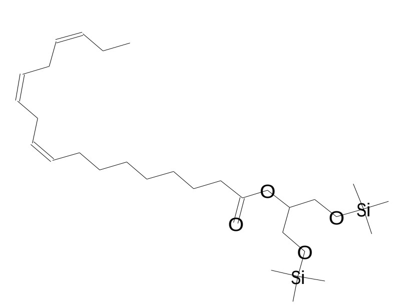

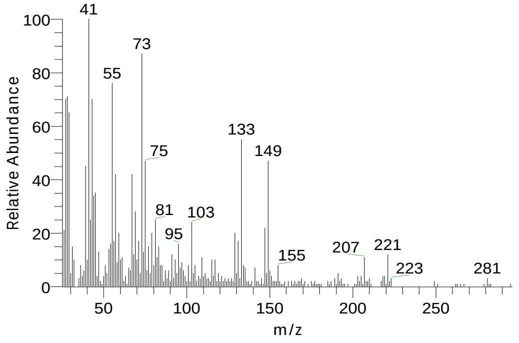

HEXADECANOIC ACID, 2,3-DIHYDROXYPROPYL ESTER
Formula C19H38O4, MW 330, CAS# 542-44-9, Entry# 214589
2,3-DIHYDROXYPROPYL PALMITATE #

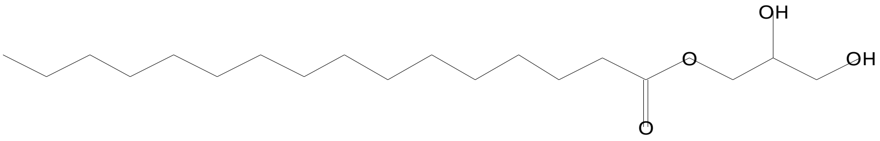

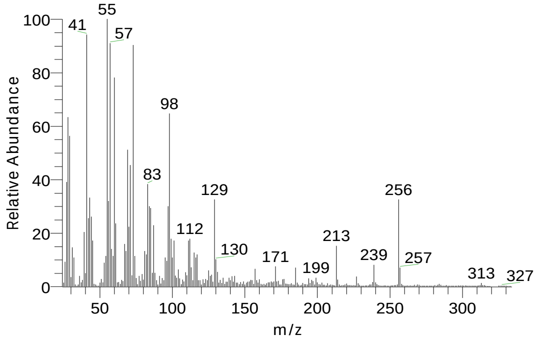

2-HYDROXY-3-[(9E)-9-OCTADECENOYLOXY]PROPYL (9E)-9-OCTADECENOATE #
Formula C39H72O5, MW 620, CAS# 2465-32-9, Entry# 298152
(Z,Z)-1,3-DIOCTADECENOYL GLYCEROL

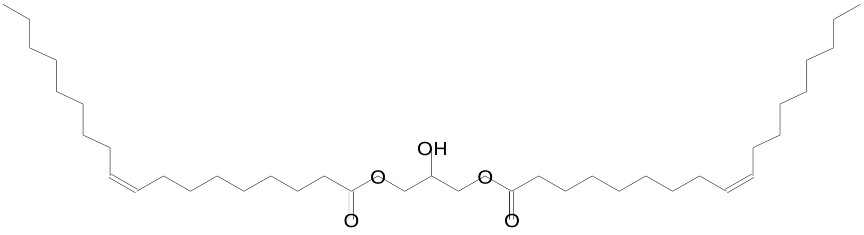

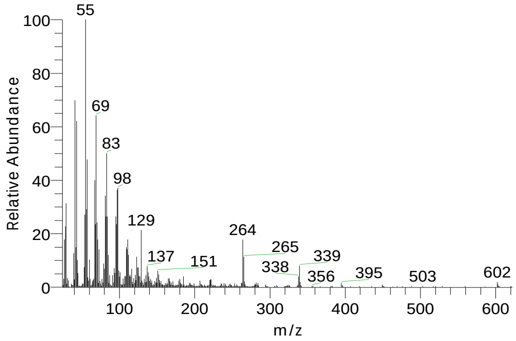

My GC-MS Report

Compound Structure

Hit Spectrum

Oleic Acid
Formula C18H34O2, MW 282, CAS# 112-80-1, Entry# 4977
9-Octadecenoic acid (Z)-

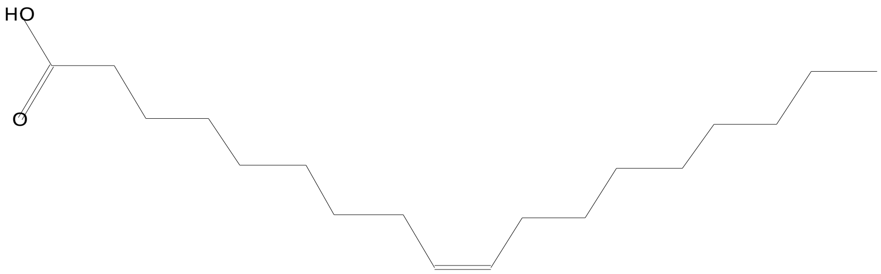

SI 711, RSI 722, replib, Entry# 4977, CAS# 112-80-1, Oleic Acid

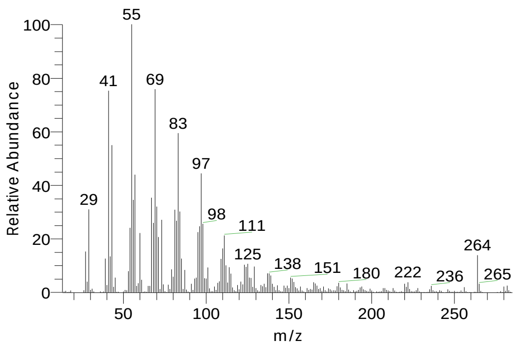

Hexadecanoic acid, 1-(hydroxymethyl)-1,2-ethanediyl ester
Formula C35H68O5, MW 568, CAS# 761-35-3, Entry# 7720
Palmitin, 1,2-di-

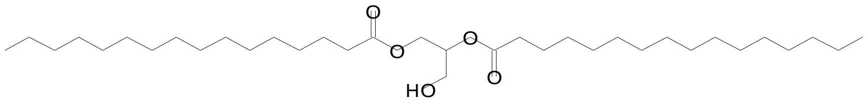

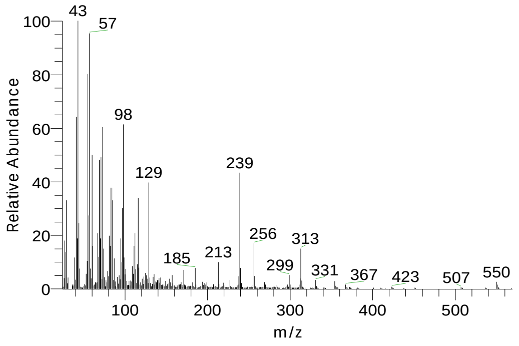

DrShreen_Egypt #9486 RT: 35.81 AV: 1 NL: 1.89E6
T: + c EI Full ms [50.000-750.000]

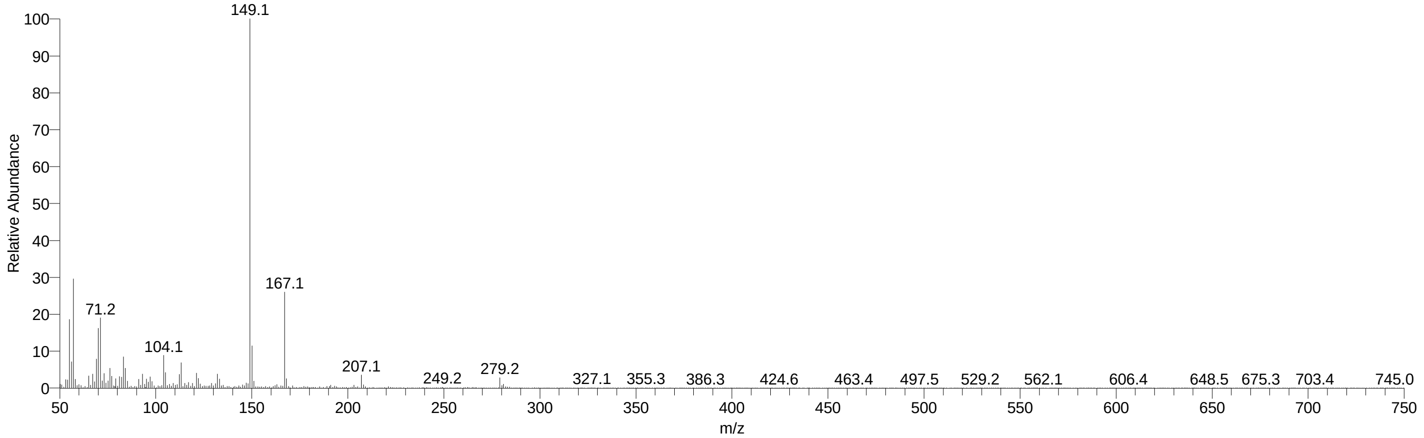

RT	Compound Name	Area %	MF	Molecular Formula	Molecular Weight	Cas #	Library
35.81	Diisooctyl phthalate	1.38	940	C24H38O4	390	131-20-4	replib
35.81	1,2-BENZENEDICARBOXYLIC ACID, 3-NITRO-	1.38	882	C8H5NO6	211	603-11-2	WileyRegistry8e
35.81	2-([(2-ETHYLHEXYL)OXY]CARBONYL)BENZOIC ACID #	1.38	847	C16H22O4	278	4376-20-9	WileyRegistry8e
35.81	Mono(2-ethylhexyl) phthalate	1.38	836	C16H22O4	278	4376-20-9	replib
35.81	Bis(2-ethylhexyl) phthalate	1.38	857	C24H38O4	390	117-81-7	replib

My GC-MS Report

Compound Structure | Hit Spectrum

Diisooctyl phthalate
Formula C24H38O4, MW 390, CAS# 131-20-4, Entry# 23542
Bis(6-methylheptyl) phthalate

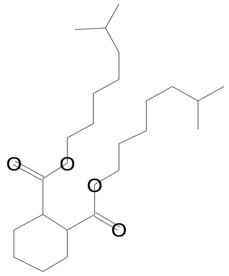

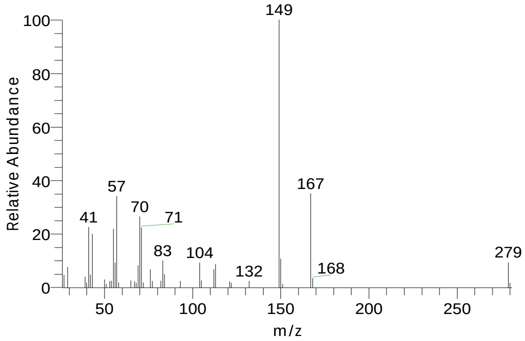

1,2-BENZENEDICARBOXYLIC ACID, 3-NITRO-
Formula C8H5NO6, MW 211, CAS# 603-11-2, Entry# 96710
3-NITROPHTHALIC ACID

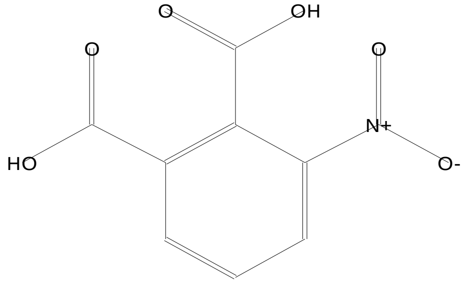

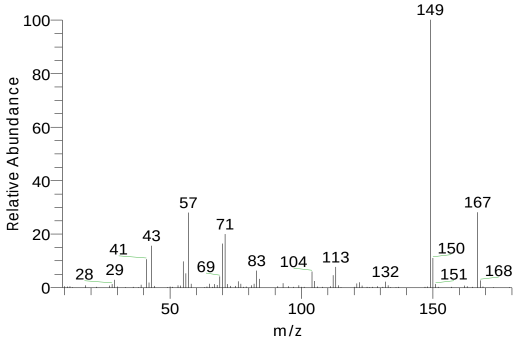

2-([(2-ETHYLHEXYL)OXY]CARBONYL)BENZOIC ACID #
Formula C16H22O4, MW 278, CAS# 4376-20-9, Entry# 168625
(2-ETHYLHEXYL) HYDROGEN PHTHALATE

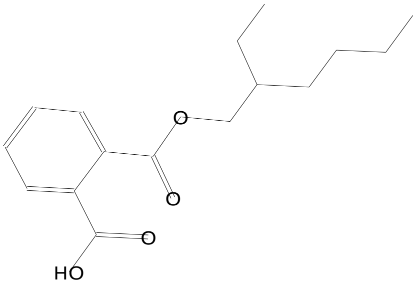

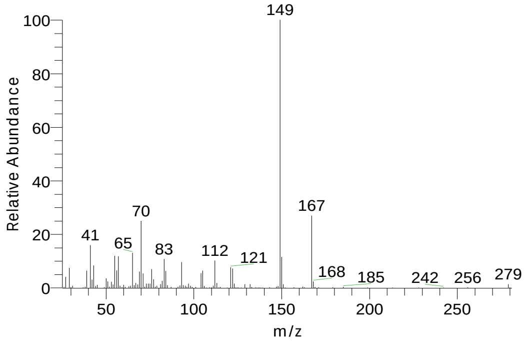

Mono(2-ethylhexyl) phthalate
Formula C16H22O4, MW 278, CAS# 4376-20-9, Entry# 23543
1,2-Benzenedicarboxylic acid, mono(2-ethylhexyl) ester

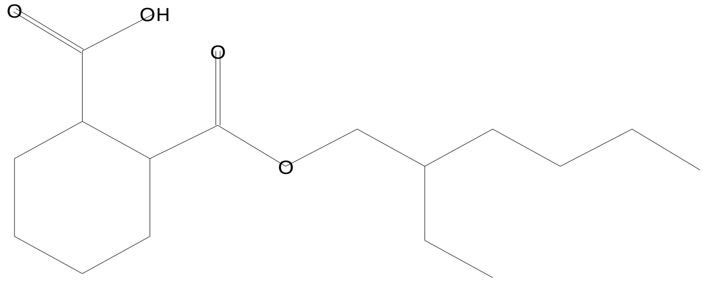

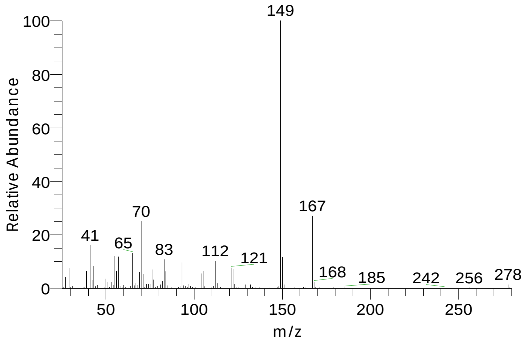

My GC-MS Report

Compound Structure

Hit Spectrum

Bis(2-ethylhexyl) phthalate
Formula C24H38O4, MW 390, CAS# 117-81-7, Entry# 23540
Phthalic acid, bis(2-ethylhexyl) ester

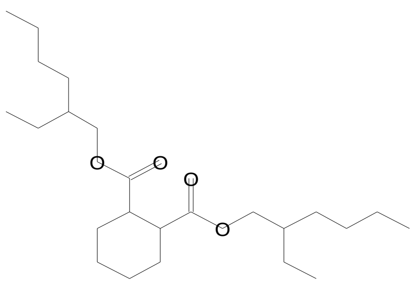

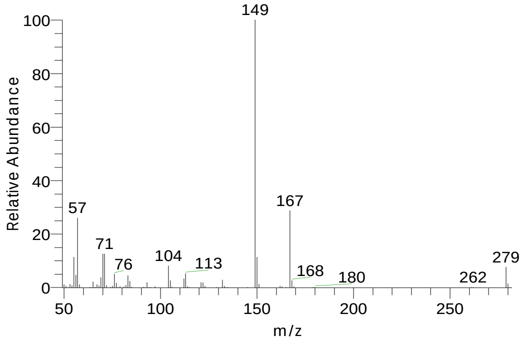

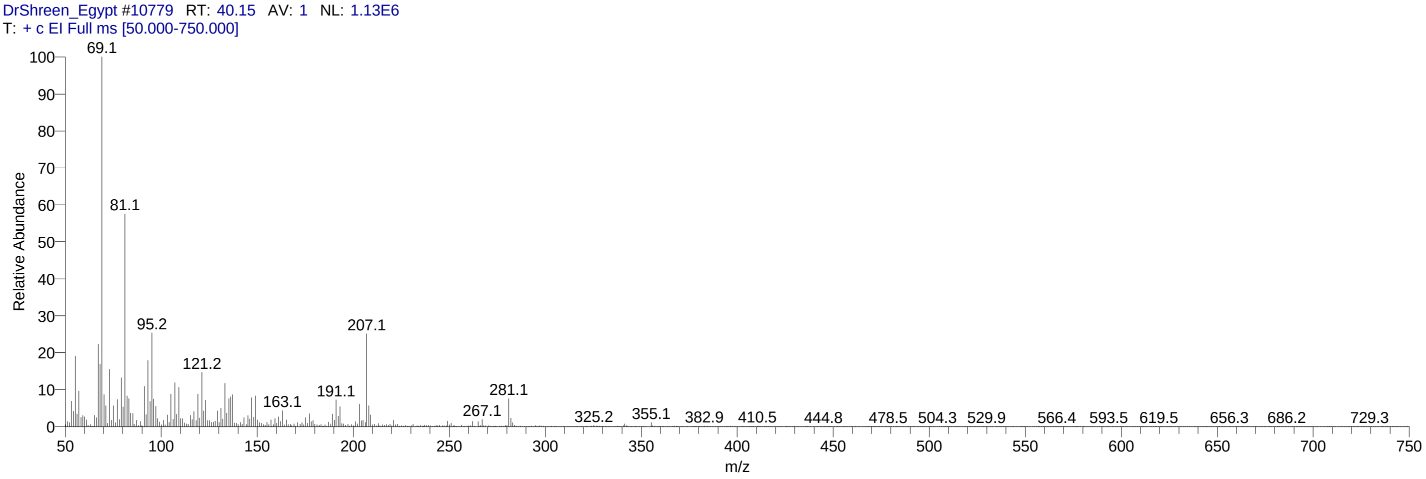

RT	Compound Name	Area %	MF	Molecular Formula	Molecular Weight	Cas #	Library
40.15	1-Heptatriacotanol	0.74	771	C37H76O	536	105794-58-9	mainlib
40.15	LUP-20(29)-ENE-3,28-DIOL, (3á)-	0.74	758	C30H50O2	442	473-98-3	WileyRegistry8e
40.15	á-D-Mannofuranoside, farnesyl-	0.74	759	C21H36O6	384	NA	mainlib
40.15	3,7,11-TRIMETHYL-2,6,10-DODECATRIENYL HEXOFURANOSIDE	0.74	759	C21H36O6	384	NA	WileyRegistry8e
40.15	Oxirane, 2,2-dimethyl-3-(3,7,12,16,20-pentamethyl-3,7,11,15,19-heneicosapentaenyl)-, (all-E)-	0.74	758	C30H50O	426	7200-26-2	replib

Compound Structure

Hit Spectrum

1-Heptatriacotanol
Formula C37H76O, MW 536, CAS# 105794-58-9, Entry# 7279
1-Heptatriacontanol #

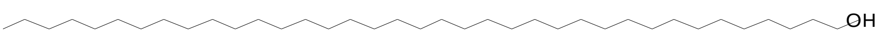

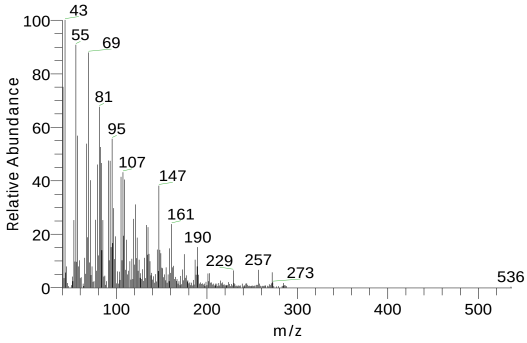

My GC-MS Report

Compound Structure | Hit Spectrum

LUP-20(29)-ENE-3,28-DIOL, (3á)-
Formula C30H50O2, MW 442, CAS# 473-98-3, Entry# 349524
LUP-20(29)-ENE-3,28-DIOL #

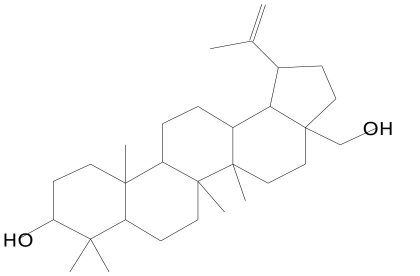

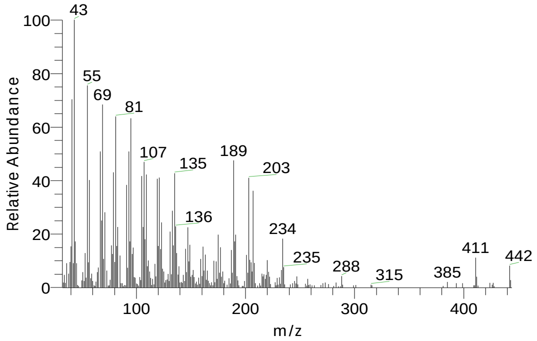

á-D-Mannofuranoside, farnesyl-
Formula C21H36O6, MW 384, CAS# NA, Entry# 34325
(2E,6Z)-3,7,11-Trimethyl-2,6,10-dodecatrienyl hexofuranoside #

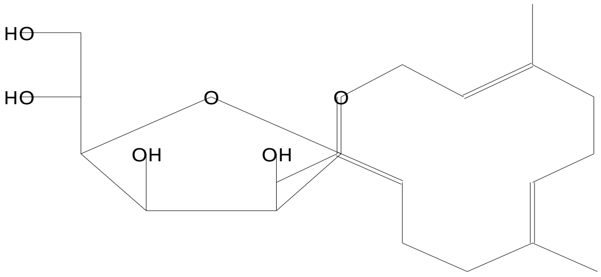

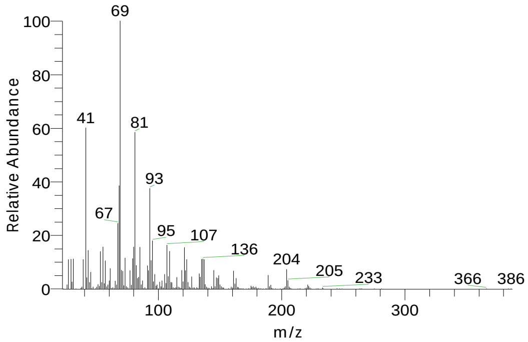

3,7,11-TRIMETHYL-2,6,10-DODECATRIENYL HEXOFURANOSIDE
Formula C21H36O6, MW 384, CAS# NA, Entry# 389191
BETA-D-MANNOFURANOSID, FARNESOL-

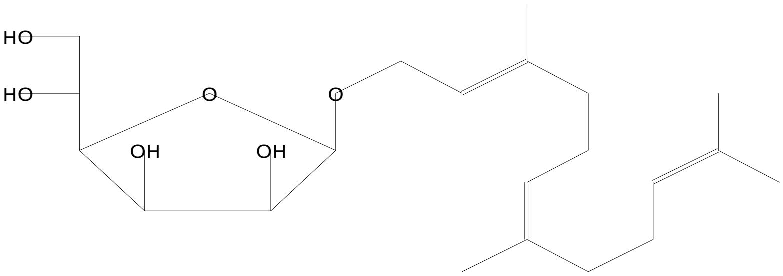

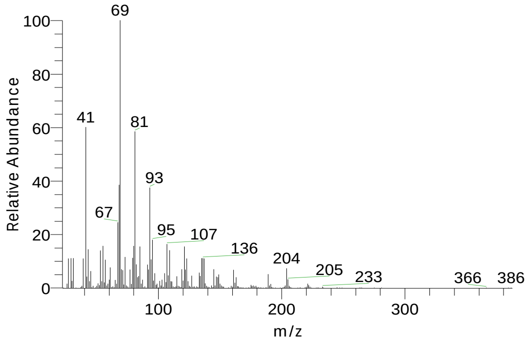

Formula C30H50O, MW 426, CAS# 7200-26-2, Entry# 8712

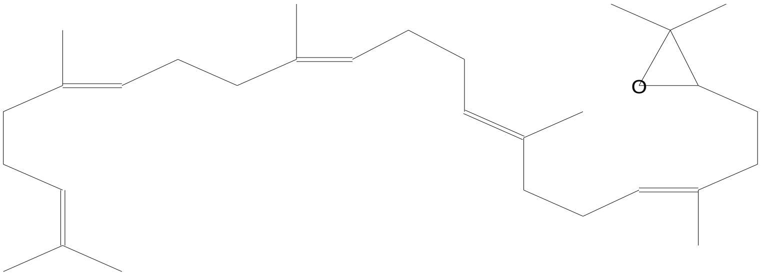

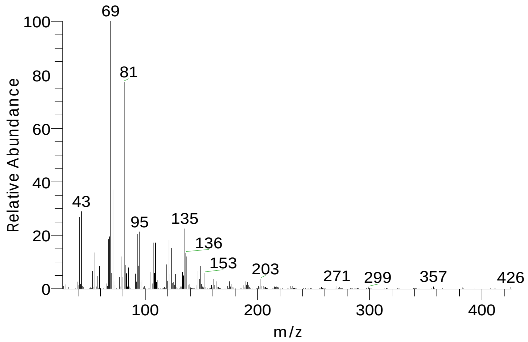

My GC-MS Report

DrShreen_Egypt #11042 RT: 41.03 AV: 1 NL: 5.62E5
T: + c EI Full ms [50.000-750.000]

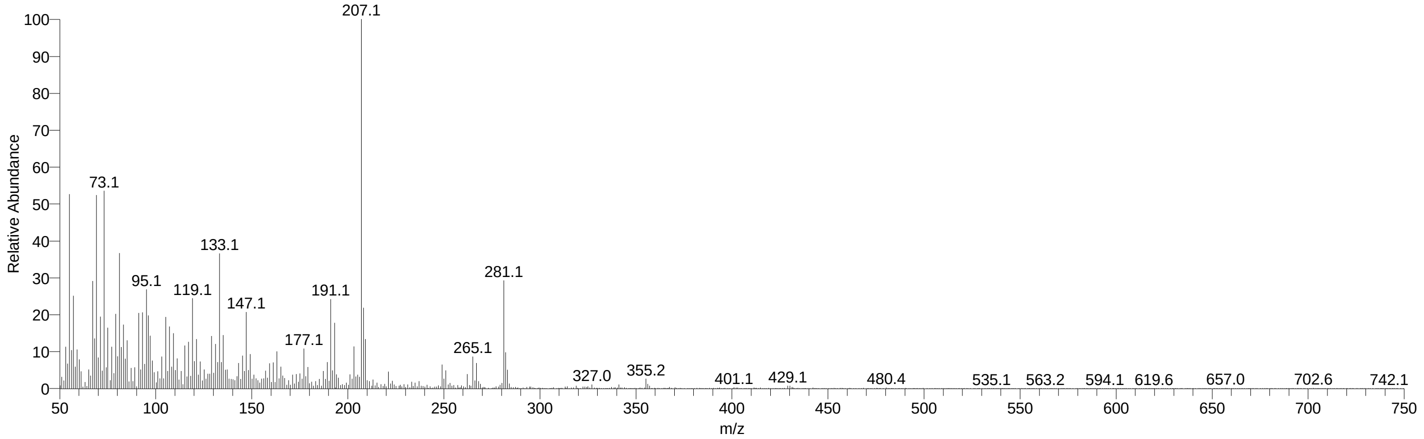

RT	Compound Name	Area %	MF	Molecular Formula	Molecular Weight	Cas #	Library
41.03	2,2-Dimethyl-6-methylene-1-[3,5-dihydroxy-1-pentenyl]cyclohexan-1-perhydrol	0.31	730	C14H24O4	256	NA	mainlib
41.03	ARABINITOL, PENTAACETATE	0.31	740	C15H22O10	362	26674-23-7	WileyRegistry8e
41.03	.psi.,.psi.-Carotene, 1,1',2,2'-tetrahydro-1,1'-dimethoxy-	0.31	654	C42H64O2	600	13833-01-7	mainlib
41.03	.PSI.,.PSI.-CAROTENE, 1,1',2,2'-TETRAHYDRO-1,1'-DIMETHOXY-	0.31	653	C42H64O2	600	13833-01-7	WileyRegistry8e
41.03	SILANE, TRIMETHYL[[(3á)-STIGMAST-5-EN-3-YL]OXY]-	0.31	685	C32H58OSi	486	2625-46-9	WileyRegistry8e

Compound Structure | Hit Spectrum

2,2-Dimethyl-6-methylene-1-[3,5-dihydroxy-1-pentenyl]cyclohexan-1-perhydrol
Formula C14H24O4, MW 256, CAS# NA, Entry# 34938
(4E)-5-(1-Hydroperoxy-2,2-dimethyl-6-methylenecyclohexyl)-4-pentene-1,3-diol #

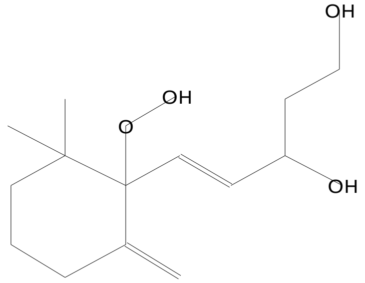

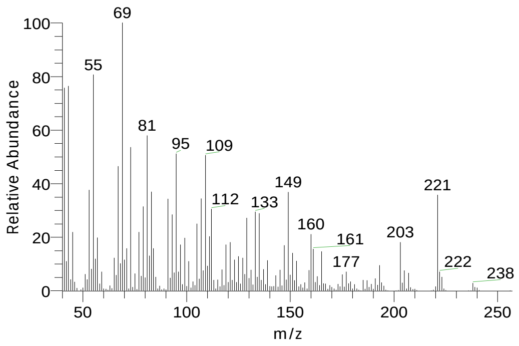

ARABINITOL, PENTAACETATE
Formula C15H22O10, MW 362, CAS# 26674-23-7, Entry# 235892
1,2,3,4,5-PENTA-O-ACETYLPENTITOL #

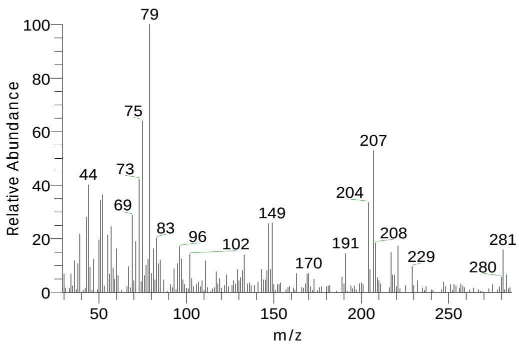

My GC-MS Report

Compound Structure | Hit Spectrum

.psi.,.psi.-Carotene, 1,1',2,2'-tetrahydro-1,1'-dimethoxy-
Formula C42H64O2, MW 600, CAS# 13833-01-7, Entry# 41205
Lycopene, 1,1',2,2'-tetrahydro-1,1'-dimethoxy-, all-trans-

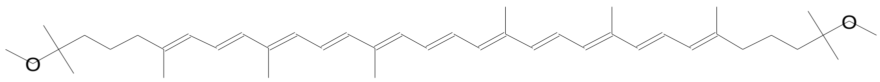

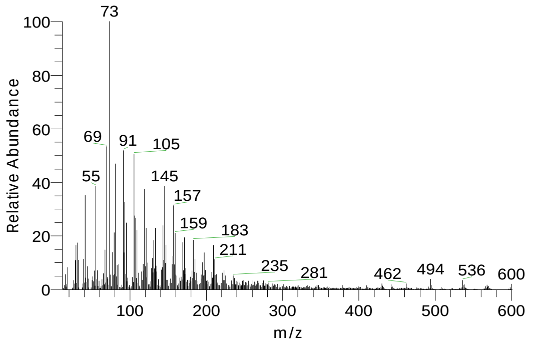

.PSI.,.PSI.-CAROTENE, 1,1',2,2'-TETRAHYDRO-1,1'-DIMETHOXY-
Formula C42H64O2, MW 600, CAS# 13833-01-7, Entry# 296796
1,1',2,2'-TETRAHYDRO-PSI,PSI-CAROTENE #

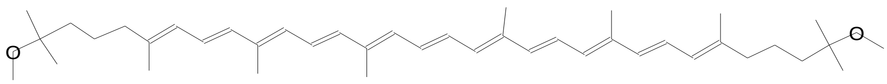

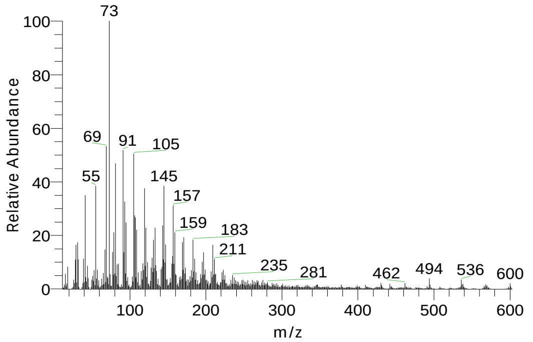

SILANE, TRIMETHYL[[(3á)-STIGMAST-5-EN-3-YL]OXY]-
Formula C32H58OSi, MW 486, CAS# 2625-46-9, Entry# 283210
3-[(TRIMETHYLSILYL)OXY]STIGMAST-5-ENE #

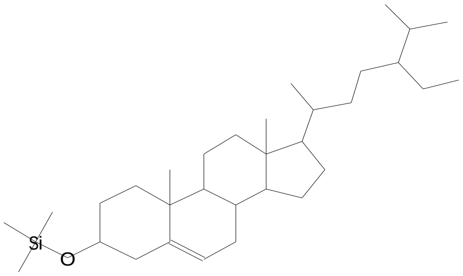

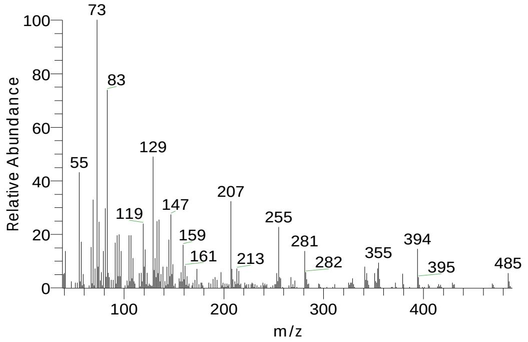

DrShreen_Egypt #11206 RT: 41.58 AV: 1 NL: 6.34E5
T: + c EI Full ms [50.000-750.000]

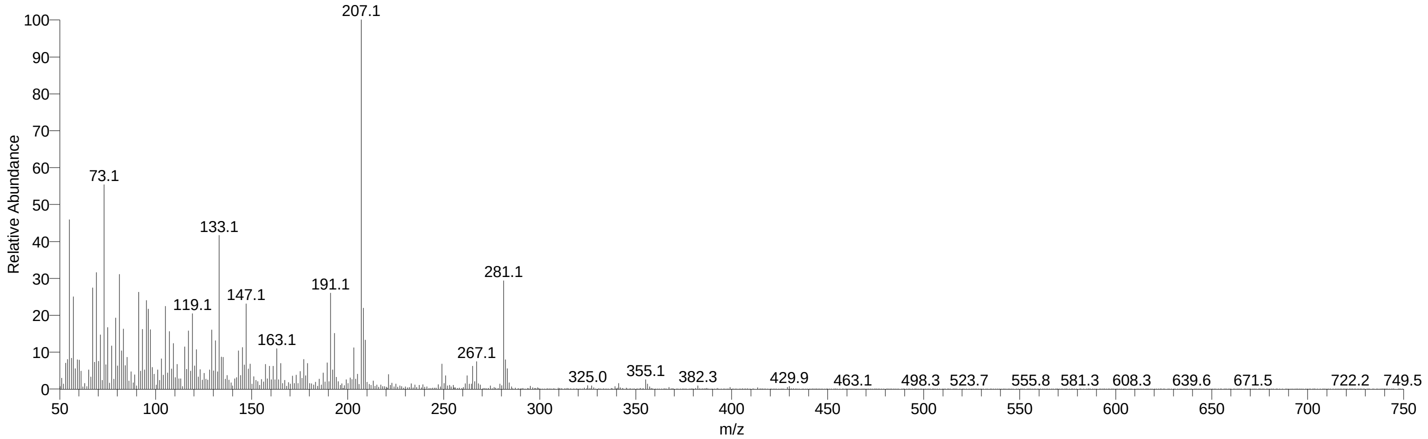

RT	Compound Name	Area %	MF	Molecular Formula	Molecular Weight	Cas #	Library
41.58	.psi.,.psi.-Carotene, 1,1',2,2'-tetrahydro-1,1'-dimethoxy-	0.37	654	C42H64O2	600	13833-01-7	mainlib
41.58	ARABINITOL, PENTAACETATE	0.37	733	C15H22O10	362	26674-23-7	WileyRegistry8e

My GC-MS Report

RT	Compound Name	Area %	MF	Molecular Formula	Molecular Weight	Cas #	Library
41.58	.PSI.,.PSI.-CAROTENE, 1,1',2,2'-TETRAHYDRO-1,1'-DIMETHOXY-	0.37	653	C42H64O2	600	13833-01-7	WileyRegistry8e
41.58	2,2-Dimethyl-6-methylene-1-[3,5-dihydroxy-1-pentenyl]cyclohexan-1-perhydrol	0.37	717	C14H24O4	256	NA	mainlib
41.58	SILANE, TRIMETHYL[[(3á)-STIGMAST-5-EN-3-YL]OXY]-	0.37	679	C32H58OSi	486	2625-46-9	WileyRegistry8e

Compound Structure | Hit Spectrum

.psi.,.psi.-Carotene, 1,1',2,2'-tetrahydro-1,1'-dimethoxy-
Formula C42H64O2, MW 600, CAS# 13833-01-7, Entry# 41205
Lycopene, 1,1',2,2'-tetrahydro-1,1'-dimethoxy-, all-trans-

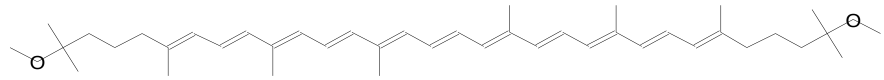

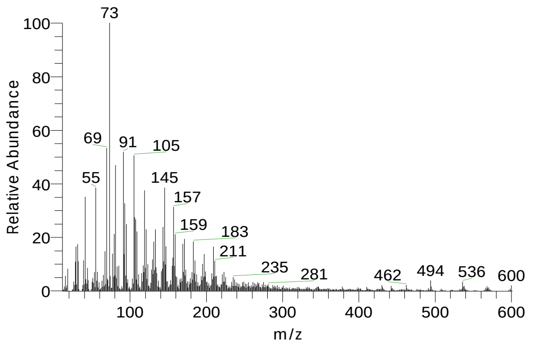

ARABINITOL, PENTAACETATE
Formula C15H22O10, MW 362, CAS# 26674-23-7, Entry# 235892
1,2,3,4,5-PENTA-O-ACETYLPENTITOL #

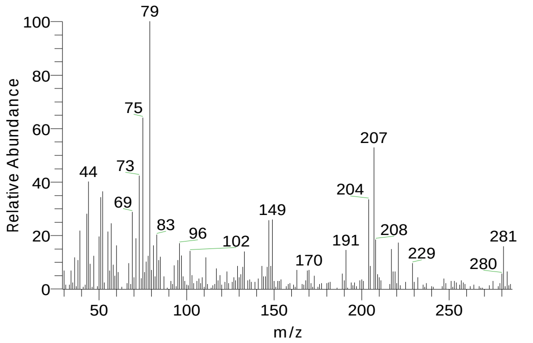

.PSI.,.PSI.-CAROTENE, 1,1',2,2'-TETRAHYDRO-1,1'-DIMETHOXY-
Formula C42H64O2, MW 600, CAS# 13833-01-7, Entry# 296796
1,1',2,2'-TETRAHYDRO-PSI,PSI-CAROTENE #

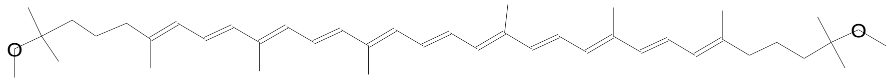

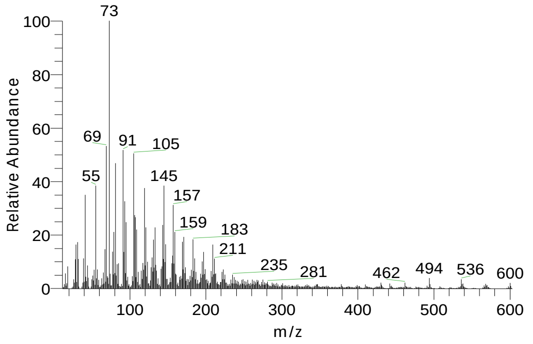

2,2-Dimethyl-6-methylene-1-[3,5-dihydroxy-1-pentenyl]cyclohexan-1-perhydrol
Formula C14H24O4, MW 256, CAS# NA, Entry# 34938
(4E)-5-(1-Hydroperoxy-2,2-dimethyl-6-methylenecyclohexyl)-4-pentene-1,3-diol #

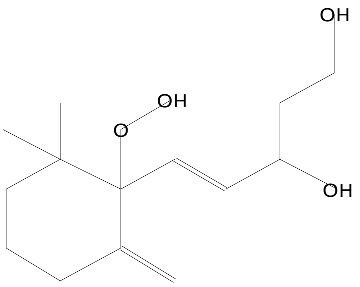

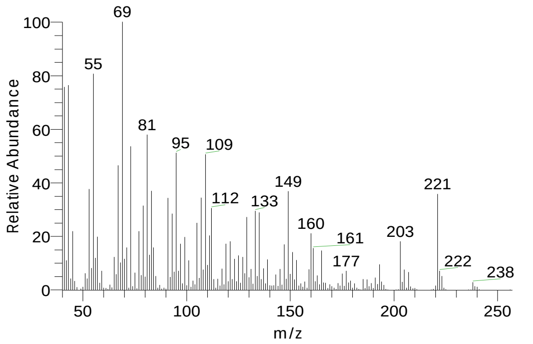

My GC-MS Report

Compound Structure | Hit Spectrum

SILANE, TRIMETHYL[[(3á)-STIGMAST-5-EN-3-YL]OXY]-
Formula C32H58OSi, MW 486, CAS# 2625-46-9, Entry# 283210
3-[(TRIMETHYLSILYL)OXY]STIGMAST-5-ENE #

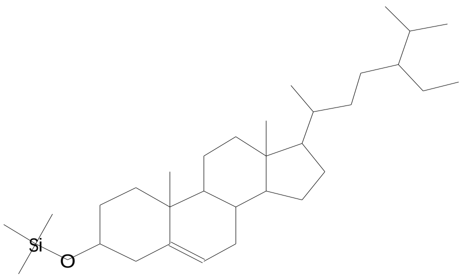

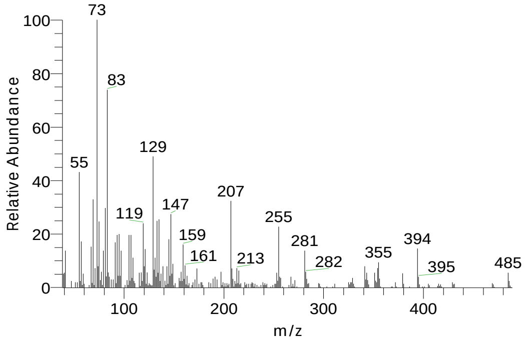

DrShreen_Egypt #11491 RT: 42.53 AV: 1 NL: 6.63E5
T: + c EI Full ms [50.000-750.000]

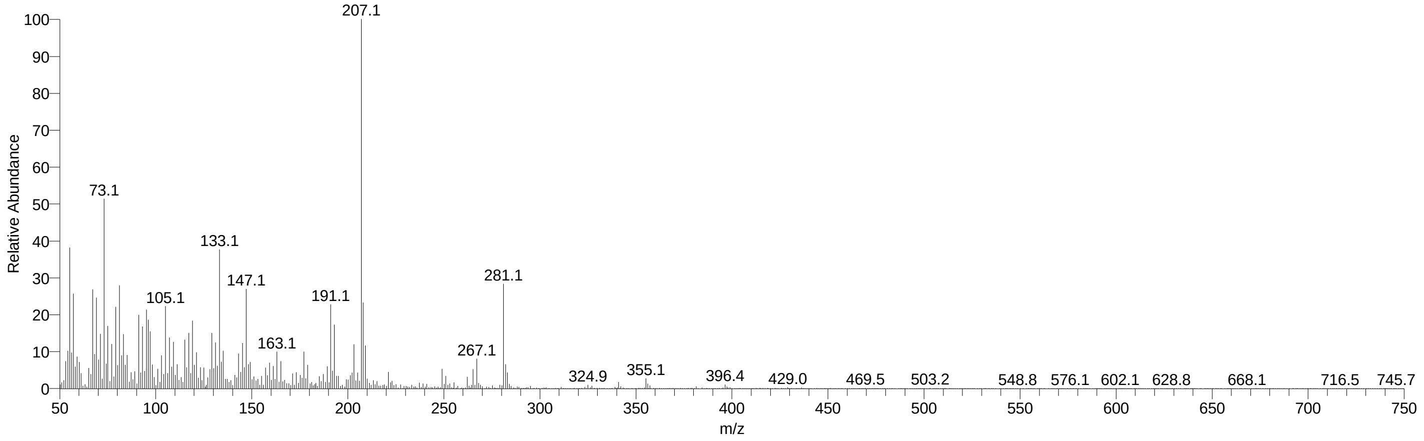

RT	Compound Name	Area %	MF	Molecular Formula	Molecular Weight	Cas #	Library
42.53	9,12-OCTADECADIENOIC ACID (Z,Z)-, 2,3-BIS[(TRIMETHYLSILYL)OXY]PROPYL ESTER	0.31	758	C27H54O4Si2	498	54284-45-6	WileyRegistry8e
42.53	à-D-GALACTOPYRANOSIDE, METHYL 2,3-BIS-O-(TRIMETHYLSILYL)-, CYCLIC BUTYLBORONATE	0.31	674	C17H37BO6Si2	404	56211-11-1	WileyRegistry8e
42.53	1,8-Dioxa-5-thiaoctane, 8-(9-borabicyclo[3.3.1]non-9-yl)-3-(9-borabicyclo[3.3.1]non-9-yloxy)-1-phenyl-	0.31	668	C27H42B2O3S	468	NA	mainlib
42.53	1,8-DIOXA-5-THIAOCTAN, 8-(9-BORABICYCLO[3.3.1]NON-9-YL)-3-(9-BORABICYCLO[3.3.1]NON-9-YLOXY)-1-PHENYL-	0.31	668	C27H42B2O3S	468	NA	WileyRegistry8e
42.53	ARABINITOL, PENTAACETATE	0.31	727	C15H22O10	362	26674-23-7	WileyRegistry8e

My GC-MS Report

Compound Structure | Hit Spectrum

9,12-OCTADECADIENOIC ACID (Z,Z)-, 2,3-BIS[(TRIMETHYLSILYL)OXY]PROPYL ESTER
Formula C27H54O4Si2, MW 498, CAS# 54284-45-6, Entry# 285148
2,3-BIS[(TRIMETHYLSILYL)OXY]PROPYL (9Z,12Z)-9,12-OCTADECADIENOATE #

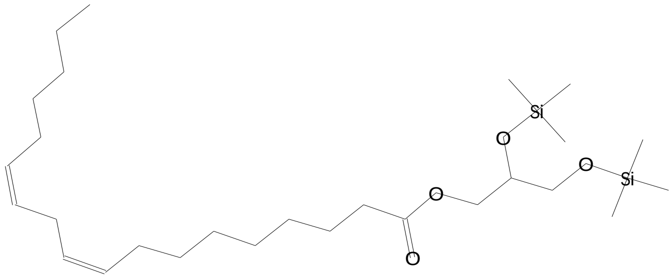

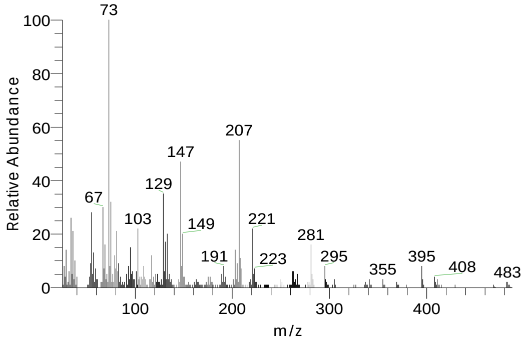

à-D-GALACTOPYRANOSIDE, METHYL 2,3-BIS-O-(TRIMETHYLSILYL)-, CYCLIC BUTYLBORONATE
Formula C17H37BO6Si2, MW 404, CAS# 56211-11-1, Entry# 257663

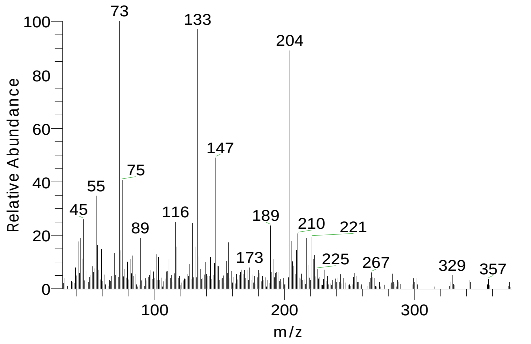

Formula C27H42B2O3S, MW 468, CAS# NA, Entry# 68432

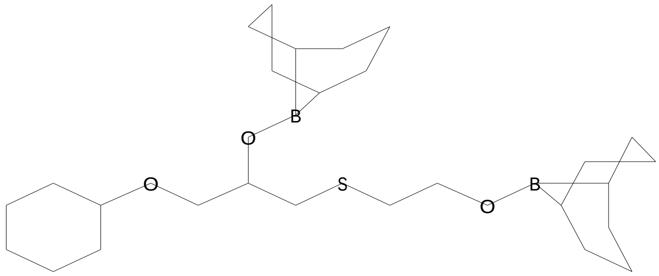

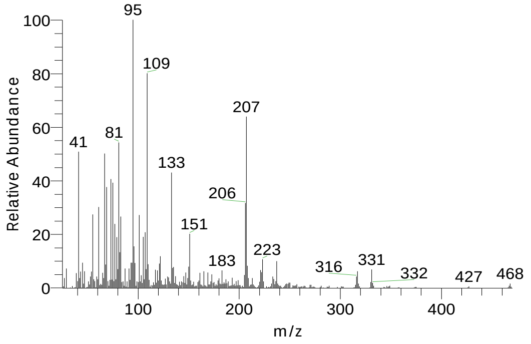

Formula C27H42B2O3S, MW 468, CAS# NA, Entry# 378917

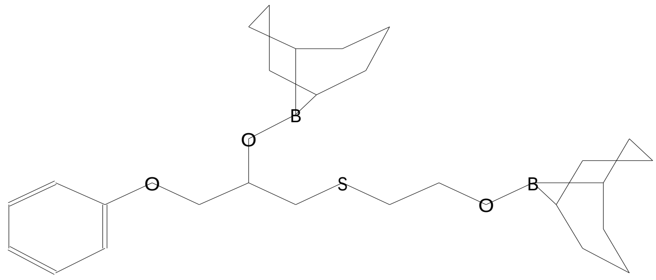

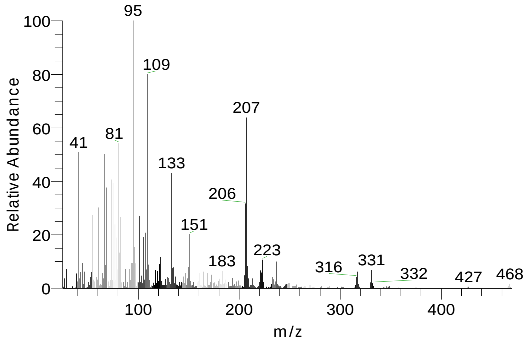

My GC-MS Report

Compound Structure | Hit Spectrum

ARABINITOL, PENTAACETATE
Formula C15H22O10, MW 362, CAS# 26674-23-7, Entry# 235892
1,2,3,4,5-PENTA-O-ACETYLPENTITOL #

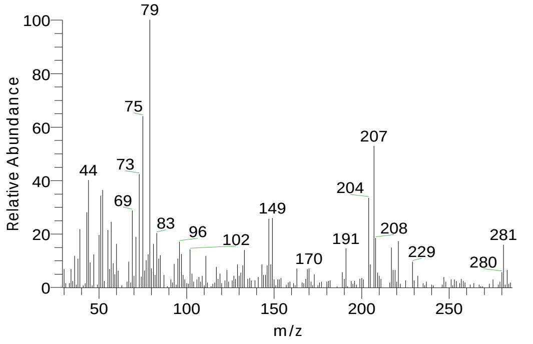

DrShreen_Egypt #11531 RT: 42.67 AV: 1 NL: 7.74E5
T: + c EI Full ms [50.000-750.000]

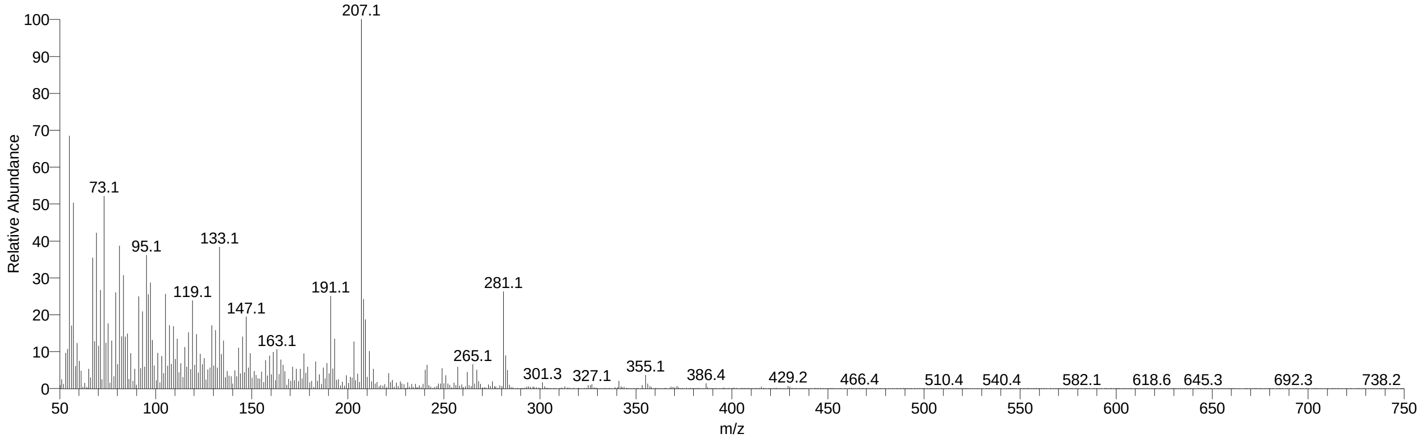

RT	Compound Name	Area %	MF	Molecular Formula	Molecular Weight	Cas #	Library
42.67	Ethyl iso-allocholate	1.64	718	C26H44O5	436	NA	mainlib
42.67	ETHYL ISO-ALLOCHOLATE	1.64	718	C26H44O5	436	NA	WileyRegistry8e
42.67	1-Heptatriacotanol	1.64	744	C37H76O	536	105794-58-9	mainlib
42.67	4H-1-BENZOPYRAN-4-ONE, 2-(3,4-DIMETHOXYPHENYL)-3,5-DIHYDROXY-7-METHOXY-	1.64	710	C18H16O7	344	6068-80-0	WileyRegistry8e
42.67	01297107001 TETRANEURIN - A - DIOL	1.64	727	C15H20O5	280	NA	WileyRegistry8e

Compound Structure | Hit Spectrum

Ethyl iso-allocholate
Formula C26H44O5, MW 436, CAS# NA, Entry# 7020
Ethyl 3,7,12-trihydroxycholan-24-oate #

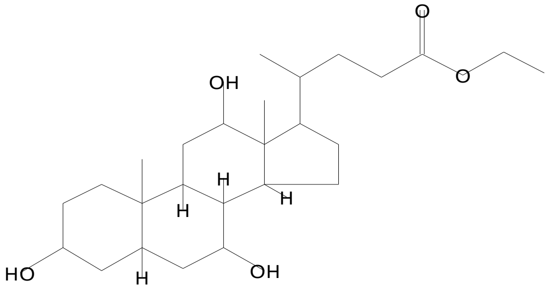

SI 717, RSI 718, mainlib, Entry# 7020, CAS# NA, Ethyl iso-allocholate

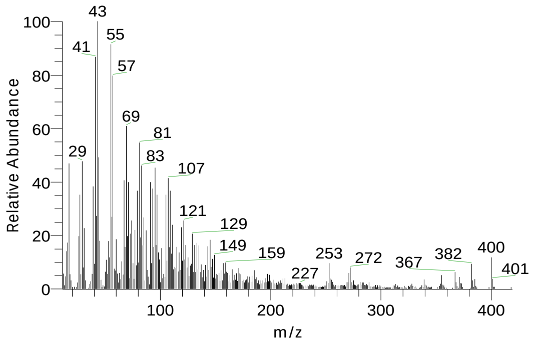

My GC-MS Report

Compound Structure | Hit Spectrum

ETHYL ISO-ALLOCHOLATE
Formula C26H44O5, MW 436, CAS# NA, Entry# 270212

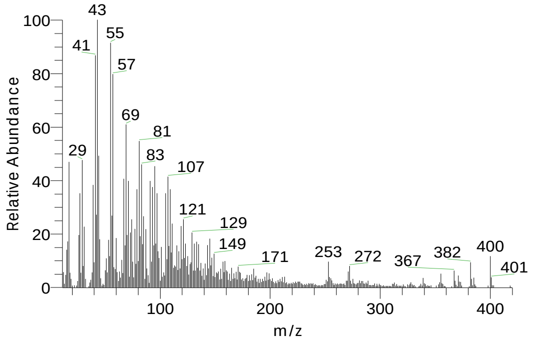

1-Heptatriacotanol
Formula C37H76O, MW 536, CAS# 105794-58-9, Entry# 7279
1-Heptatriacontanol #

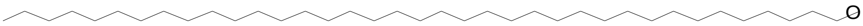

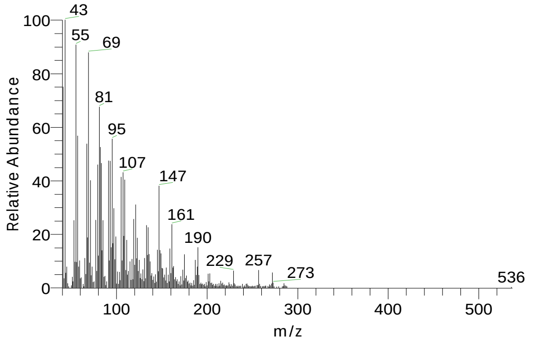

4H-1-BENZOPYRAN-4-ONE, 2-(3,4-DIMETHOXYPHENYL)-3,5-DIHYDROXY-7-METHOXY-
Formula C18H16O7, MW 344, CAS# 6068-80-0, Entry# 224392
3',4',7-TRIMETHYLQUERCETIN

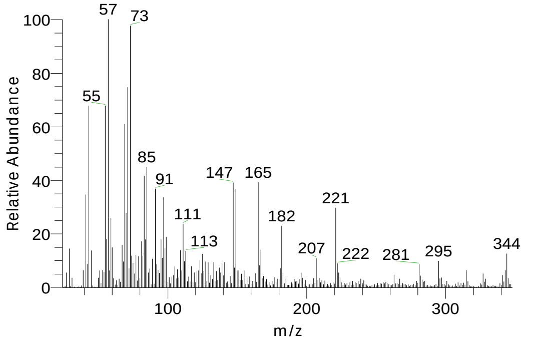

01297107001 TETRANEURIN - A - DIOL
Formula C15H20O5, MW 280, CAS# NA, Entry# 170378

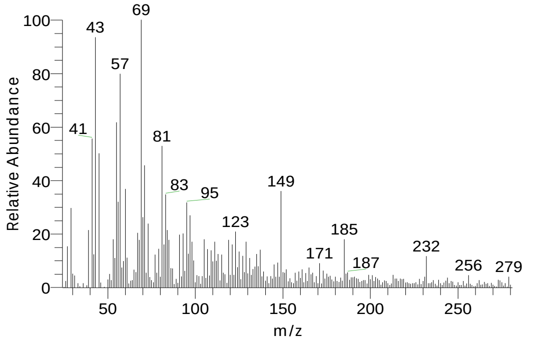

My GC-MS Report

DrShreen_Egypt #11914 RT: 43.95 AV: 1 NL: 2.93E6
T: + c EI Full ms [50.000-750.000]

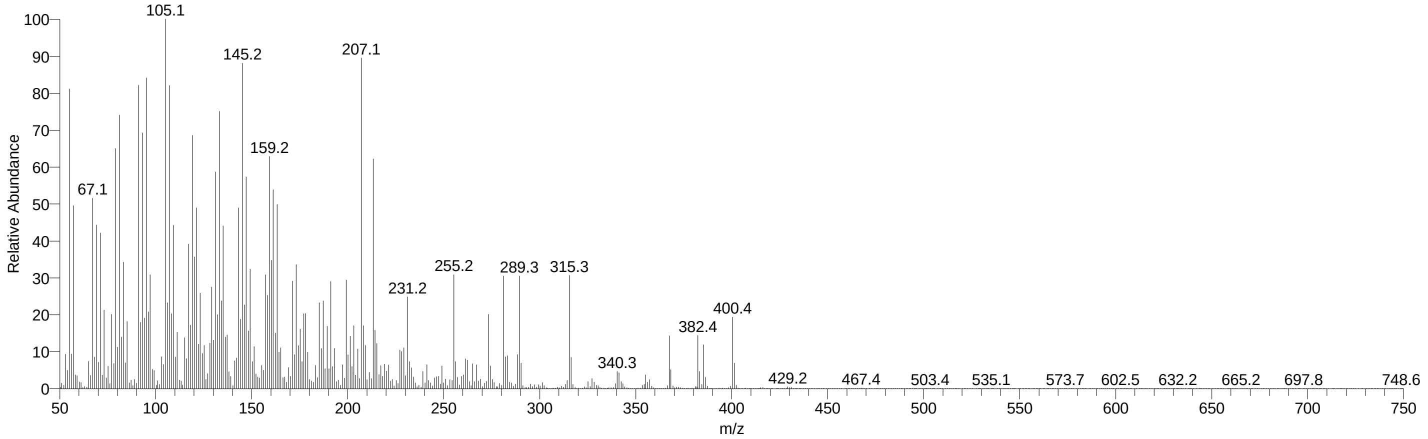

RT	Compound Name	Area %	MF	Molecular Formula	Molecular Weight	Cas #	Library
43.95	(E)-5,10-SECOCHOLEST-1(10)-EN -3,5-DIONE	28.70	922	C27H44O2	400	NA	WileyRegistry8e
43.95	Campesterol	28.70	868	C28H48O	400	474-62-4	mainlib
43.95	ERGOST-5-EN-3-OL	28.70	868	C28H48O	400	NA	WileyRegistry8e
43.95	ERGOST-5-EN-3-OL, (3á)-	28.70	865	C28H48O	400	4651-51-8	WileyRegistry8e
43.95	19-METHYLENE-5,10-SECOCHOLESTAN-3,5-DIONE	28.70	874	C27H44O2	400	NA	WileyRegistry8e

Compound Structure	Hit Spectrum

(E)-5,10-SECOCHOLEST-1(10)-EN-3,5-DIONE
Formula C27H44O2, MW 400, CAS# NA, Entry# 256333

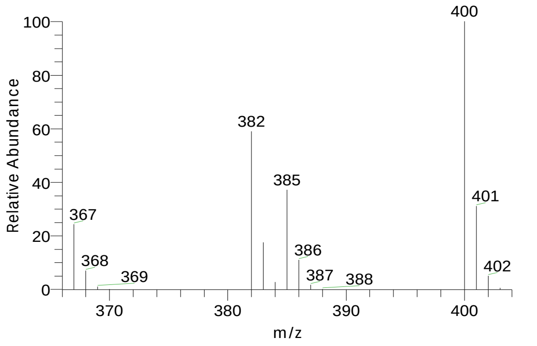

Campesterol
Formula C28H48O, MW 400, CAS# 474-62-4, Entry# 7079
Ergost-5-en-3-ol, (3á,24R)-

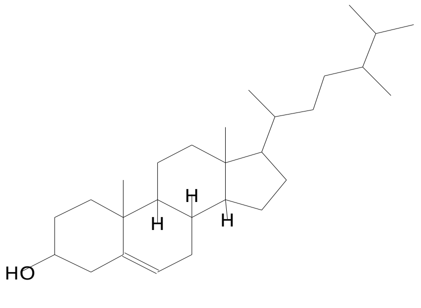

SI 861, RSI 868, mainlib, Entry# 7079, CAS# 474-62-4, Campesterol

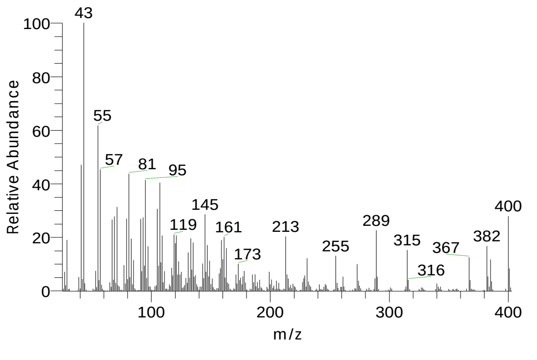

My GC-MS Report

Compound Structure | Hit Spectrum

ERGOST-5-EN-3-OL
Formula C28H48O, MW 400, CAS# NA, Entry# 387447
ERGOST-5-EN-3B-OL

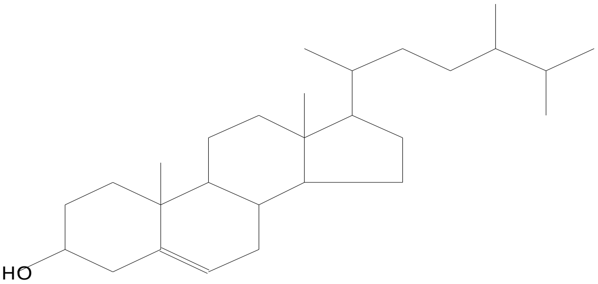

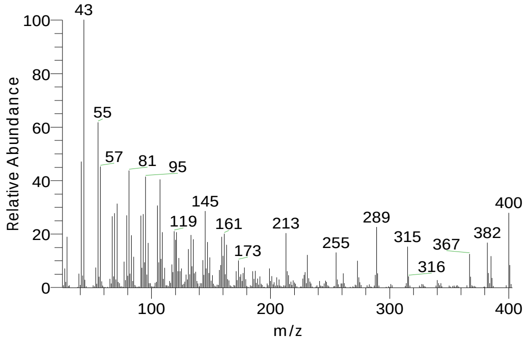

ERGOST-5-EN-3-OL, (3á)-
Formula C28H48O, MW 400, CAS# 4651-51-8, Entry# 256351
ERGOST-5-EN-3-OL #

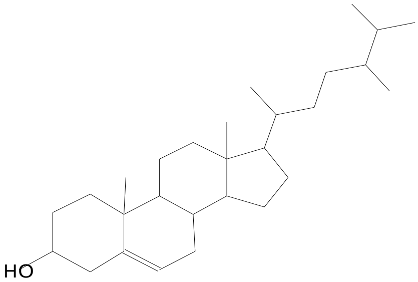

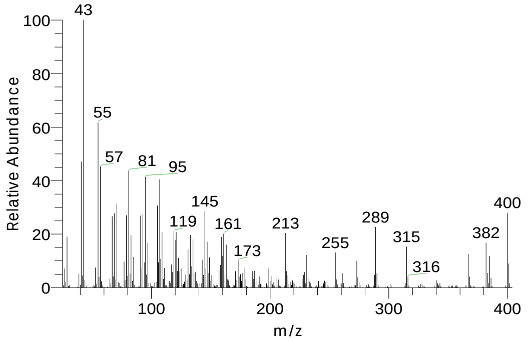

19-METHYLENE-5,10-SECOCHOLESTAN-3,5-DIONE
Formula C27H44O2, MW 400, CAS# NA, Entry# 256331

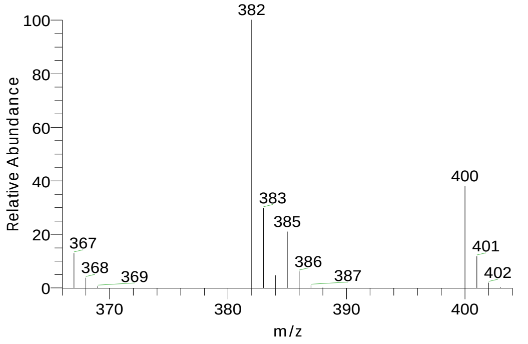

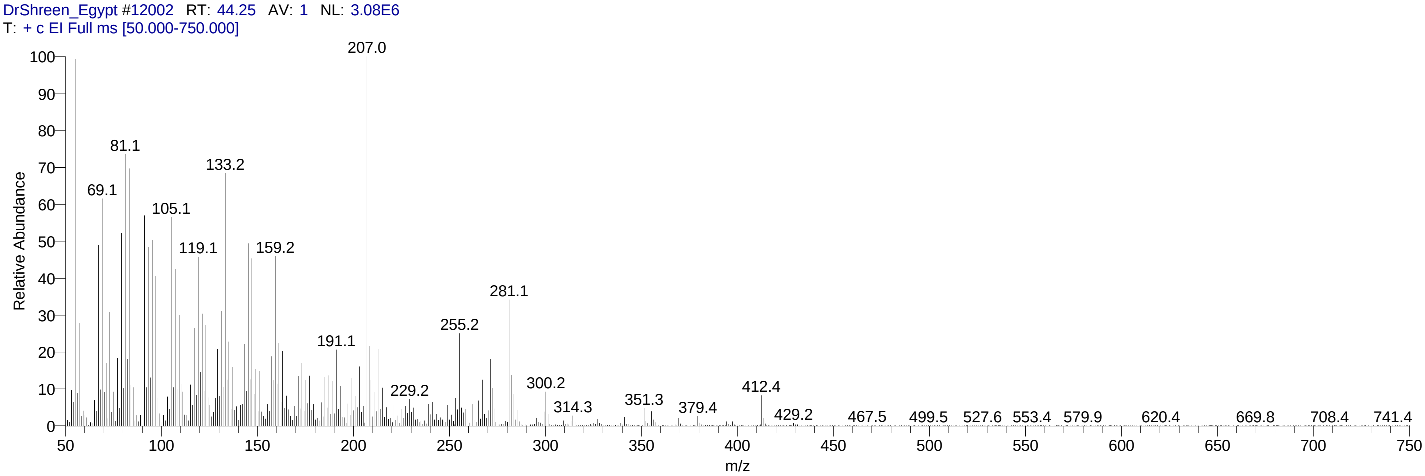

RT	Compound Name	Area %	MF	Molecular Formula	Molecular Weight	Cas #	Library
44.25	Stigmasterol	16.11	867	C29H48O	412	83-48-7	mainlib
44.25	STIGMASTA-5,22-DIEN-3-OL, (3á,22E)-	16.11	820	C29H48O	412	83-48-7	WileyRegistry8e
44.25	Stigmasterol	16.11	806	C29H48O	412	83-48-7	replib

My GC-MS Report

RT	Compound Name	Area %	MF	Molecular Formula	Molecular Weight	Cas #	Library
44.25	STIGMASTA-5,22-DIEN-3-OL	16.11	836	C29H48O	412	NA	WileyRegistry8e
44.25	Cholesta-22,24-dien-5-ol, 4,4-dimethyl-	16.11	826	C29H48O	412	NA	mainlib

Compound Structure | Hit Spectrum

Stigmasterol
Formula C29H48O, MW 412, CAS# 83-48-7, Entry# 20820
Stigmasta-5,22-dien-3-ol, (3á,22E)-

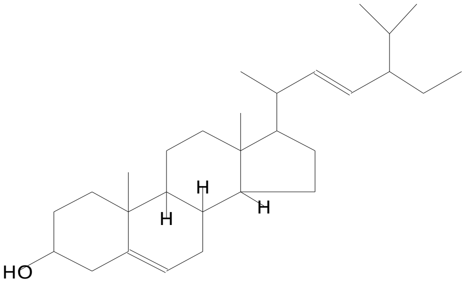

SI 819, RSI 867, mainlib, Entry# 20820, CAS# 83-48-7, Stigmasterol

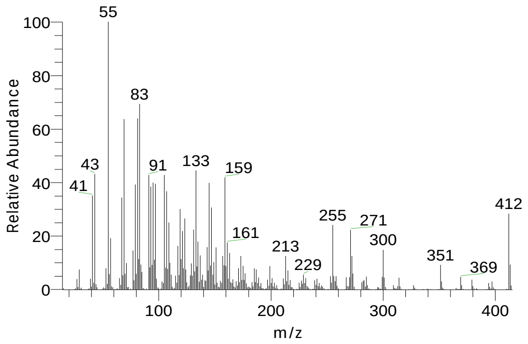

STIGMASTA-5,22-DIEN-3-OL, (3á,22E)-
Formula C29H48O, MW 412, CAS# 83-48-7, Entry# 261297
(22E)-STIGMASTA-5,22-DIEN-3-OL #

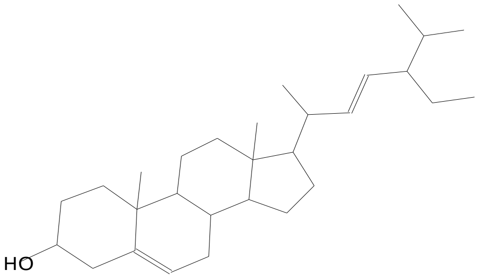

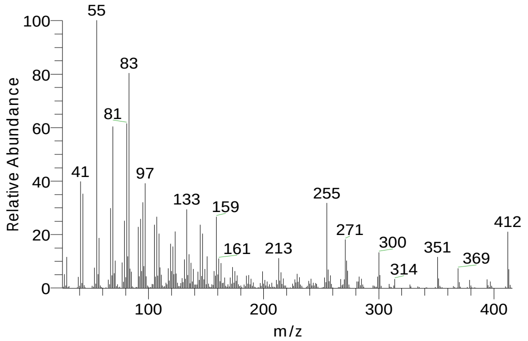

Stigmasterol
Formula C29H48O, MW 412, CAS# 83-48-7, Entry# 5167
Stigmasta-5,22-dien-3-ol, (3á,22E)-

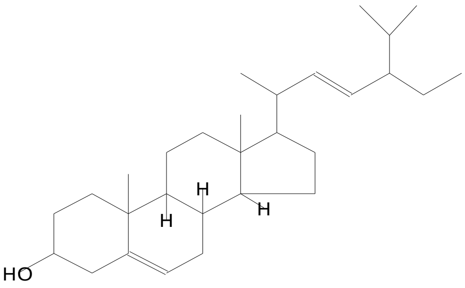

SI 805, RSI 806, replib, Entry# 5167, CAS# 83-48-7, Stigmasterol

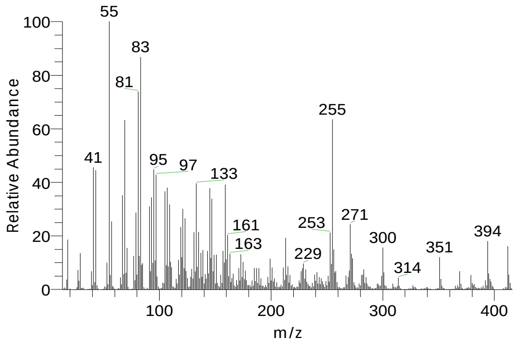

STIGMASTA-5,22-DIEN-3-OL
Formula C29H48O, MW 412, CAS# NA, Entry# 383444
STIGMASTA-5,22E-DIEN-3B-OL

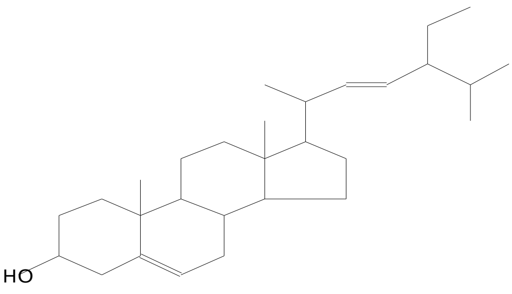

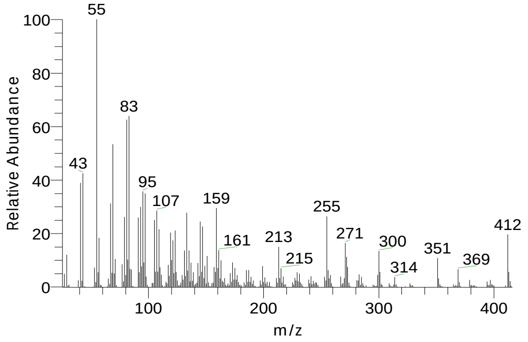

My GC-MS Report

Compound Structure | Hit Spectrum

Cholesta-22,24-dien-5-ol, 4,4-dimethyl-
Formula C29H48O, MW 412, CAS# NA, Entry# 19679
(22E)-4,4-Dimethylcholesta-22,24-dien-6-ol #

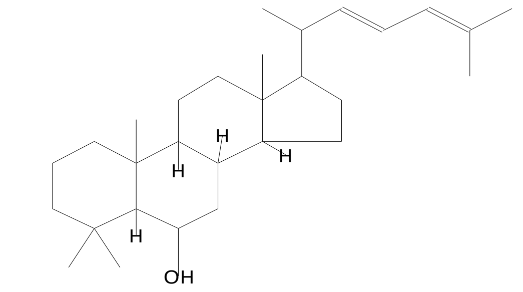

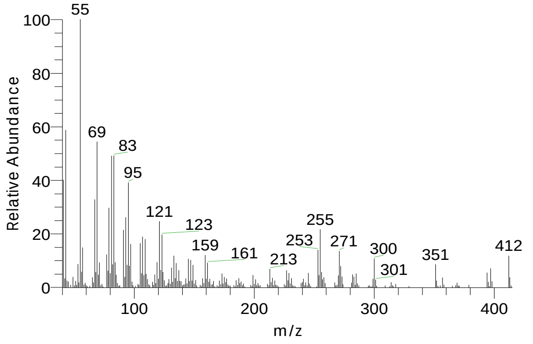

DrShreen_Egypt #12159 RT: 44.77 AV: 1 NL: 5.36E6
T: + c EI Full ms [50.000-750.000]

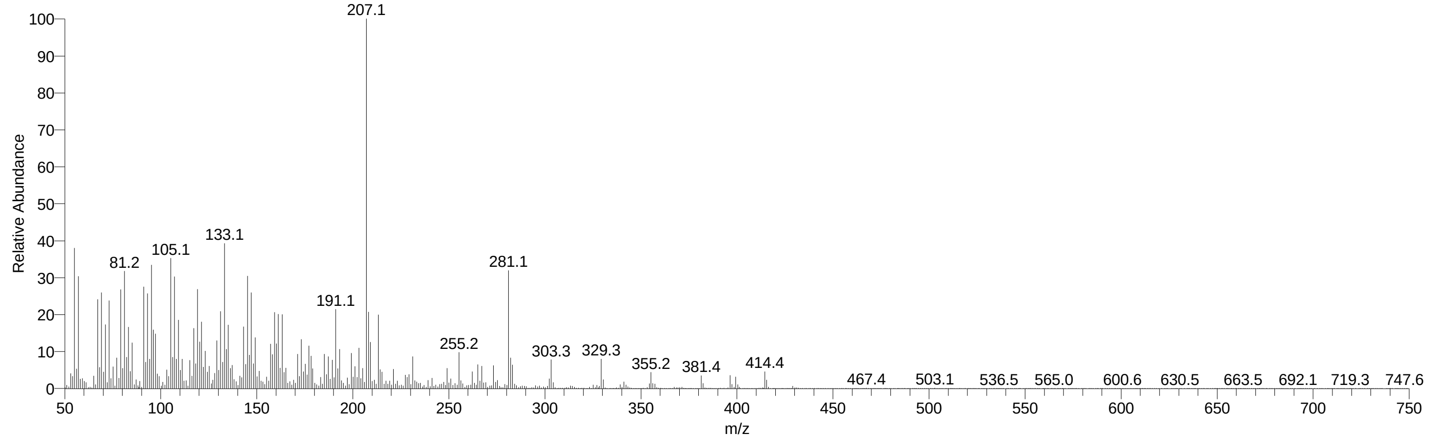

RT	Compound Name	Area %	MF	Molecular Formula	Molecular Weight	Cas #	Library
44.77	á-Sitosterol	17.66	823	C29H50O	414	83-46-5	replib
44.77	ç-Sitosterol	17.66	787	C29H50O	414	83-47-6	mainlib
44.77	STIGMAST-5-EN-3-OL, (3á,24S)-	17.66	787	C29H50O	414	83-47-6	WileyRegistry8e
44.77	STIGMAST-5-EN-3-OL, (3á,24S)-	17.66	783	C29H50O	414	83-47-6	WileyRegistry8e
44.77	ç-Sitosterol	17.66	824	C29H50O	414	83-47-6	replib

Compound Structure | Hit Spectrum

á-Sitosterol
Formula C29H50O, MW 414, CAS# 83-46-5, Entry# 2073
Stigmast-5-en-3-ol, (3á)-

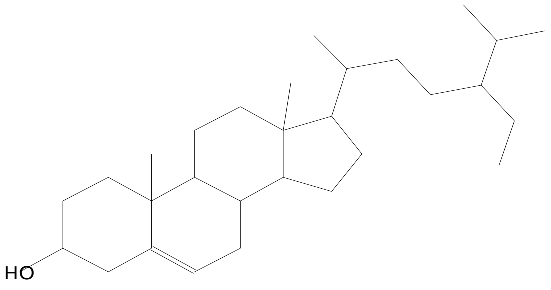

SI 776, RSI 823, replib, Entry# 2073, CAS# 83-46-5, á-Sitosterol

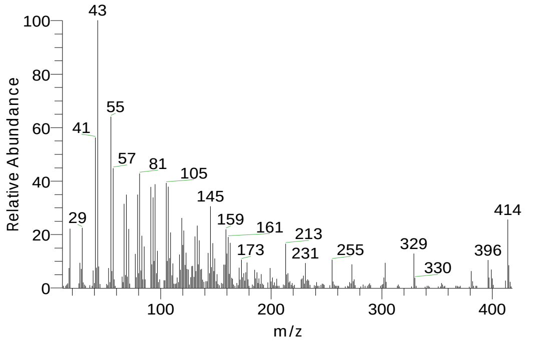

My GC-MS Report

Compound Structure | Hit Spectrum

ç-Sitosterol
Formula C29H50O, MW 414, CAS# 83-47-6, Entry# 7212
Stigmast-5-en-3-ol, (3á,24S)-

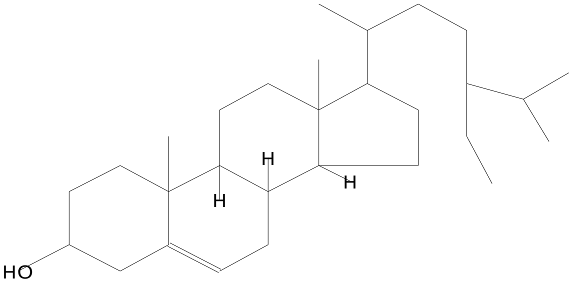

SI 776, RSI 787, mainlib, Entry# 7212, CAS# 83-47-6, ç-Sitosterol

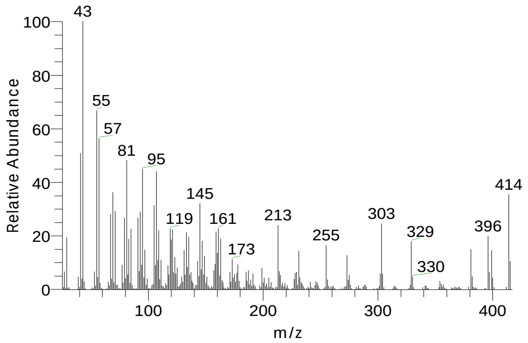

STIGMAST-5-EN-3-OL, (3á,24S)-
Formula C29H50O, MW 414, CAS# 83-47-6, Entry# 387446
STIGMAST-5-EN-3-OL #

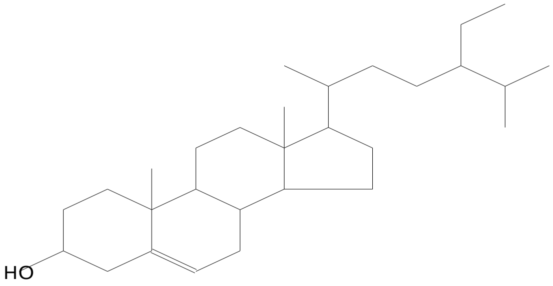

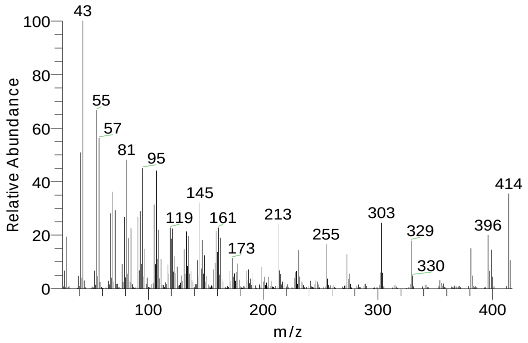

STIGMAST-5-EN-3-OL, (3á,24S)-
Formula C29H50O, MW 414, CAS# 83-47-6, Entry# 262357
STIGMAST-5-EN-3-OL #

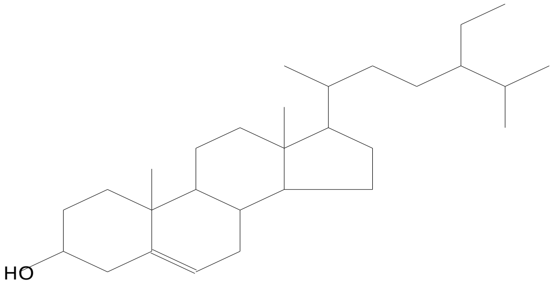

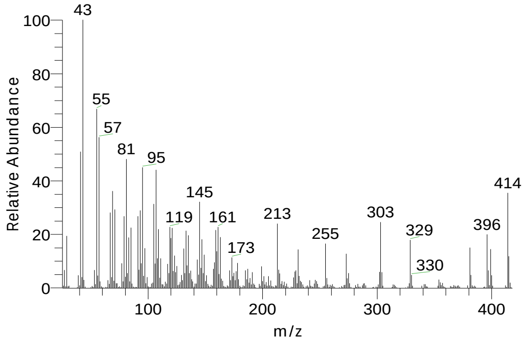

ç-Sitosterol
Formula C29H50O, MW 414, CAS# 83-47-6, Entry# 33634
Stigmast-5-en-3-ol, (3á,24S)-

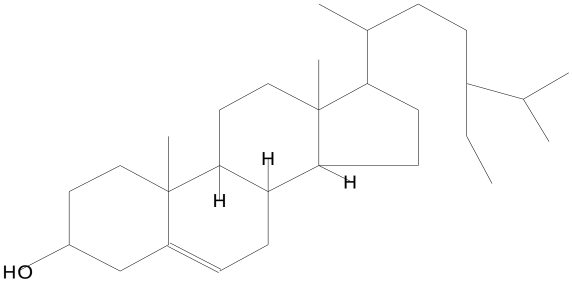

SI 757, RSI 824, replib, Entry# 33634, CAS# 83-47-6, ç-Sitosterol

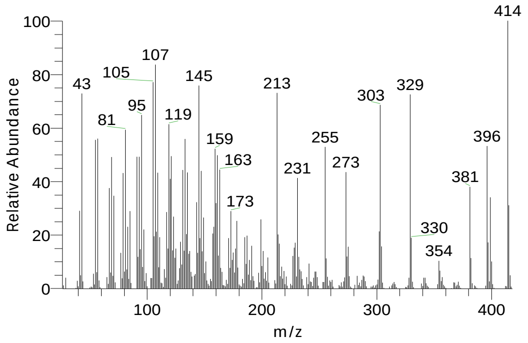

My GC-MS Report

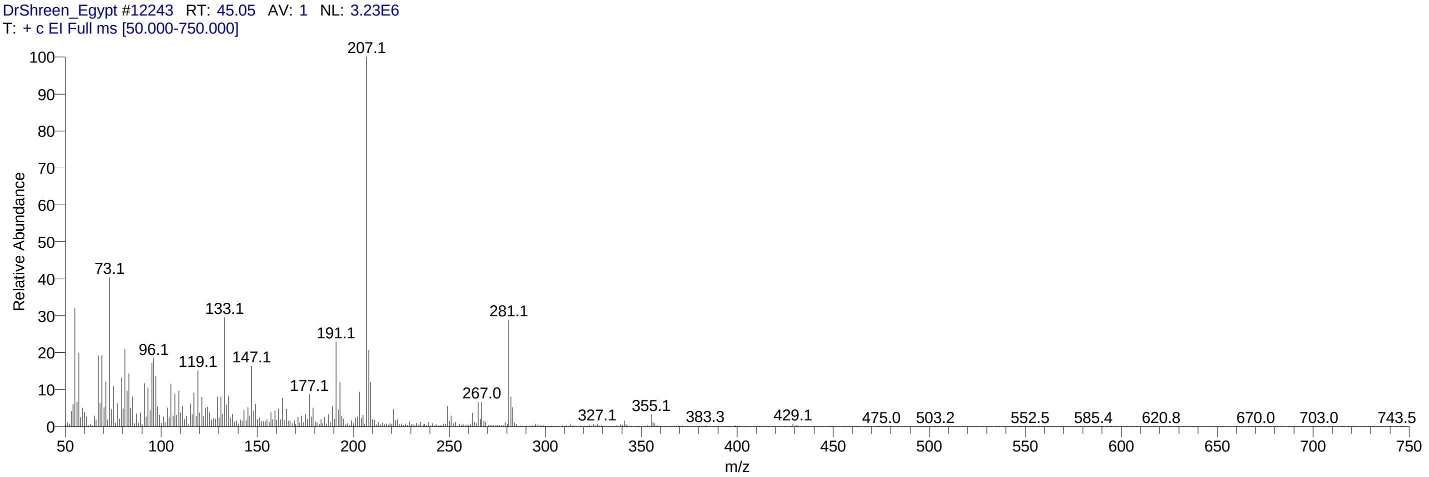

RT	Compound Name	Area %	MF	Molecular Formula	Molecular Weight	Cas #	Library
45.05	9,12-OCTADECADIENOIC ACID (Z,Z)-, 2,3-BIS[(TRIMETHYLSILYL)OXY]PROPYL ESTER	0.66	768	C27H54O4Si2	498	54284-45-6	WileyRegistry8e
45.05	à-D-GALACTOPYRANOSIDE, METHYL 2,3-BIS-O-(TRIMETHYLSILYL)-, CYCLIC BUTYLBORONATE	0.66	667	C17H37BO6Si2	404	56211-11-1	WileyRegistry8e
45.05	Loperamide	0.66	651	C29H33ClN2O2	476	34552-83-5	CaymanSpectralLibrary-NIST.HP
45.05	ARABINITOL, PENTAACETATE	0.66	719	C15H22O10	362	26674-23-7	WileyRegistry8e
45.05	SILANE, TRIMETHYL[[(3á)-STIGMAST-5-EN-3-YL]OXY]-	0.66	674	C32H58OSi	486	2625-46-9	WileyRegistry8e

Compound Structure	Hit Spectrum

9,12-OCTADECADIENOIC ACID (Z,Z)-, 2,3-BIS[(TRIMETHYLSILYL)OXY]PROPYL ESTER
Formula C27H54O4Si2, MW 498, CAS# 54284-45-6, Entry# 285148
2,3-BIS[(TRIMETHYLSILYL)OXY]PROPYL (9Z,12Z)-9,12-OCTADECADIENOATE #

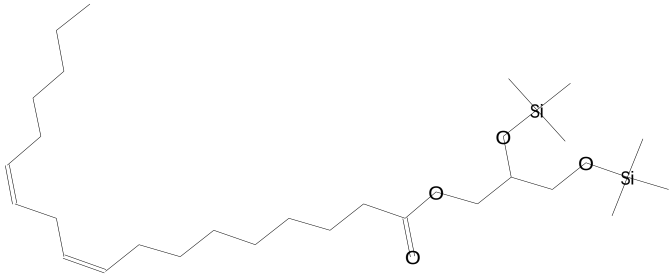

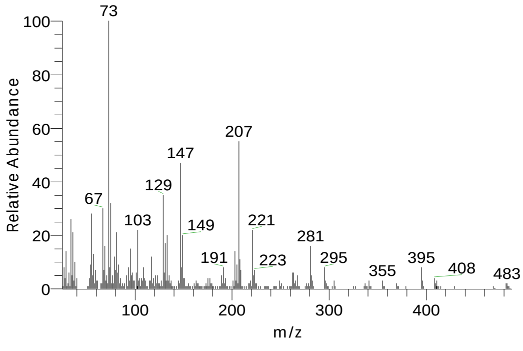

à-D-GALACTOPYRANOSIDE, METHYL 2,3-BIS-O-(TRIMETHYLSILYL)-, CYCLIC BUTYLBORONATE
Formula C17H37BO6Si2, MW 404, CAS# 56211-11-1, Entry# 257663

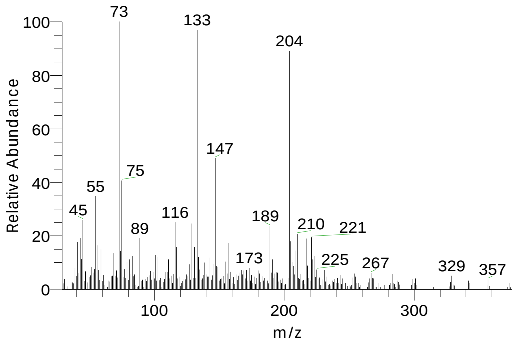

My GC-MS Report

Compound Structure | Hit Spectrum

Loperamide
Formula C29H33ClN2O2, MW 476, CAS# 34552-83-5, Entry# 1181

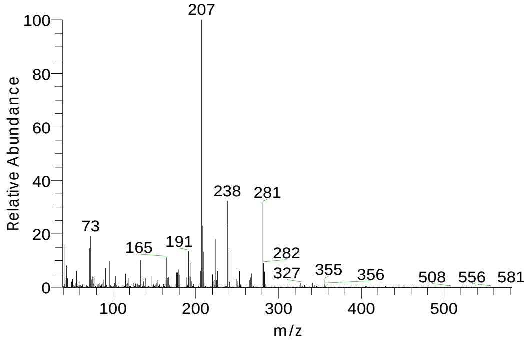

ARABINITOL, PENTAACETATE
Formula C15H22O10, MW 362, CAS# 26674-23-7, Entry# 235892
1,2,3,4,5-PENTA-O-ACETYLPENTITOL #

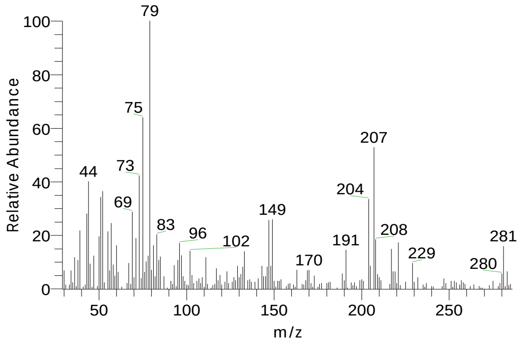

SILANE, TRIMETHYL[[(3á)-STIGMAST-5-EN-3-YL]OXY]-
Formula C32H58OSi, MW 486, CAS# 2625-46-9, Entry# 283210
3-[(TRIMETHYLSILYL)OXY]STIGMAST-5-ENE #

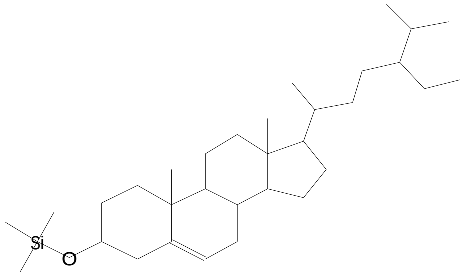

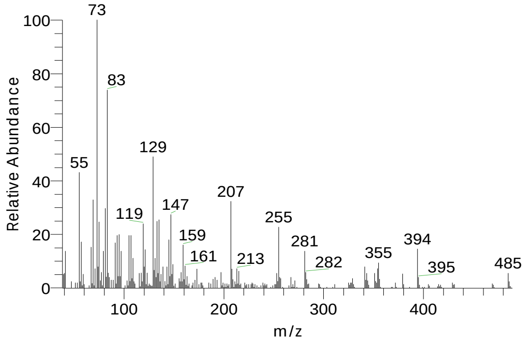

Supplement: Supplementary file 3 — Supplementary Material 3 [file 41598_2025_25896_MOESM3_ESM.pdf]
