# Supplementary material for: A comparative gas chromatography-mass spectrometry (GC-MS) profiling of Egyptian and Indian ashwagandha (Withania somnifera) root extracts
Source: Sci Rep. 2025 Nov 21;15:41156. doi: 10.1038/s41598-025-25896-3 (PMC12639082; doi:10.1038/s41598-025-25896-3)

My GC-MS Report

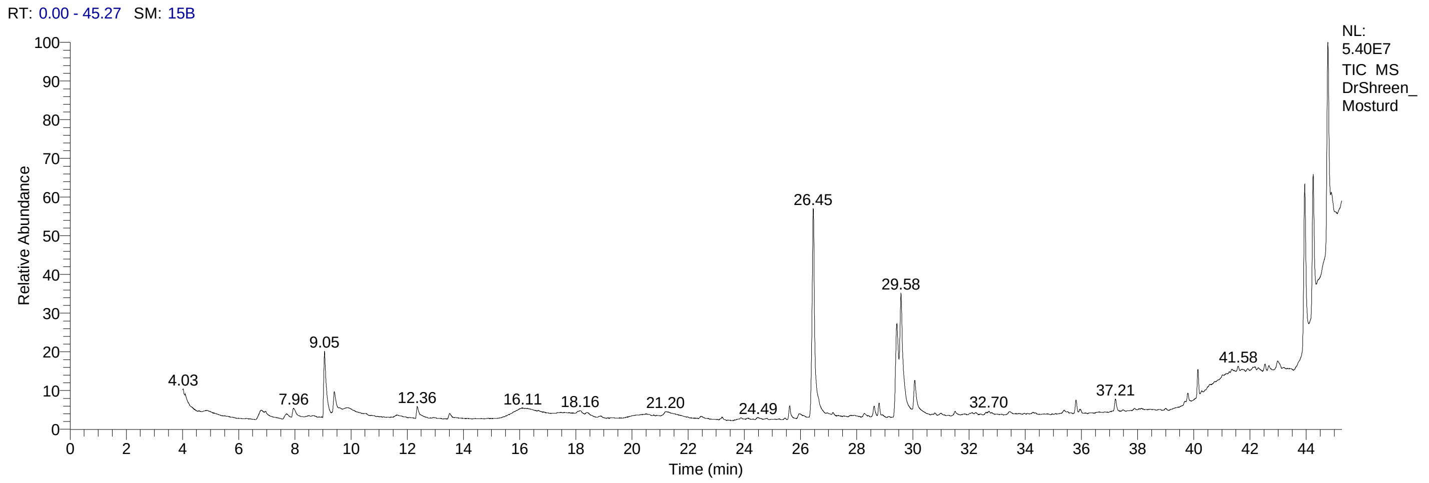

RT	Area %	Peak Area	Peak Height
6.79	1.22	9756701.16	952211.94
6.95	0.38	3058874.34	693440.45
7.95	0.95	7629332.21	1185318.15
9.05	5.03	40254886.31	9083999.38
9.40	1.66	13248353.22	2722409.02
12.35	1.24	9914334.29	1744630.39
13.51	0.48	3838503.94	725258.46
25.61	0.96	7651536.29	2028222.36
26.46	16.29	130385055.17	27760175.46
28.62	0.82	6563406.22	1558589.54
28.79	0.94	7490624.11	2133050.97
29.43	8.62	68948558.49	11364061.80
29.58	9.14	73129002.75	14288645.49
30.06	2.40	19178488.24	4019652.66
35.81	1.07	8568424.37	2132634.54
35.95	0.39	3086478.05	654109.06
37.21	0.94	7548405.79	1808662.48
39.79	0.77	6194003.34	1540256.57
40.15	1.69	13545308.97	4709815.10
41.58	0.33	2649995.76	763353.05
42.53	0.49	3958521.42	1107680.89
42.67	0.42	3329008.59	664115.65
42.99	1.10	8840213.50	1156960.08
43.95	12.58	100676568.50	22896367.09
44.24	9.75	78058079.16	20002173.76
44.77	20.34	162748111.40	30086229.84

My GC-MS Report

DrShreen_Mosturd #833 RT: 6.79 AV: 1 NL: 5.41E5
T: + c EI Full ms [50.000-750.000]

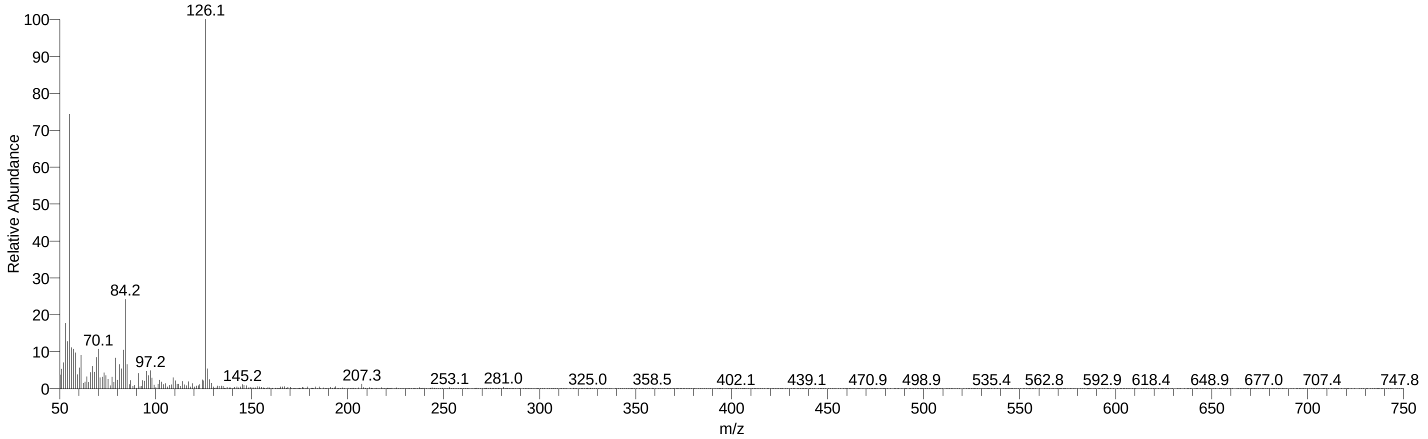

RT	Compound Name	Area %	MF	Molecular Formula	Molecular Weight	Cas #	Library
6.79	1H-Azonine, octahydro-1-nitroso-	1.22	813	C8H16N2O	156	20917-50-4	mainlib
6.79	1H-AZONINE, OCTAHYDRO-1-NITROSO-	1.22	806	C8H16N2O	156	20917-50-4	WileyRegistry8e
6.79	AH 7563	1.22	702	C16H24N2O	260	63886-94-2	CaymanSpectralLibrary-NIST.HP
6.79	1H-Azonine, octahydro-1-nitroso-	1.22	788	C8H16N2O	156	20917-50-4	replib
6.79	1H-AZONINE, OCTAHYDRO-1-NITROSO-	1.22	788	C8H16N2O	156	20917-50-4	WileyRegistry8e

Compound Structure | Hit Spectrum

1H-Azonine, octahydro-1-nitroso-
Formula C8H16N2O, MW 156, CAS# 20917-50-4, Entry# 110228
N-Nitrosoazacyclononane

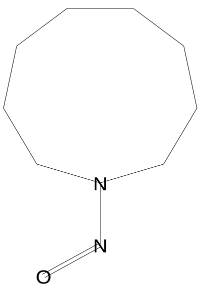

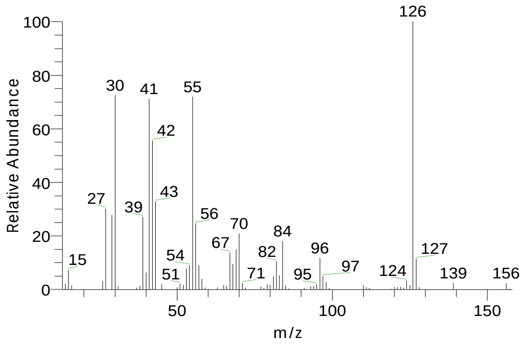

1H-AZONINE, OCTAHYDRO-1-NITROSO-
Formula C8H16N2O, MW 156, CAS# 20917-50-4, Entry# 40108
1-NITROSOAZONANE

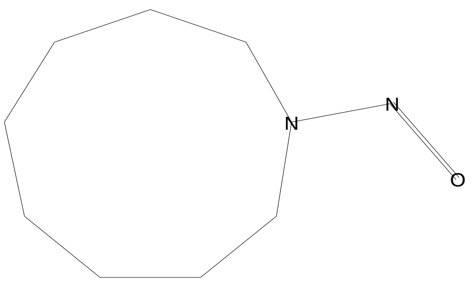

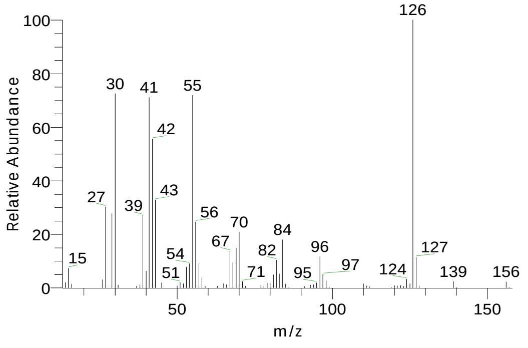

My GC-MS Report

Compound Structure	Hit Spectrum

AH 7563
Formula C16H24N2O, MW 260, CAS# 63886-94-2, Entry# 1064

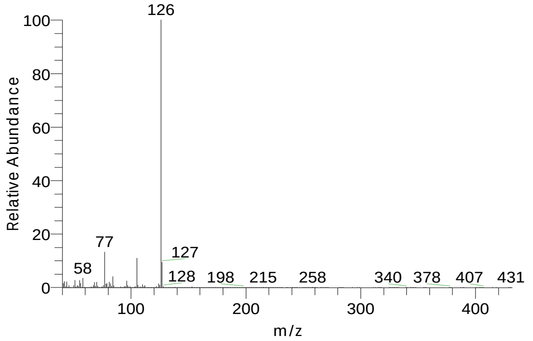

1H-Azonine, octahydro-1-nitroso-
Formula C8H16N2O, MW 156, CAS# 20917-50-4, Entry# 20216
N-Nitrosoazacyclononane

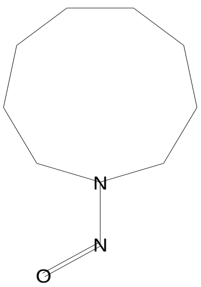

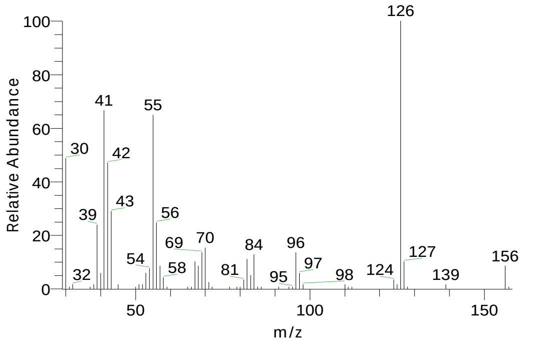

1H-AZONINE, OCTAHYDRO-1-NITROSO-
Formula C8H16N2O, MW 156, CAS# 20917-50-4, Entry# 40109
1-NITROSOAZONANE

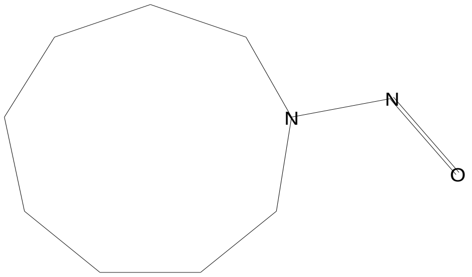

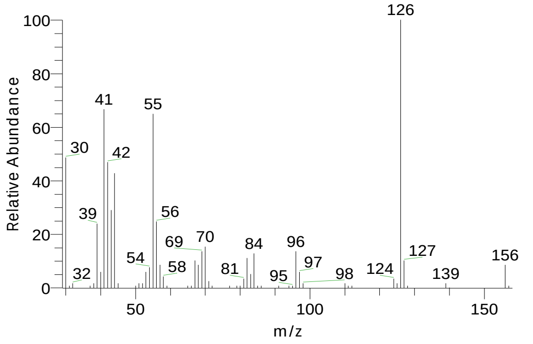

DrShreen_Mosturd #881 RT: 6.95 AV: 1 NL: 3.78E5
T: + c EI Full ms [50.000-750.000]

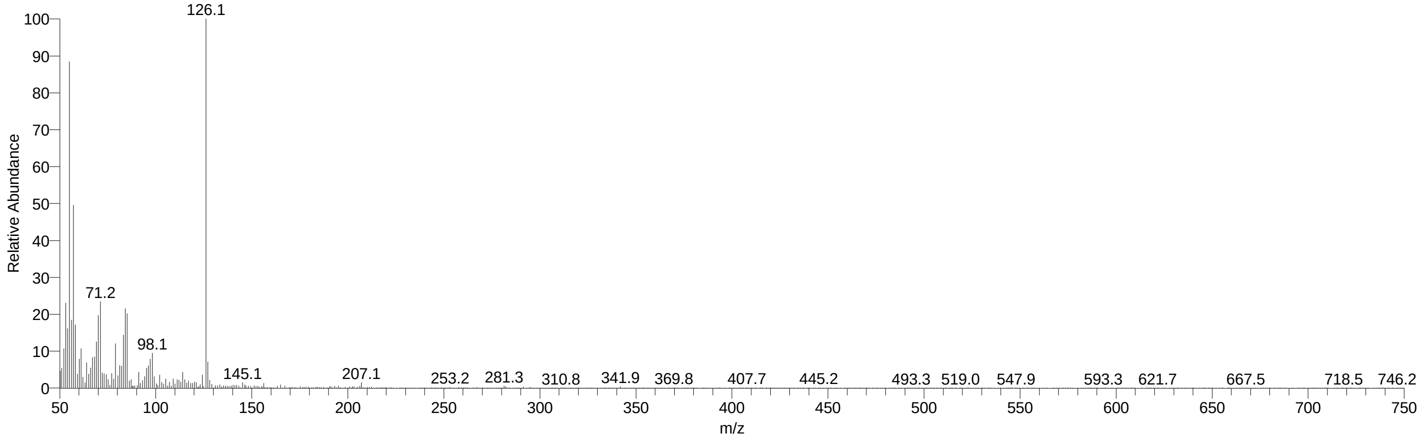

RT	Compound Name	Area %	MF	Molecular Formula	Molecular Weight	Cas #	Library
6.95	1H-Azonine, octahydro-1-nitroso-	0.38	784	C8H16N2O	156	20917-50-4	mainlib
6.95	1H-AZONINE, OCTAHYDRO-1-NITROSO-	0.38	777	C8H16N2O	156	20917-50-4	WileyRegistry8e

My GC-MS Report

RT	Compound Name	Area %	MF	Molecular Formula	Molecular Weight	Cas #	Library
6.95	4-Hexenoic acid, 6-(acetyloxy)-4-methyl-	0.38	684	C9H14O4	186	27872-60-2	mainlib
6.95	(4Z)-6-(ACETYLOXY)-4-METHYL-4-HEXENOIC ACID #	0.38	684	C9H14O4	186	27872-60-2	WileyRegistry8e
6.95	2,2-DIDEUTERO HEPTADECANAL	0.38	655	C17H32D2O	256	56555-01-2	WileyRegistry8e

Compound Structure	Hit Spectrum

1H-Azonine, octahydro-1-nitroso-
Formula C8H16N2O, MW 156, CAS# 20917-50-4, Entry# 110228
N-Nitrosoazacyclononane

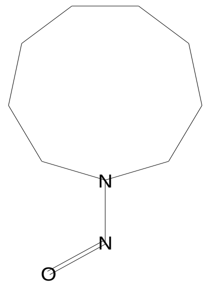

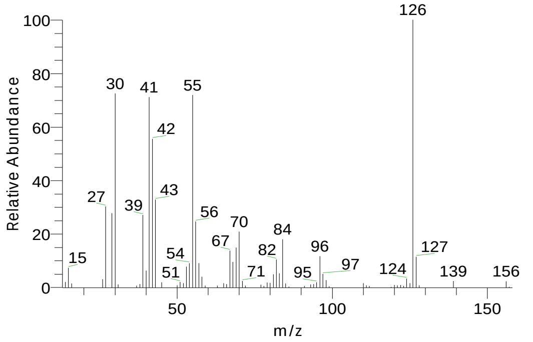

1H-AZONINE, OCTAHYDRO-1-NITROSO-
Formula C8H16N2O, MW 156, CAS# 20917-50-4, Entry# 40108
1-NITROSOAZONANE

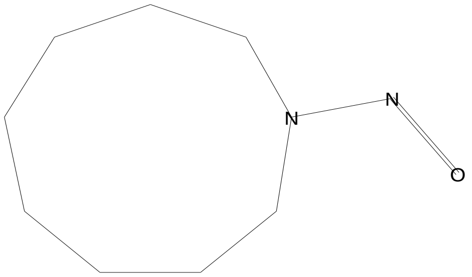

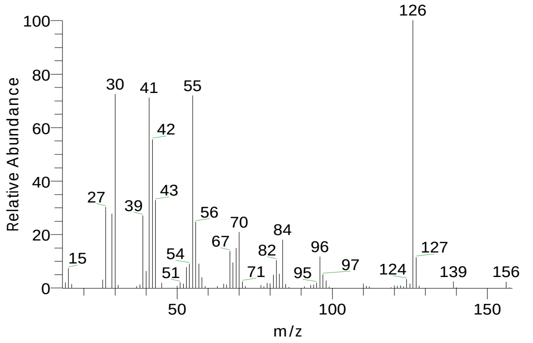

4-Hexenoic acid, 6-(acetyloxy)-4-methyl-
Formula C9H14O4, MW 186, CAS# 27872-60-2, Entry# 9547
(4Z)-6-(Acetyloxy)-4-methyl-4-hexenoic acid #

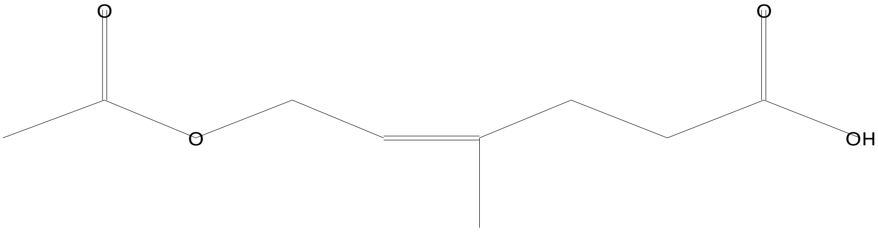

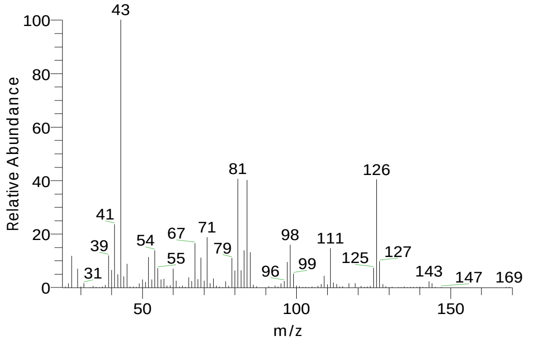

(4Z)-6-(ACETYLOXY)-4-METHYL-4-HEXENOIC ACID #
Formula C9H14O4, MW 186, CAS# 27872-60-2, Entry# 375091
(4Z)-6-(ACETYLOXY)-4-METHYL-4-HEXENOIC ACID

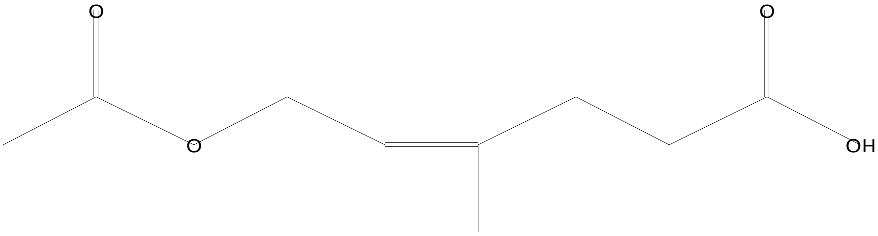

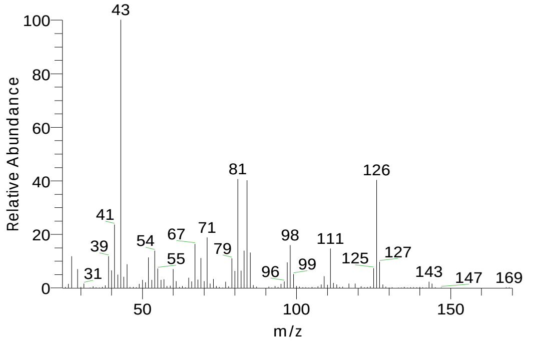

My GC-MS Report

Compound Structure | Hit Spectrum

2,2-DIDEUTERO HEPTADECANAL
Formula C17H32D2O, MW 256, CAS# 56555-01-2, Entry# 144932

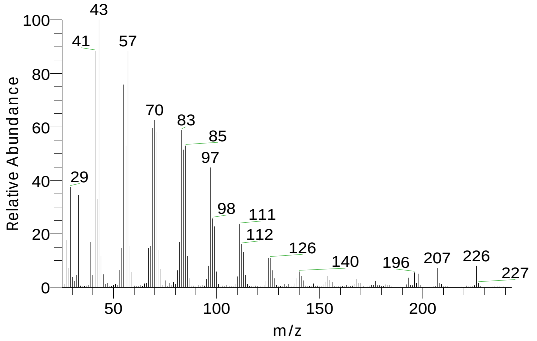

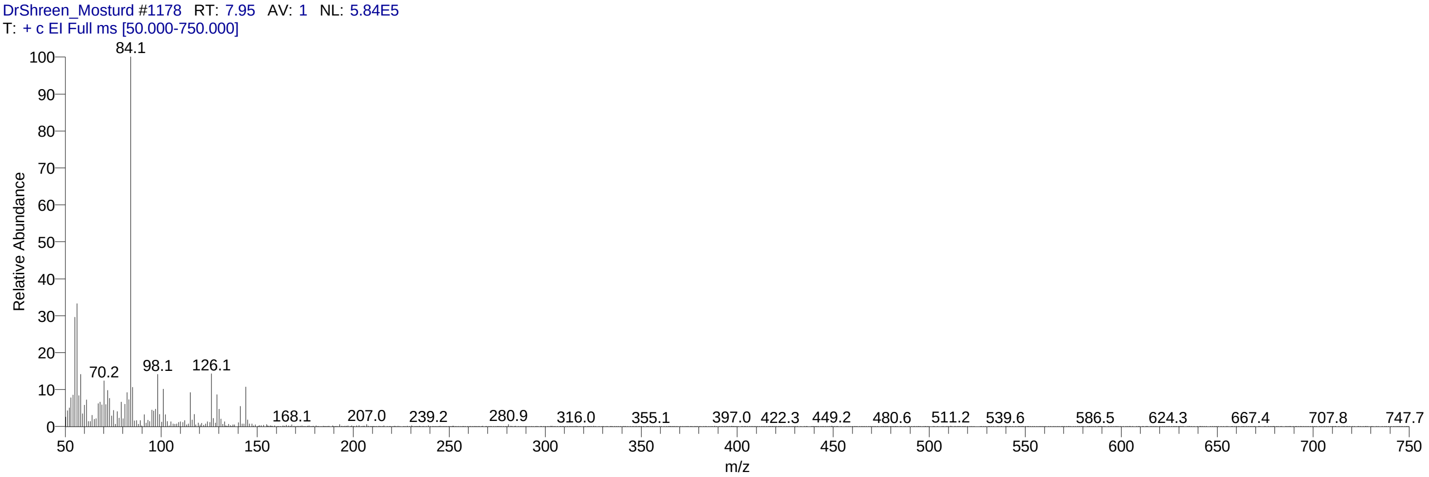

RT	Compound Name	Area %	MF	Molecular Formula	Molecular Weight	Cas #	Library
7.95	1H-Azepine, hexahydro-3,3,5-trimethyl-	0.95	686	C9H19N	141	35466-89-8	replib
7.95	á-METHYL-ç,.DELTA.-DIHYDROXY-LEUCINE	0.95	720	C7H15NO4	177	NA	WileyRegistry8e
7.95	L-ISOLEUCINE, 4,5-DIHYDROXY-4-METHYL-	0.95	720	C7H15NO4	177	69597-54-2	WileyRegistry8e
7.95	NONANOYL CHLORIDE	0.95	637	C9H17ClO	176	764-85-2	WileyRegistry8e
7.95	5-Oxopyrrolidine-2-carboxylic acid, (2-hydroxyethyl)amide	0.95	674	C7H12N2O3	172	NA	mainlib

Compound Structure | Hit Spectrum

1H-Azepine, hexahydro-3,3,5-trimethyl-
Formula C9H19N, MW 141, CAS# 35466-89-8, Entry# 5472
3,3,5-Trimethylhexahydroazepine

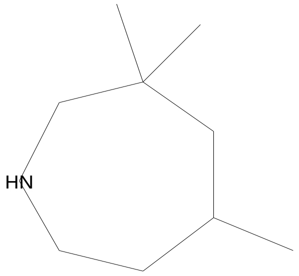

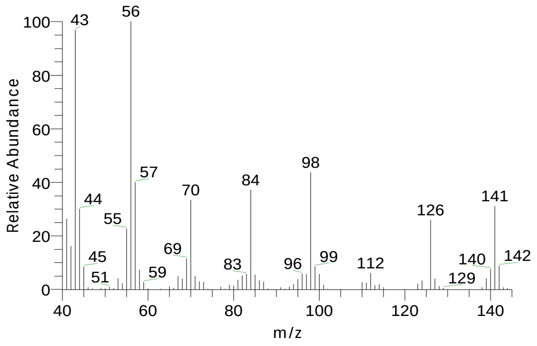

My GC-MS Report

Compound Structure | Hit Spectrum

á-METHYL-ç,.DELTA.-DIHYDROXY-LEUCINE
Formula C7H15NO4, MW 177, CAS# NA, Entry# 59572

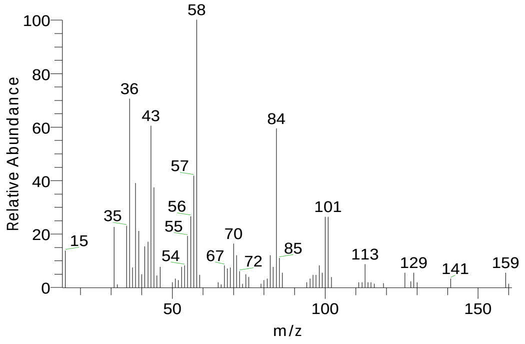

L-ISOLEUCINE, 4,5-DIHYDROXY-4-METHYL-
Formula C7H15NO4, MW 177, CAS# 69597-54-2, Entry# 59573

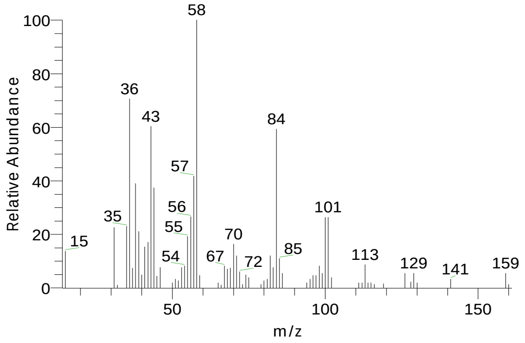

NONANOYL CHLORIDE
Formula C9H17ClO, MW 176, CAS# 764-85-2, Entry# 58772
EINECS 212-131-2

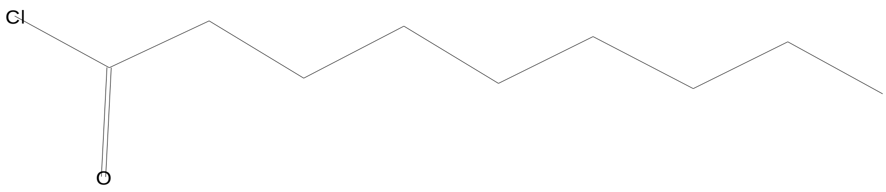

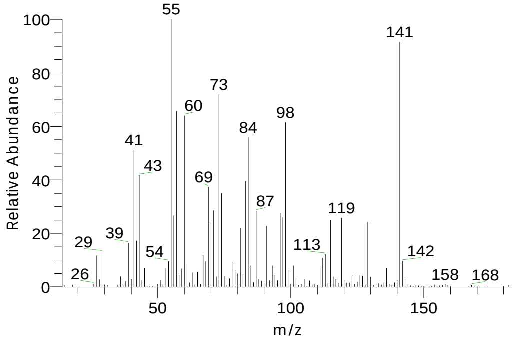

5-Oxopyrrolidine-2-carboxylic acid, (2-hydroxyethyl)amide
Formula C7H12N2O3, MW 172, CAS# NA, Entry# 54040
$:28NXFCCDYJVCRYDG-UHFFFAOYSA-N

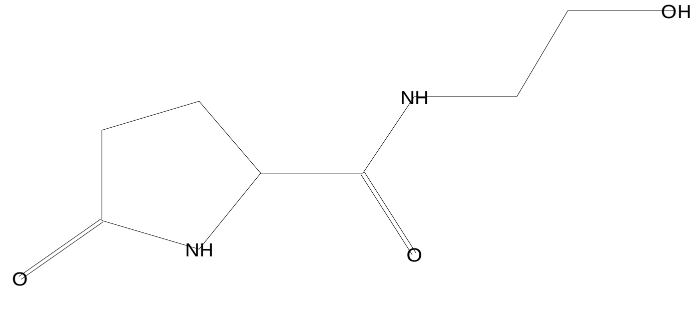

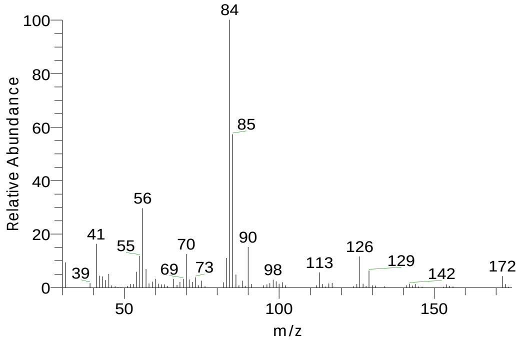

My GC-MS Report

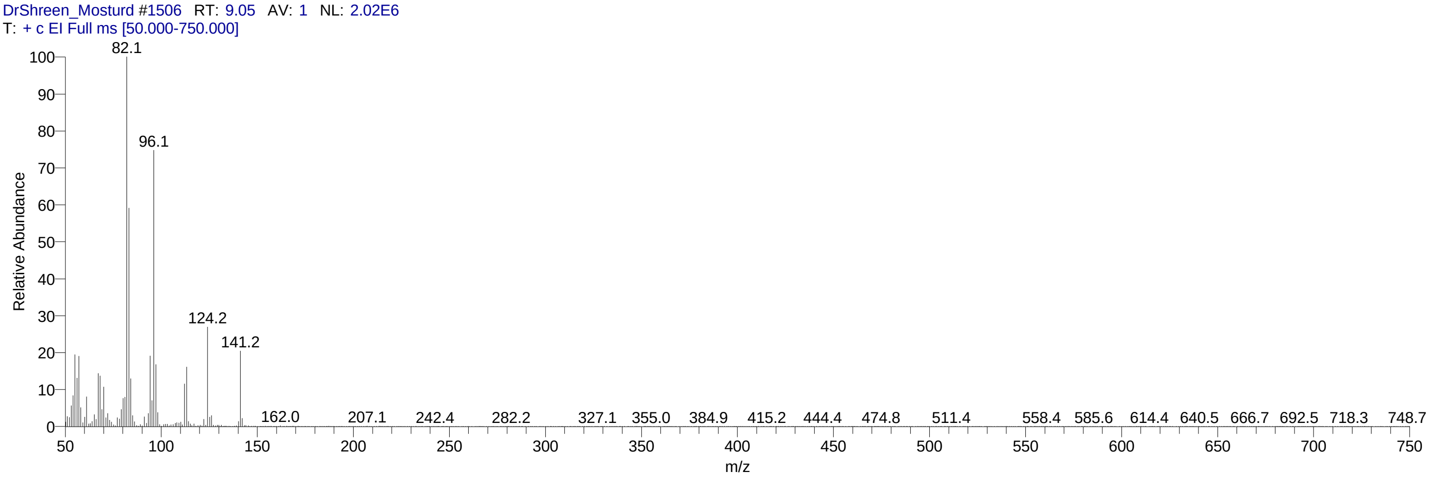

RT	Compound Name	Area %	MF	Molecular Formula	Molecular Weight	Cas #	Library
9.05	8-METHYL-8-AZABICYCLO[3.2.1] OCTAN-3-OL	5.03	926	C8H15NO	141	NA	WileyRegistry8e
9.05	8-Azabicyclo[3.2.1]octan-3-ol, 8-methyl-, endo-	5.03	925	C8H15NO	141	120-29-6	mainlib
9.05	8-METHYL-8-AZABICYCLO[3.2.1] OCTAN-3-OL	5.03	925	C8H15NO	141	120-29-6	WileyRegistry8e
9.05	8-Azabicyclo[3.2.1]octan-3-ol, 8-methyl-, endo-	5.03	920	C8H15NO	141	120-29-6	replib
9.05	8-AZABICYCLO[3.2.1]OCTAN-3-OL, 8-METHYL-, ENDO-	5.03	920	C8H15NO	141	120-29-6	WileyRegistry8e

Compound Structure | Hit Spectrum

8-METHYL-8-AZABICYCLO[3.2.1]OCTAN-3-OL
Formula C8H15NO, MW 141, CAS# NA, Entry# 397029
8-METHYL-8-AZA-BICYCLO[3.2.1]OCTAN-3-OL

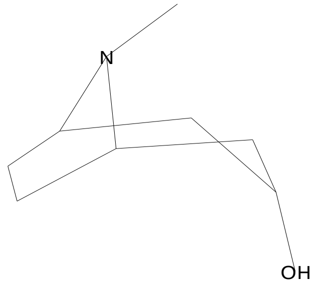

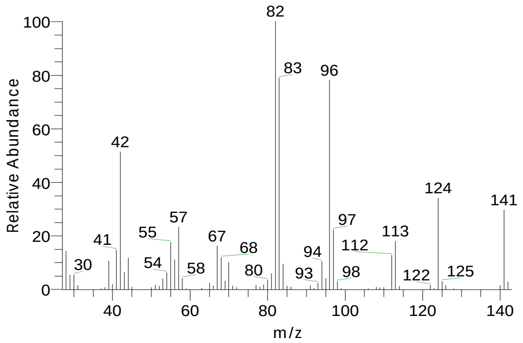

8-Azabicyclo[3.2.1]octan-3-ol, 8-methyl-, endo-
Formula C8H15NO, MW 141, CAS# 120-29-6, Entry# 51507
Tropine

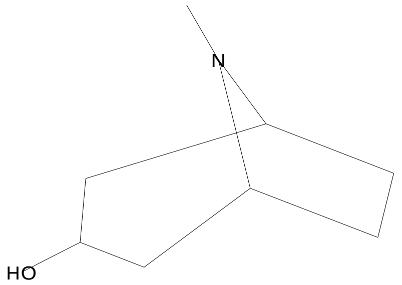

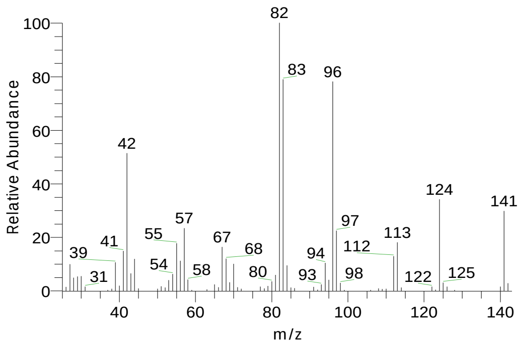

My GC-MS Report

Compound Structure | Hit Spectrum

8-METHYL-8-AZABICYCLO[3.2.1]OCTAN-3-OL
Formula C8H15NO, MW 141, CAS# 120-29-6, Entry# 359696
8-METHYL-8-AZA-BICYCLO[3.2.1]OCTAN-3-OL

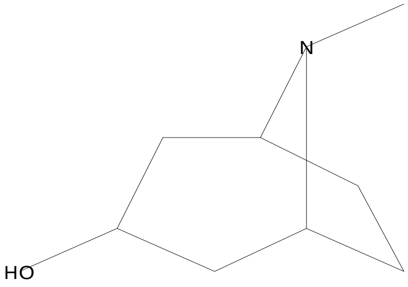

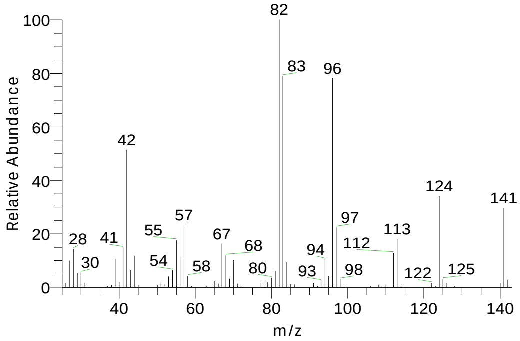

8-Azabicyclo[3.2.1]octan-3-ol, 8-methyl-, endo-
Formula C8H15NO, MW 141, CAS# 120-29-6, Entry# 11803
Tropine

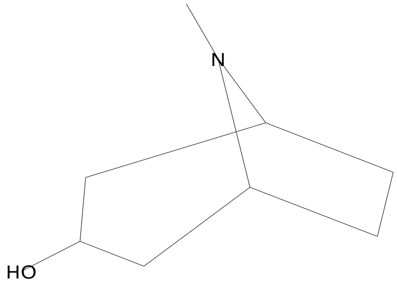

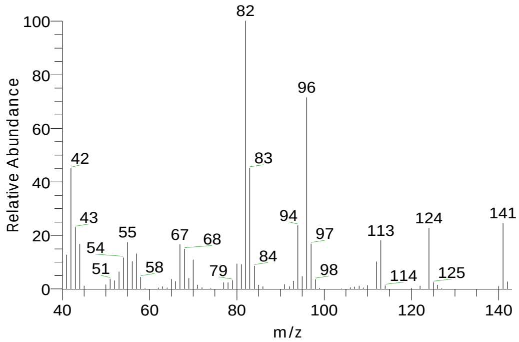

8-AZABICYCLO[3.2.1]OCTAN-3-OL, 8-METHYL-, ENDO-
Formula C8H15NO, MW 141, CAS# 120-29-6, Entry# 394844
8-METHYL-8-AZABICYCLO[3.2.1]OCTAN-3-OL

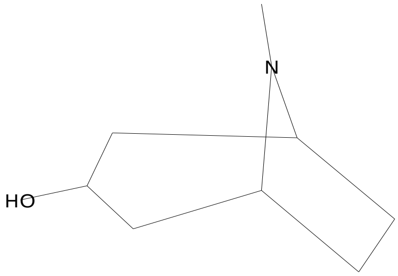

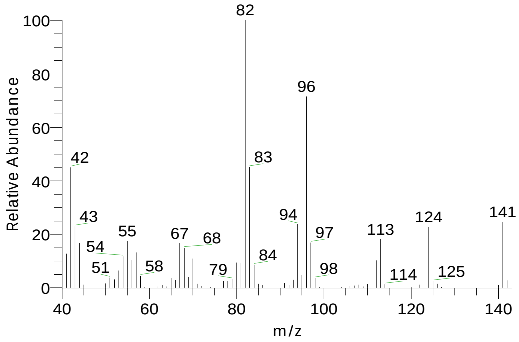

DrShreen_Mosturd #1611 RT: 9.40 AV: 1 NL: 9.17E5
T: + c EI Full ms [50.000-750.000]

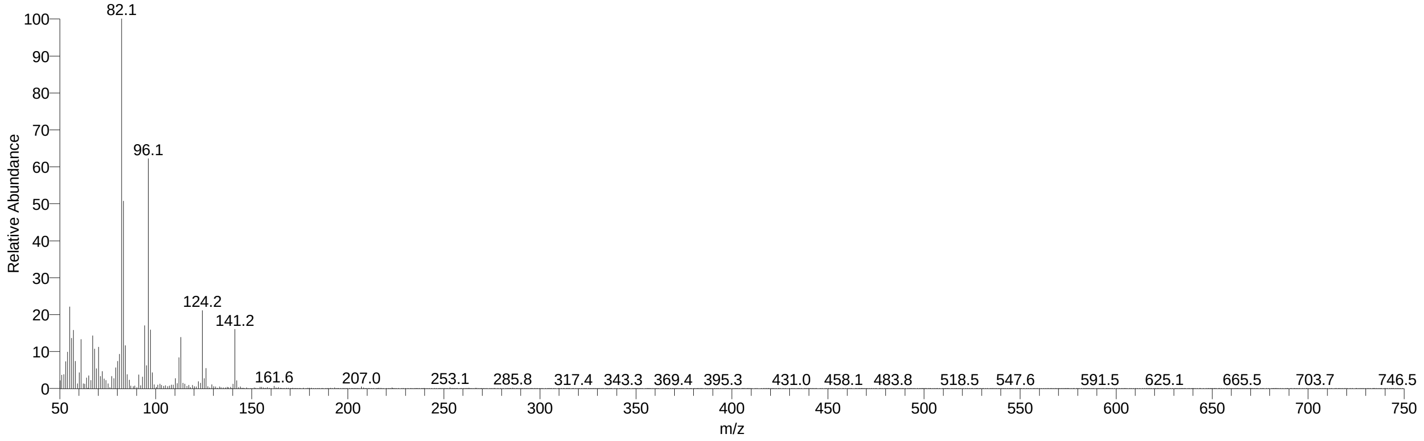

RT	Compound Name	Area %	MF	Molecular Formula	Molecular Weight	Cas #	Library
9.40	8-Azabicyclo[3.2.1]octan-3-ol, 8-methyl-, endo-	1.66	899	C8H15NO	141	120-29-6	replib
9.40	8-AZABICYCLO[3.2.1]OCTAN-3-OL, 8-METHYL-, ENDO-	1.66	899	C8H15NO	141	120-29-6	WileyRegistry8e

My GC-MS Report

RT	Compound Name	Area %	MF	Molecular Formula	Molecular Weight	Cas #	Library
9.40	8-METHYL-8-AZABICYCLO[3.2.1] OCTAN-3-OL	1.66	879	C8H15NO	141	NA	WileyRegistry8e
9.40	8-METHYL-8-AZABICYCLO[3.2.1] OCTAN-3-OL	1.66	878	C8H15NO	141	120-29-6	WileyRegistry8e
9.40	8-Azabicyclo[3.2.1]octan-3-ol, 8-methyl-, endo-	1.66	877	C8H15NO	141	120-29-6	mainlib

Compound Structure | Hit Spectrum

8-Azabicyclo[3.2.1]octan-3-ol, 8-methyl-, endo-
Formula C8H15NO, MW 141, CAS# 120-29-6, Entry# 11803
Tropine

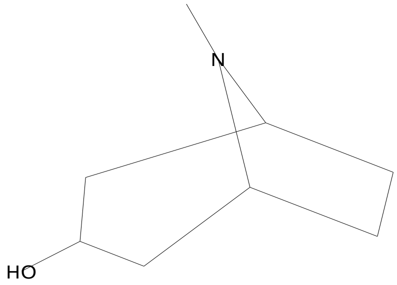

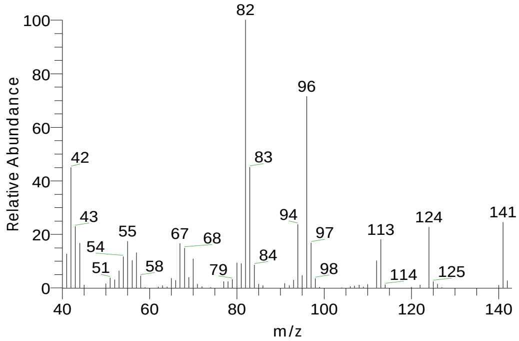

8-AZABICYCLO[3.2.1]OCTAN-3-OL, 8-METHYL-, ENDO-
Formula C8H15NO, MW 141, CAS# 120-29-6, Entry# 394844
8-METHYL-8-AZABICYCLO[3.2.1]OCTAN-3-OL

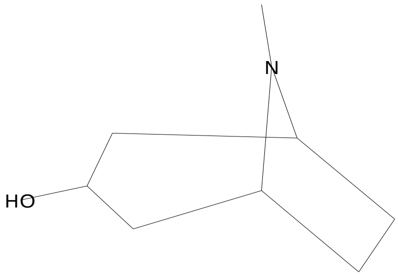

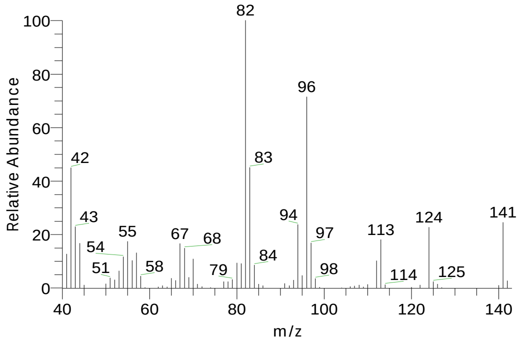

8-METHYL-8-AZABICYCLO[3.2.1]OCTAN-3-OL
Formula C8H15NO, MW 141, CAS# NA, Entry# 397029
8-METHYL-8-AZA-BICYCLO[3.2.1]OCTAN-3-OL

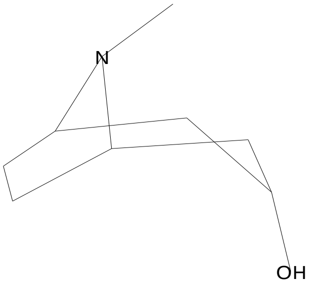

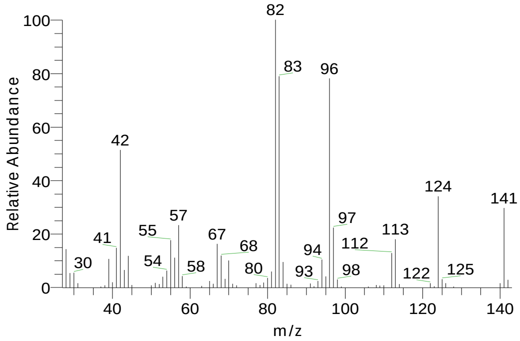

8-METHYL-8-AZABICYCLO[3.2.1]OCTAN-3-OL
Formula C8H15NO, MW 141, CAS# 120-29-6, Entry# 359696
8-METHYL-8-AZA-BICYCLO[3.2.1]OCTAN-3-OL

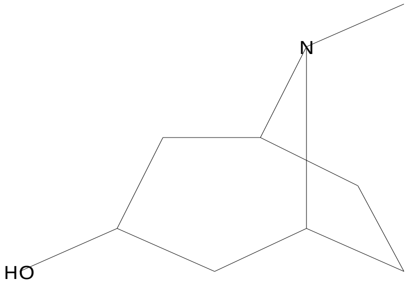

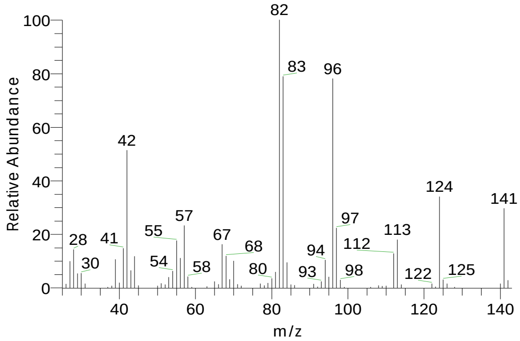

My GC-MS Report

Compound Structure | Hit Spectrum

8-Azabicyclo[3.2.1]octan-3-ol, 8-methyl-, endo-
Formula C8H15NO, MW 141, CAS# 120-29-6, Entry# 51507
Tropine

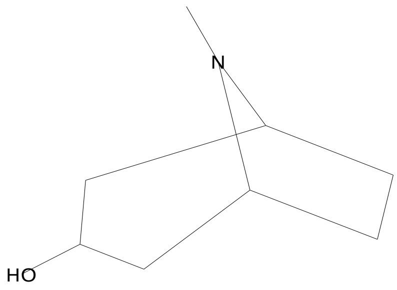

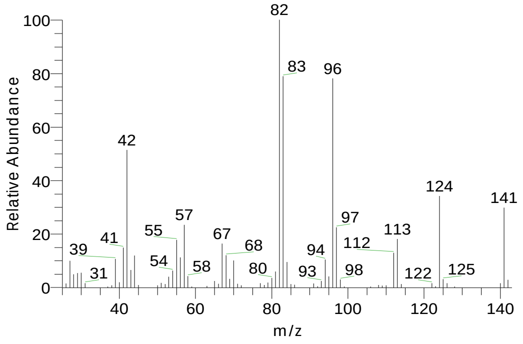

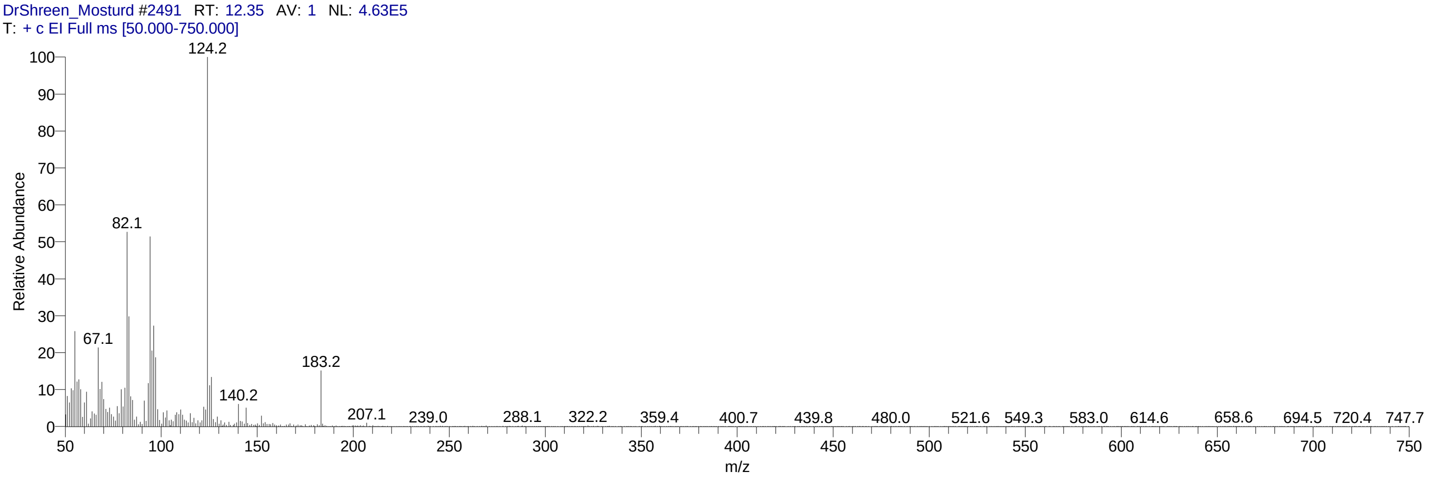

RT	Compound Name	Area %	MF	Molecular Formula	Molecular Weight	Cas #	Library
12.35	8-AZABICYCLO[3.2.1]OCTANE-2-CARBOXYLIC ACID, 3-(BENZOYLOXY)-8-METHYL-, [1R-(EXO,EXO)]-	1.24	758	C16H19NO4	289	519-09-5	WileyRegistry8e
12.35	Benzoylecgonine	1.24	758	C16H19NO4	289	519-09-5	mainlib
12.35	8-Azabicyclo[3.2.1]octan-3-ol,8-methyl-acetate(ester),exo-	1.24	871	C10H17NO2	183	3423-26-5	mainlib
12.35	3,4-Dichloroatropine	1.24	765	C17H21Cl2NO3	357	134842-74-3	mainlib
12.35	8-AZABICYCLO[3.2.1]OCTAN-3-OL, 8-METHYL-, BENZOATE (ESTER), EXO-	1.24	707	C15H19NO2	245	537-26-8	WileyRegistry8e

Compound Structure | Hit Spectrum

Formula C16H19NO4, MW 289, CAS# 519-09-5, Entry# 395609
3-(BENZOYLOXY)-8-METHYL-8-AZABICYCLO[3.2.1]OCTANE-2-CARBOXYLIC ACID #

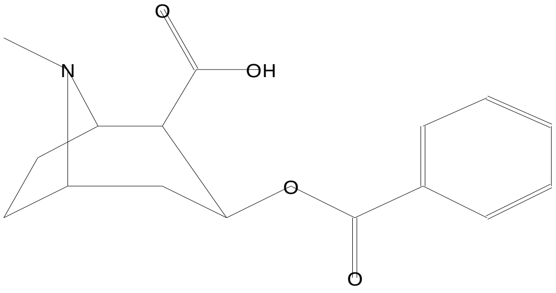

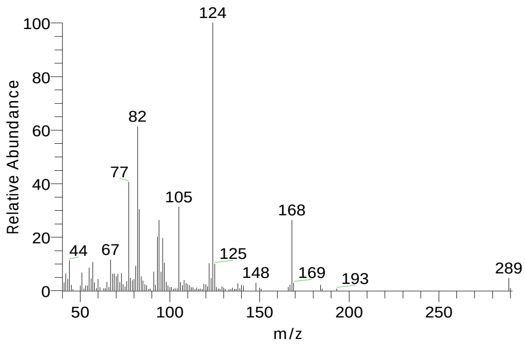

My GC-MS Report

Compound Structure | Hit Spectrum

Benzoylecgonine
Formula C16H19NO4, MW 289, CAS# 519-09-5, Entry# 108027
8-Azabicyclo[3.2.1]octane-2-carboxylic acid, 3-(benzoyloxy)-8-methyl-, [1R-(exo,exo)]-

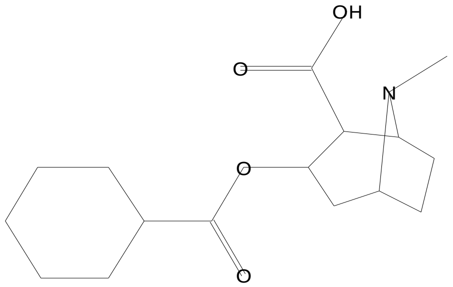

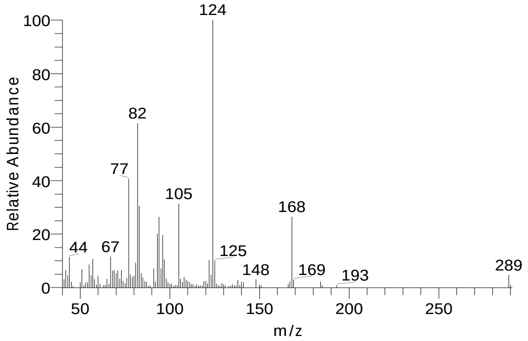

8-Azabicyclo[3.2.1]octan-3-ol,8-methyl-acetate(ester),exo-
Formula C10H17NO2, MW 183, CAS# 3423-26-5, Entry# 108048
$:28MDIDMOWWLBGYPG-UHFFFAOYSA-N

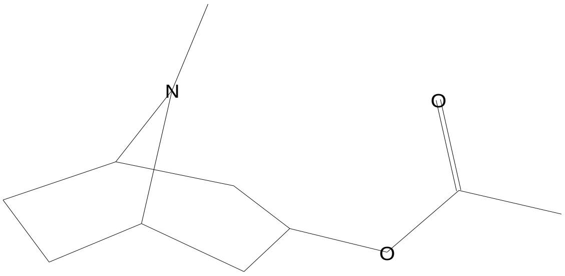

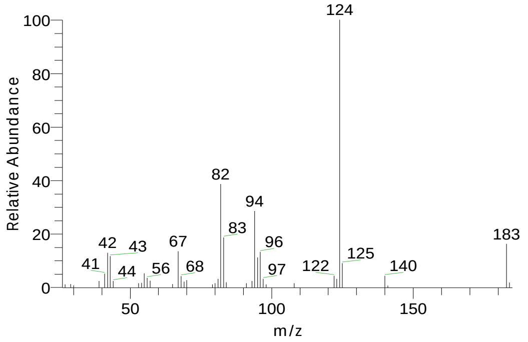

3,4-Dichloroatropine
Formula C17H21Cl2NO3, MW 357, CAS# 134842-74-3, Entry# 108037
8-Methyl-8-azabicyclo[3.2.1]oct-3-yl 2-(3,4-dichlorophenyl)-3-hydroxypropanoate #

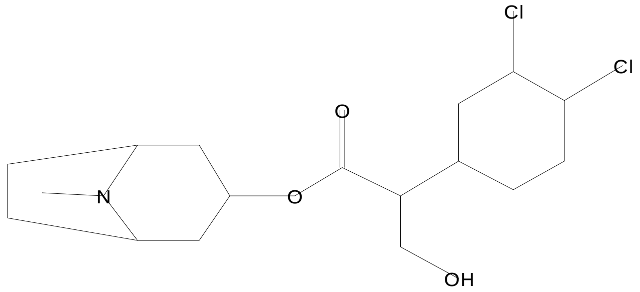

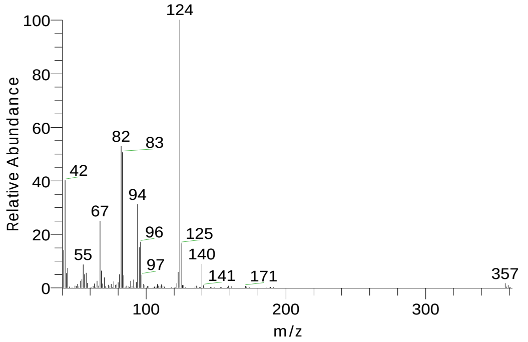

8-AZABICYCLO[3.2.1]OCTAN-3-OL, 8-METHYL-, BENZOATE (ESTER), EXO-
Formula C15H19NO2, MW 245, CAS# 537-26-8, Entry# 395307
8-METHYL-8-AZABICYCLO[3.2.1]OCT-3-YL BENZOATE #

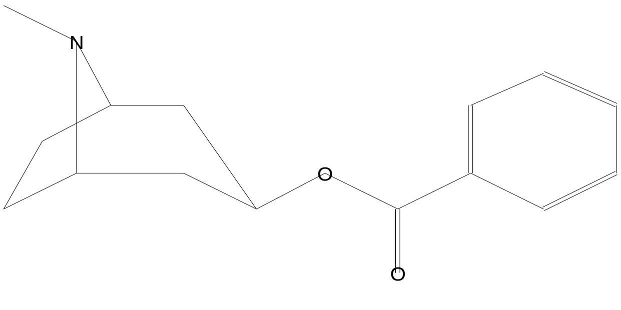

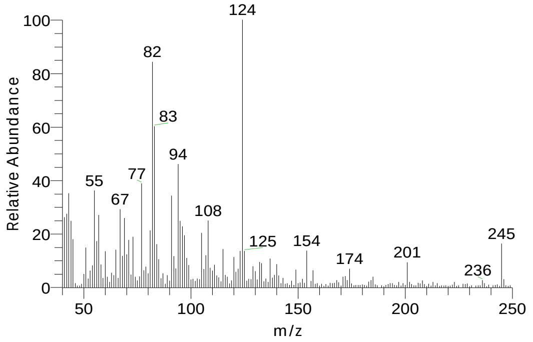

My GC-MS Report

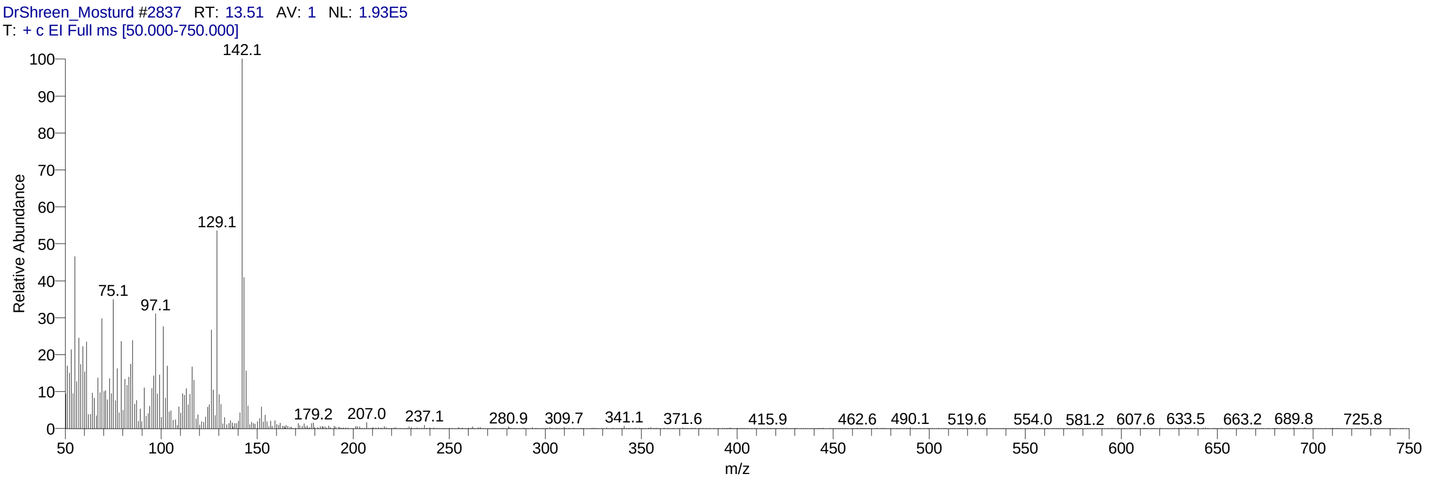

RT	Compound Name	Area %	MF	Molecular Formula	Molecular Weight	Cas #	Library
13.51	4-Methyl(trimethylene)silyloxyoctane	0.48	682	C12H26OSi	214	NA	mainlib
13.51	11-AMINOUNDECANOIC ACID	0.48	691	C11H23NO2	201	2432-99-7	WileyRegistry8e
13.51	Decanedioic acid, 3,8-dioxo-, dimethyl ester	0.48	652	C12H18O6	258	55030-37-0	mainlib
13.51	DECANEDIOIC ACID, 3,8-DIOXO-, DIMETHYL ESTER	0.48	652	C12H18O6	258	55030-37-0	WileyRegistry8e
13.51	Undecanoic acid, 11-amino-	0.48	686	C11H23NO2	201	2432-99-7	replib

Compound Structure | Hit Spectrum

4-Methyl(trimethylene)silyloxyoctane
Formula C12H26OSi, MW 214, CAS# NA, Entry# 130662
1-Methyl-1-[(1-propylpentyl)oxy]siletane #

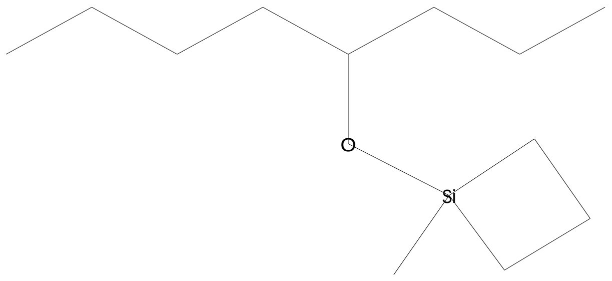

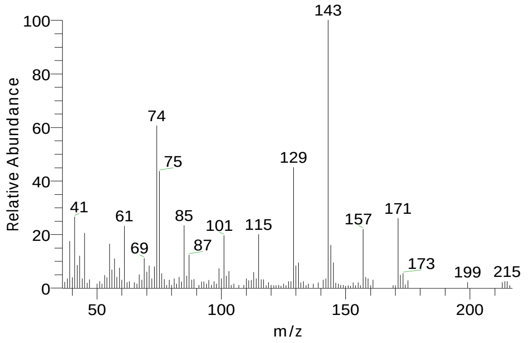

11-AMINOUNDECANOIC ACID
Formula C11H23NO2, MW 201, CAS# 2432-99-7, Entry# 85354
ê-AMINOUNDECANOIC ACID

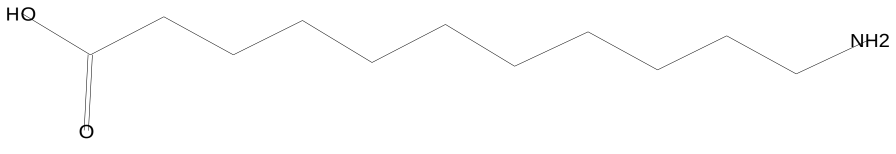

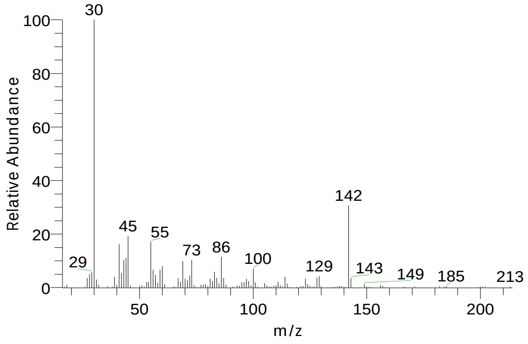

My GC-MS Report

Compound Structure | Hit Spectrum

Decanedioic acid, 3,8-dioxo-, dimethyl ester
Formula C12H18O6, MW 258, CAS# 55030-37-0, Entry# 12344
Dimethyl 3,8-dioxodecanedioate #

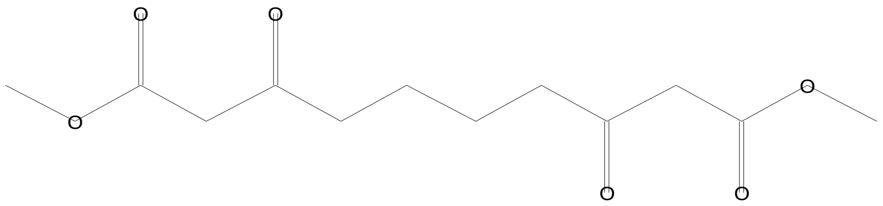

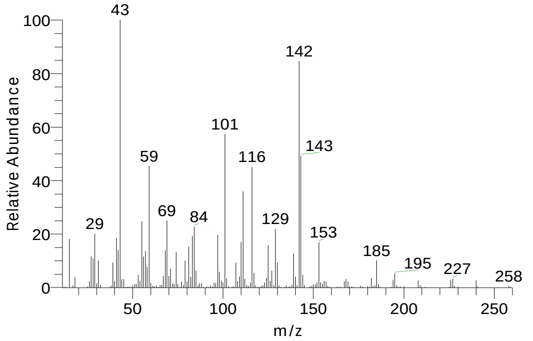

DECANEDIOIC ACID, 3,8-DIOXO-, DIMETHYL ESTER
Formula C12H18O6, MW 258, CAS# 55030-37-0, Entry# 147984
DIMETHYL 3,8-DIOXODECANEDIOATE

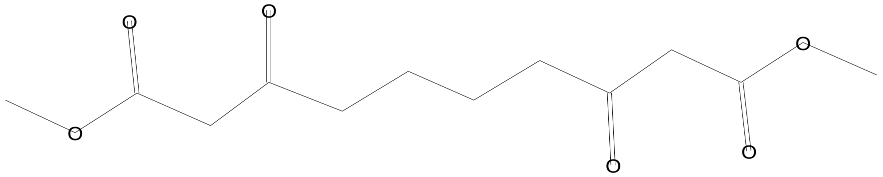

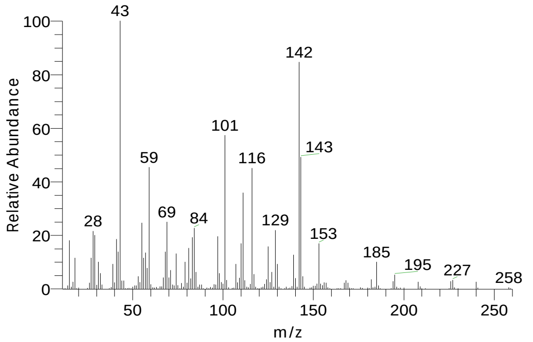

Undecanoic acid, 11-amino-
Formula C11H23NO2, MW 201, CAS# 2432-99-7, Entry# 556
11-Aminoundecanoic acid

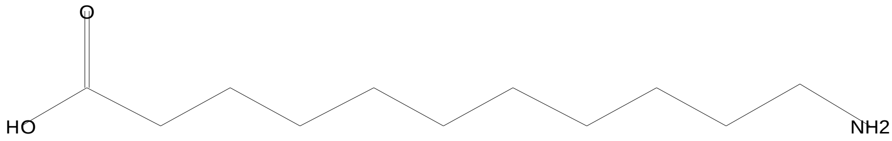

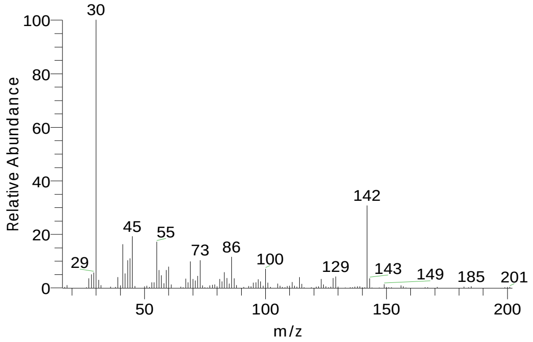

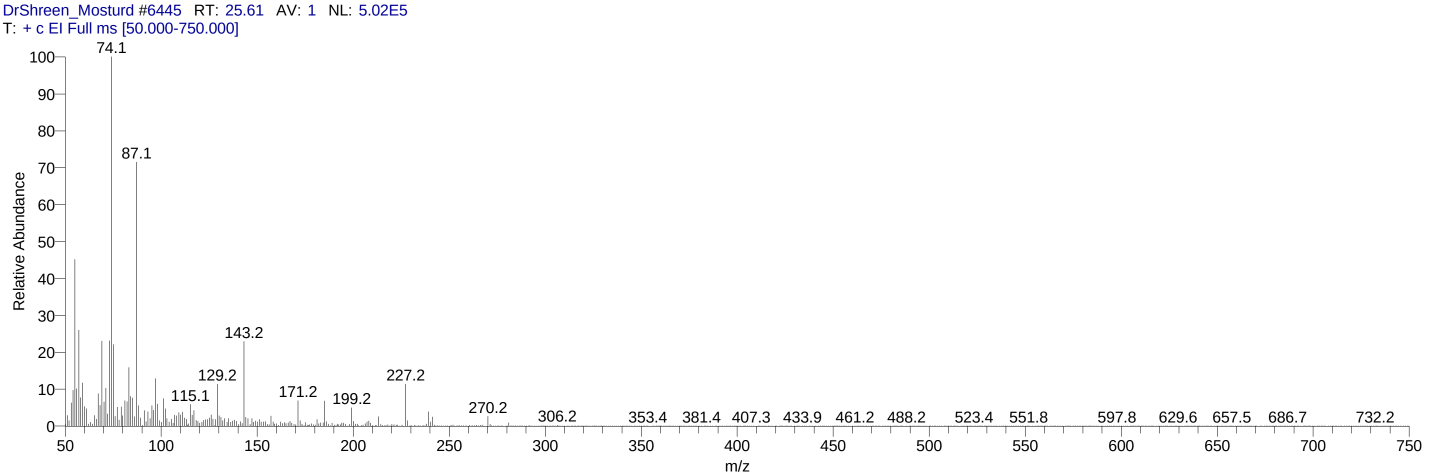

RT	Compound Name	Area %	MF	Molecular Formula	Molecular Weight	Cas #	Library
25.61	PENTADECANOIC ACID, 14-METHYL-, METHYL ESTER	0.96	783	C17H34O2	270	5129-60-2	WileyRegistry8e
25.61	HEXADECANOIC ACID, METHYL ESTER	0.96	796	C17H34O2	270	112-39-0	WileyRegistry8e

My GC-MS Report

RT	Compound Name	Area %	MF	Molecular Formula	Molecular Weight	Cas #	Library
25.61	PENTADECANOIC ACID, 14-METHYL-, METHYL ESTER	0.96	804	C17H34O2	270	5129-60-2	WileyRegistry8e
25.61	HEXADECANOIC ACID, METHYL ESTER	0.96	843	C17H34O2	270	112-39-0	WileyRegistry8e
25.61	Hexadecanoic acid, methyl ester	0.96	841	C17H34O2	270	112-39-0	replib

Compound Structure

Hit Spectrum

PENTADECANOIC ACID, 14-METHYL-, METHYL ESTER
Formula C17H34O2, MW 270, CAS# 5129-60-2, Entry# 161312
METHYL 14-METHYLPENTADECANOATE

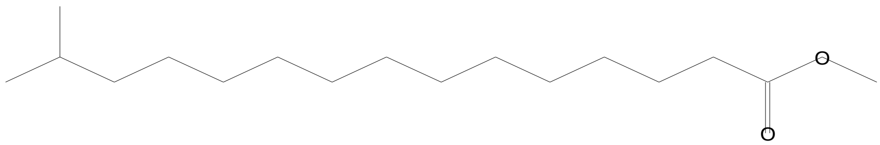

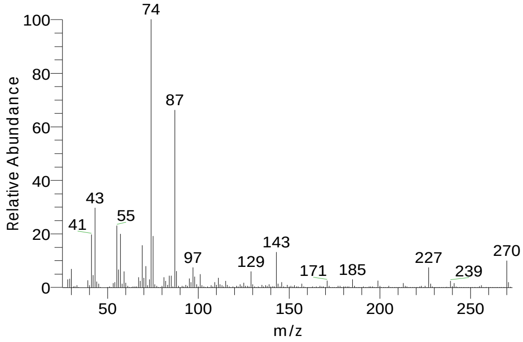

HEXADECANOIC ACID, METHYL ESTER
Formula C17H34O2, MW 270, CAS# 112-39-0, Entry# 161288
METHYL HEXADECANOATE

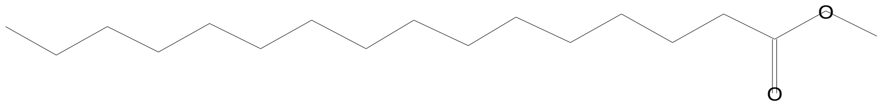

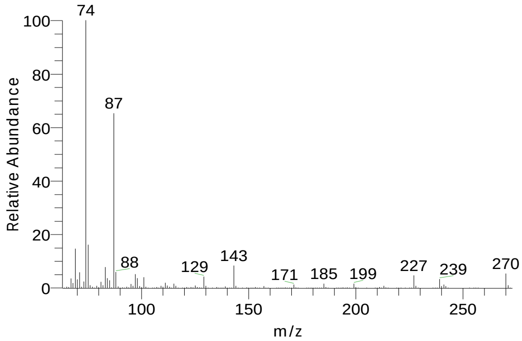

PENTADECANOIC ACID, 14-METHYL-, METHYL ESTER
Formula C17H34O2, MW 270, CAS# 5129-60-2, Entry# 161313
METHYL 14-METHYLPENTADECANOATE

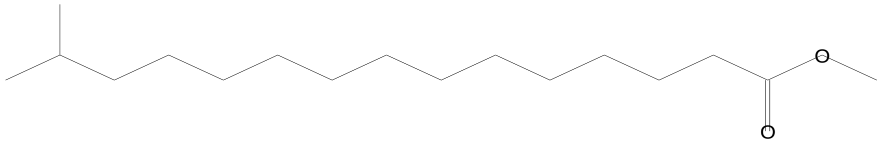

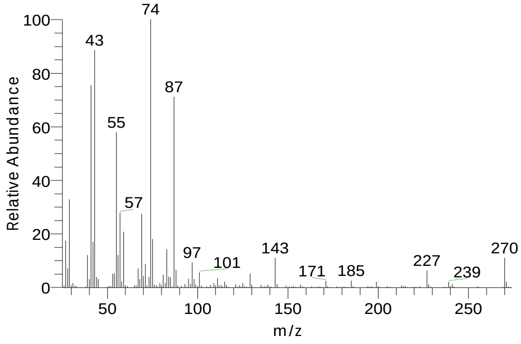

HEXADECANOIC ACID, METHYL ESTER
Formula C17H34O2, MW 270, CAS# 112-39-0, Entry# 161284
METHYL HEXADECANOATE

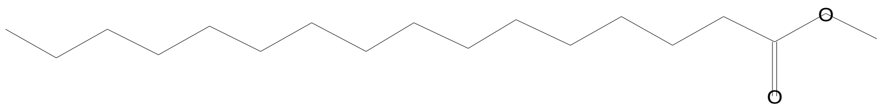

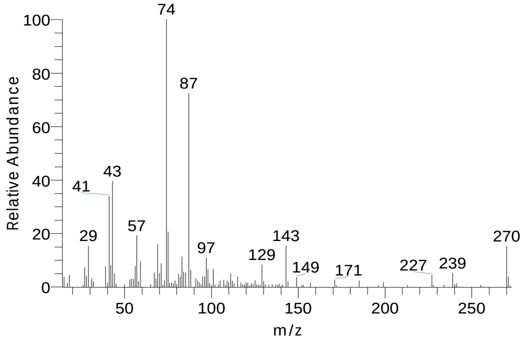

My GC-MS Report

Compound Structure | Hit Spectrum

Hexadecanoic acid, methyl ester
Formula C17H34O2, MW 270, CAS# 112-39-0, Entry# 10411
Palmitic acid, methyl ester

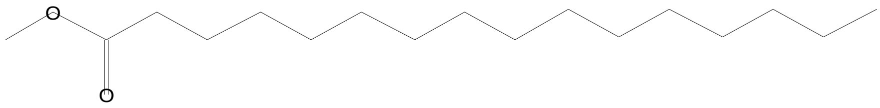

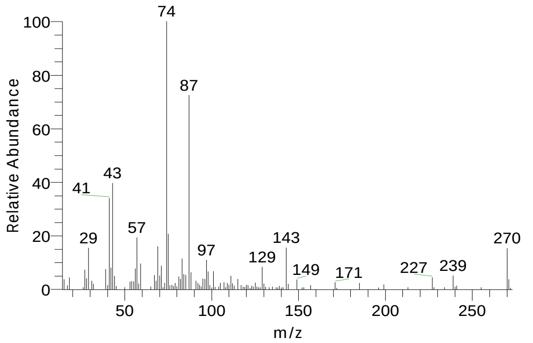

DrShreen_Mosturd #6697 RT: 26.46 AV: 1 NL: 3.36E6
T: + c EI Full ms [50.000-750.000]

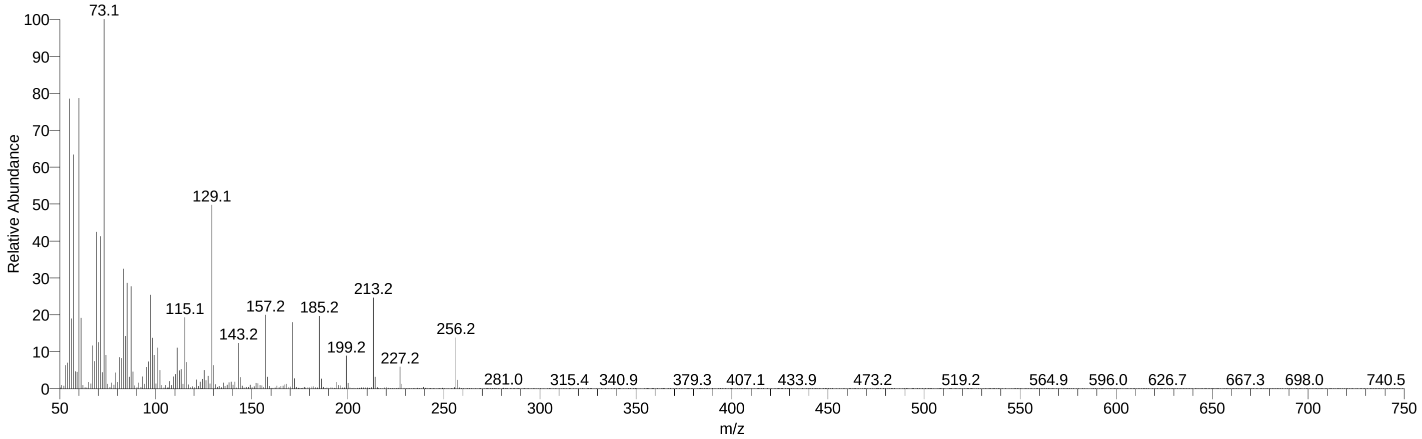

RT	Compound Name	Area %	MF	Molecular Formula	Molecular Weight	Cas #	Library
26.46	n-Hexadecanoic acid	16.29	932	C16H32O2	256	57-10-3	replib
26.46	HEXADECANOIC ACID	16.29	906	C16H32O2	256	57-10-3	WileyRegistry8e
26.46	HEXADECANOIC ACID	16.29	905	C16H32O2	256	57-10-3	WileyRegistry8e
26.46	n-Hexadecanoic acid	16.29	908	C16H32O2	256	57-10-3	replib
26.46	HEXADECANOIC ACID	16.29	886	C16H32O2	256	57-10-3	WileyRegistry8e

Compound Structure | Hit Spectrum

n-Hexadecanoic acid
Formula C16H32O2, MW 256, CAS# 57-10-3, Entry# 7566
Hexadecanoic acid

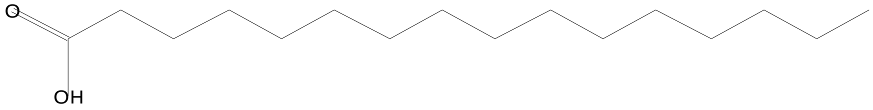

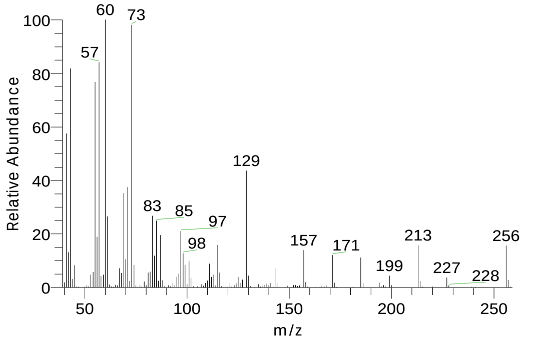

My GC-MS Report

Compound Structure | Hit Spectrum

HEXADECANOIC ACID
Formula C16H32O2, MW 256, CAS# 57-10-3, Entry# 397116
HEXADECANOATE

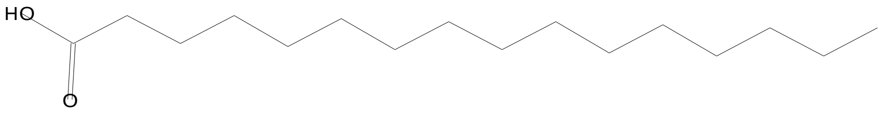

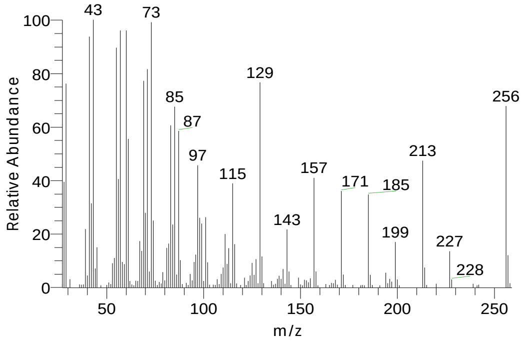

HEXADECANOIC ACID
Formula C16H32O2, MW 256, CAS# 57-10-3, Entry# 146744
HEXADECANOATE

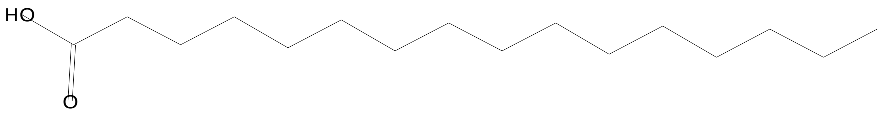

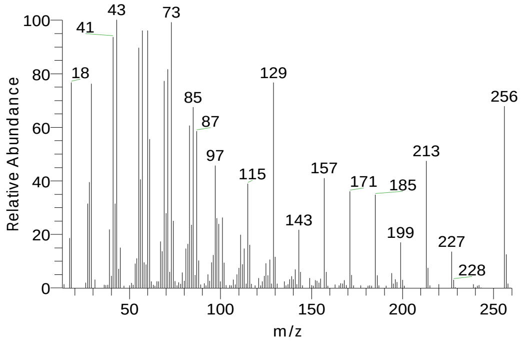

n-Hexadecanoic acid
Formula C16H32O2, MW 256, CAS# 57-10-3, Entry# 9622
Hexadecanoic acid

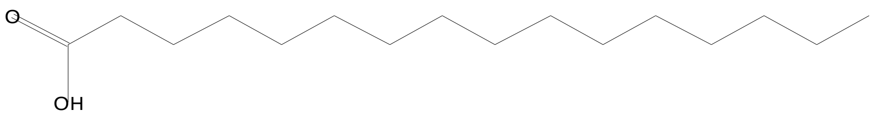

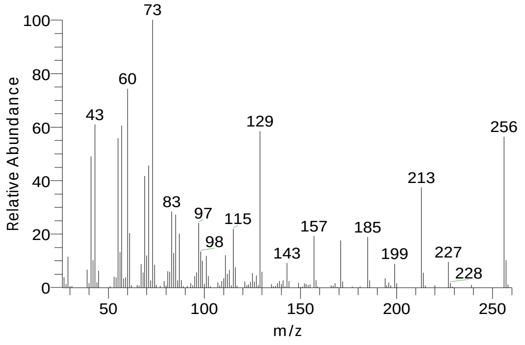

HEXADECANOIC ACID
Formula C16H32O2, MW 256, CAS# 57-10-3, Entry# 146746
HEXADECANOATE

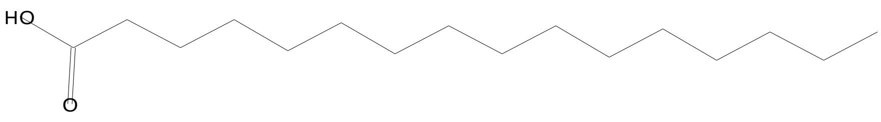

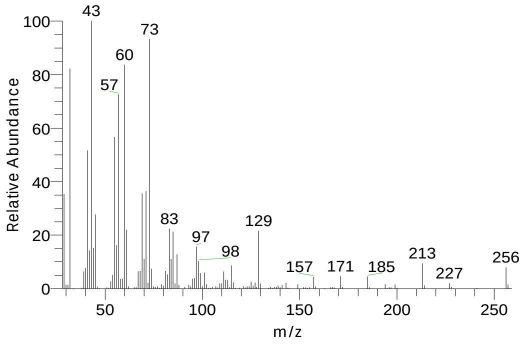

My GC-MS Report

DrShreen_Mosturd #7341 RT: 28.62 AV: 1 NL: 2.06E5
T: + c EI Full ms [50.000-750.000]

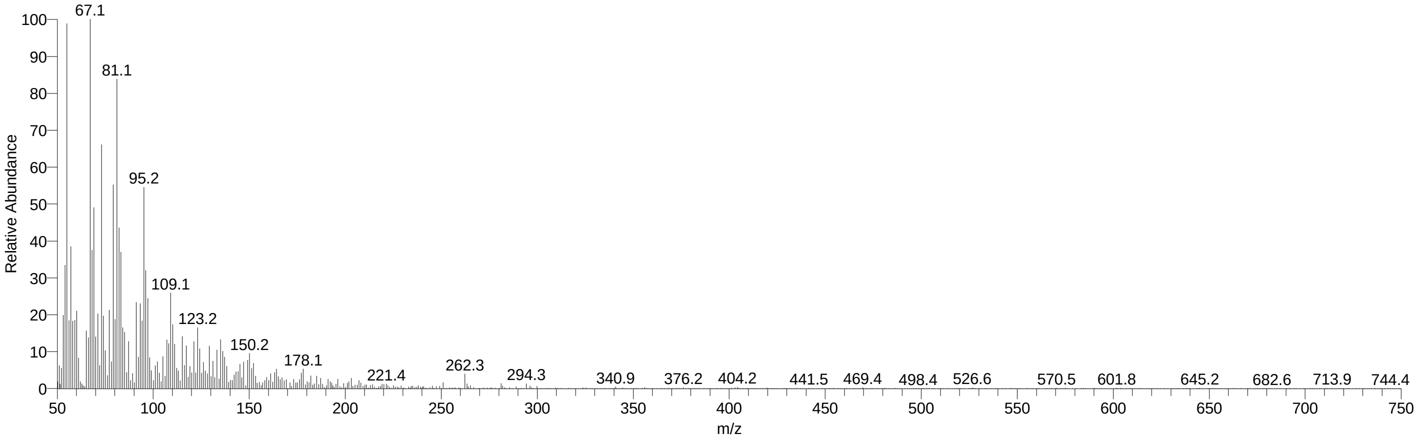

RT	Compound Name	Area %	MF	Molecular Formula	Molecular Weight	Cas #	Library
28.62	[1,1'-Bicyclopropyl]-2-octanoic acid, 2'-hexyl-, methyl ester	0.82	848	C21H38O2	322	56687-68-4	mainlib
28.62	[1,1'-BICYCLOPROPYL]-2-OCTANOIC ACID, 2'-HEXYL-, METHYL ESTER	0.82	848	C21H38O2	322	56687-68-4	WileyRegistry8e
28.62	HEXADECADIENOIC ACID, METHYL ESTER	0.82	808	C17H30O2	266	29961-54-4	WileyRegistry8e
28.62	Linoleic acid ethyl ester	0.82	847	C20H36O2	308	544-35-4	mainlib
28.62	ETHYL 9,12-OCTADECADIENOATE	0.82	847	C20H36O2	308	NA	WileyRegistry8e

Compound Structure	Hit Spectrum

[1,1'-Bicyclopropyl]-2-octanoic acid, 2'-hexyl-, methyl ester
Formula C21H38O2, MW 322, CAS# 56687-68-4, Entry# 40749
$:28BNXIGQHDTCPKMN-UHFFFAOYSA-N

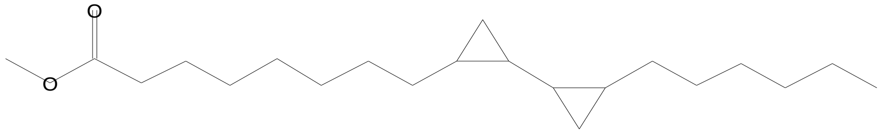

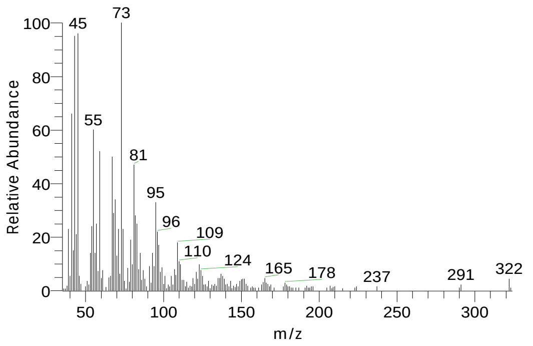

[1,1'-BICYCLOPROPYL]-2-OCTANOIC ACID, 2'-HEXYL-, METHYL ESTER
Formula C21H38O2, MW 322, CAS# 56687-68-4, Entry# 208698
METHYL 9,10,11,12-DIMETHYLENE OCTADECANOATE

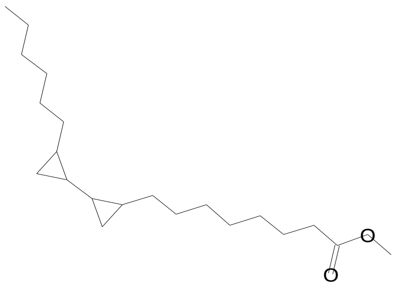

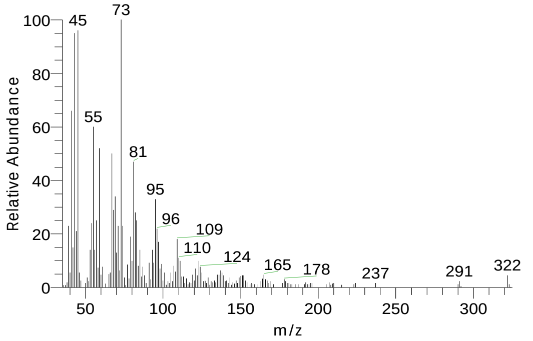

My GC-MS Report

Compound Structure | Hit Spectrum

HEXADECADIENOIC ACID, METHYL ESTER
Formula C17H30O2, MW 266, CAS# 29961-54-4, Entry# 157129
METHYL HEXADECADIENOATE

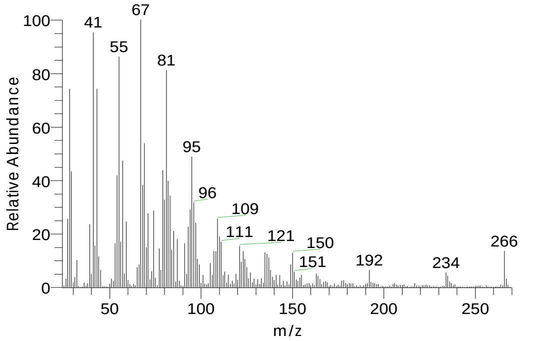

Linoleic acid ethyl ester
Formula C20H36O2, MW 308, CAS# 544-35-4, Entry# 32779
Ethyl linoleate

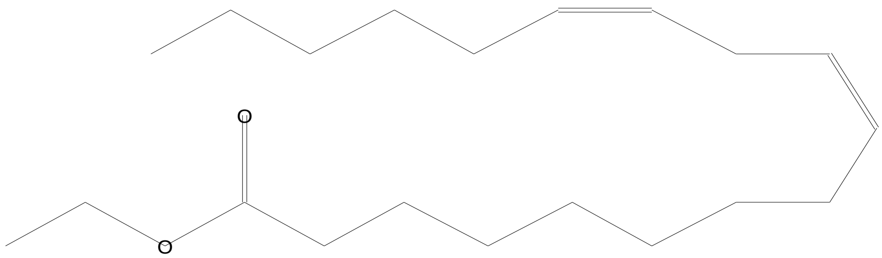

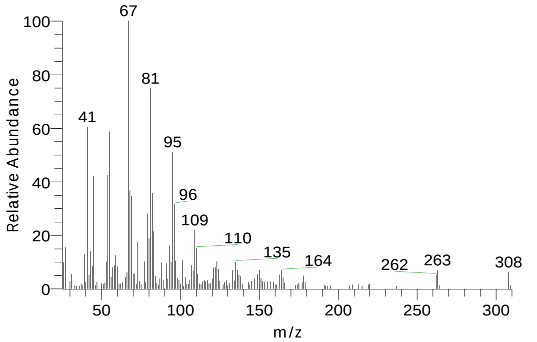

ETHYL 9,12-OCTADECADIENOATE
Formula C20H36O2, MW 308, CAS# NA, Entry# 388654
LINOLSAEURE, ETHYLESTER

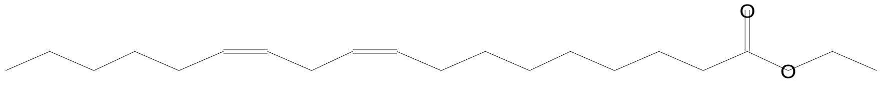

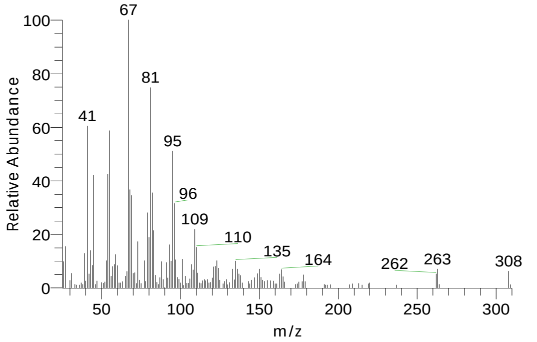

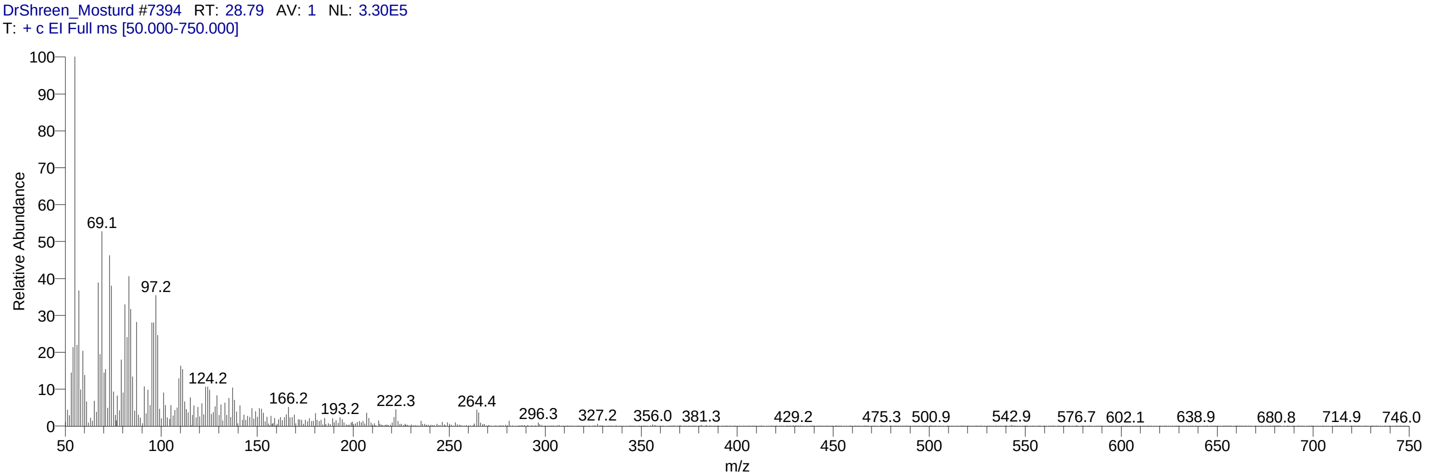

RT	Compound Name	Area %	MF	Molecular Formula	Molecular Weight	Cas #	Library
28.79	11-Octadecenoic acid, methyl ester	0.94	837	C19H36O2	296	52380-33-3	mainlib
28.79	11-OCTADECENOIC ACID, METHYL ESTER	0.94	837	C19H36O2	296	52380-33-3	WileyRegistry8e

My GC-MS Report

RT	Compound Name	Area %	MF	Molecular Formula	Molecular Weight	Cas #	Library
28.79	9-OCTADECENOIC ACID (Z)-	0.94	830	C18H34O2	282	112-80-1	WileyRegistry8e
28.79	9-Octadecenoic acid (Z)-, methyl ester	0.94	883	C19H36O2	296	112-62-9	replib
28.79	10-Octadecenoic acid, methyl ester	0.94	821	C19H36O2	296	13481-95-3	mainlib

Compound Structure Hit Spectrum

11-Octadecenoic acid, methyl ester
Formula C19H36O2, MW 296, CAS# 52380-33-3, Entry# 19082
Methyl 11-octadecenoate

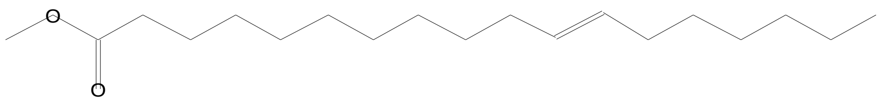

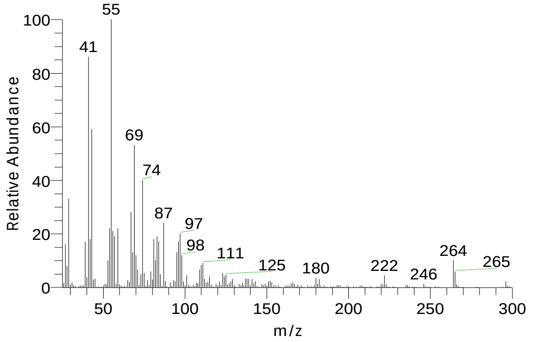

11-OCTADECENOIC ACID, METHYL ESTER
Formula C19H36O2, MW 296, CAS# 52380-33-3, Entry# 186176
OCTADEC-11-ENOIC ACID METHYL ESTER

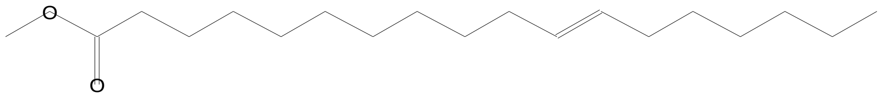

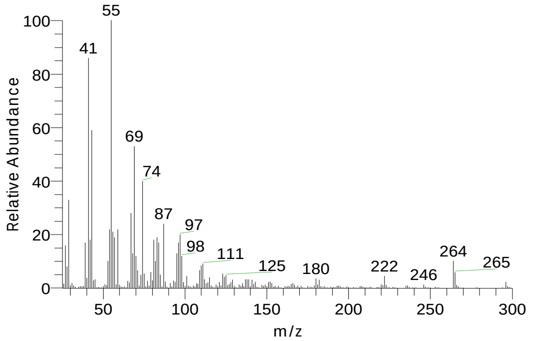

9-OCTADECENOIC ACID (Z)-
Formula C18H34O2, MW 282, CAS# 112-80-1, Entry# 172910
OCTADEC-9-ENOIC ACID

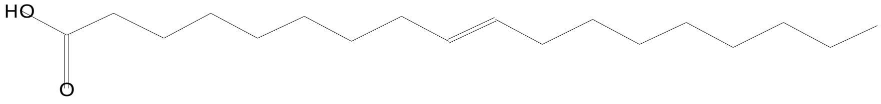

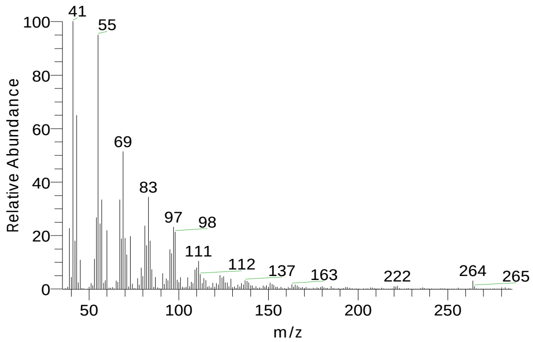

9-Octadecenoic acid (Z)-, methyl ester
Formula C19H36O2, MW 296, CAS# 112-62-9, Entry# 4747
Oleic acid, methyl ester

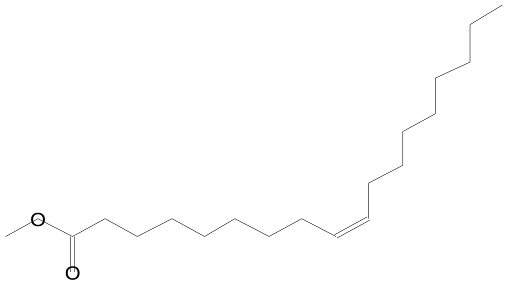

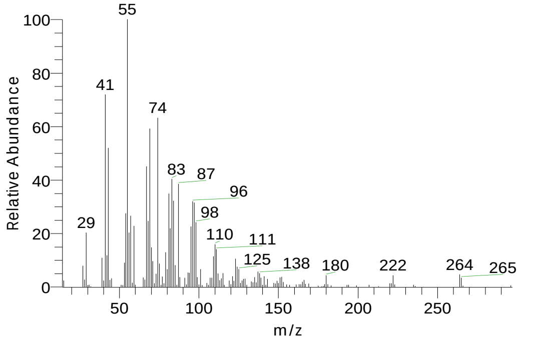

My GC-MS Report

Compound Structure | Hit Spectrum

10-Octadecenoic acid, methyl ester
Formula C19H36O2, MW 296, CAS# 13481-95-3, Entry# 19319
Methyl 10-octadecenoate

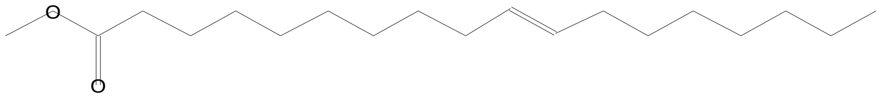

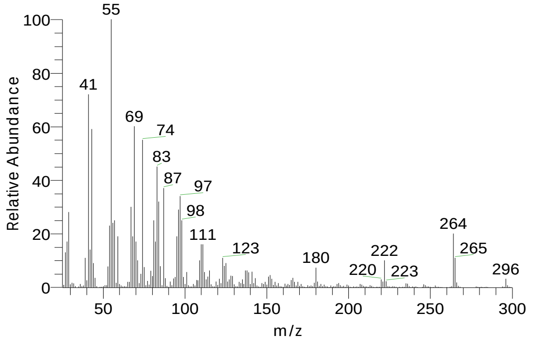

DrShreen_Mosturd #7583 RT: 29.43 AV: 1 NL: 1.49E6
T: + c EI Full ms [50.000-750.000]

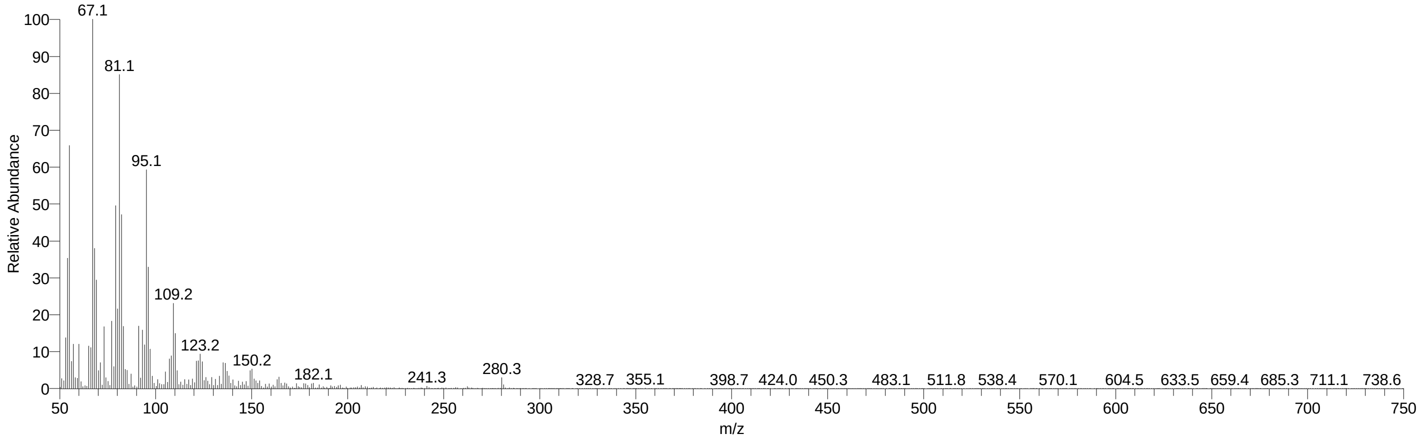

RT	Compound Name	Area %	MF	Molecular Formula	Molecular Weight	Cas #	Library
29.43	9,12-Octadecadienoic acid (Z,Z)-	8.62	912	C18H32O2	280	60-33-3	replib
29.43	9,12-Octadecadienoic acid (Z,Z)-	8.62	894	C18H32O2	280	60-33-3	replib
29.43	Linoelaidic acid	8.62	936	C18H32O2	280	506-21-8	mainlib
29.43	(Z)-18-Octadec-9-enolide	8.62	870	C18H32O2	280	80060-76-0	mainlib
29.43	ETHYL (9Z,12Z)-9,12-OCTADECADIENOATE #	8.62	880	C20H36O2	308	544-35-4	WileyRegistry8e

Compound Structure | Hit Spectrum

9,12-Octadecadienoic acid (Z,Z)-
Formula C18H32O2, MW 280, CAS# 60-33-3, Entry# 8129
cis-9,cis-12-Octadecadienoic acid

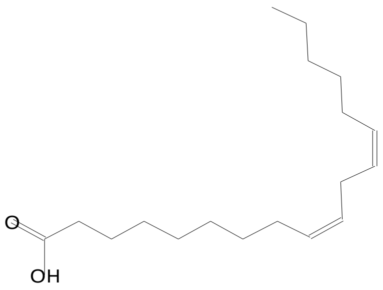

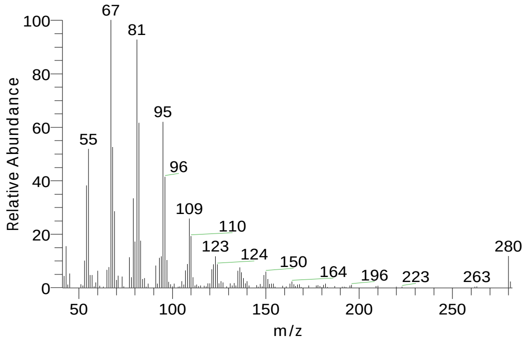

My GC-MS Report

Compound Structure | Hit Spectrum

9,12-Octadecadienoic acid (Z,Z)-
Formula C18H32O2, MW 280, CAS# 60-33-3, Entry# 8112
cis-9,cis-12-Octadecadienoic acid

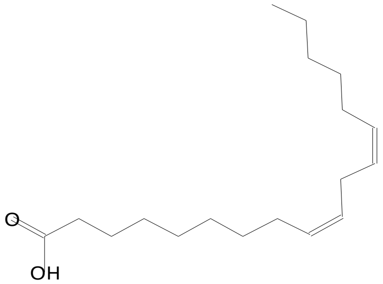

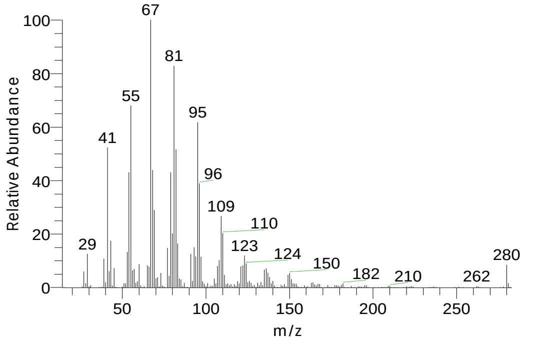

SI 864, RSI 936, mainlib, Entry# 32814, CAS# 506-21-8, Linoelaidic acid

Linoelaidic acid
Formula C18H32O2, MW 280, CAS# 506-21-8, Entry# 32814
$:28OYHQOLUKZRVURQ-AVQMFFATSA-N

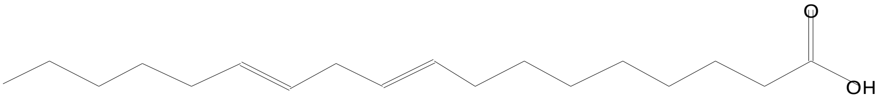

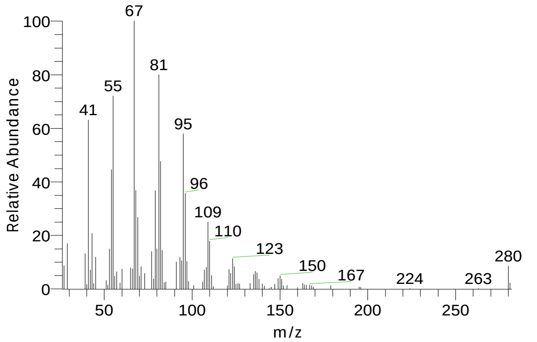

(Z)-18-Octadec-9-enolide
Formula C18H32O2, MW 280, CAS# 80060-76-0, Entry# 51421
Oxacyclononadec-10-en-2-one, (10Z)-

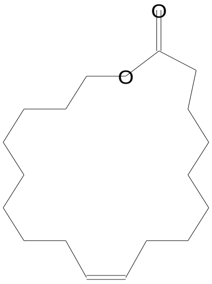

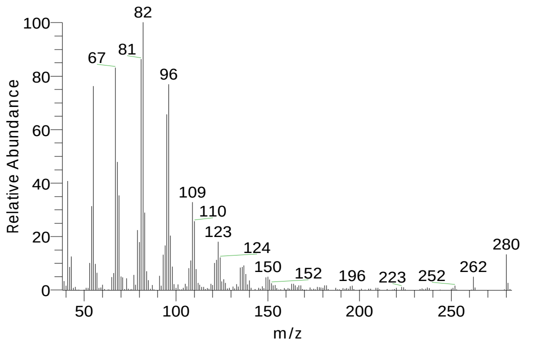

ETHYL (9Z,12Z)-9,12-OCTADECADIENOATE #
Formula C20H36O2, MW 308, CAS# 544-35-4, Entry# 196852
9,12-OCTADECADIENOIC ACID (9Z,12Z)-, ETHYL ESTER

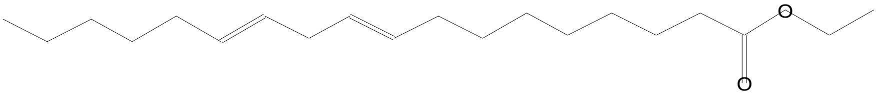

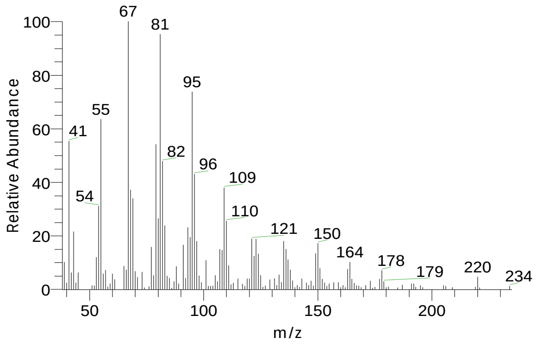

My GC-MS Report

DrShreen_Mosturd #7627 RT: 29.58 AV: 1 NL: 1.76E6
T: + c EI Full ms [50.000-750.000]

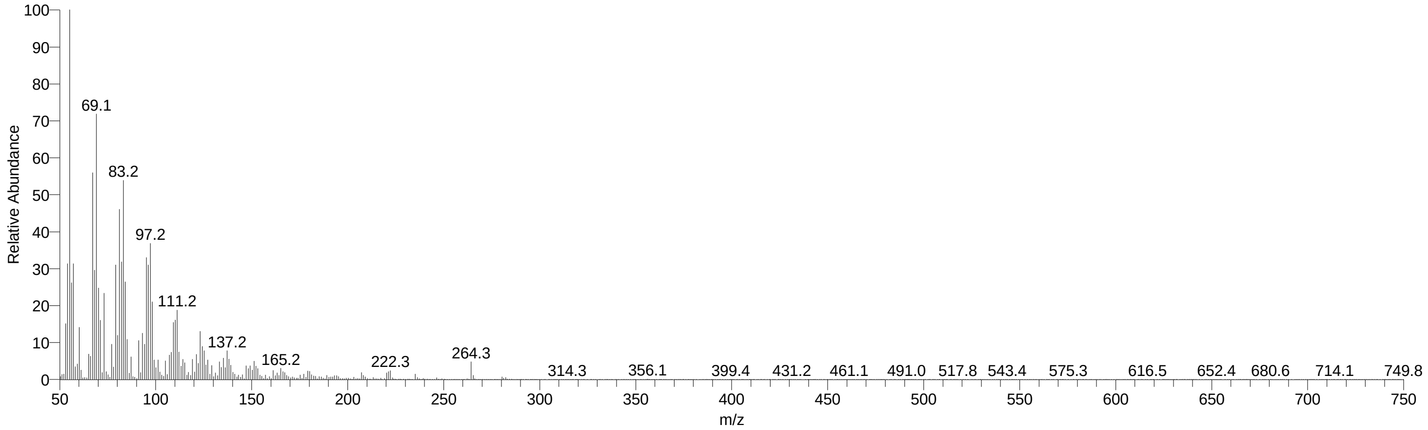

RT	Compound Name	Area %	MF	Molecular Formula	Molecular Weight	Cas #	Library
29.58	trans-13-Octadecenoic acid	9.14	897	C18H34O2	282	693-71-0	mainlib
29.58	cis-Vaccenic acid	9.14	892	C18H34O2	282	506-17-2	mainlib
29.58	cis-13-Octadecenoic acid	9.14	887	C18H34O2	282	13126-39-1	mainlib
29.58	9-OCTADECENOIC ACID (Z)-	9.14	885	C18H34O2	282	112-80-1	WileyRegistry8e
29.58	Oleic Acid	9.14	893	C18H34O2	282	112-80-1	replib

Compound Structure	Hit Spectrum

trans-13-Octadecenoic acid
Formula C18H34O2, MW 282, CAS# 693-71-0, Entry# 19306
$:28BDLLSHRIFPDGQB-AATRIKPKSA-N

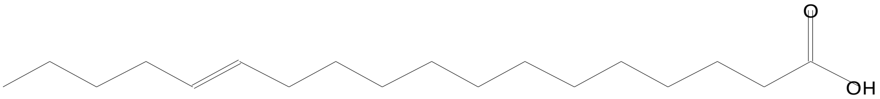

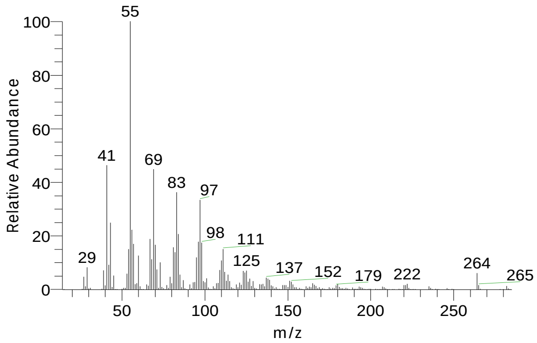

cis-Vaccenic acid
Formula C18H34O2, MW 282, CAS# 506-17-2, Entry# 20090
11-Octadecenoic acid, (Z)-

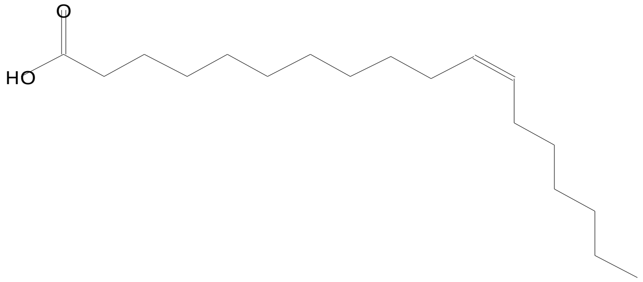

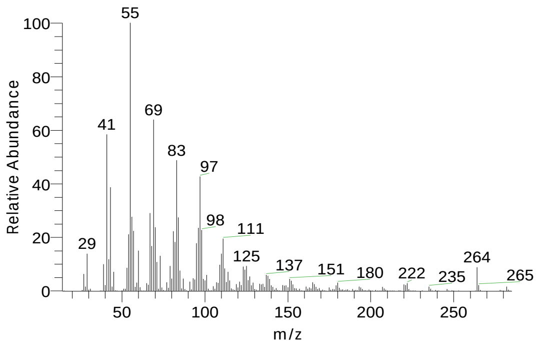

My GC-MS Report

Compound Structure | Hit Spectrum

cis-13-Octadecenoic acid
Formula C18H34O2, MW 282, CAS# 13126-39-1, Entry# 20126
$:28BDLLSHRIFPDGQB-WAYWQWQTSA-N

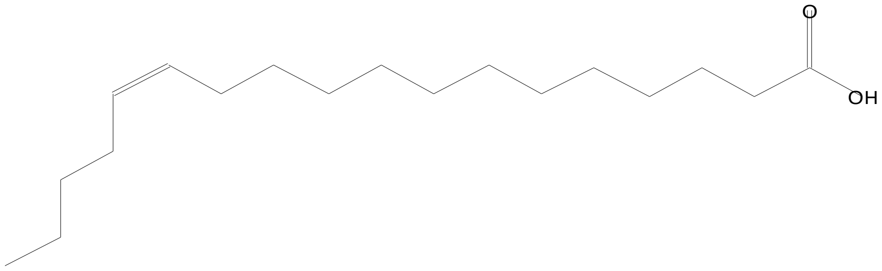

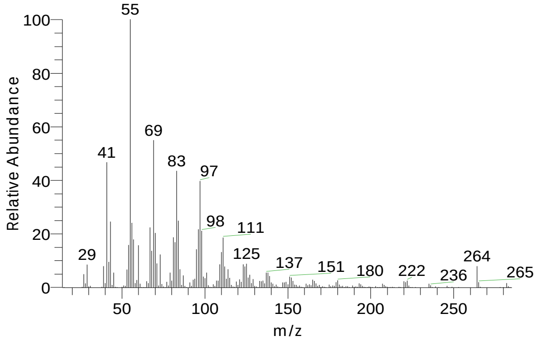

9-OCTADECENOIC ACID (Z)-
Formula C18H34O2, MW 282, CAS# 112-80-1, Entry# 172910
OCTADEC-9-ENOIC ACID

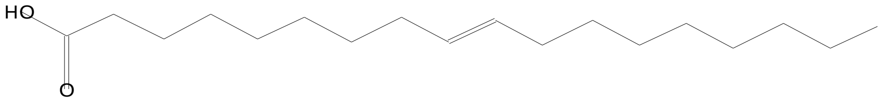

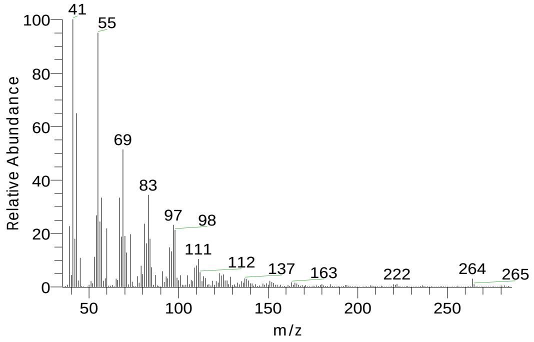

Oleic Acid
Formula C18H34O2, MW 282, CAS# 112-80-1, Entry# 5017
9-Octadecenoic acid (Z)-

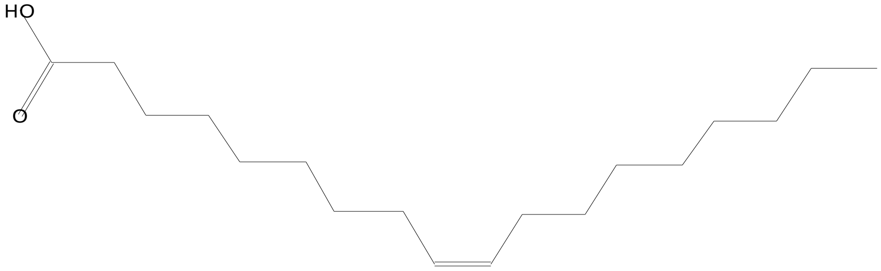

SI 883, RSI 893, replib, Entry# 5017, CAS# 112-80-1, Oleic Acid

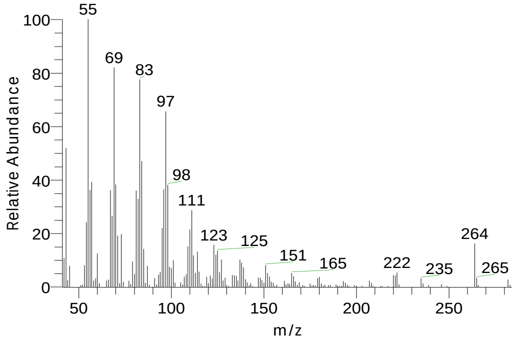

DrShreen_Mosturd #7773 RT: 30.06 AV: 1 NL: 5.48E5
T: + c EI Full ms [50.000-750.000]

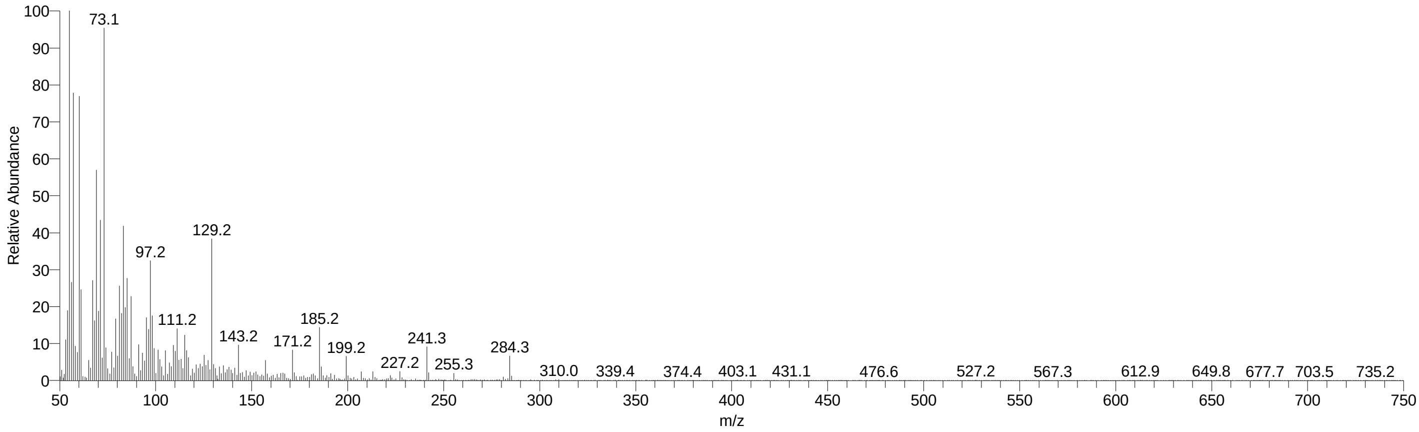

RT	Compound Name	Area %	MF	Molecular Formula	Molecular Weight	Cas #	Library
30.06	OCTADECANOIC ACID	2.40	834	C18H36O2	284	57-11-4	WileyRegistry8e
30.06	PENTADECANOIC ACID	2.40	827	C15H30O2	242	1002-84-2	WileyRegistry8e

My GC-MS Report

RT	Compound Name	Area %	MF	Molecular Formula	Molecular Weight	Cas #	Library
30.06	9-OCTADECENOIC ACID (Z)-	2.40	924	C18H34O2	282	112-80-1	WileyRegistry8e
30.06	Octadecanoic acid	2.40	897	C18H36O2	284	57-11-4	replib
30.06	STEARIC ACID	2.40	882	C18H36O2	284	NA	WileyRegistry8e

Compound Structure | Hit Spectrum

OCTADECANOIC ACID
Formula C18H36O2, MW 284, CAS# 57-11-4, Entry# 174897
STEARATE

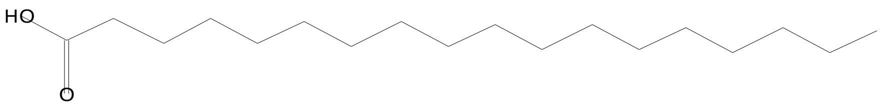

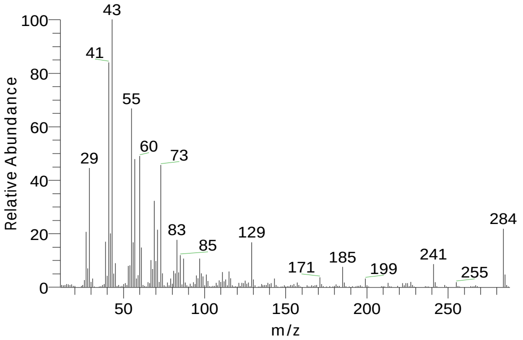

PENTADECANOIC ACID
Formula C15H30O2, MW 242, CAS# 1002-84-2, Entry# 131990
14FA

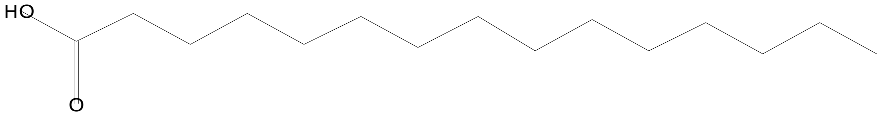

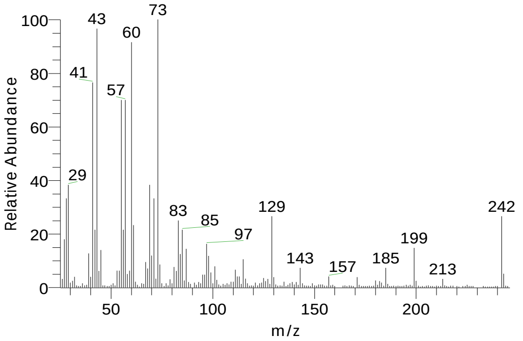

9-OCTADECENOIC ACID (Z)-
Formula C18H34O2, MW 282, CAS# 112-80-1, Entry# 172901
OCTADEC-9-ENOIC ACID

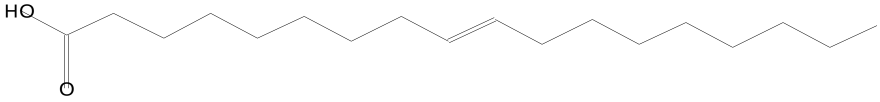

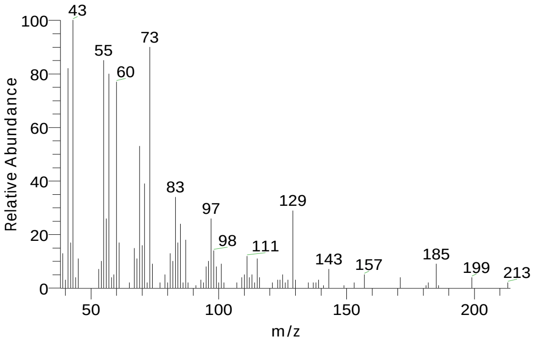

Octadecanoic acid
Formula C18H36O2, MW 284, CAS# 57-11-4, Entry# 2781
Stearic acid

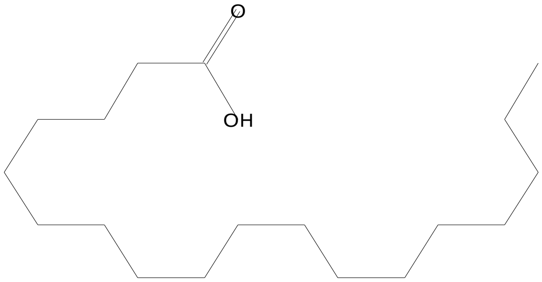

SI 791, RSI 897, replib, Entry# 2781, CAS# 57-11-4, Octadecanoic acid

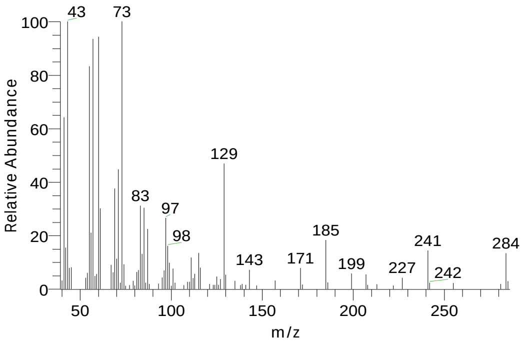

My GC-MS Report

Compound Structure | Hit Spectrum

STEARIC ACID
Formula C18H36O2, MW 284, CAS# NA, Entry# 359409
STEARINSAEURE

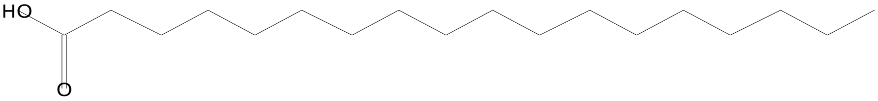

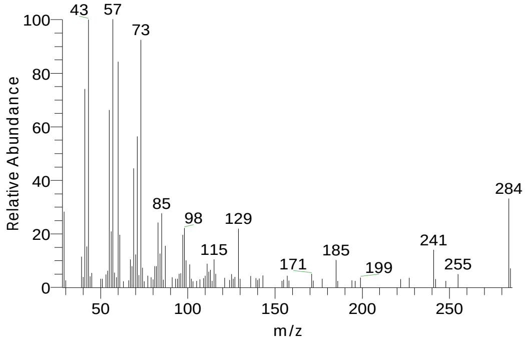

DrShreen_Mosturd #9485 RT: 35.81 AV: 1 NL: 7.43E5
T: + c EI Full ms [50.000-750.000]

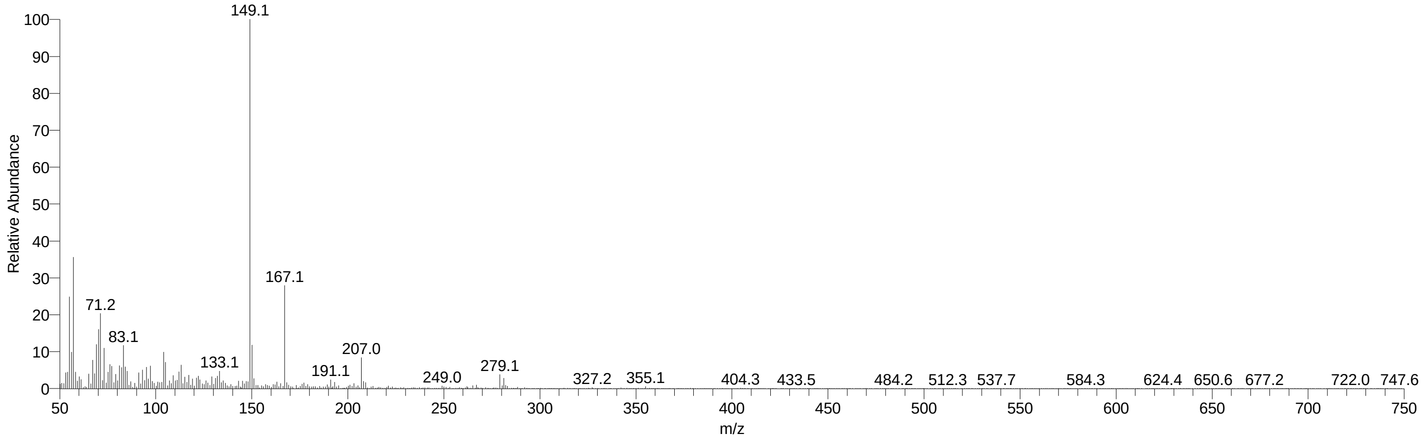

RT	Compound Name	Area %	MF	Molecular Formula	Molecular Weight	Cas #	Library
35.81	1,2-BENZENEDICARBOXYLIC ACID	1.07	773	C24H38O4	390	117-81-7	WileyRegistry8e
35.81	3',8,8'-Trimethoxy-3-piperidyl-2,2'-binaphthalene-1,1',4,4'-tetrone	1.07	740	C28H25NO7	487	127611-84-1	mainlib
35.81	9-(2',2'-Dimethylpropanoilhydrazono)-3,6-dichloro-2,7-bis-[2-(diethylamino)-ethoxy]fluorene	1.07	765	C30H42Cl2N4O3	576	NA	mainlib
35.81	1,2-BENZENEDICARBOXYLIC ACID	1.07	733	C24H38O4	390	117-81-7	WileyRegistry8e
35.81	2-([(2-ETHYLHEXYL)OXY]CARBONYL)BENZOIC ACID #	1.07	787	C16H22O4	278	4376-20-9	WileyRegistry8e

Compound Structure | Hit Spectrum

1,2-BENZENEDICARBOXYLIC ACID
Formula C24H38O4, MW 390, CAS# 117-81-7, Entry# 251626
1,2-BENZENEDICARBOXYLIC ACID, BIS(2-ETHYLHEXYL) ESTER

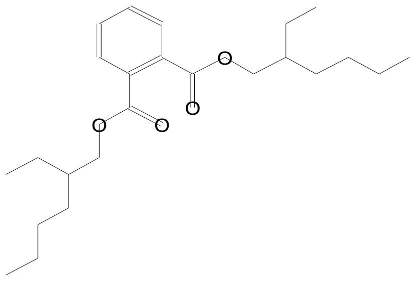

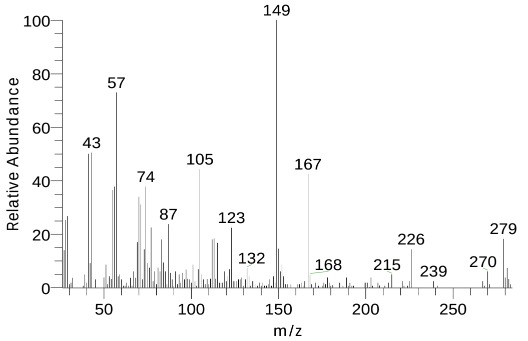

My GC-MS Report

Compound Structure | Hit Spectrum

3',8,8'-Trimethoxy-3-piperidyl-2,2'-binaphthalene-1,1',4,4'-tetrone
Formula C28H25NO7, MW 487, CAS# 127611-84-1, Entry# 137069
3',8,8'-Trimethoxy-3-piperidin-1-yl-2,2'-binaphthyl-1,1',4,4'-tetrone

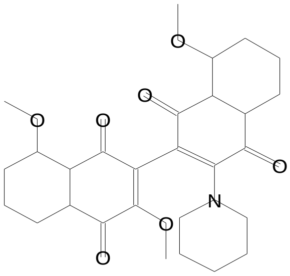

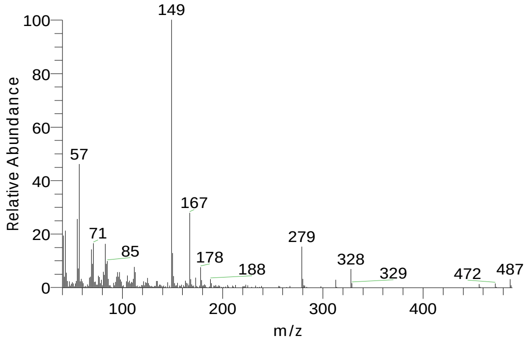

9-(2',2'-Dimethylpropanoilhydrazono)-3,6-dichloro-2,7-bis-[2-(diethylamino)-ethoxy]fluorene
Formula C30H42Cl2N4O3, MW 576, CAS# NA, Entry# 136954

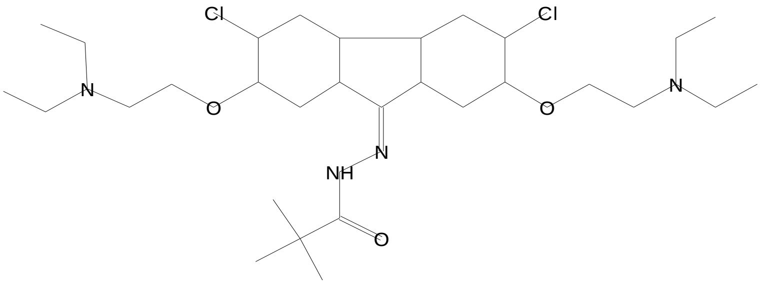

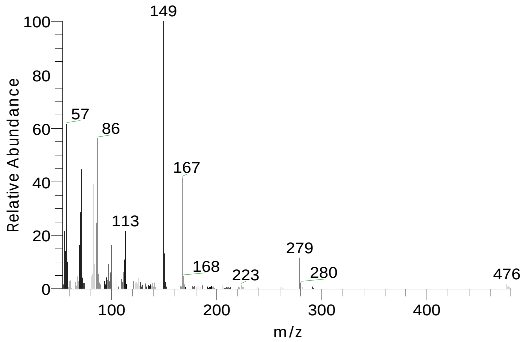

1,2-BENZENEDICARBOXYLIC ACID
Formula C24H38O4, MW 390, CAS# 117-81-7, Entry# 251627
1,2-BENZENEDICARBOXYLIC ACID, BIS(2-ETHYLHEXYL) ESTER

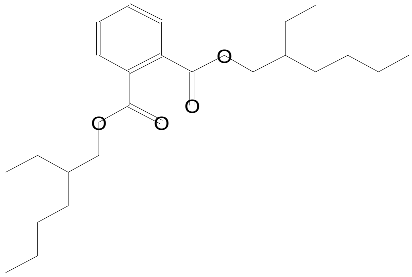

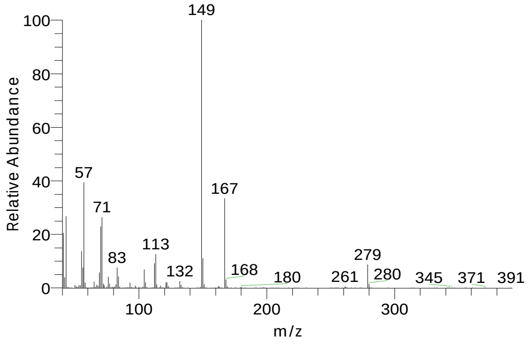

2-([(2-ETHYLHEXYL)OXY]CARBONYL)BENZOIC ACID #
Formula C16H22O4, MW 278, CAS# 4376-20-9, Entry# 168625
(2-ETHYLHEXYL) HYDROGEN PHTHALATE

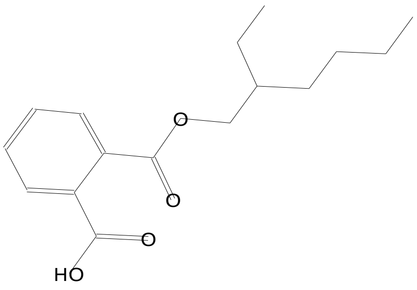

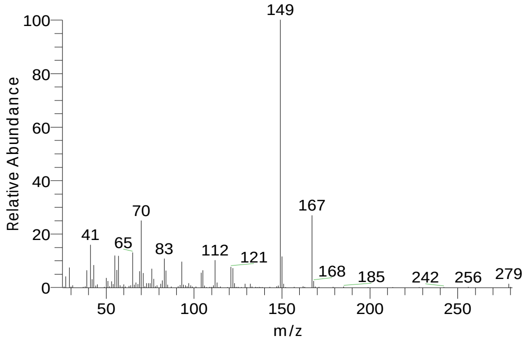

My GC-MS Report

DrShreen_Mosturd #9527 RT: 35.95 AV: 1 NL: 1.58E5
T: + c EI Full ms [50.000-750.000]

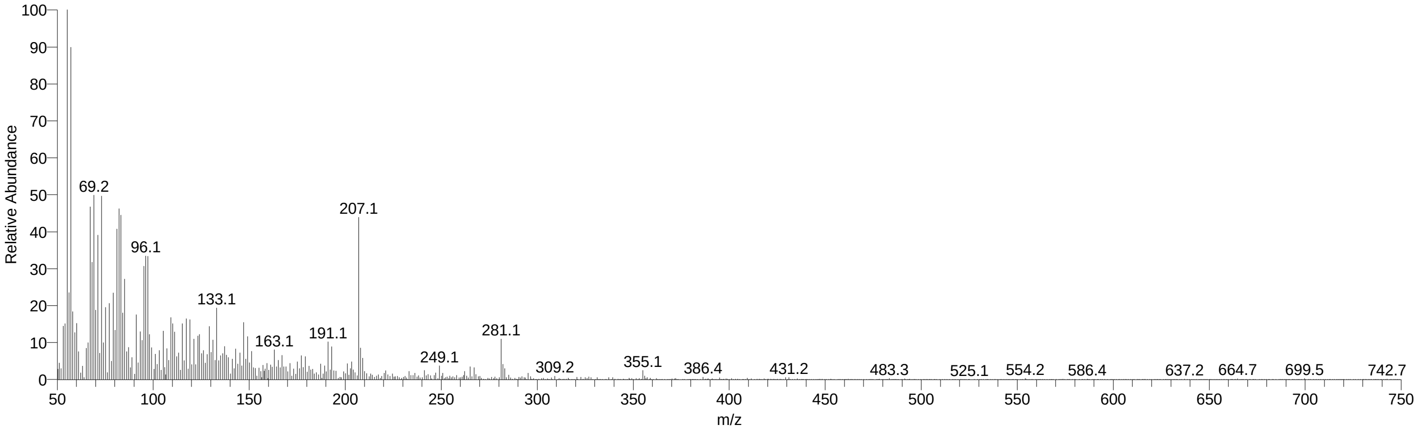

RT	Compound Name	Area %	MF	Molecular Formula	Molecular Weight	Cas #	Library
35.95	9,12,15-OCTADECATRIENOIC ACID, 2-[(TRIMETHYLSILYL)OXY]-1-[[(TRIMETHYLSILYL)OXY]METHYL]ETHYL ESTER, (Z,Z,Z)-	0.39	806	C27H52O4Si2	496	55521-23-8	WileyRegistry8e
35.95	9-OCTADECENOIC ACID (Z)-	0.39	737	C18H34O2	282	112-80-1	WileyRegistry8e
35.95	9,10 DIDEUTERO OCTADECANAL	0.39	743	C18H34D2O	270	56554-40-0	WileyRegistry8e
35.95	11-OCTADECENAL (SPECTRUM DISAGREES)	0.39	748	C18H34O	266	56554-95-1	WileyRegistry8e
35.95	2-HYDROXY-3-[(9E)-9-OCTADECENOYLOXY]PROPYL (9E)-9-OCTADECENOATE #	0.39	723	C39H72O5	620	2465-32-9	WileyRegistry8e

Compound Structure | Hit Spectrum

Formula C27H52O4Si2, MW 496, CAS# 55521-23-8, Entry# 284835

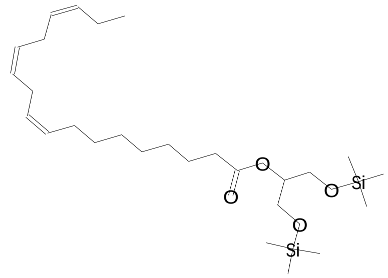

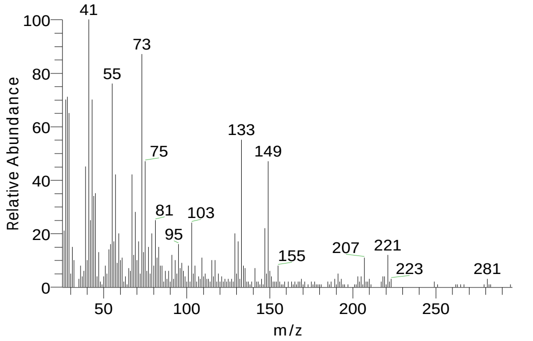

9-OCTADECENOIC ACID (Z)-
Formula C18H34O2, MW 282, CAS# 112-80-1, Entry# 172910
OCTADEC-9-ENOIC ACID

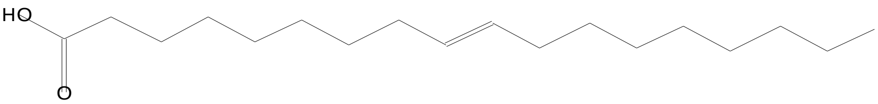

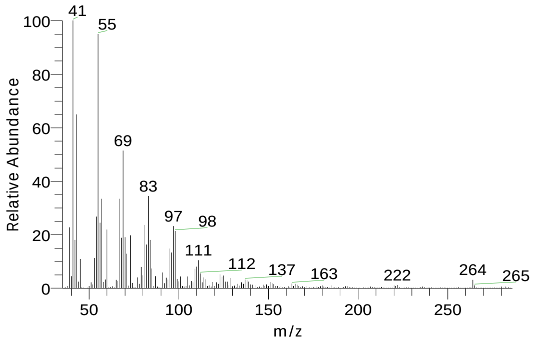

My GC-MS Report

Compound Structure | Hit Spectrum

9,10 DIDEUTERO OCTADECANAL
Formula C18H34D2O, MW 270, CAS# 56554-44-0, Entry# 159362
IDEUTERO OCTADECANAL

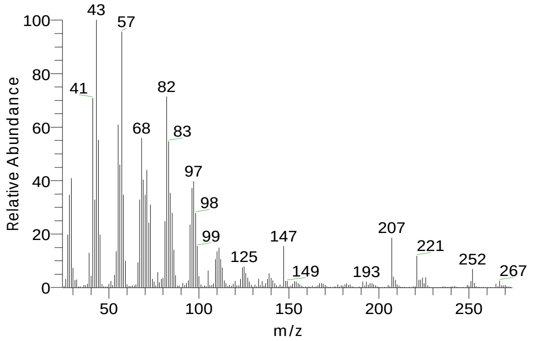

11-OCTADECENAL (SPECTRUM DISAGREES)
Formula C18H34O, MW 266, CAS# 56554-95-1, Entry# 157333
11-OCTADECENAL

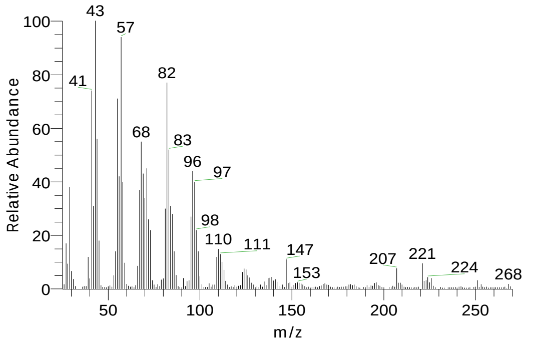

2-HYDROXY-3-[(9E)-9-OCTADECENOYLOXY]PROPYL (9E)-9-OCTADECENOATE #
Formula C39H72O5, MW 620, CAS# 2465-32-9, Entry# 298152
(Z,Z)-1,3-DIOCTADECENOYL GLYCEROL

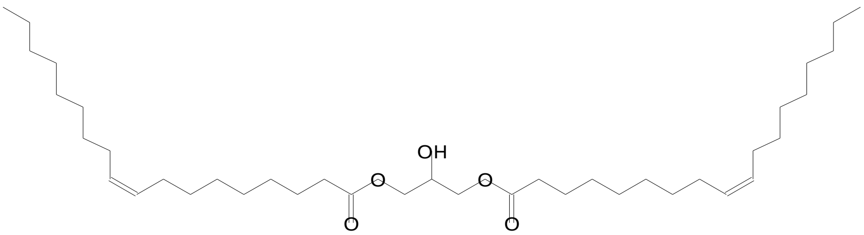

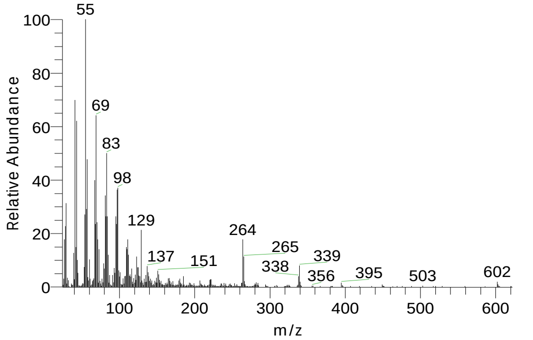

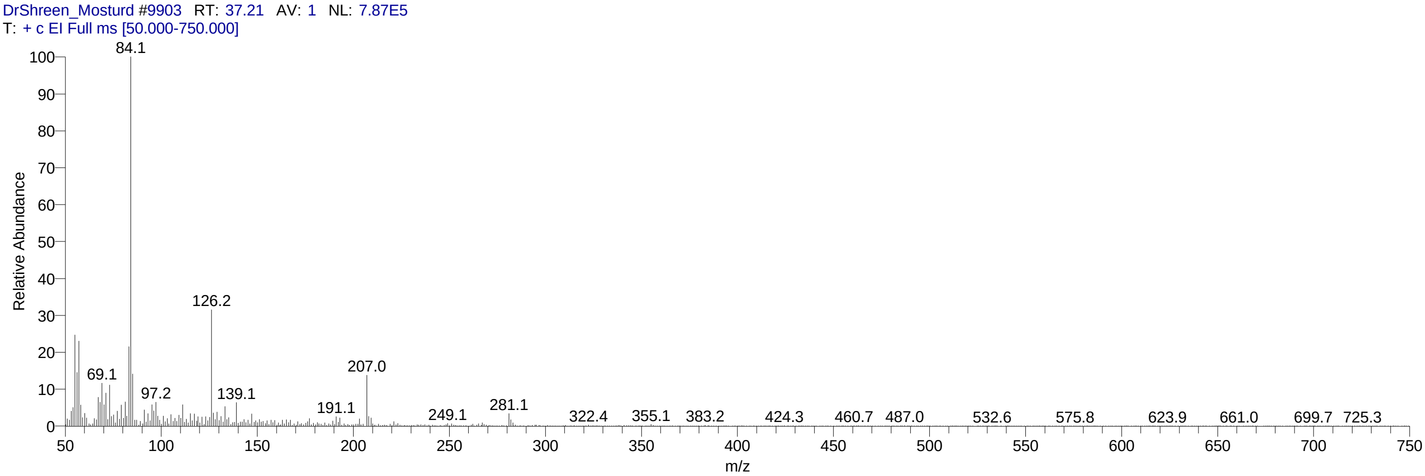

RT	Compound Name	Area %	MF	Molecular Formula	Molecular Weight	Cas #	Library
37.21	5-OCTADECENAL (SPECTRUM DISAGREES)	0.94	680	C18H34O	266	56554-88-2	WileyRegistry8e
37.21	5-Octadecenal	0.94	677	C18H34O	266	56554-88-2	mainlib

My GC-MS Report

RT	Compound Name	Area %	MF	Molecular Formula	Molecular Weight	Cas #	Library
37.21	4-OCTADECENAL (SPECTRUM DISAGREES)	0.94	672	C18H34O	266	56554-98-4	WileyRegistry8e
37.21	3-OCTADECENAL (SPECTRUM DISAGREES)	0.94	669	C18H34O	266	56554-99-5	WileyRegistry8e
37.21	4-Octadecenal	0.94	667	C18H34O	266	56554-98-4	mainlib

Compound Structure | Hit Spectrum

5-OCTADECENAL (SPECTRUM DISAGREES)
Formula C18H34O, MW 266, CAS# 56554-88-2, Entry# 157326
(5E)-5-OCTADECENAL #

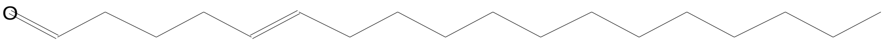

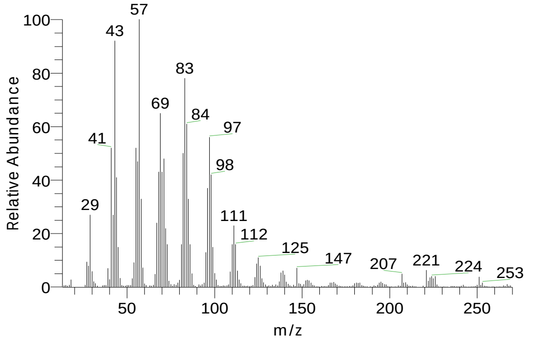

5-Octadecenal
Formula C18H34O, MW 266, CAS# 56554-88-2, Entry# 24442
(5E)-5-Octadecenal #

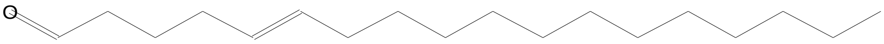

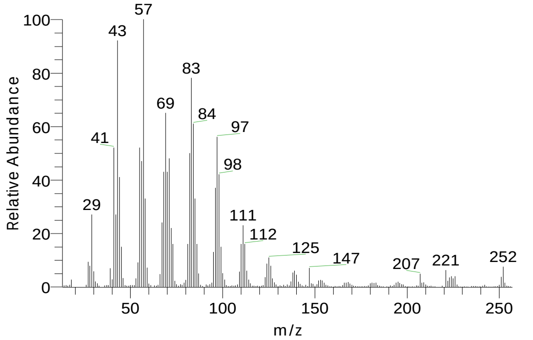

4-OCTADECENAL (SPECTRUM DISAGREES)
Formula C18H34O, MW 266, CAS# 56554-98-4, Entry# 157325
(4E)-4-OCTADECENAL #

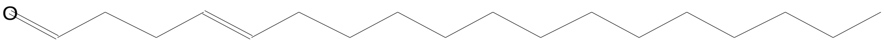

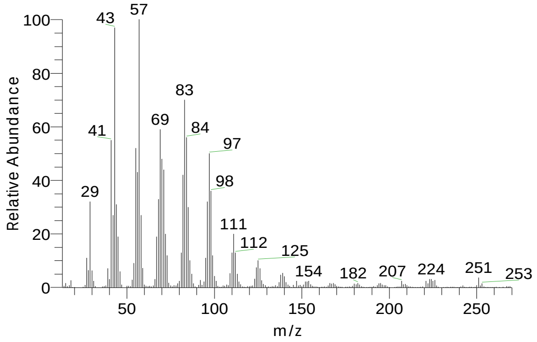

3-OCTADECENAL (SPECTRUM DISAGREES)
Formula C18H34O, MW 266, CAS# 56554-99-5, Entry# 157324
3-OCTADECENAL

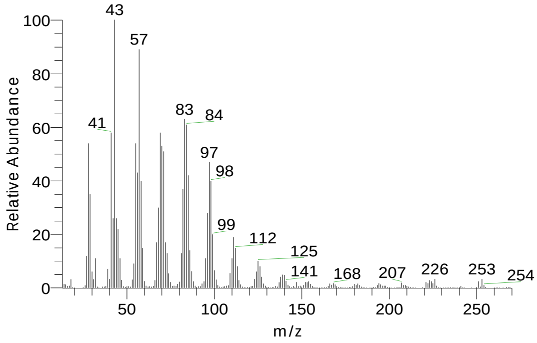

My GC-MS Report

Compound Structure | Hit Spectrum

4-Octadecenal
Formula C18H34O, MW 266, CAS# 56554-98-4, Entry# 24444
(4E)-4-Octadecenal #

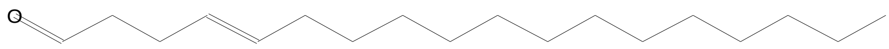

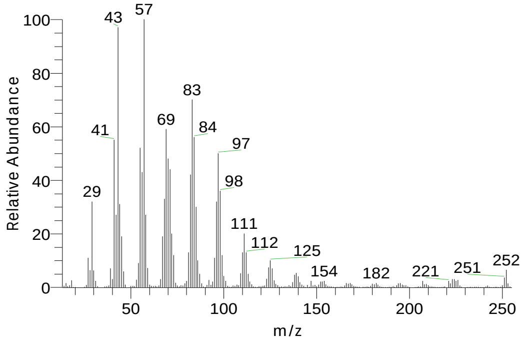

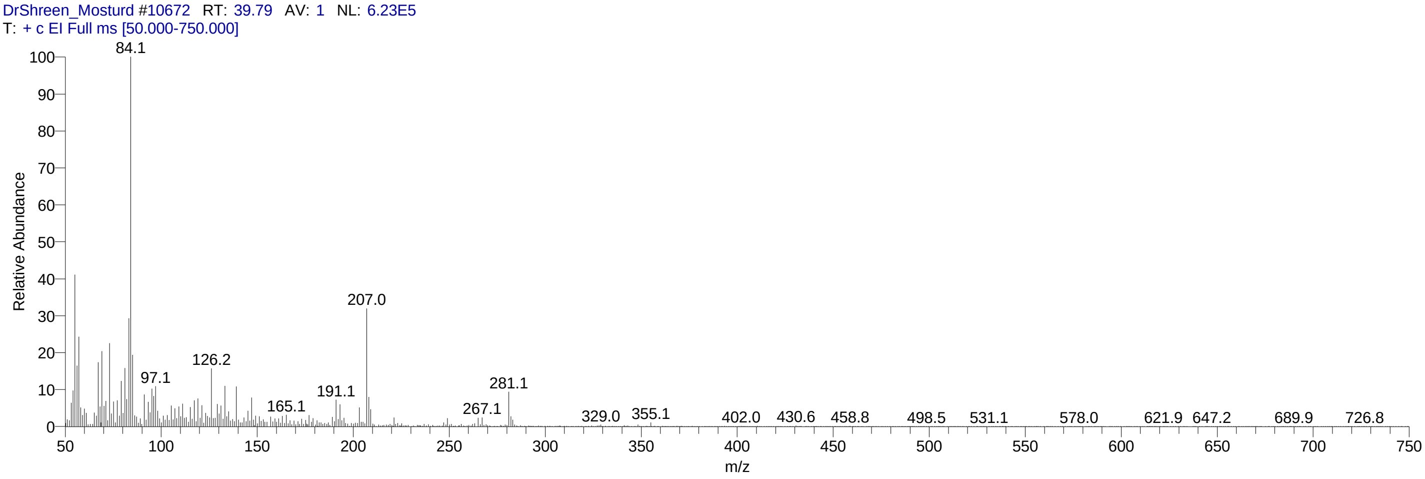

RT	Compound Name	Area %	MF	Molecular Formula	Molecular Weight	Cas #	Library
39.79	11-OCTADECENAL (SPECTRUM DISAGREES)	0.77	695	C18H34O	266	56554-95-1	WileyRegistry8e
39.79	Erucic acid	0.77	677	C22H42O2	338	112-86-7	replib
39.79	2-ACETYL-3-(2-BENZENESULPHONAMIDO)ETHYL-7-METHOXYINDOLE	0.77	662	C19H20N2O4S	372	NA	WileyRegistry8e
39.79	Oleic Acid	0.77	687	C18H34O2	282	112-80-1	replib
39.79	ARABINITOL, PENTAACETATE	0.77	688	C15H22O10	362	26674-23-7	WileyRegistry8e

Compound Structure | Hit Spectrum

11-OCTADECENAL (SPECTRUM DISAGREES)
Formula C18H34O, MW 266, CAS# 56554-95-1, Entry# 157333
11-OCTADECENAL

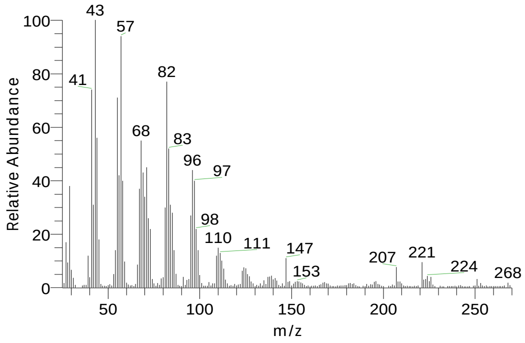

My GC-MS Report

Compound Structure | Hit Spectrum

Erucic acid
Formula C22H42O2, MW 338, CAS# 112-86-7, Entry# 5018
13-Docosenoic acid, (Z)-

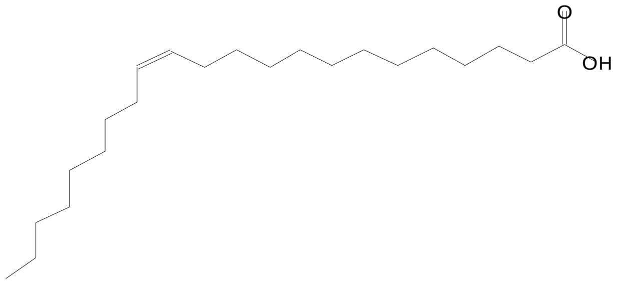

SI 637, RSI 677, replib, Entry# 5018, CAS# 112-86-7, Erucic acid

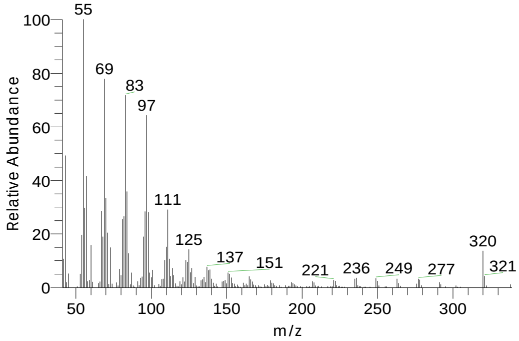

2-ACETYL-3-(2-BENZENESULPHONAMIDO)ETHYL-7-METHOXYINDOLE
Formula C19H20N2O4S, MW 372, CAS# NA, Entry# 241933

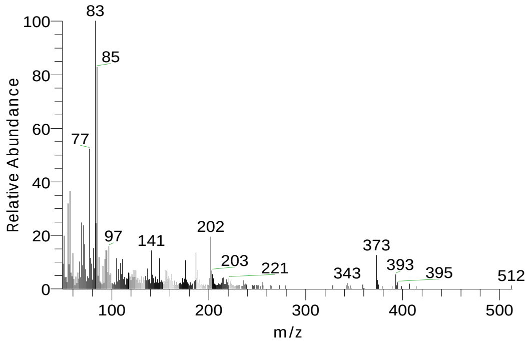

Oleic Acid
Formula C18H34O2, MW 282, CAS# 112-80-1, Entry# 5017
9-Octadecenoic acid (Z)-

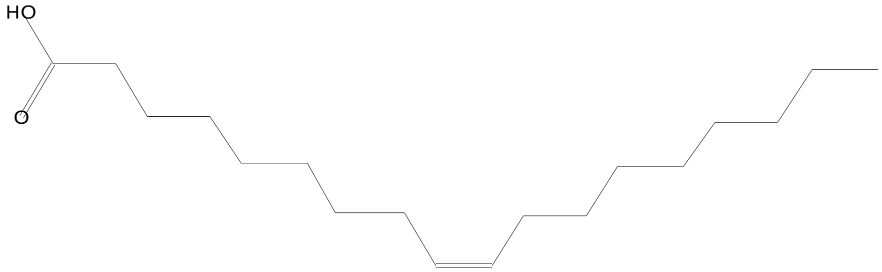

SI 634, RSI 687, replib, Entry# 5017, CAS# 112-80-1, Oleic Acid

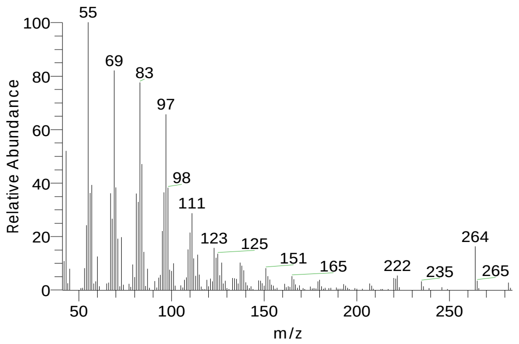

ARABINITOL, PENTAACETATE
Formula C15H22O10, MW 362, CAS# 26674-23-7, Entry# 235892
1,2,3,4,5-PENTA-O-ACETYLPENTITOL #

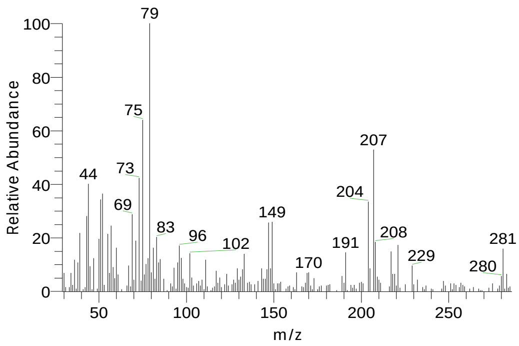

My GC-MS Report

DrShreen_Mosturd #10779 RT: 40.15 AV: 1 NL: 1.32E6
T: + c EI Full ms [50.000-750.000]

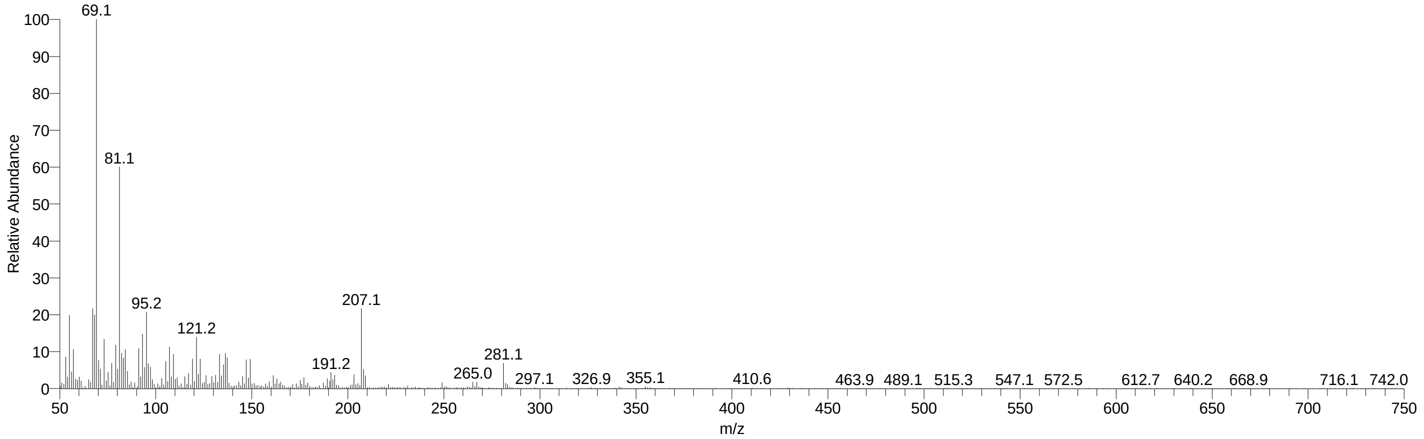

RT	Compound Name	Area %	MF	Molecular Formula	Molecular Weight	Cas #	Library
40.15	trans-Geranylgeraniol	1.69	793	C20H34O	290	24034-73-9	mainlib
40.15	2,2,4-Trimethyl-3-(3,8,12,16-tetramethyl-heptadeca-3,7,11,15-tetraenyl)-cyclohexanol	1.69	754	C30H52O	428	NA	mainlib
40.15	2,2,4-TRIMETHYL-3-(3,8,12,16-TETRAMETHYL-HEPTADECA-3,7,11,15-TETRAENYL)-CYCLOHEXANOL	1.69	754	C30H52O	428	NA	WileyRegistry8e
40.15	á-D-Mannofuranoside, farnesyl-	1.69	765	C21H36O6	384	NA	mainlib
40.15	3,7,11-TRIMETHYL-2,6,10-DODECATRIENYL HEXOFURANOSIDE	1.69	765	C21H36O6	384	NA	WileyRegistry8e

Compound Structure | Hit Spectrum

trans-Geranylgeraniol
Formula C20H34O, MW 290, CAS# 24034-73-9, Entry# 35392
2,6,10,14-Hexadecatetraen-1-ol, 3,7,11,15-tetramethyl-, (E,E,E)-

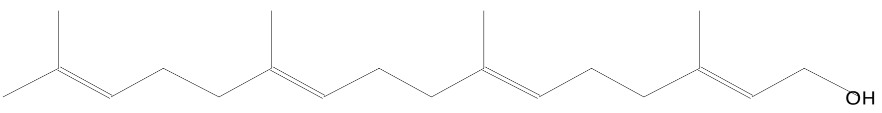

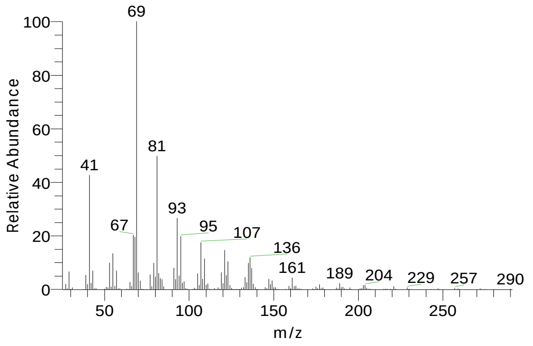

2,2,4-Trimethyl-3-(3,8,12,16-tetramethyl-heptadeca-3,7,11,15-tetraenyl)-cyclohexanol
Formula C30H52O, MW 428, CAS# NA, Entry# 35374

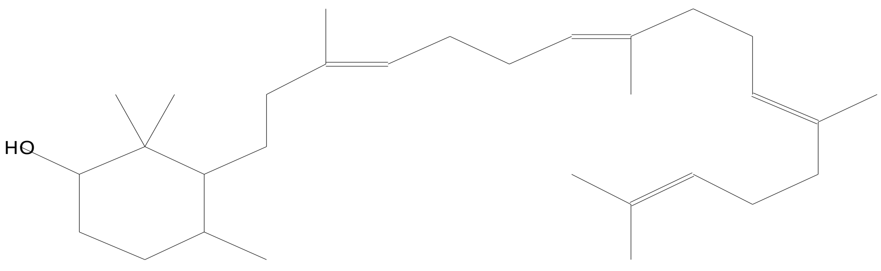

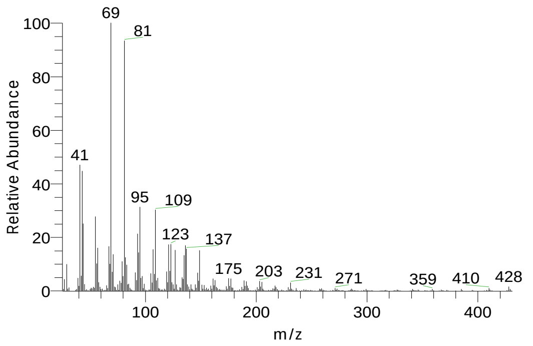

My GC-MS Report

Compound Structure	Hit Spectrum

Formula C30H52O, MW 428, CAS# NA, Entry# 367788
2,2,4-TRIMETHYL-3-[3,8,12,16-TETRAMETHYL-3,7,11,15-HEPTADECATETRAENYL]CYCLOHEXANOL

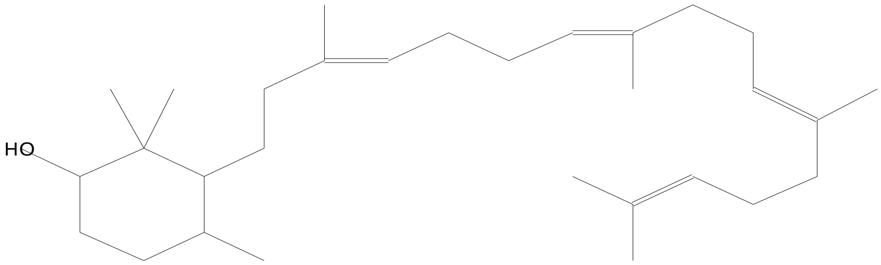

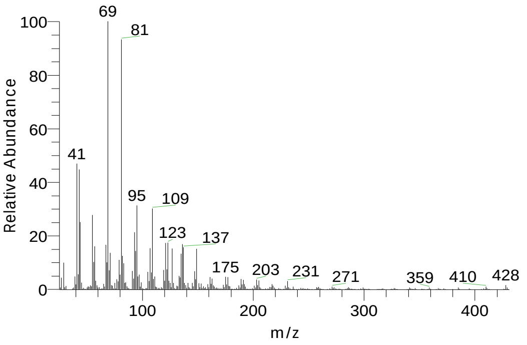

á-D-Mannofuranoside, farnesyl-
Formula C21H36O6, MW 384, CAS# NA, Entry# 34325
(2E,6Z)-3,7,11-Trimethyl-2,6,10-dodecatrienyl hexofuranoside #

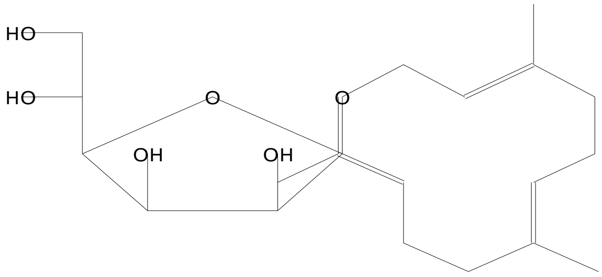

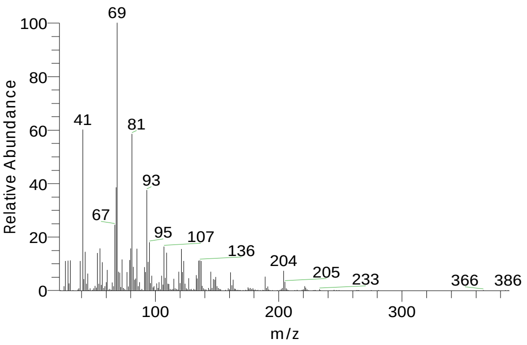

3,7,11-TRIMETHYL-2,6,10-DODECATRIENYL HEXOFURANOSIDE
Formula C21H36O6, MW 384, CAS# NA, Entry# 389191
BETA-D-MANNOFURANOSID, FARNESOL-

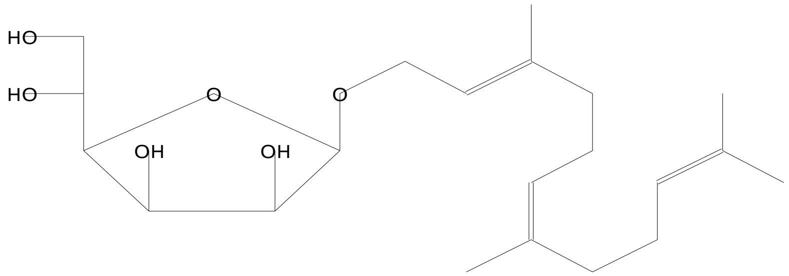

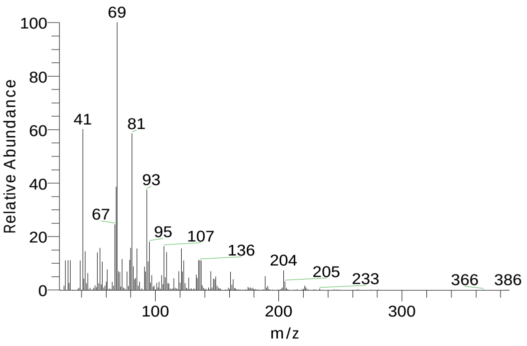

DrShreen_Mosturd #11206 RT: 41.58 AV: 1 NL: 6.45E5
T: + c EI Full ms [50.000-750.000]

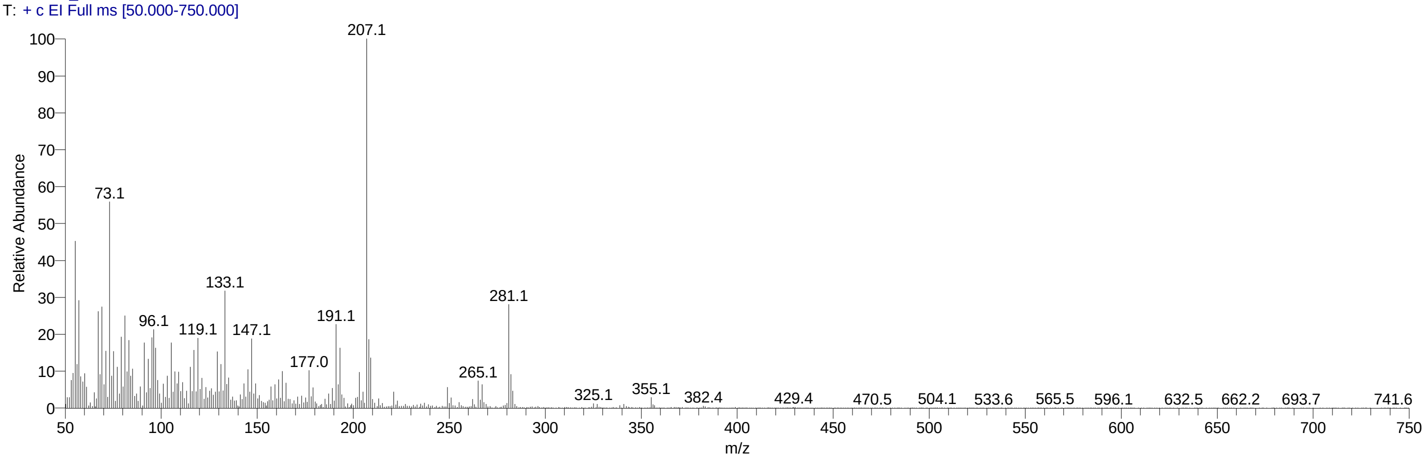

RT	Compound Name	Area %	MF	Molecular Formula	Molecular Weight	Cas #	Library
41.58	9,12-OCTADECADIENOIC ACID (Z,Z)-, 2,3-BIS[(TRIMETHYLSILYL)OXY]PROPYL ESTER	0.33	757	C27H54O4Si2	498	54284-45-6	WileyRegistry8e

My GC-MS Report

RT	Compound Name	Area %	MF	Molecular Formula	Molecular Weight	Cas #	Library
41.58	TRISTRIMETHYLSILYL ETHER DERIVATIVE OF 1,25-DIHYDROXYVITAMIN D2	0.33	712	C37H68O3Si3	644	NA	WileyRegistry8e
41.58	4H-1-BENZOPYRAN-4-ONE, 2-(3,4-DIMETHOXYPHENYL)-3,5-DIHYDROXY-7-METHOXY-	0.33	686	C18H16O7	344	6068-80-0	WileyRegistry8e
41.58	1,25-Dihydroxyvitamin D3, TMS derivative	0.33	731	C30H52O3Si	488	55759-94-9	mainlib
41.58	9,10-SECOCHOLESTA-5,7,10(19)-TRIENE-1,3-DIOL, 25-[(TRIMETHYLSILYL)OXY]-, (3á,5Z,7E)-	0.33	731	C30H52O3Si	488	55759-94-9	WileyRegistry8e

Compound Structure	Hit Spectrum

9,12-OCTADECADIENOIC ACID (Z,Z)-, 2,3-BIS[(TRIMETHYLSILYL)OXY]PROPYL ESTER
Formula C27H54O4Si2, MW 498, CAS# 54284-45-6, Entry# 285148
2,3-BIS[(TRIMETHYLSILYL)OXY]PROPYL (9Z,12Z)-9,12-OCTADECADIENOATE #

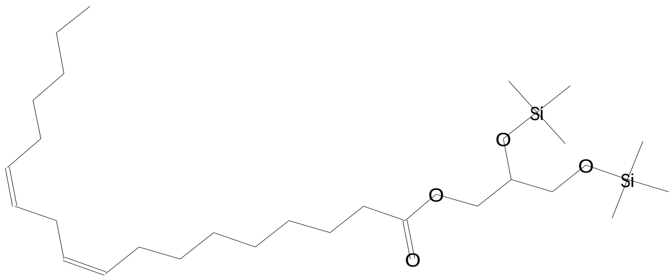

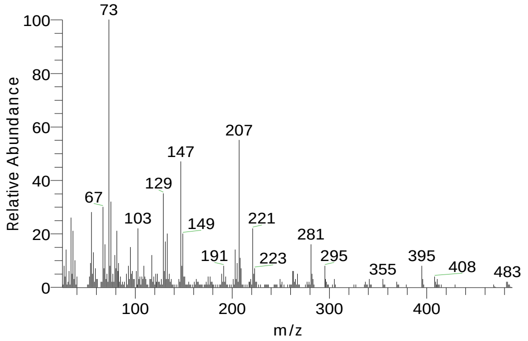

TRISTRIMETHYLSILYL ETHER DERIVATIVE OF 1,25-DIHYDROXYVITAMIN D2
Formula C37H68O3Si3, MW 644, CAS# NA, Entry# 299431

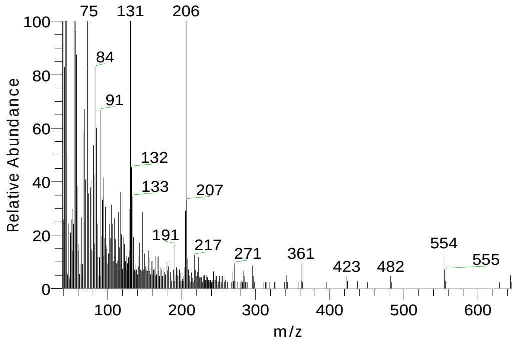

4H-1-BENZOPYRAN-4-ONE, 2-(3,4-DIMETHOXYPHENYL)-3,5-DIHYDROXY-7-METHOXY-
Formula C18H16O7, MW 344, CAS# 6068-80-0, Entry# 224392
3',4',7-TRIMETHYLQUERCETIN

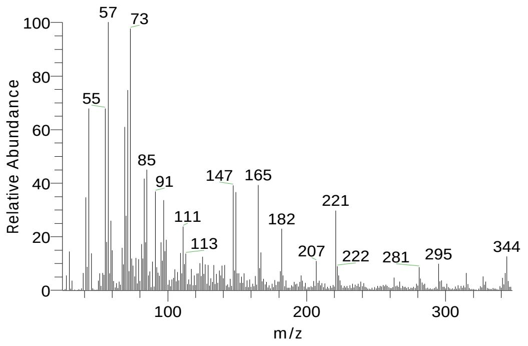

My GC-MS Report

Compound Structure | Hit Spectrum

1,25-Dihydroxyvitamin D3, TMS derivative
Formula C30H52O3Si, MW 488, CAS# 55759-94-9, Entry# 19952
9,10-Secocholesta-5,7,10(19)-triene-1,3-diol, 25-[(trimethylsilyl)oxy]-, (3á,5Z,7E)-

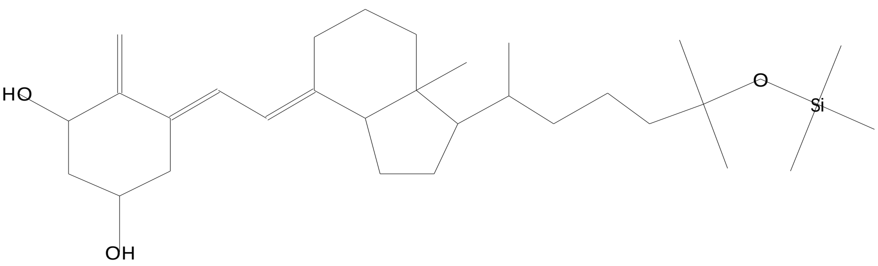

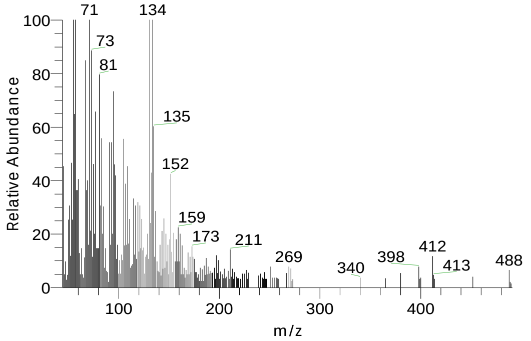

9,10-SECOCHOLESTA-5,7,10(19)-TRIENE-1,3-DIOL, 25-[(TRIMETHYLSILYL)OXY]-, (3á,5Z,7E)-
Formula C30H52O3Si, MW 488, CAS# 55759-94-9, Entry# 283552
(5E,7E)-25-[(TRIMETHYLSILYL)OXY]-9,10-SECOCHOLESTA-5,7,10-TRIENE-1,3-DIOL #

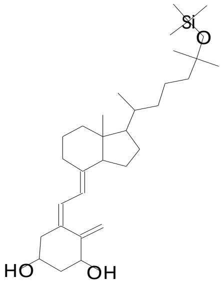

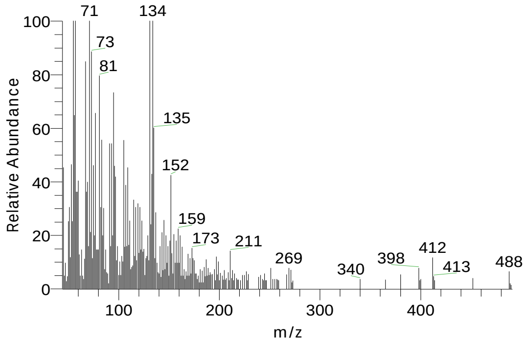

DrShreen_Mosturd #11491 RT: 42.53 AV: 1 NL: 6.21E5
T: + c EI Full ms [50.000-750.000]

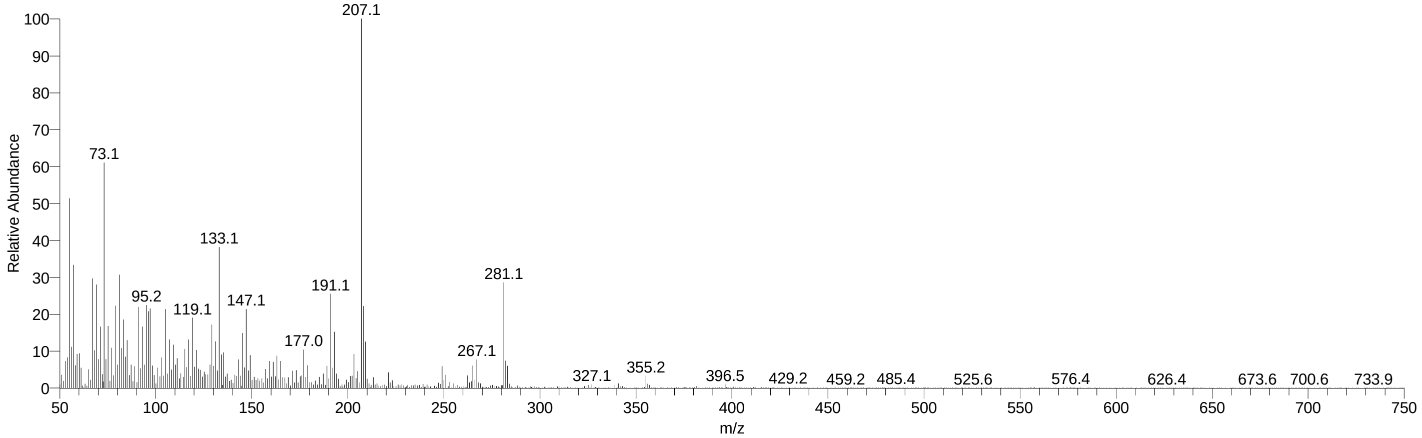

RT	Compound Name	Area %	MF	Molecular Formula	Molecular Weight	Cas #	Library
42.53	9,12-OCTADECADIENOIC ACID (Z,Z)-, 2,3-BIS[(TRIMETHYLSILYL)OXY]PROPYL ESTER	0.49	773	C27H54O4Si2	498	54284-45-6	WileyRegistry8e
42.53	TRISTRIMETHYLSILYL ETHER DERIVATIVE OF 1,25-DIHYDROXYVITAMIN D2	0.49	727	C37H68O3Si3	644	NA	WileyRegistry8e
42.53	Ethyl iso-allocholate	0.49	706	C26H44O5	436	NA	mainlib
42.53	ETHYL ISO-ALLOCHOLATE	0.49	706	C26H44O5	436	NA	WileyRegistry8e
42.53	1-Heptatriacotanol	0.49	723	C37H76O	536	105794-58-9	mainlib

My GC-MS Report

Compound Structure	Hit Spectrum

9,12-OCTADECADIENOIC ACID (Z,Z)-, 2,3-BIS[(TRIMETHYLSILYL)OXY]PROPYL ESTER
Formula C27H54O4Si2, MW 498, CAS# 54284-45-6, Entry# 285148
2,3-BIS[(TRIMETHYLSILYL)OXY]PROPYL (9Z,12Z)-9,12-OCTADECADIENOATE #

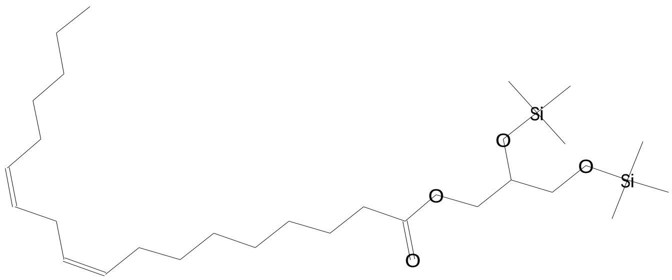

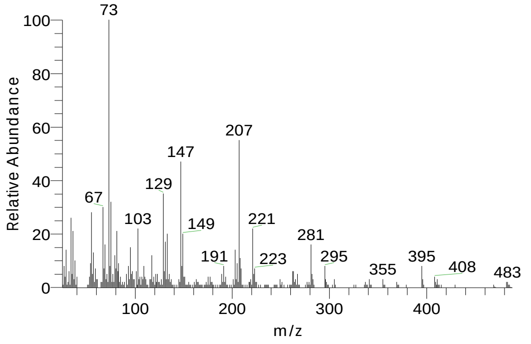

TRISTRIMETHYLSILYL ETHER DERIVATIVE OF 1,25-DIHYDROXYVITAMIN D2
Formula C37H68O3Si3, MW 644, CAS# NA, Entry# 299431

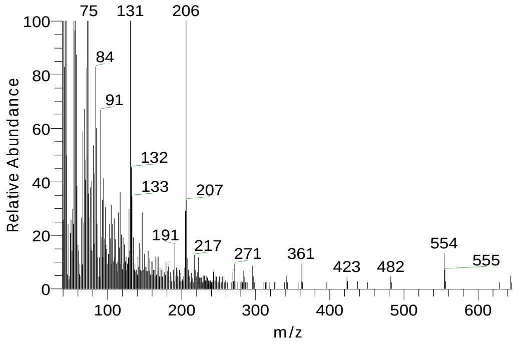

Ethyl iso-allocholate
Formula C26H44O5, MW 436, CAS# NA, Entry# 7020
Ethyl 3,7,12-trihydroxycholan-24-oate #

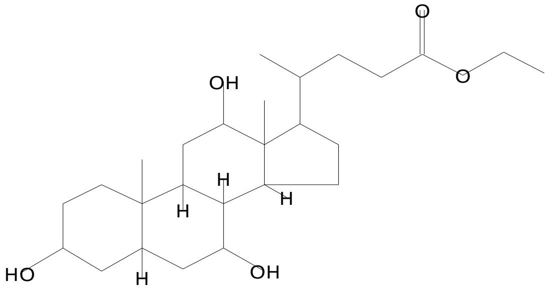

SI 704, RSI 706, mainlib, Entry# 7020, CAS# NA, Ethyl iso-allocholate

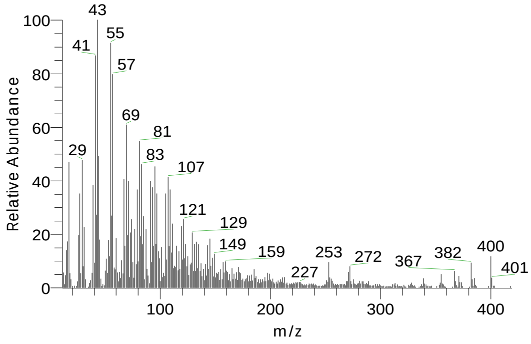

ETHYL ISO-ALLOCHOLATE
Formula C26H44O5, MW 436, CAS# NA, Entry# 270212

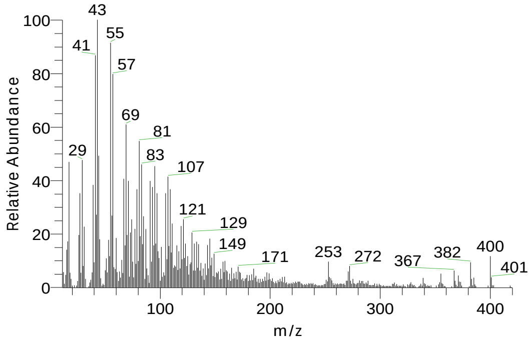

My GC-MS Report

Compound Structure | Hit Spectrum

1-Heptatriacotanol
Formula C37H76O, MW 536, CAS# 105794-58-9, Entry# 7279
1-Heptatriacontanol #

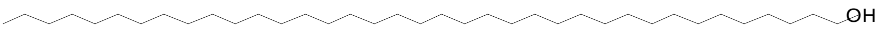

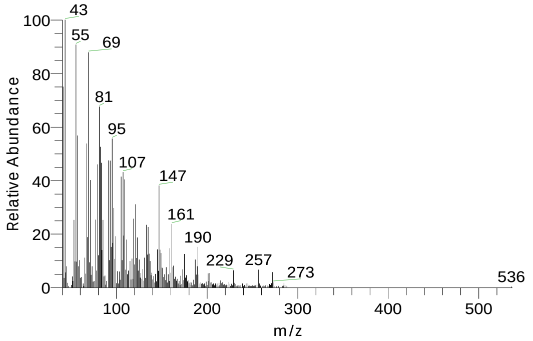

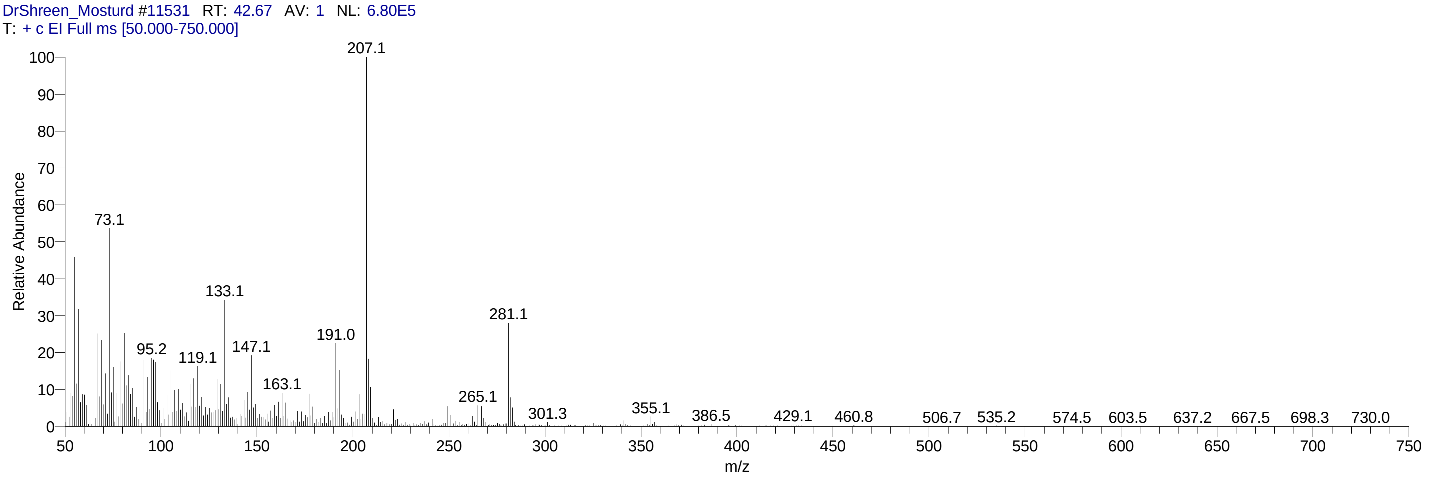

RT	Compound Name	Area %	MF	Molecular Formula	Molecular Weight	Cas #	Library
42.67	9,12-OCTADECADIENOIC ACID (Z,Z)-, 2,3-BIS[(TRIMETHYLSILYL)OXY]PROPYL ESTER	0.42	770	C27H54O4Si2	498	54284-45-6	WileyRegistry8e
42.67	TRISTRIMETHYLSILYL ETHER DERIVATIVE OF 1,25-DIHYDROXYVITAMIN D2	0.42	718	C37H68O3Si3	644	NA	WileyRegistry8e
42.67	4H-1-BENZOPYRAN-4-ONE, 2-(3,4-DIMETHOXYPHENYL)-3,5-DIHYDROXY-7-METHOXY-	0.42	691	C18H16O7	344	6068-80-0	WileyRegistry8e
42.67	1,25-Dihydroxyvitamin D3, TMS derivative	0.42	750	C30H52O3Si	488	55759-94-9	mainlib
42.67	9,10-SECOCHOLESTA-5,7,10(19)-TRIENE-1,3-DIOL, 25-[(TRIMETHYLSILYL)OXY]-, (3á,5Z,7E)-	0.42	750	C30H52O3Si	488	55759-94-9	WileyRegistry8e

Compound Structure | Hit Spectrum

9,12-OCTADECADIENOIC ACID (Z,Z)-, 2,3-BIS[(TRIMETHYLSILYL)OXY]PROPYL ESTER
Formula C27H54O4Si2, MW 498, CAS# 54284-45-6, Entry# 285148
2,3-BIS[(TRIMETHYLSILYL)OXY]PROPYL (9Z,12Z)-9,12-OCTADECADIENOATE #

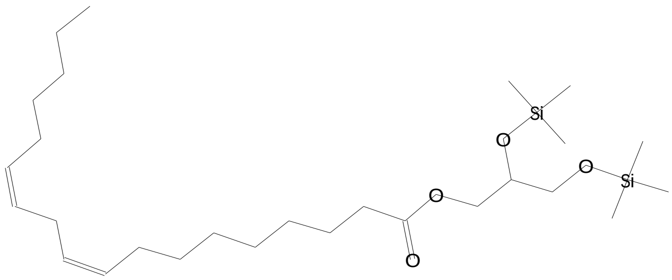

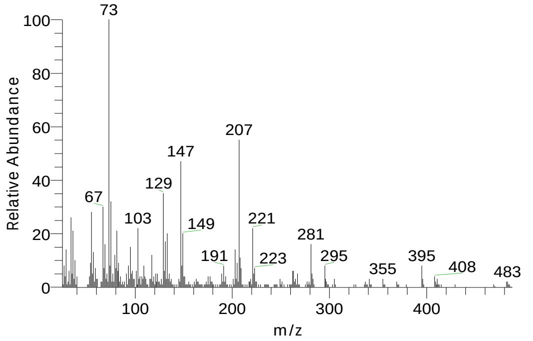

My GC-MS Report

Compound Structure | Hit Spectrum

TRISTRIMETHYLSILYL ETHER DERIVATIVE OF 1,25-DIHYDROXYVITAMIN D2
Formula C37H68O3Si3, MW 644, CAS# NA, Entry# 299431

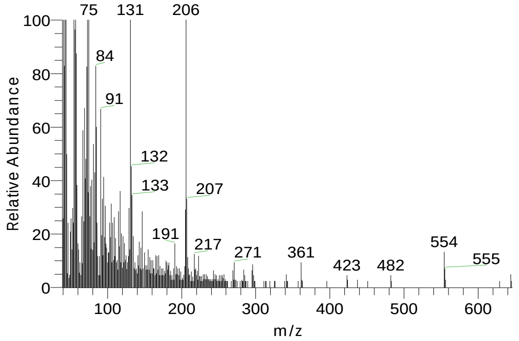

4H-1-BENZOPYRAN-4-ONE, 2-(3,4-DIMETHOXYPHENYL)-3,5-DIHYDROXY-7-METHOXY-
Formula C18H16O7, MW 344, CAS# 6068-80-0, Entry# 224392
3',4',7-TRIMETHYLQUERCETIN

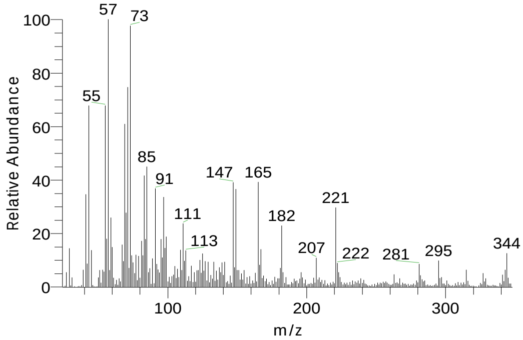

1,25-Dihydroxyvitamin D3, TMS derivative
Formula C30H52O3Si, MW 488, CAS# 55759-94-9, Entry# 19952
9,10-Secocholesta-5,7,10(19)-triene-1,3-diol, 25-[(trimethylsilyl)oxy]-, (3á,5Z,7E)-

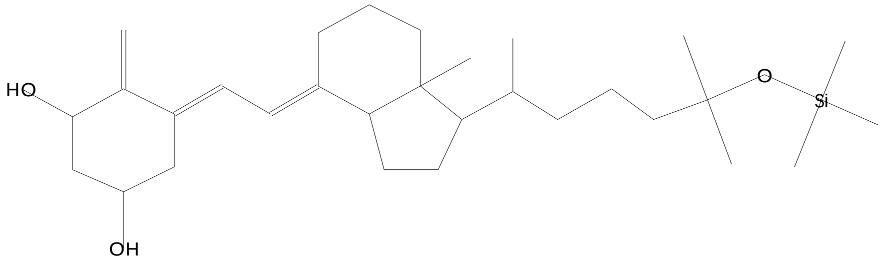

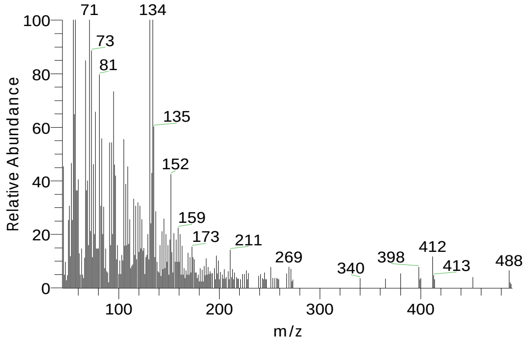

9,10-SECOCHOLESTA-5,7,10(19)-TRIENE-1,3-DIOL, 25-[(TRIMETHYLSILYL)OXY]-, (3á,5Z,7E)-
Formula C30H52O3Si, MW 488, CAS# 55759-94-9, Entry# 283552
(5E,7E)-25-[(TRIMETHYLSILYL)OXY]-9,10-SECOCHOLESTA-5,7,10-TRIENE-1,3-DIOL #

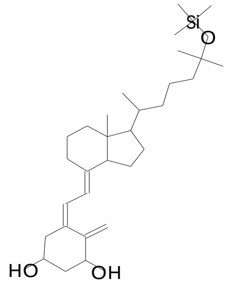

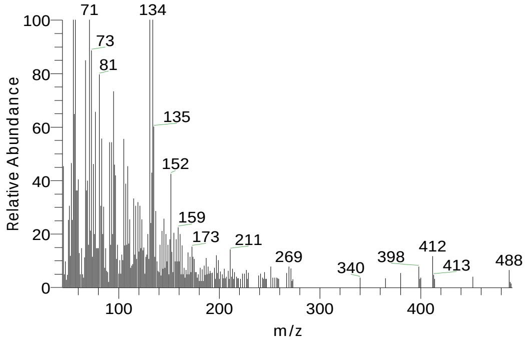

My GC-MS Report

DrShreen_Mosturd #11628 RT: 42.99 AV: 1 NL: 6.81E5
T: + c EI Full ms [50.000-750.000]

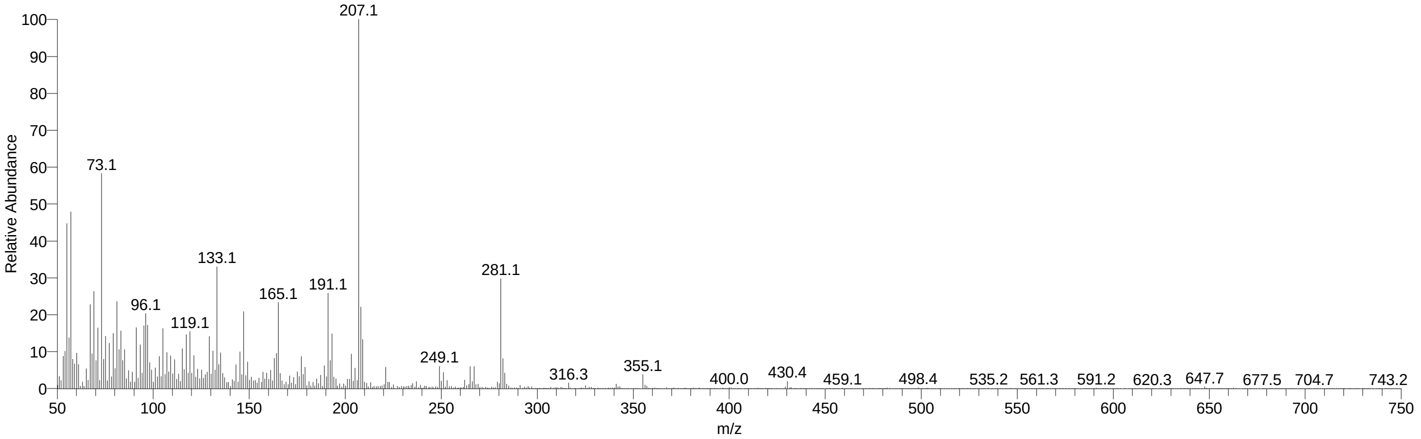

RT	Compound Name	Area %	MF	Molecular Formula	Molecular Weight	Cas #	Library
42.99	9,12-OCTADECADIENOIC ACID (Z,Z)-, 2,3-BIS[(TRIMETHYLSILYL)OXY]PROPYL ESTER	1.10	775	C27H54O4Si2	498	54284-45-6	WileyRegistry8e
42.99	TRISTRIMETHYLSILYL ETHER DERIVATIVE OF 1,25-DIHYDROXYVITAMIN D2	1.10	711	C37H68O3Si3	644	NA	WileyRegistry8e
42.99	4H-1-BENZOPYRAN-4-ONE, 2-(3,4-DIMETHOXYPHENYL)-3,5-DIHYDROXY-7-METHOXY-	1.10	696	C18H16O7	344	6068-80-0	WileyRegistry8e
42.99	1,25-Dihydroxyvitamin D3, TMS derivative	1.10	740	C30H52O3Si	488	55759-94-9	mainlib
42.99	9,10-SECOCHOLESTA-5,7,10(19)-TRIENE-1,3-DIOL, 25-[(TRIMETHYLSILYL)OXY]-, (3á,5Z,7E)-	1.10	740	C30H52O3Si	488	55759-94-9	WileyRegistry8e

Compound Structure | Hit Spectrum

9,12-OCTADECADIENOIC ACID (Z,Z)-, 2,3-BIS[(TRIMETHYLSILYL)OXY]PROPYL ESTER
Formula C27H54O4Si2, MW 498, CAS# 54284-45-6, Entry# 285148
2,3-BIS[(TRIMETHYLSILYL)OXY]PROPYL (9Z,12Z)-9,12-OCTADECADIENOATE #

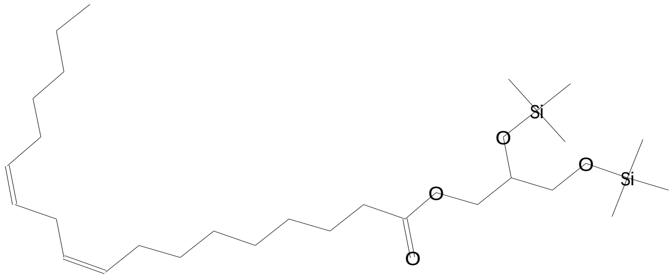

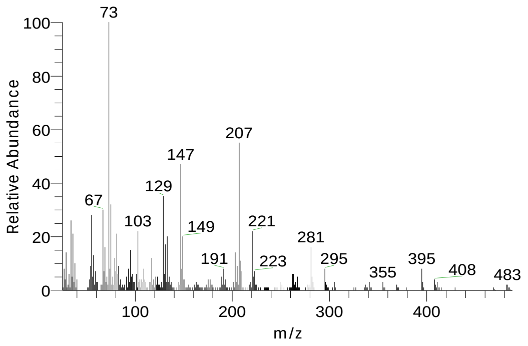

TRISTRIMETHYLSILYL ETHER DERIVATIVE OF 1,25-DIHYDROXYVITAMIN D2
Formula C37H68O3Si3, MW 644, CAS# NA, Entry# 299431

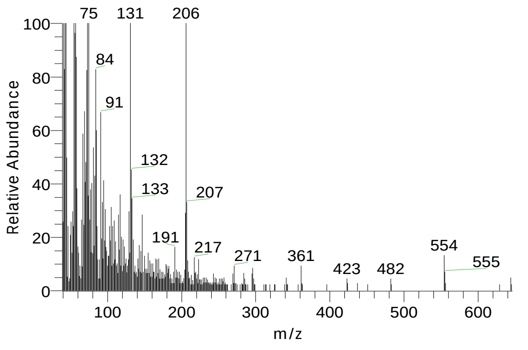

My GC-MS Report

Compound Structure	Hit Spectrum

4H-1-BENZOPYRAN-4-ONE, 2-(3,4-DIMETHOXYPHENYL)-3,5-DIHYDROXY-7-METHOXY-
Formula C18H16O7, MW 344, CAS# 6068-80-0, Entry# 224392
3',4',7-TRIMETHYLQUERCETIN

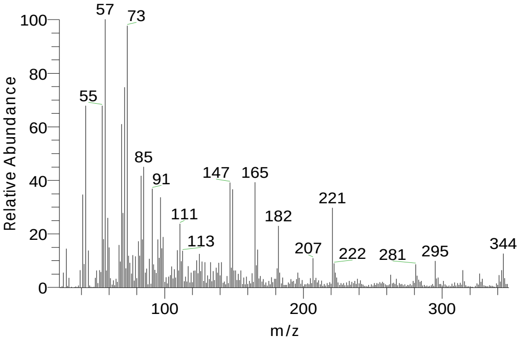

1,25-Dihydroxyvitamin D3, TMS derivative
Formula C30H52O3Si, MW 488, CAS# 55759-94-9, Entry# 19952
9,10-Secocholesta-5,7,10(19)-triene-1,3-diol, 25-[(trimethylsilyl)oxy]-, (3á,5Z,7E)-

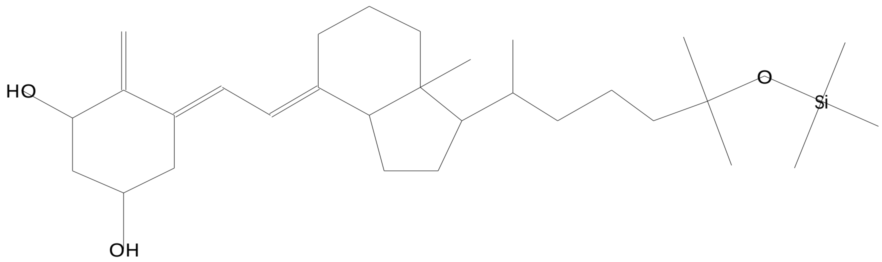

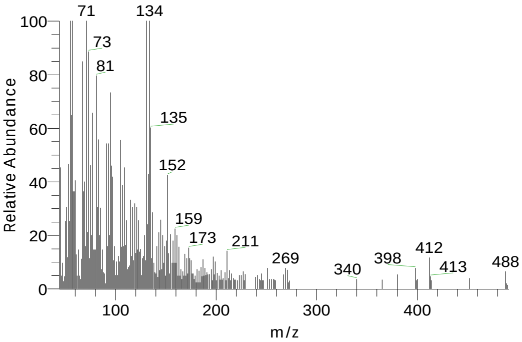

9,10-SECOCHOLESTA-5,7,10(19)-TRIENE-1,3-DIOL, 25-[(TRIMETHYLSILYL)OXY]-, (3á,5Z,7E)-
Formula C30H52O3Si, MW 488, CAS# 55759-94-9, Entry# 283552
(5E,7E)-25-[(TRIMETHYLSILYL)OXY]-9,10-SECOCHOLESTA-5,7,10-TRIENE-1,3-DIOL #

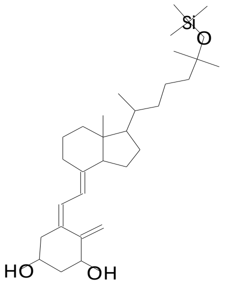

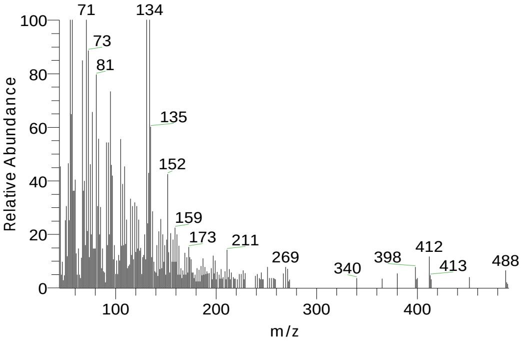

DrShreen_Mosturd #11912 RT: 43.95 AV: 1 NL: 1.49E6
T: + c EI Full ms [50.000-750.000]

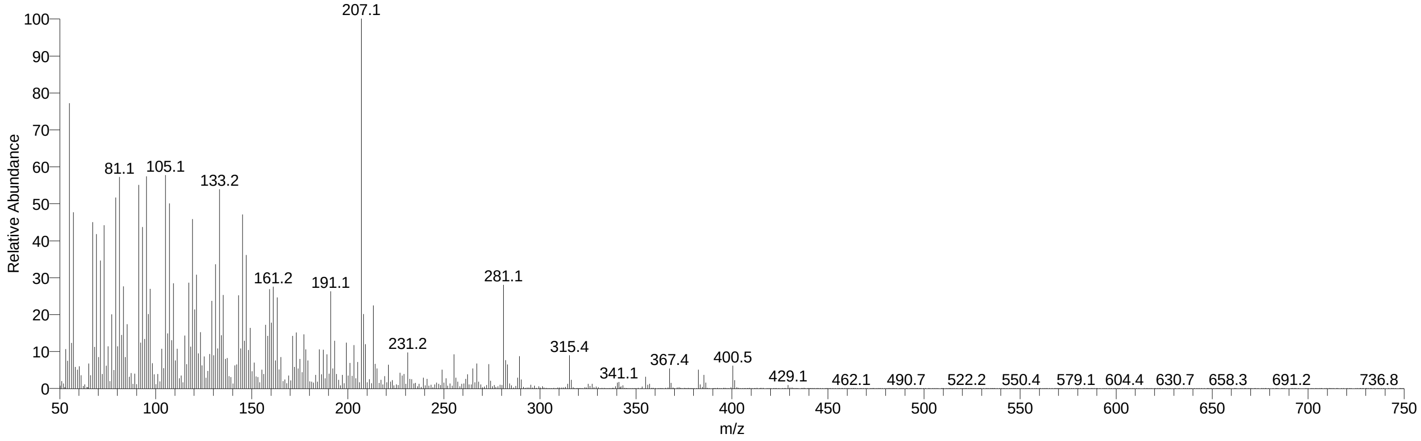

RT	Compound Name	Area %	MF	Molecular Formula	Molecular Weight	Cas #	Library
43.95	Campesterol	12.58	796	C28H48O	400	474-62-4	mainlib
43.95	ERGOST-5-EN-3-OL	12.58	796	C28H48O	400	NA	WileyRegistry8e

My GC-MS Report

RT	Compound Name	Area %	MF	Molecular Formula	Molecular Weight	Cas #	Library
43.95	ERGOST-5-EN-3-OL, (3á)-	12.58	796	C28H48O	400	4651-51-8	WileyRegistry8e
43.95	5-Cholestene-3-ol, 24-methyl-	12.58	802	C28H48O	400	290299-12-6	mainlib
43.95	ERGOST-5-EN-3-OL, (3á,24R)-	12.58	902	C28H48O	400	474-62-4	WileyRegistry8e

Compound Structure | Hit Spectrum

Campesterol
Formula C28H48O, MW 400, CAS# 474-62-4, Entry# 7079
Ergost-5-en-3-ol, (3á,24R)-

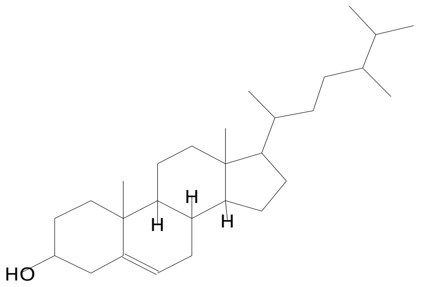

SI 788, RSI 796, mainlib, Entry# 7079, CAS# 474-62-4, Campesterol

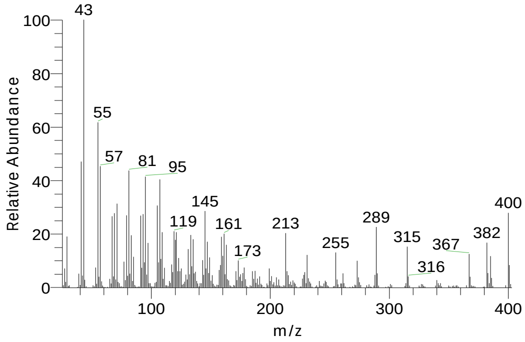

ERGOST-5-EN-3-OL
Formula C28H48O, MW 400, CAS# NA, Entry# 387447
ERGOST-5-EN-3B-OL

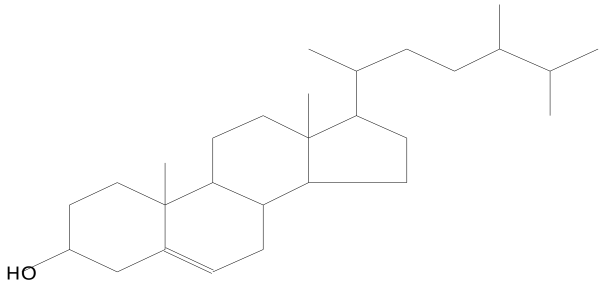

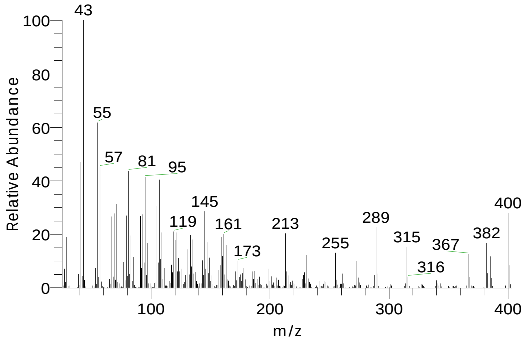

ERGOST-5-EN-3-OL, (3á)-
Formula C28H48O, MW 400, CAS# 4651-51-8, Entry# 256351
ERGOST-5-EN-3-OL #

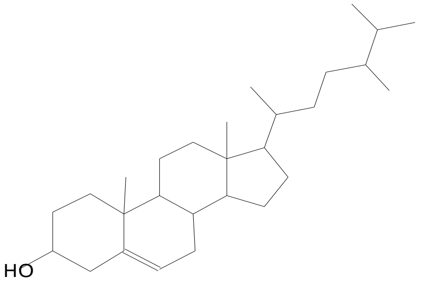

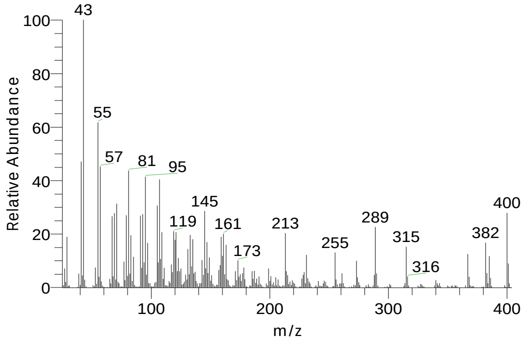

5-Cholestene-3-ol, 24-methyl-
Formula C28H48O, MW 400, CAS# 290299-12-6, Entry# 7172
Ergost-5-en-3-ol #

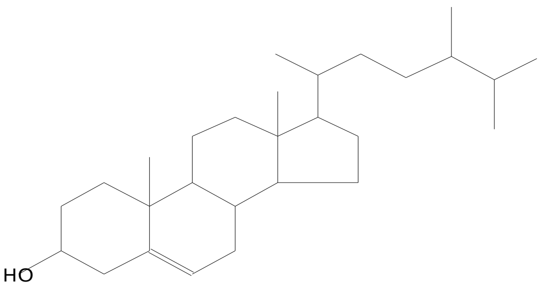

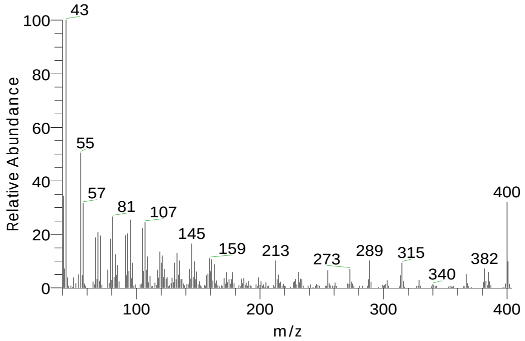

My GC-MS Report

Compound Structure | Hit Spectrum

ERGOST-5-EN-3-OL, (3á,24R)-
Formula C28H48O, MW 400, CAS# 474-62-4, Entry# 396001
ERGOST-5-EN-3-OL #

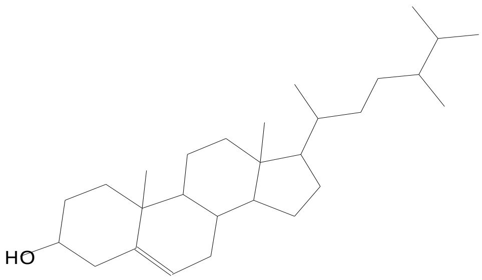

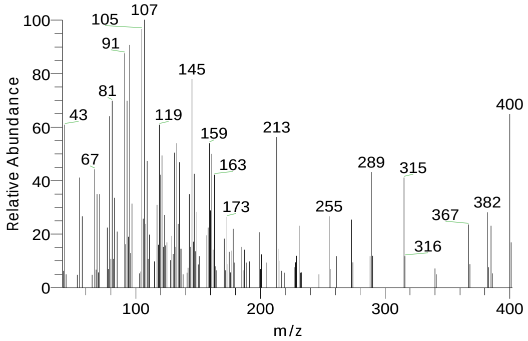

DrShreen_Mosturd #12001 RT: 44.24 AV: 1 NL: 1.85E6
T: + c EI Full ms [50.000-750.000]

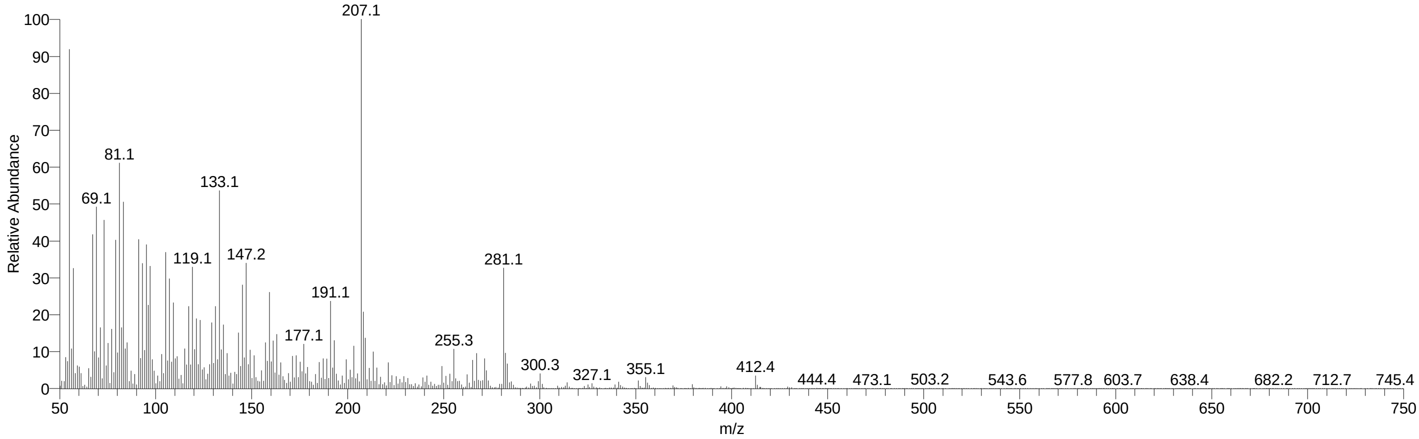

RT	Compound Name	Area %	MF	Molecular Formula	Molecular Weight	Cas #	Library
44.24	Stigmasterol	9.75	757	C29H48O	412	83-48-7	replib
44.24	Stigmasterol	9.75	812	C29H48O	412	83-48-7	mainlib
44.24	STIGMASTA-5,22-DIEN-3-OL, (3á,22E)-	9.75	760	C29H48O	412	83-48-7	WileyRegistry8e
44.24	STIGMASTA-5,22-DIEN-3-OL	9.75	781	C29H48O	412	NA	WileyRegistry8e
44.24	Cholesta-22,24-dien-5-ol, 4,4-dimethyl-	9.75	795	C29H48O	412	NA	mainlib

Compound Structure | Hit Spectrum

Stigmasterol
Formula C29H48O, MW 412, CAS# 83-48-7, Entry# 5167
Stigmasta-5,22-dien-3-ol, (3á,22E)-

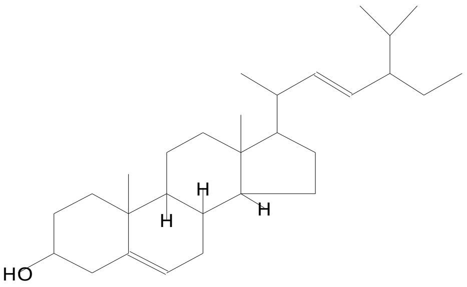

SI 755, RSI 757, replib, Entry# 5167, CAS# 83-48-7, Stigmasterol

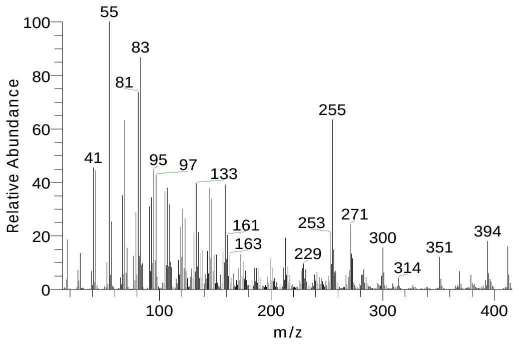

My GC-MS Report

Compound Structure | Hit Spectrum

Stigmasterol
Formula C29H48O, MW 412, CAS# 83-48-7, Entry# 20820
Stigmasta-5,22-dien-3-ol, (3á,22E)-

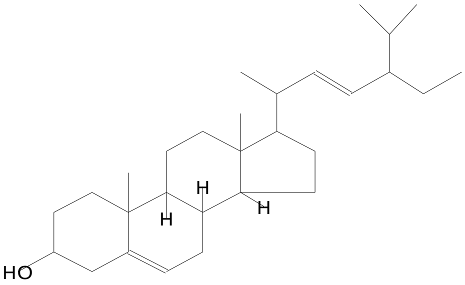

SI 753, RSI 812, mainlib, Entry# 20820, CAS# 83-48-7, Stigmasterol

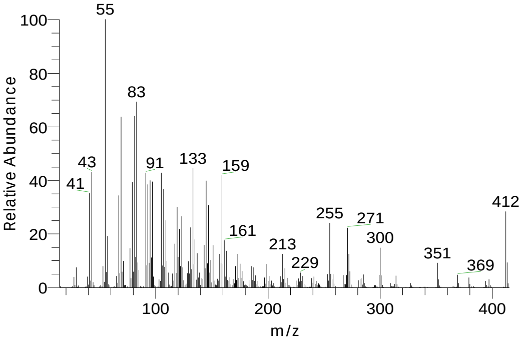

STIGMASTA-5,22-DIEN-3-OL, (3á,22E)-
Formula C29H48O, MW 412, CAS# 83-48-7, Entry# 261297
(22E)-STIGMASTA-5,22-DIEN-3-OL #

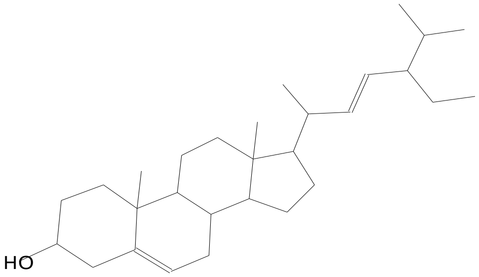

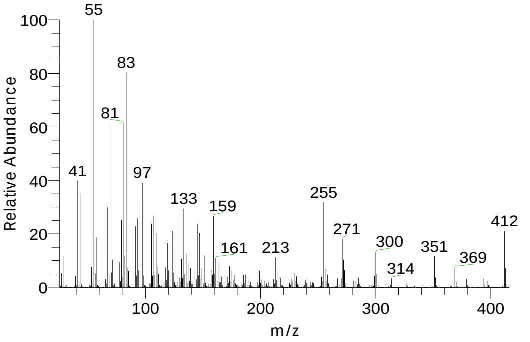

STIGMASTA-5,22-DIEN-3-OL
Formula C29H48O, MW 412, CAS# NA, Entry# 383444
STIGMASTA-5,22E-DIEN-3B-OL

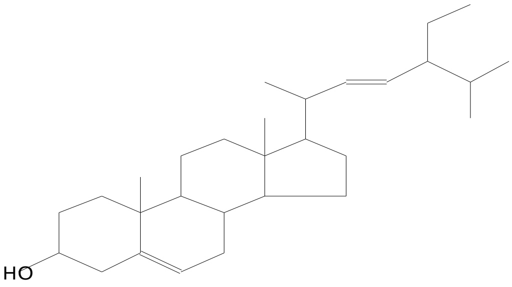

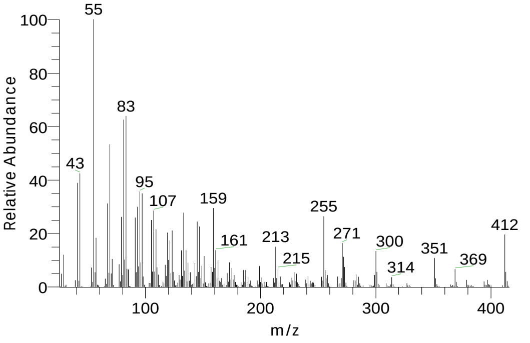

Cholesta-22,24-dien-5-ol, 4,4-dimethyl-
Formula C29H48O, MW 412, CAS# NA, Entry# 19679
(22E)-4,4-Dimethylcholesta-22,24-dien-6-ol #

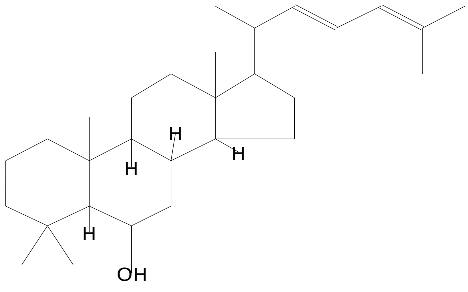

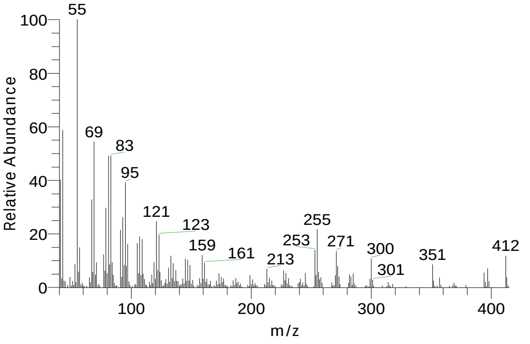

My GC-MS Report

DrShreen_Mosturd #12158 RT: 44.77 AV: 1 NL: 3.91E6
T: + c EI Full ms [50.000-750.000]

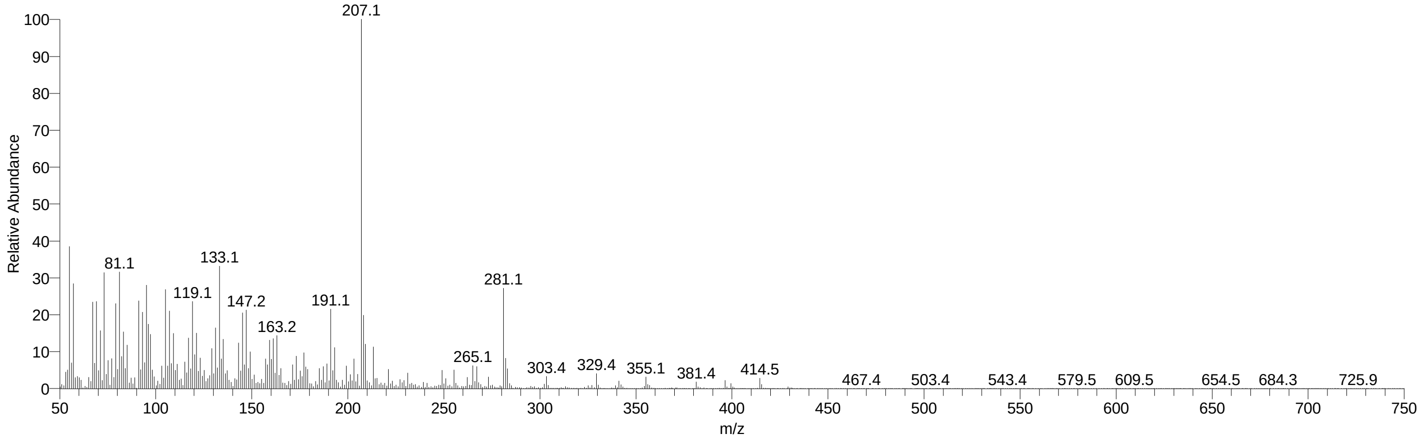

RT	Compound Name	Area %	MF	Molecular Formula	Molecular Weight	Cas #	Library
44.77	á-Sitosterol	20.34	782	C29H50O	414	83-46-5	replib
44.77	9,12-OCTADECADIENOIC ACID (Z,Z)-, 2,3-BIS[(TRIMETHYLSILYL)OXY]PROPYL ESTER	20.34	767	C27H54O4Si2	498	54284-45-6	WileyRegistry8e
44.77	ç-Sitosterol	20.34	735	C29H50O	414	83-47-6	mainlib
44.77	STIGMAST-5-EN-3-OL, (3á,24S)-	20.34	735	C29H50O	414	83-47-6	WileyRegistry8e
44.77	STIGMAST-5-EN-3-OL, (3á,24S)-	20.34	733	C29H50O	414	83-47-6	WileyRegistry8e

Compound Structure | Hit Spectrum

á-Sitosterol
Formula C29H50O, MW 414, CAS# 83-46-5, Entry# 2073
Stigmast-5-en-3-ol, (3á)-

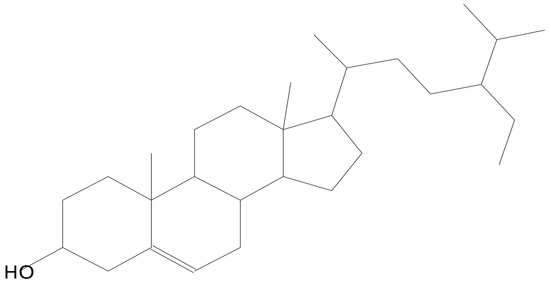

SI 731, RSI 782, replib, Entry# 2073, CAS# 83-46-5, á-Sitosterol

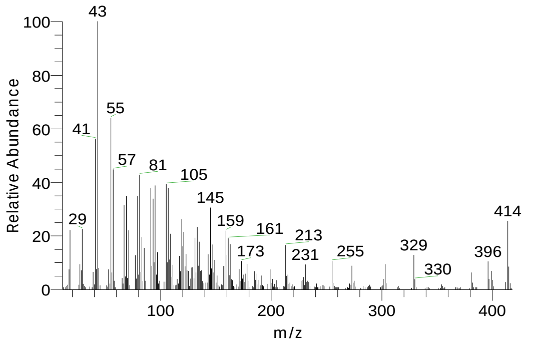

9,12-OCTADECADIENOIC ACID (Z,Z)-, 2,3-BIS[(TRIMETHYLSILYL)OXY]PROPYL ESTER
Formula C27H54O4Si2, MW 498, CAS# 54284-45-6, Entry# 285148
2,3-BIS[(TRIMETHYLSILYL)OXY]PROPYL (9Z,12Z)-9,12-OCTADECADIENOATE #

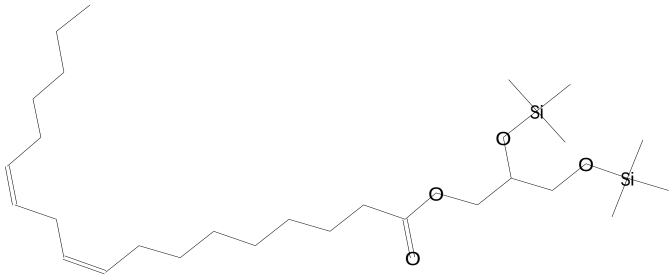

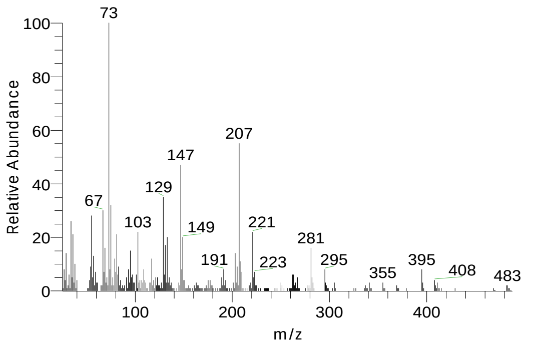

My GC-MS Report

Compound Structure

Hit Spectrum

ç-Sitosterol
Formula C29H50O, MW 414, CAS# 83-47-6, Entry# 7212
Stigmast-5-en-3-ol, (3á,24S)-

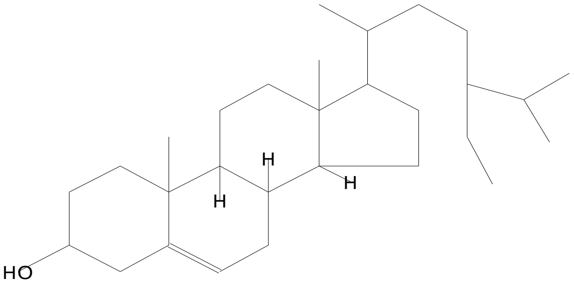

SI 724, RSI 735, mainlib, Entry# 7212, CAS# 83-47-6, ç-Sitosterol

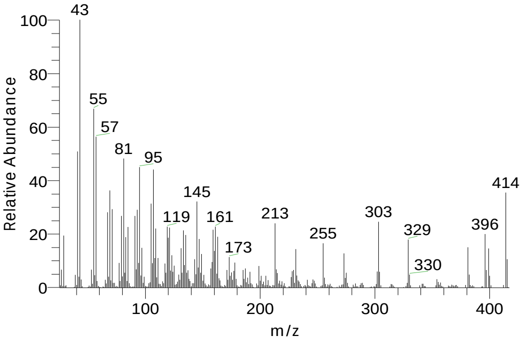

STIGMAST-5-EN-3-OL, (3á,24S)-
Formula C29H50O, MW 414, CAS# 83-47-6, Entry# 387446
STIGMAST-5-EN-3-OL #

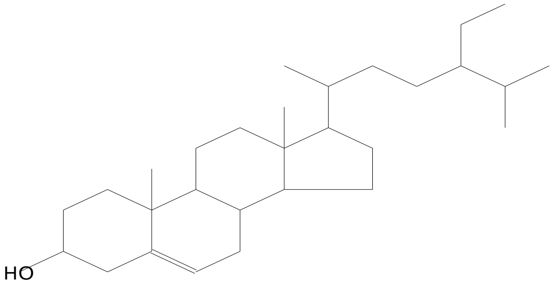

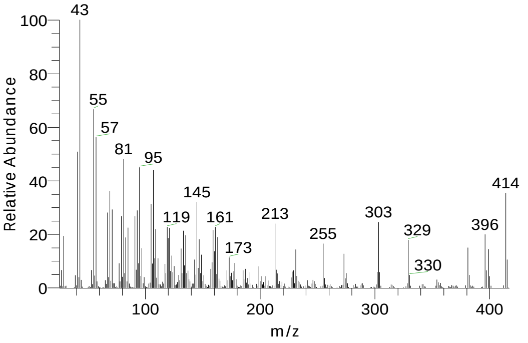

STIGMAST-5-EN-3-OL, (3á,24S)-
Formula C29H50O, MW 414, CAS# 83-47-6, Entry# 262357
STIGMAST-5-EN-3-OL #

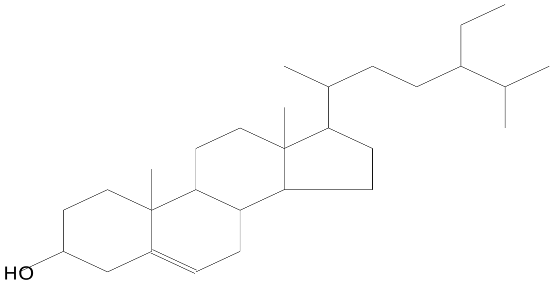

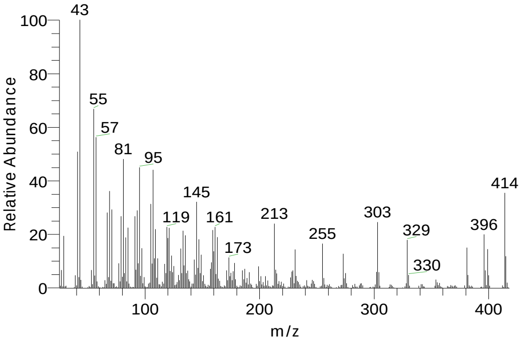

Supplement: Supplementary file 4 — Supplementary Material 4 [file 41598_2025_25896_MOESM4_ESM.pdf]
